# Mendelian randomization integrated with multi-omics analysis identifies TNIK as a key gene in gut microbiota-induced IBD development

**DOI:** 10.3389/fimmu.2025.1678444

**Published:** 2025-11-18

**Authors:** Xin Chai, Hongli Wang, Boxiang Wang, Yanchun Ma, Xiaoyan Zhang, Jing Guo, Shuping Luo, Yan Wang, Jinpeng Hong, Qiang Ma, Jiayu Chen, Biaomeng Wang, Yixuan Wang

**Affiliations:** 1 Department of Emergency, An Ning Attached Medical Area, The 940th Hospital of Joint Logistics Support Force of Chinese People's Liberation Army (PLA), Lanzhou, China; 2 Graduate School of Gansu University of Traditional Chinese Medicine, Lanzhou, China; 3 Department of Gastroenterology, The 940th Hospital of Joint Logistics Support Force of Chinese People's Liberation Army (PLA), Lanzhou, China

**Keywords:** inflammatory bowel disease, gut microbiota, mendelian randomization, TNIK, multi-omics integration, immune-epithelial crosstalk

## Abstract

**Background:**

Dysbiosis of the gut microbiota (GM) has been linked to inflammatory bowel disease (IBD), yet its associated molecular mechanisms remain poorly defined. Identifying causal host genes mediating GM-IBD interactions is therefore of great importance.

**Objective:**

To identify GM-associated causal genes for IBD and to prioritize key targets and cell types underlying GM-host crosstalk.

**Methods:**

We integrated GWAS datasets of GM, UC, and CD using a two-sample Mendelian randomization (MR) framework with IVW as the primary estimator. Causal SNPs were mapped to genes for enrichment analyses. Candidate genes were refined by intersecting MR-derived genes with bulk RNA-seq DEGs (training: GSE87473, validation: GSE75214) and prioritized using nested cross-validated machine-learning models. Single-cell RNA-seq (GSE116222) was used to localize key genes to specific cell types. The functional role of TNIK was validated in IL-10-/- IBD mice via AAV9-mediated overexpression. Immunohistochemical staining of Ki67 and Cleaved caspase 3 was conducted to evaluate epithelial proliferation and apoptosis in colonic tissues.

**Results:**

MR analysis identified 307 and 360 GM-associated causal genes for UC and CD, respectively. TNIK (TRAF2 and NCK-interacting kinase) was highlighted as a key candidate gene. Seven TNIK-associated immune cell subsets showed altered infiltration in UC. Single-cell transcriptomics revealed TNIK dysregulation in colonocytes, goblet cells. T/NK cells in UC. TNIK overexpression in IL-10^-/-^ mice reduced disease severity and downregulated IL-1β, IL-6, and TNF-α. Immunohistochemistry confirmed that TNIK overexpression enhanced Ki67 expression and reduced Cleaved caspase 3 expression.

**Conclusion:**

By integrating MR with transcriptomics and single-cell seq results, we identified TNIK as a potential GM-associated host kinase linking dysbiosis to epithelial and immune dysfunction in IBD. TNIK emerges as a promising node for IBD prognosis through barrier maintenance and immune regulation.

## Introduction

Inflammatory bowel disease (IBD) is recognized as a chronic, relapsing inflammatory disorder of the gastrointestinal tract, encompassing primarily ulcerative colitis (UC) and Crohn’s disease (CD) (1). Previous researches indicated that the pathogenesis of IBD involves genetic predisposition, dysregulated intestinal immune responses, and environmental factors (2, 3). Current therapies aim mainly to control inflammation and induce remission, but no curative therapy is available (4–7). Existing treatments exhibit limited success rates and notable drawbacks (8).

Recent studies have suggested that imbalances in gut microbiota (GM) disrupt mucosal homeostasis and promote aberrant inflammatory responses, leading to the initiation and progression of IBD (9–11). Patients with IBD consistently show GM dysbiosis, characterized by disrupted microbial composition and diversity (12–14). Typical features include reduced microbial richness, depletion of health-promoting microbes (e.g., short-chain fatty acid-producing bacteria), and overgrowth of potentially pathogenic species (15). For instance, CD patients exhibit significantly reduced abundance of the anti-inflammatory commensal bacterium *Faecalibacterium prausnitzii*, while opportunistic pathogens showed abnormal proliferation within the intestinal environment of IBD gut (16–18). Accordingly, therapeutic strategies targeting GM modulation have gained attention for IBD intervention, including probiotic administration and fecal microbiota transplantation (FMT) (19). Nevertheless, the molecular pathways through which gut microbes modulate the intestinal milieu of IBD patients are far from fully elucidated.

Mendelian randomization (MR) is a genetic approach for causal inference that uses genetic variants associated with an exposure as instrumental variables (IVs) to evaluate the causal relationship between the exposure and disease outcomes, thereby reducing the influence of confounding factors and reverse causation to some extent. Previous studies have applied MR to investigate inflammatory bowel disease (IBD) in relation to periodontitis (20), atrial fibrillation (21), and plasma caffeine concentration (22). In recent years, several studies have applied MR to investigate the causal relationship between the GM and IBD, but most have focused on the microbial level (23–25). However, the molecular regulatory mechanisms linking GM and the host remain unclear.

Based on this background, we performed a two-sample MR analysis and integrated bulk RNA-seq data with machine-learning approaches to identify potential key nodes. We then conducted immune infiltration analysis and single-cell RNA-seq to characterize their cellular heterogeneity. Finally, vivo experiments were performed to validate the candidate nodes. By uncovering key microbial–host molecular pathways, this study may provide novel targets for more effective and personalized therapeutic strategies in IBD.

## Materials and methods

### Study design

**Figure 1:**
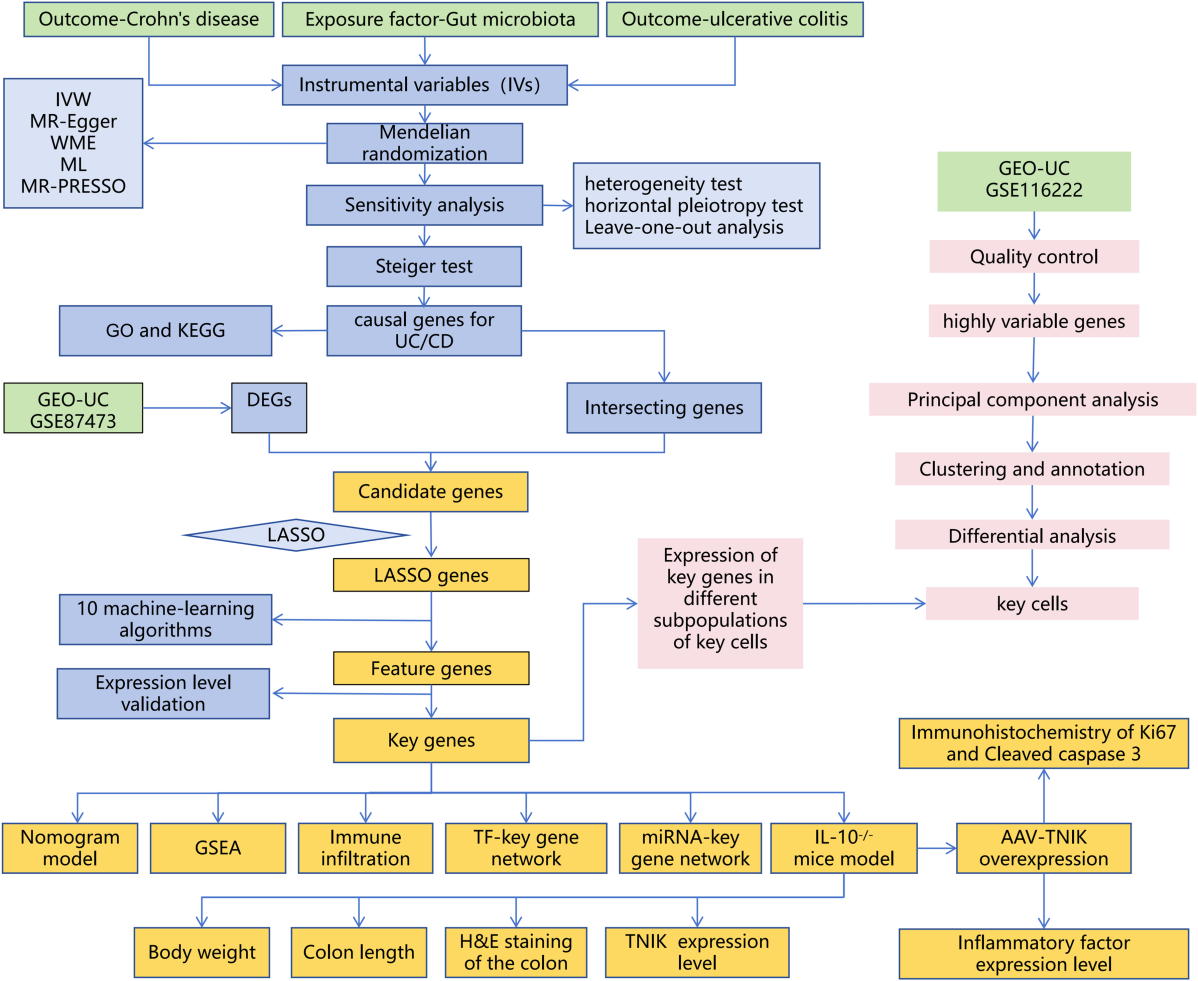
Overall workflow of the study. The study integrates MR, bulk RNA-seq, sc RNA-seq, and animal experiments.

The experimental design of this study is shown in **Figure 1**.

### Data acquisition

GM data were retrieved from the MiBioGen consortium database (26), comprising 211 microbial classifications with 14, 587 associated single nucleotide polymorphisms (SNPs). The genome-wide association study (GWAS) data for UC and Crohn’s disease (CD) were sourced from the IEU OpenGWAS database (27). The UC data were obtained from the “EBI-A-GCST90018933” dataset (European population, 5, 371 cases and 412, 561 controls, 24, 187, 301 SNPs). The CD data were obtained from the “IEU-A-30” dataset (5, 956 cases and 14, 927 controls, 12, 276, 506 SNPs). The bulk RNA-seq datasets included GSE87473 (28) (UC = 106, controls = 21, used as the training set) and GSE75214 (29) (UC = 74, controls = 11, used as the validation set). GSE92415 (30) (placebo-treated UC patients = 53, controls = 21) was used for correlation analysis with the Mayo score. The scRNA-seq dataset selected was GSE116222 (31) (UC = 3, controls = 3).

### Mendelian randomization

GM was treated as the exposure factor, while UC and CD served as outcome variables. The MR analysis was based on three principal assumptions: (1) The selected IVs are closely linked to the exposure variable. (2) IVs exert their effect on the endpoint uniquely through the exposure mechanism. (3) The IVs are not confounded by external factors. Five MR methods were applied: Inverse Variance Weighted (IVW), weighted median, MR-Egger, weighted mode, and simple mode. Among these, IVW was adopted as the primary method to identify causal microbial associations (*P* < 0.05) (32). Cochran’s Q test was used to evaluate heterogeneity (33), while the Egger intercept and MR-PRESSO were employed to evaluate horizontal pleiotropy (34). The leave-one-out (LOO) method was applied to verify the stability of MR estimates, and the Steiger’s approach for directional causality assessment was implemented to verify the cause-effect relationship. Finally, the identified SNPs were mapped to their corresponding genes, followed by biological functional enrichment analysis (GO and KEGG pathway).

### Selection of IVs

To identify IVs associated with the exposure factor, SNPs related to five taxonomic levels of gut bacterial traits were extracted from the GWAS database extraction of IVs was performed with the extract_instruments function from TwoSampleMR (v0.6.8). Independent SNPs linked to the exposure factor were determined according to the following filtering thresholds: (1) *P* < 1×10^−5^ (35, 36). (2) R^2^ < 0.001, kb = 10 (to avoid bias from LD). (3) SNPs exhibiting p-value significance with the endpoint were filtered out (proxies = TRUE, rsq = 0.8). (4) Weak Instrument Filtering (F < 10), the F-statistic was calculated as: F=R^2^(N-K-1)/[K(1-R)^2^)], with *R²* being the sum of variance explained by the chosen SNPs, *K* representing SNP quantity, and *N* referring to the GWAS population size. (5) Palindromic SNPs and exposure factors with fewer than 3 SNPs were excluded. Finally, we mapped the selected SNPs to their corresponding genes using the gprofiler2 (v0.2.3) package for downstream functional analyses.

### Identification of differentially expressed genes and screening of candidate genes

DEG analysis in the GSE87473 dataset was conducted with the R package limma (v3.62.0). Thresholds of |log_2_FC| > 0.5 and adjusted p-value < 0.05 were applied, and the results were visualized using ggplot2 (v3.5.1) and pheatmap (v1.0.12). Subsequently, overlapping genes were identified by intersecting the DEGs with UC causal genes and CD causal genes. The candidate genes were further refined using LASSO regression implemented in the R package glmnet (v4.1.8), with 10-fold cross-validation applied to select the most predictive candidates.

### Screening of key genes

The mlr3 (v0.22.1.9000) R package was employed to construct an ensemble machine learning model, in which ten distinct algorithms were integrated for training and evaluation. Subsequently, the pROC (v1.18.5) package was used to generate ROC curves and compute area under the curve (AUC) values, enabling the identification of optimal-performing models for candidate gene analysis and key gene selection. To assess model stability, 10 repetitions of 10-fold cross-validation were performed. ROC curves were plotted for each model, and the mean AUC values from repeated cross-validation were reported. For each model, the AUC, sensitivity, and specificity values were recorded. Models with all metrics > 0.8 and AUC ≠ 1 were considered stable and well-performing and were designated as “optimal models.”. Genes retained in the optimal models were defined as feature genes. To assess the clinical relevance of the key genes, Spearman correlation analysis was performed using the psych (v2.4.12) package on the GSE92415 dataset, evaluating their relationship with the Mayo score. Correlations with |cor| > 0.3 and *P* < 0.05 were considered statistically meaningful. Finally, the key genes expressions were validated across the GSE87473 and GSE75214 datasets using the Wilcoxon rank-sum test to ensure consistent differential expression trends.

### Construction and evaluation of the nomogram model

To estimate the probability of key genes predicting IBD risk, a nomogram model was developed using the rms (v7.0.0) R package in the training set GSE87473. The model assigned scores according to expression levels of selected genes. The aggregated score was utilized to estimate diagnostic probability. The final model was formulated as follows: Logit(P)=15.9996+(1.0108×TMEM163) + (−2.7965×TNIK). Where P represents the predicted probability of UC. Subsequently, the calibrate function from the rms (v7.0.0) package was employed to plot calibration curves in the training set GSE87473, assessing the predictive accuracy of the nomogram for IBD occurrence. To assess the clinical applicability of the nomogram model, decision curve analysis (DCA) was conducted via the ggDCA (v1.2) package in the training set GSE87473, illustrating the net benefit (Net Benefit) of different models under varying gene combinations. Finally, the pROC package applied to generate ROC curves for both the nomogram and individual genes, with the AUC computed to assess predictive effectiveness.

### Immune infiltration analysis

To profile the immunological microenvironment in IBD, immune cell infiltration proportions in UC and normal control samples from the training set GSE87473 were calculated using CIBERSORT (*P* < 0.05). Immune cells exhibiting significant alterations were identified using the Wilcoxon rank-sum test (*P* < 0.05). Subsequently, Spearman correlations between key genes and differentially infiltrated immune cells in GSE87473 were analyzed using the psych (v2.4.12) package, with statistical significance defined as |cor| > 0.3 and *P* < 0.05.

### Single-cell transcriptome processing and identification of key cell types

To elucidate the distribution and functional significance of key genes across cellular subpopulations, the GSE116222 dataset was processed using the Seurat (v5.1.0) package’s CreateSeuratObject function. The analysis incorporated these quality assurance parameters: (1) Number of genes detected per cell (nFeature_RNA): 200–8, 000. (2) Total UMI counts per cell (nCount_RNA): 200–30, 000. (3) Mitochondrial gene proportion (percent.mt): < 20%. (4) Gene inclusion threshold: detected in ≥ 3 cells. The top 2, 000 highly variable genes (HVGs) were selected for principal component analysis (PCA). Cell clustering was performed using the top 30 PCs through the FindClusters function, with subsequent visualization via UMAP (RunUMAP). Cluster - specific marker genes were identified via the FindMarkers function (Seurat v5.1.0), with thresholds of log_2_FC > 1 and adjusted *P*-value ≤ 0.05. Cell type annotation was carried out based on the CellMarker database and relevant literature references. Differentially enriched cell populations were identified by calculating the odds ratio (OR) across samples. Gene ontology enrichment of these cell types was carried out employing the analyze_sc_clusters function provided by the ReactomeGSA (v1.20.0) package. Finally, key cell populations were identified by analyzing the expression profiles of key genes across differentially enriched clusters using the Seurat package.

### Animals

Sixteen-week-old male BALB/cAnCya and IL-10^-/-^ (BALB/c-Il10 KO) mice were purchased from Cyagen Biosciences Inc. (USA). All mice were acclimatized for 1–2 weeks and housed under standardized conditions (23 ± 2 °C, 55 ± 5% humidity, 12-hour light/dark cycle) (37). All animal experiments were carried out under approved guidelines with oversight from the Ethics Committee of the 940th Hospital of Joint Logistics Support Force of Chinese PLA (approval number: 2023KYLL028). Since body weight change serves as a key indicator of intestinal health, we monitored the mice’s body weight during the experimental period (20–22 weeks of age). Upon study completion, mice were euthanized by cervical dislocation performed by trained personnel and complete colon tissues were collected for length measurement.

### Construction of recombinant virus

AAV9, widely utilized in intestinal research for its high transduction efficiency, was employed to construct recombinant viruses in this study (38). Since AAV-mediated gene expression typically begins 1–2 weeks after injection and IL-10^−^/^−^ mice develop severe phenotypes by 22 weeks of age, the mice were divided into two groups at 20 weeks of age (n = 8 per group) and were administered AAV-EGFP or AAV-TNIK via tail vein injection.

### Hematoxylin and eosin staining

H&E staining was performed in accordance with the manufacturer’s instructions (Beyotime, China). The isolated distal colon was first fixed in 4% paraformaldehyde, followed by stepwise dehydration in ethanol solutions of progressively higher concentrations (70%, 80%, 90%, and 100%), with 1-hour treatment durations, followed by xylene clearing (three changes, 1 hour each), and subsequently embedded in paraffin. Paraffin-embedded tissues were sliced into 7 μm sections, processed with H&E staining, and examined under bright-field microscopy. The scoring criteria included inflammatory cell infiltration, mucosal edema, crypt swelling and disruption, and epithelial cell damage (39). Each parameter was graded on a scale of 0–3 according to the extent and severity of pathological changes, and the total histological score was calculated as the sum of all parameters.

### qRT-PCR

Total RNA was isolated from colon tissues using the TRIzol reagent (Invitrogen, USA). GAPDH served as the internal control gene. All reactions were conducted in triplicate. We analyzed the comparative expression of selected genes using the 2^-ΔΔCt^ method. The primer sequences were as follows: TNIK, F-5’-CTGCTCGTTGACCTCACAGT-3’, R-5’-CCTGGTGGTCTCTTAAAATGCAA-3’. GAPDH, F-5’-TGTGTCCGTCGTGGaTCTGA-3’, R-5’-TTGCTGTTGAAGTCGCAGGAG-3’.

### Western blot

Proteins from colon tissues were extracted using RIPA lysis buffer (Beyotime, China), and quantification of protein levels was performed via the BCA method (Servicebio, China). After SDS-PAGE separation, proteins were transferred via semi-dry transfer and subsequently blocked with blocking solution (Beyotime, China) for 1 hour. Membranes were incubated overnight at 4 °C with primary antibodies: anti-TNIK (ab308194, Abcam), anti-IL-1β (ab2105, Abcam), anti-IL-6 (ab9324, Abcam), anti-TNFα (ab1793, Abcam), and anti-GAPDH (ab9485, Abcam). Then, Goat Anti-Rabbit IgG H&L (HRP) (ab6721, Abcam) or Goat Anti-Mouse IgG H&L (HRP) (ab205719, Abcam) were incubated at room temperature for 1 hour. ECL reagent was applied for immunoblot signal detection (Solarbio, China).

### Immunohistochemistry

Colon tissues were fixed in 4% paraformaldehyde (PFA, Servicebio, China), dehydrated, embedded in paraffin, and sectioned at a thickness of 4 μm. The sections were deparaffinized in xylene and rehydrated through graded ethanol solutions. Antigen retrieval was performed by boiling the sections in 10 mM sodium citrate buffer (pH 6.0, Servicebio, China) for 15 min. Endogenous peroxidase activity was quenched with 3% hydrogen peroxide (Sinopharm Chemical Reagent, China), followed by blocking with 5% bovine serum albumin (BSA, Sigma–Aldrich, USA) for 30 min at room temperature. The sections were then incubated overnight at 4 °C with the following primary antibodies: Ki67 (ab15580, Abcam, UK) and Cleaved caspase 3 (#9661, Cell Signaling Technology, USA). After washing with PBS, the sections were incubated with an HRP-conjugated goat anti-rabbit secondary antibody (Servicebio, China) for 1 h at room temperature. Immunoreactivity was visualized using a DAB detection kit (Servicebio, China), and nuclei were counterstained with hematoxylin (Solarbio, China). Finally, the slides were dehydrated, mounted with neutral resin (Solarbio, China), and examined under a Leica DM2000 light microscope (Leica Microsystems, Germany).

### Statistical analysis

Statistical computations were performed with R (v4.2.0) and GraphPad Prism 9.0. For MR analysis, multiple testing correction was performed using the Hochberg method. For experimental data analysis, continuous variables were expressed as the mean ± standard deviation (SD). Normality of data distribution was assessed using the Shapiro–Wilk test. For comparisons between two groups, an unpaired two-tailed Student’s t-test was used for normally distributed data, whereas the Wilcoxon rank-sum test was applied for nonparametric data. For comparisons among multiple groups, a two-way ANOVA followed by Tukey’s *post hoc* test was conducted. All statistical tests were two-tailed, and results were considered statistically significant at *P < 0.05, **P < 0.01, and ***P < 0.001.

## Results

### Selection and validation of instrumental SNPs for MR

In accordance with the methodology described above, a total of 965 SNPs were obtained, all of which showed F-statistics > 10, indicating no weak instrument bias. These selected SNPs met the relevance assumption required for MR analysis. Only the top 10 SNPs, ranked by F-statistics, are presented in **Table 1**, while the full list is provided in **Supplementary Table 1**.

**Table 1:** SNPs obtained by screening.

SNP	chr	pos	se	Exposure	Pval	R	F
rs182549	2	1.37E+08	0.012729	genus.Bifidobacterium.id.436	5.27E-21	0.069275	88.42999
rs182549	2	1.37E+08	0.012066	class.Actinobacteria.id.419	2.47E-20	0.068075	85.37717
rs182549	2	1.37E+08	0.01267	family.Bifidobacteriaceae.id.433	2.47E-20	0.068073	85.37167
rs182549	2	1.37E+08	0.01267	order.Bifidobacteriales.id.432	2.47E-20	0.068073	85.37167
rs6754311	2	1.37E+08	0.012708	genus.Bifidobacterium.id.436	3.29E-20	0.067848	84.8072
rs6730157	2	1.36E+08	0.011909	class.Actinobacteria.id.419	1.72E-19	0.066533	81.53636
rs7570971	2	1.36E+08	0.012575	genus.Bifidobacterium.id.436	2.01E-19	0.066409	81.23261
rs7570971	2	1.36E+08	0.012518	family.Bifidobacteriaceae.id.433	2.52E-19	0.066225	80.78078
rs7570971	2	1.36E+08	0.012518	order.Bifidobacteriales.id.432	2.52E-19	0.066225	80.78078
rs6754311	2	1.37E+08	0.012047	class.Actinobacteria.id.419	2.94E-19	0.066102	80.47834

### Causal relationships between GM and IBD

**Figure 2:**
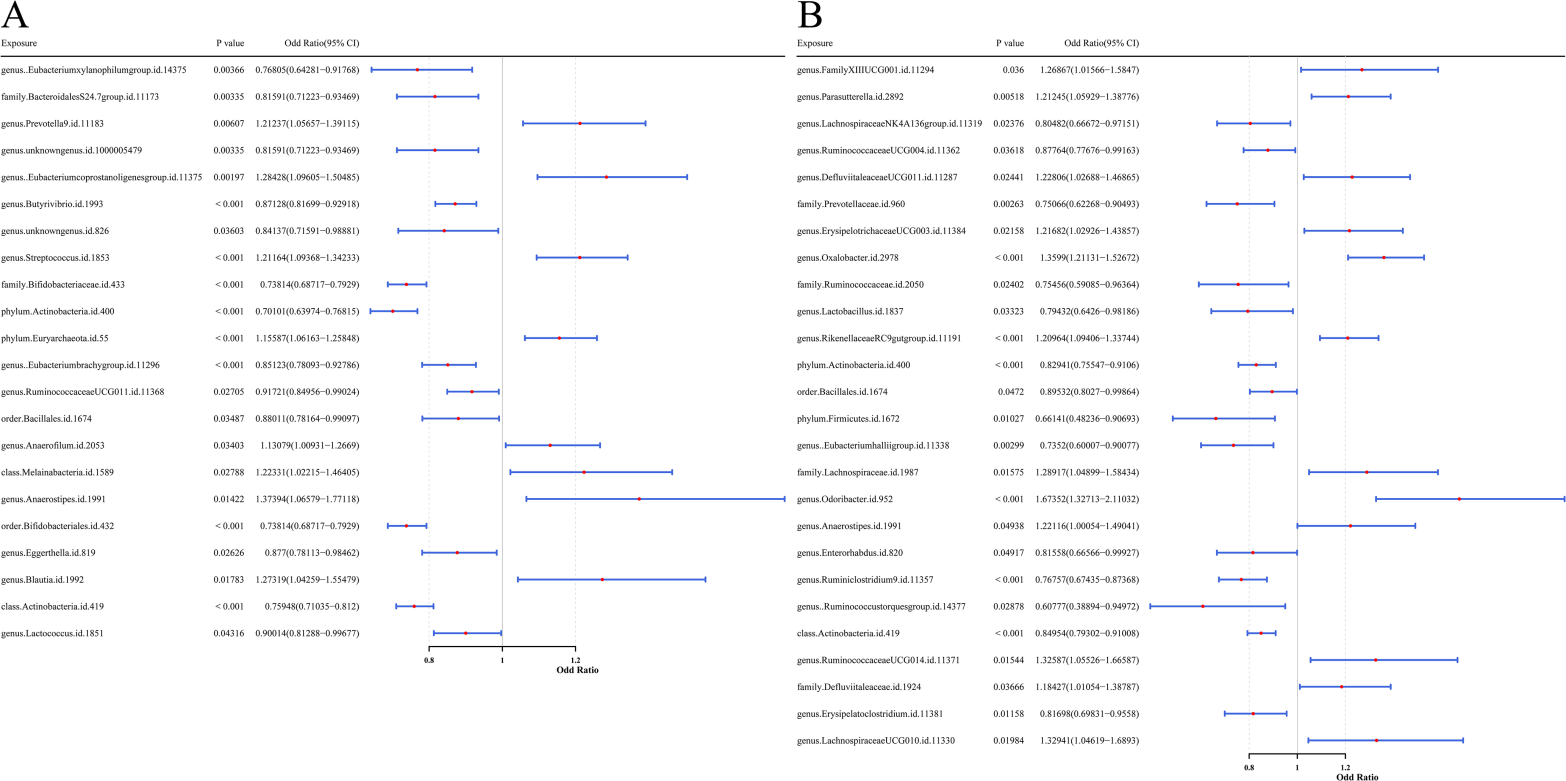
Forest plots presenting the predictive results of causal relationships from MR analysis. **(A)** Results with GM as the exposure factor and UC as the outcome variable. **(B)** Results with GM as the exposure factor and CD as the outcome variable. Each plot illustrates the OR with 95% CI, providing evidence for potential causal associations between microbial features and disease outcomes.

By MR analysis, based on the IVW method, 22 gut microbial taxa significantly linked to UC (*P* < 0.05, **Figure 2A**), including 8 risk factors (OR > 1, 95% confidence interval [CI] > 1) and 14 protective factors (OR < 1, 95% CI < 1). Meanwhile, 26 intestinal flora were found to be significantly causally associated with CD (*P* < 0.05), including 12 risk factors and 14 protective factors (**Figure 2B**). Detailed causal effect estimates between specific gut microbial taxa and IBD types were shown in **Supplementary Figure 1**.

For protective factors, the SNP effect estimates were less than 0, whereas for risk factors, the effect estimates were greater than 0 (**Supplementary Figure 2**). The SNP distribution of gut microbial taxa was symmetrical and evenly distributed, indicating that the MR analysis complied with Mendel’s second law (**Supplementary Figure 3**). The Cochran’s Q test analysis showed that heterogeneity (*P* < 0.05) was observed in seven UC-associated microbial taxa (family Bifidobacteriaceae, order Bifidobacteriales, genus Anaerostipes, class Melainabacteria, class Actinobacteria, phylum Actinobacteria, and order Bacillales) and two CD-associated taxa (genus Ruminococcustorquesgroup and phylum Firmicutes). These variables were modeled accounting for random heterogeneity effects, while the remaining taxa were analyzed using a fixed-effects model (**Supplementary Table 2**).

In addition, *P*-values from pleiotropy tests were all above 0.05, suggesting the absence of horizontal pleiotropy and thereby reinforcing the validity of the MR analysis (**Supplementary Table 3**). The results of LOO analysis showed that individual SNPs did not exert a dominant effect on the outcome variables, confirming the robustness of the causal inference (**Supplementary Figure 4**). The Steiger directionality test also confirmed that the MR analyses were not disturbed by reverse causality (*P* < 0.05, **Supplementary Table 4**).

In summary, 22 gut microbial taxa were significantly causally associated with UC. Mapping their corresponding SNPs to genes, a total of 307 UC-related microbial causal genes were obtained. Similarly, 26 taxa showed significant causal relationship with CD, and a total of 360 flora causal genes related to CD were obtained after SNP mapping. All SNPs met criteria (2) and (3) of the MR analysis.

### Functional and pathway enrichment analysis of GM-associated causal genes

**Figure 3:**
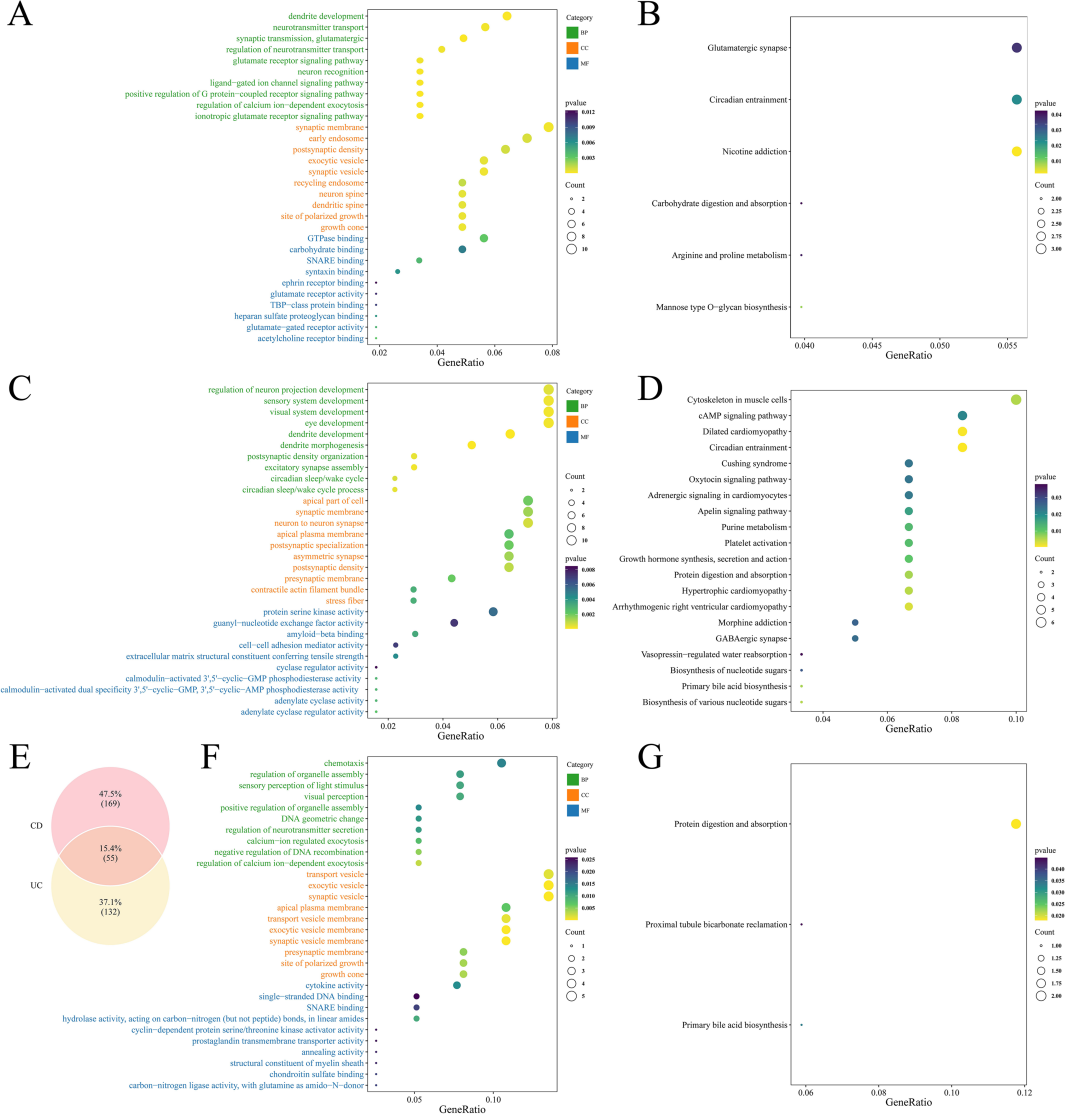
Functional enrichment result plots of causal genes. **(A)** GO enrichment plot of GM associated with UC causal genes. **(B)** KEGG enrichment plot of GM associated with UC causal genes. **(C)** GO enrichment plot of GM associated with CD causal genes. **(D)** KEGG enrichment plot of GM associated with CD causal genes. **(E)** Venn diagram showing overlapping genes between UC- and CD-associated microbial causal genes. **(F)** GO enrichment plot of intersecting genes. **(G)** KEGG enrichment plot of intersecting genes.

GO enrichment analysis of the 307 UC-related microbial causal genes identified 237 significant pathways (*P* < 0.05, **Supplementary Table 5**), mainly involving dendritic development, neurotransmitter transport, and synaptic vesicle function (**Figure 3A**). KEGG pathways were significantly enriched in glutamatergic synapse, circadian rhythm, and metabolic processes (**Figure 3B**).

The 360 CD-related genes were enriched to 278 GO terms (*P* < 0.05, **Supplementary Table 5**), highlighting neural projection development, sensory system regulation, and kinase activity (**Figure 3C**). KEGG pathways were significantly enriched in cytoskeletal organization and the cAMP signaling pathway (**Figure 3D**).

Intersections were taken for causal genes of UC and CD, yielding a total of 55 intersecting genes (**Figure 3E**). GO enrichment involved a total of 179 terms (**Supplementary Table 6**), of which BP included cell chemotaxis, regulation of organelle assembly, visual perception, etc., CC included transport vesicles, synaptic vesicles, apical plasma membrane, etc., and MF included cytokine activity, DNA binding, etc. (**Figure 3F**). KEGG was enriched to three pathways: protein digestion and absorption, renal tubular bicarbonate recycling and primary bile acid synthesis (**Figure 3G**).

### TMEM163 and TNIK were identified as characterized genes

**Figure 4:**
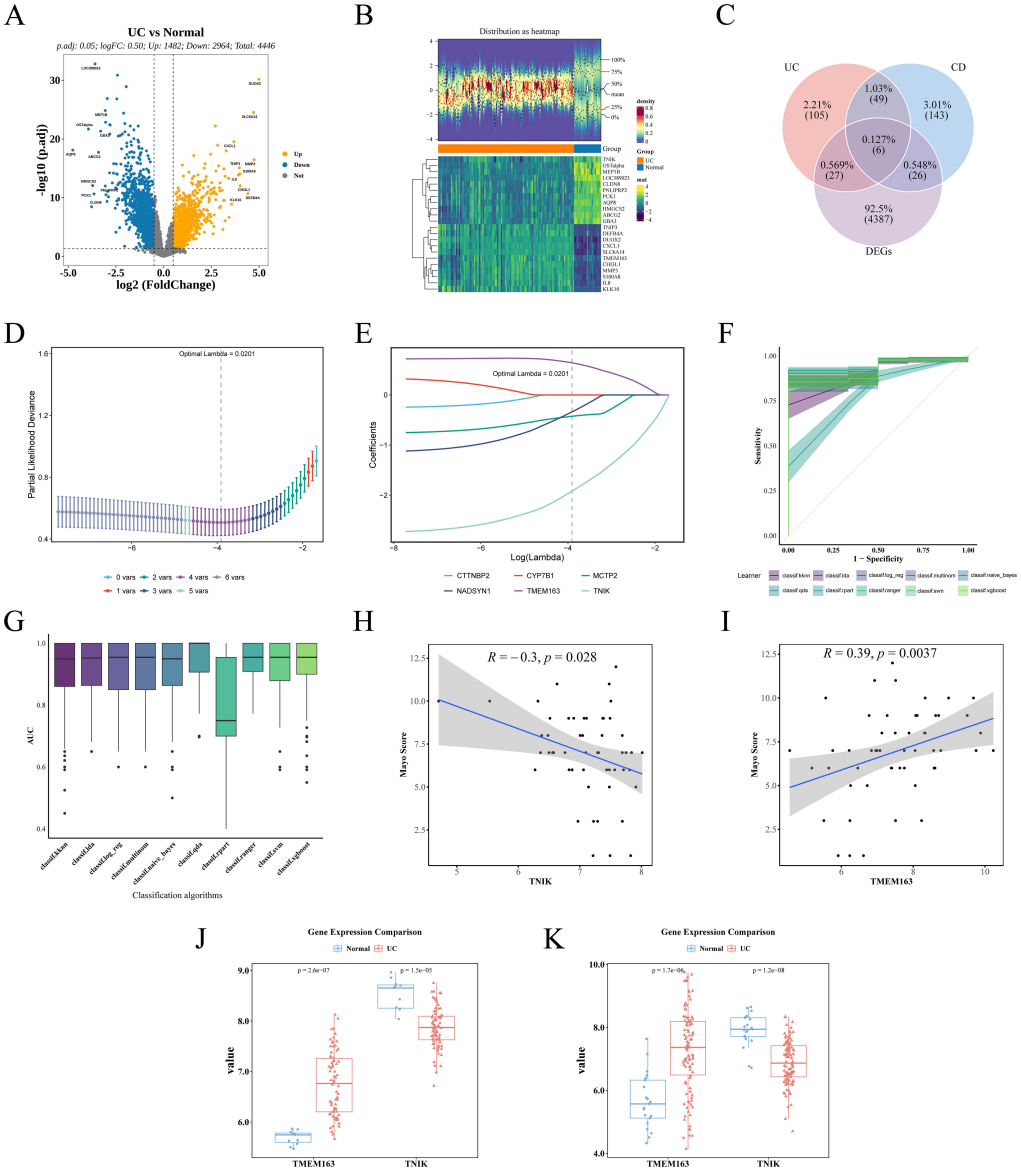
Identification of key genes and validation of dataset results. **(A)** Volcano plot demonstrating DEGs. **(B)** Heat map demonstrating the expression distribution of DEGs. **(C)** Venn plot of intersecting genes of causal genes and DEGs. **(D)** Lasso coefficient path diagram. **(E)** Partial likelihood deviation coefficient plot was used to evaluate model fit. **(F)** ROC curves for the 10 machine learning models, with the shaded areas of the curves indicating the standard errors of the curves. **(G)** AUC box plot. **(H)** Spearman correlation between TNIK and Mayo score. **(I)** Spearman correlation between TMEM163 and Mayo score. **(J)** Expression trends of TMEM163 and TNIK in the training set GSE87473 (UC vs Control). **(K)** Expression trends of TMEM163 and TNIK in the validation set GSE75214 (UC vs Control).

Differential expression analysis of the GSE87473 dataset identified 4, 446 DEGs, including 1, 482 upregulated and 2, 964 downregulated genes (**Figure 4A**). A volcano plot labeled the top 10 most significant DEGs, and a heatmap depicted the expression profiles of the top 20 DEGs (**Figure 4B**). By intersecting these DEGs with GM-associated causal genes for UC and CD, six overlapping candidate genes were identified (**Figure 4C**). LASSO regression minimized error at an optimal Lambda of 0.0201, resulting in the selection of four candidate genes: TNIK, TMEM163, MCTP2, and NADSYN1 (**Figures 4D, E**). Evaluation across ten machine learning algorithms showed that the RF and QDA models yielded the highest performance in ROC analysis (**Figures 4F, G**; **Table 2**), leading to the identification of TMEM163 and TNIK as key featured genes (**Supplementary Table 7**). All four genes were significantly correlated with Mayo score (|cor| > 0.3, *P* < 0.05), with TNIK showing a negative and TMEM163 a positive correlation (**Figures 4H, I**). The Wilcoxon rank-sum test validated consistent differential expression trends in both GSE87473 and GSE75214 datasets (*P* < 0.05). TMEM163 was significantly upregulated, while TNIK was significantly downregulated in UC samples (**Figures 4J, K**).

**Table 2:** Evaluation results of machine learning model performance.

Learner	CE	AUC	Sensitivity	Specificity	FNR	FPR
RANGER	0.095183	0.955939	0.959909	0.628333	0.040091	0.371667
QDA	0.087106	0.948864	0.947182	0.738333	0.052818	0.261667
XGBOOST	0.10815	0.92203	0.952273	0.583333	0.047727	0.416667
SVM	0.093132	0.921303	0.970818	0.585	0.029182	0.415
LDA	0.093507	0.920576	0.954455	0.668333	0.045545	0.331667
LOG_REG	0.091126	0.916348	0.954455	0.681667	0.045545	0.318333
MULTINOM	0.091126	0.916348	0.954455	0.681667	0.045545	0.318333
NAIVE_BAYES	0.128855	0.91603	0.903636	0.711667	0.096364	0.288333
KKNN	0.096328	0.904447	0.963364	0.603333	0.036636	0.396667
RPART	0.131328	0.778402	0.918727	0.611667	0.081273	0.388333

### Construction of nomogram model and selection of key genes

**Figure 5:**
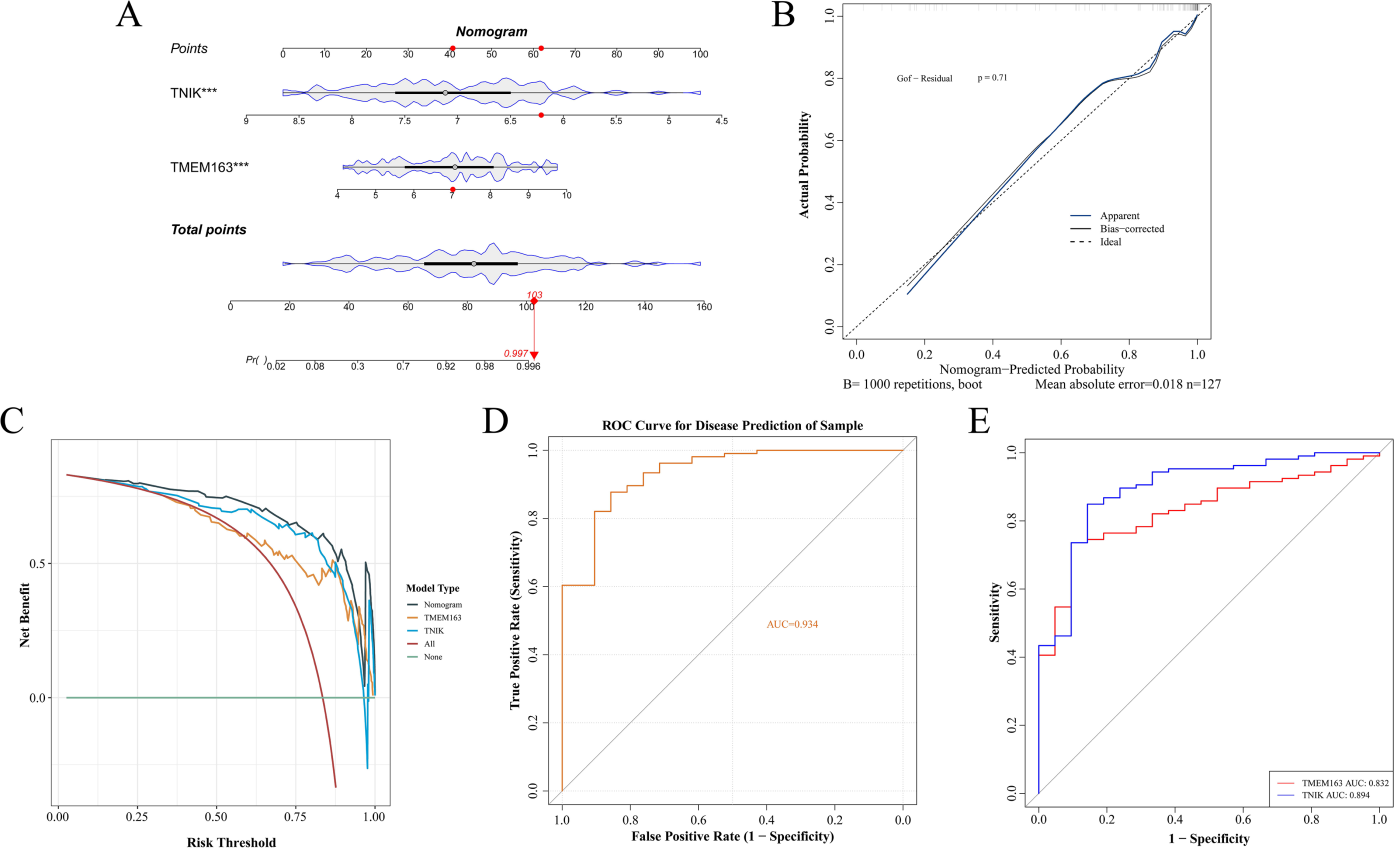
Construction of a nomogram for risk prediction. **(A)** Nomogram-based prediction model. **(B)** Calibration curve evaluating the nomogram’s predictive performance. **(C)** DCA curves of TMEM163 and TNIK. **(D)** ROC curves of the nomogram model. **(E)** ROC curves of TMEM163 and TNIK. This figure demonstrated the translational potential of incorporating molecular markers into clinical risk prediction.

To further evaluate the clinical predictive value of the identified feature genes, a nomogram model was constructed in the training dataset GSE87473 based on TMEM163 and TNIK (**Figure 5A**). The results indicated that, based on the combined expression levels of the two genes, the total score was 103, corresponding to an estimated probability of approximately 99.7% for developing IBD. Moreover, TNIK expression exerted a greater impact on the prediction, with lower expression levels associated with higher risk. TMEM163 also contributed significantly to the prediction, although its effect was less pronounced than that of TNIK.

The calibration curve indicated a small prediction error (P = 0.71) and demonstrated good model fit (**Figure 5B**). The DCA curve indicated that the net clinical benefit of the model exceeded that of single-gene predictors (**Figure 5C**), supporting its potential for clinical application. The AUC of the model reached 0.934, while AUCs of individual key genes exceeded 0.8 (**Figures 5D, E**).

Although both TNIK and TMEM163 demonstrated satisfactory predictive performance (AUC > 0.8), TNIK exhibited the highest diagnostic accuracy (AUC = 0.894). Notably, TNIK is a serine/threonine kinase implicated in immune signaling and epithelial barrier regulation, two essential processes in IBD pathogenesis. Given its mechanistic relevance and feasibility for *in vivo* validation, TNIK was selected as the primary target gene for subsequent functional studies.

### Immune infiltration analysis and regulatory network analysis

**Figure 6:**
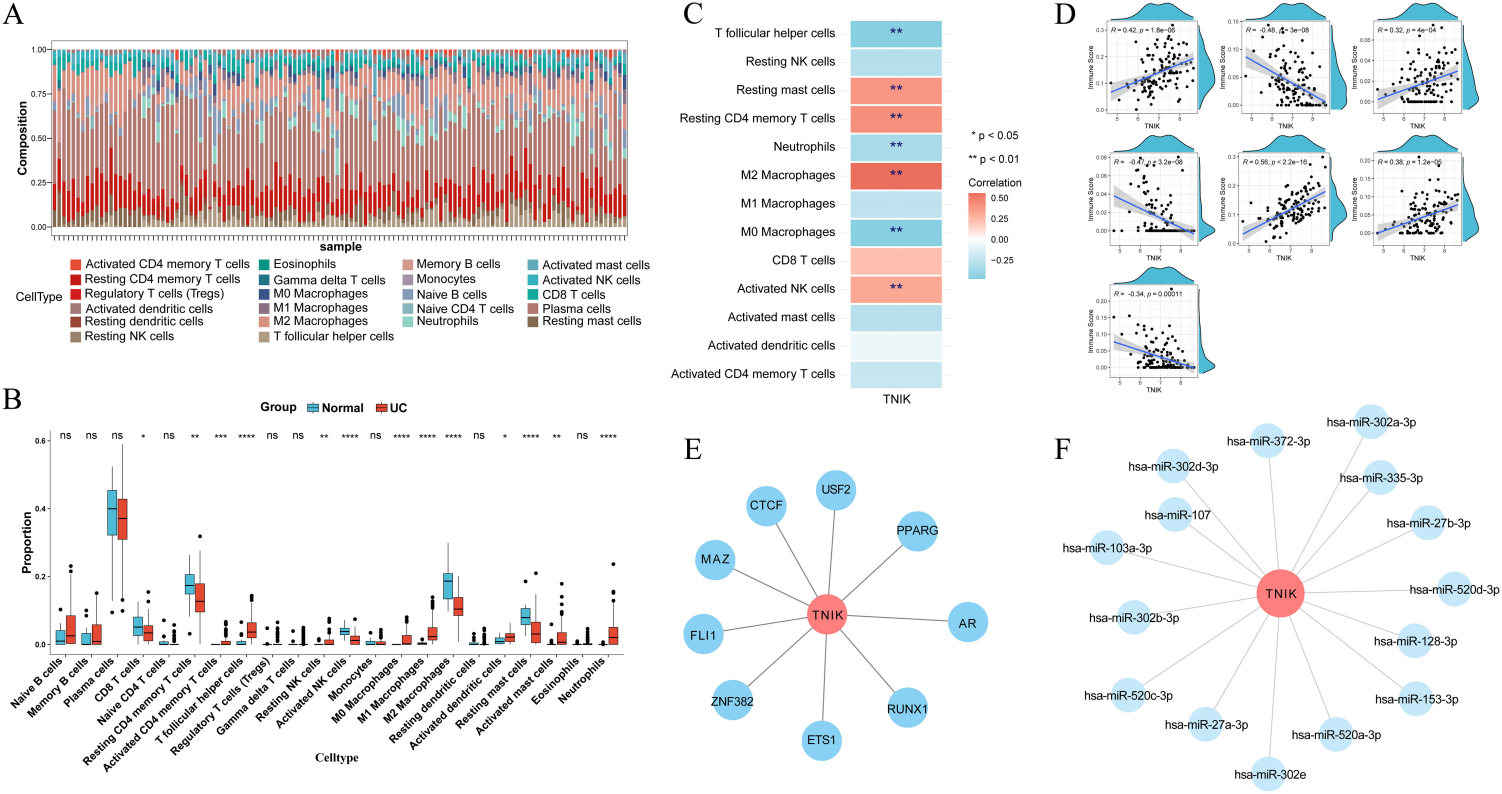
Results of immune cell infiltration analysis and regulatory network analysis. **(A)** CIBERSORT algorithm to get the percentage of immune cell infiltration. **(B)** Wilcoxon rank sum test to get the immune cell differences. **(C)** Heat map of correlation between TNIK and immune cells. **(D)** Scatter plot of correlation between TNIK and immune cells. These analyses linked molecular findings to immune dysregulation in UC. Statistical significance: *P < 0.05, **P < 0.01, ***P < 0.001 and ****P < 0.0001.

Immune infiltration analysis identified notable differences between UC and healthy across 13 immune cell types, including CD8^+^ T cells, activated memory CD4^+^ T cells, T follicular helper cells, and M1 macrophages (**Figures 6A–C**). TNIK was significantly correlated with 7 types of cells: significantly correlated with follicular helper T cells, neutrophils, M0 macrophages negatively, and positively with resting mast cells, resting CD4^+^ T cells, M2 macrophages, and activated NK cells (**Figure 6D**; **Supplementary Table 8**). Transcription factor network analysis revealed that TNIK (TRAF2 and NCK-interacting kinase) was positioned at the regulatory core of several key transcription factors, which played important roles in inflammatory responses and immune regulation (**Figure 6E**). Furthermore, miRNA–TNIK interaction predictions indicated that TNIK may also be regulated by a series of microRNAs (**Figure 6F**).

### Screening of key TNIK-related cells in UC

**Figure 7:**
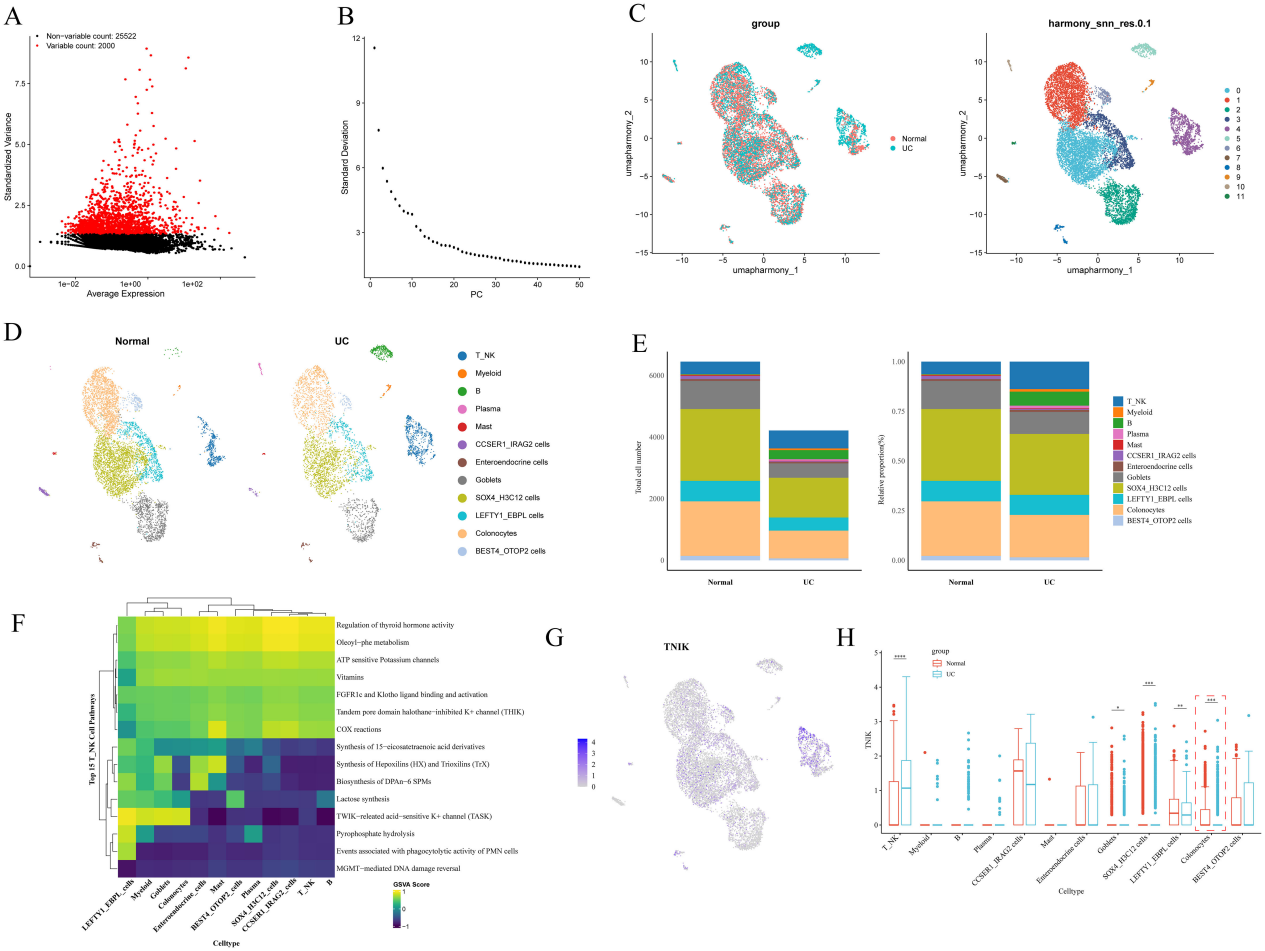
Key cell screening results. **(A)** Graph of screening results for highly variable genes. Highly variable genes are shown in red. **(B)** Principal component ElbowPlot. **(C)** Cell clustering and annotation UMAP plot. **(D)** Plot of cell type distribution in each subgroup. **(E)** Differential results plot of the proportion of cell types. **(F)** Differential cell function enrichment analysis plot. **(G)** The UMAP plot of TNIK expression in differential cells. **(H)** Plot of the results of differential expression of TNIK between groups in each differential cell. This figure highlighted the specific cell subsets contributing to TNIK dysregulation in IBD. Statistical significance: *P < 0.05, **P < 0.01, ***P < 0.001 and ****P < 0.0001.

To delineate the expression characteristics and cellular heterogeneity of TNIK, we next analyzed scRNA-seq data. The raw data of the single-cell dataset GSE116222 were first processed for quality control for subsequent analysis. A total of 10, 661 cells and 27, 522 genes were screened after QC analysis (**Supplementary Figure 5**). The top 2, 000 HVGs with high coefficients of variation were screened (**Figure 7A**). The ElbowPlot showed a plateau at 30 principal components (PCs), which were then selected for downstream dimensionality reduction and clustering analysis (**Figure 7B**). Subsequently, 12 distinct cell clusters were identified (**Figure 7C**). Based on reference marker gene expression, the 12 clusters were annotated into 12 cell types, categorized into six major cell classes: T/NK cells, B cells, myeloid cells, plasma cells, mast cells, and colonic epithelial cells (including BEST4^+^/OTOP2^+^ cells, CCSER1^+^/IRAG2^+^ cells, enteroendocrine cells, SOX4^+^/H3C12^+^ cells, LEFTY1^+^/EBPL^+^ cells, and colonocytes) (**Figure 7D**). The expression of marker genes in various types of cells was also confirmed (**Supplementary Figure 6**). Canonical marker genes exhibited consistent expression patterns for each cell type. A marked difference in cell type distribution between UC and control samples was observed (**Figure 7E**), with notable enrichment of T/NK cells, myeloid cells, B cells, plasma cells, and CCSER1^+^/IRAG2^+^ cells in the UC group (**Supplementary Figure 7**). These cells were enriched in 15 biological pathways, including thyroid hormone activity regulation, oleoylphenylalanine metabolism, ATP-sensitive potassium channels, vitamin pathways, FGFR1c, and Klotho ligand binding and activation (**Figure 7F**). TNIK expression levels were compared across the 12 annotated cell types between UC and control samples (**Figures 7G, H**). Notably, TNIK expression was significantly reduced in colonocytes and goblet cells, but elevated in T/NK cells.

### The key gene TNIK was significantly underexpressed in IBD

**Figure 8:**
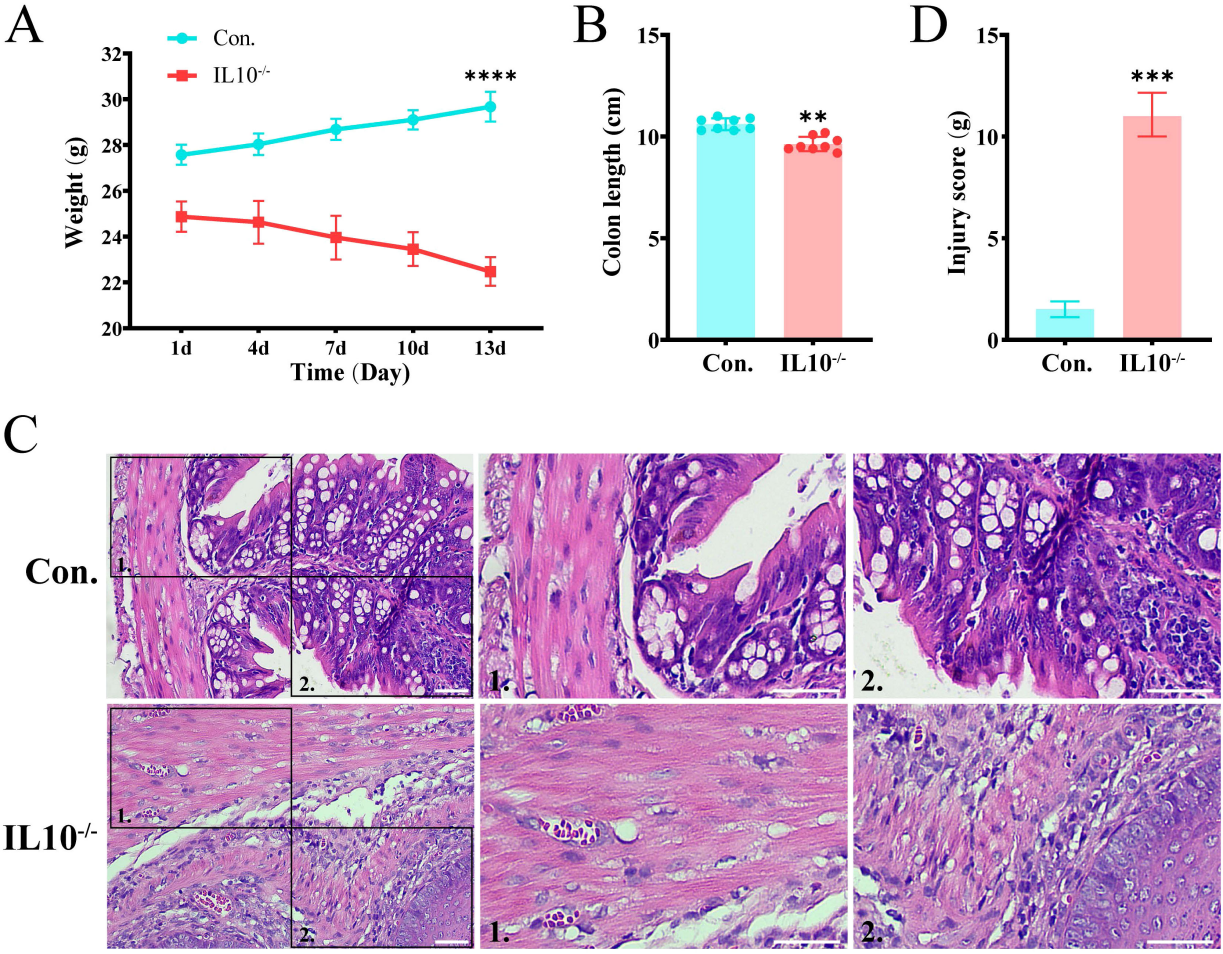
IBD symptoms in IL-10^-/-^ mice. **(A)** Body weight changes of mice. **(B)** Comparison of colon lengths in different groups. **(C)** H&E staining of mouse colon sections. Scale bars, 50 µm. **(D)** The quantitative histological scoring results of H&E staining. Data are presented as mean ± SD. Statistical significance was determined by unpaired t-test. These results confirmed that IL-10^-/-^ mice develop IBD-like symptoms. Statistical significance: **P < 0.01, ***P < 0.001 and ****P < 0.0001.

**Figure 9:**
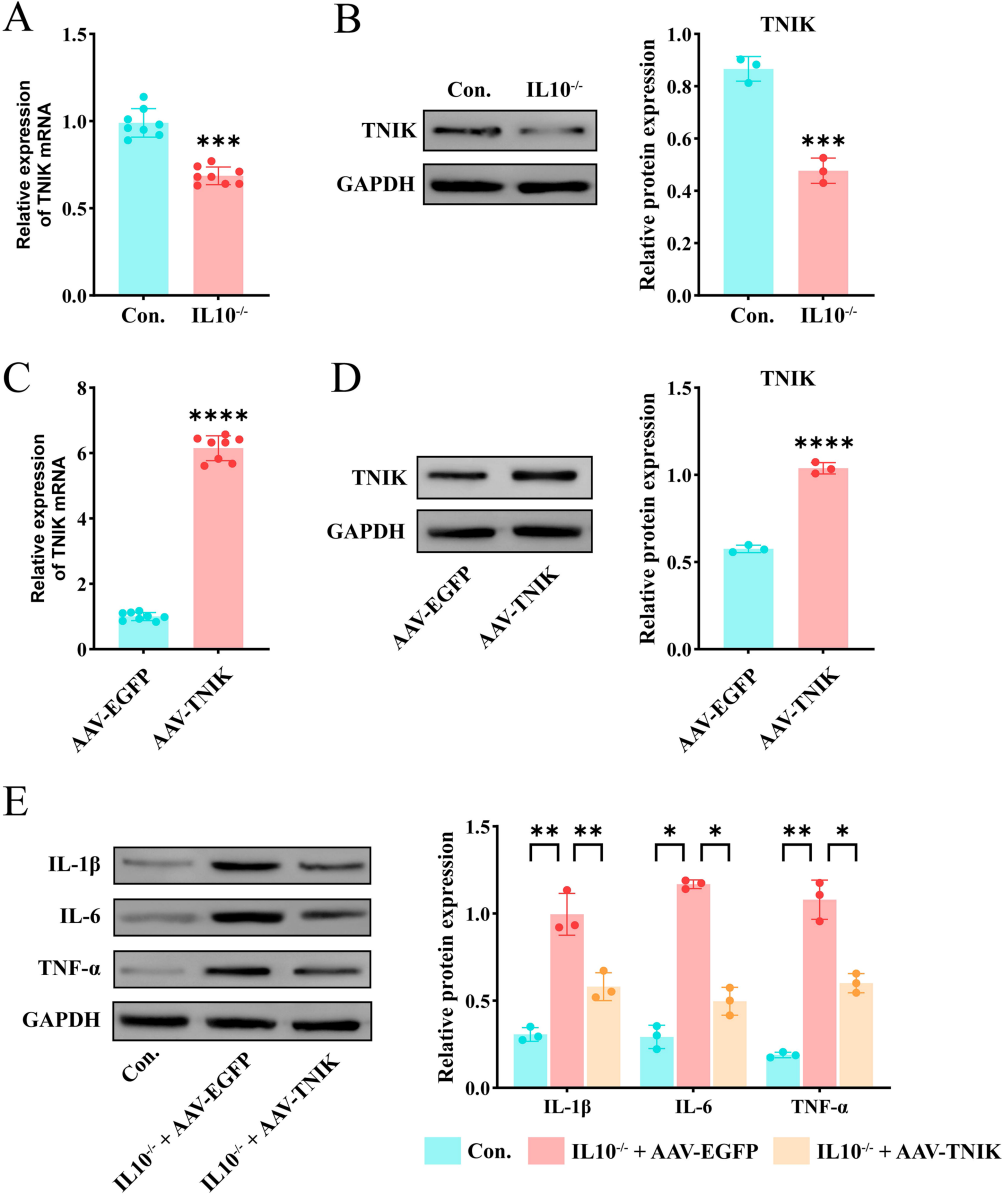
Up-regulation of TNIK alleviates inflammatory response in IL-10^-/-^ mediated IBD. **(A)** Comparison of mRNA expression level of TNIK gene in IBD and Control groups(n=8). **(B)** Comparison of TNIK protein expression levels between IBD and Control groups. **(C)** Results of AAV-TNIK overexpression efficiency (mRNA level) (n=8). **(D)** Results of AAV-TNIK overexpression efficiency (protein level). **(E)** Changes in protein levels of inflammatory factors in IBD after AAV-TNIK overexpression. Data are presented as mean ± SD. Statistical significance in **(A–D)** was determined by unpaired t-test. In **(E)**, two-way ANOVA followed by Tukey’s *post hoc* test was used for multiple comparisons. Collectively, these findings demonstrated that TNIK up-regulation exerts protective effects by attenuating inflammatory responses in IBD. Statistical significance: *P < 0.05, **P < 0.01, ***P < 0.001 and ****P < 0.0001.

In summary, the key gene TNIK was identified and selected as a target for validation and functional investigation. To verify the trend of TNIK expression in IBD, its expression was assessed in both control and IL-10^−^/^−^/^−^/^−^ groups. First, IBD lesion assessment was performed based on weight change, colon length, and H&E staining. The results (**Figures 8A, B**) showed that mice from the IL-10^-/-^ group displayed a marked reduction in body weight and colon length compared to controls (*P* < 0.01). H&E staining (**Figures 8C, D**) revealed features characteristic of IBD in IL-10^−^/^−^/^−^/^−^ mice, including disorganized intestinal structure, disrupted glands, and loss of crypts. Subsequently, qRT-PCR and Western blot (WB) analyses were performed on intestinal tissues from both groups (**Figures 9A, B**). The results demonstrated a significant decrease in TNIK expression in IL-10^−^/^−^/^−^/^−^ mice compared to controls.

### AAV9-mediated TNIK overexpression alleviated the colonic inflammatory response in IL-10^-/-^ mice

To further investigate the biological function of TNIK’s cellular functions, BALB/cAnCya mice were randomly assigned to two groups: AAV-TNIK and AAV-EGFP. Each group received a tail vein injection of the respective recombinant AAV9 vector. AAV-EGFP served as the negative control. Changes in TNIK expression were detected 2 weeks after injection. qRT-PCR and Western blotting (WB) analyses confirmed efficient AAV9-mediated overexpression, as evidenced by markedly elevated TNIK expression in the AAV-TNIK group compared to AAV-EGFP (*P* < 0.001, **Figures 9C, D**). Afterwards, mice were allocated into Control (untreated BALB/cAnCya mice), IL-10^-/-^ + AAV-EGFP, and IL-10^-/-^ + AAV-TNIK group. The WB analysis (**Figure 9E**) showed that, compared to the Control group, the IBD group exhibited markedly elevated levels of inflammatory cytokines IL-1β, IL-6, and TNF-α (*P* < 0.05). In contrast, these cytokines were significantly downregulated in the IL-10^-/-^ + AAV-TNIK group.

### TNIK promoted colonic epithelial cell proliferation, suppresses apoptosis, and alleviates IL-10^-/-^ -mediated tissue injury

**Figure 10:**
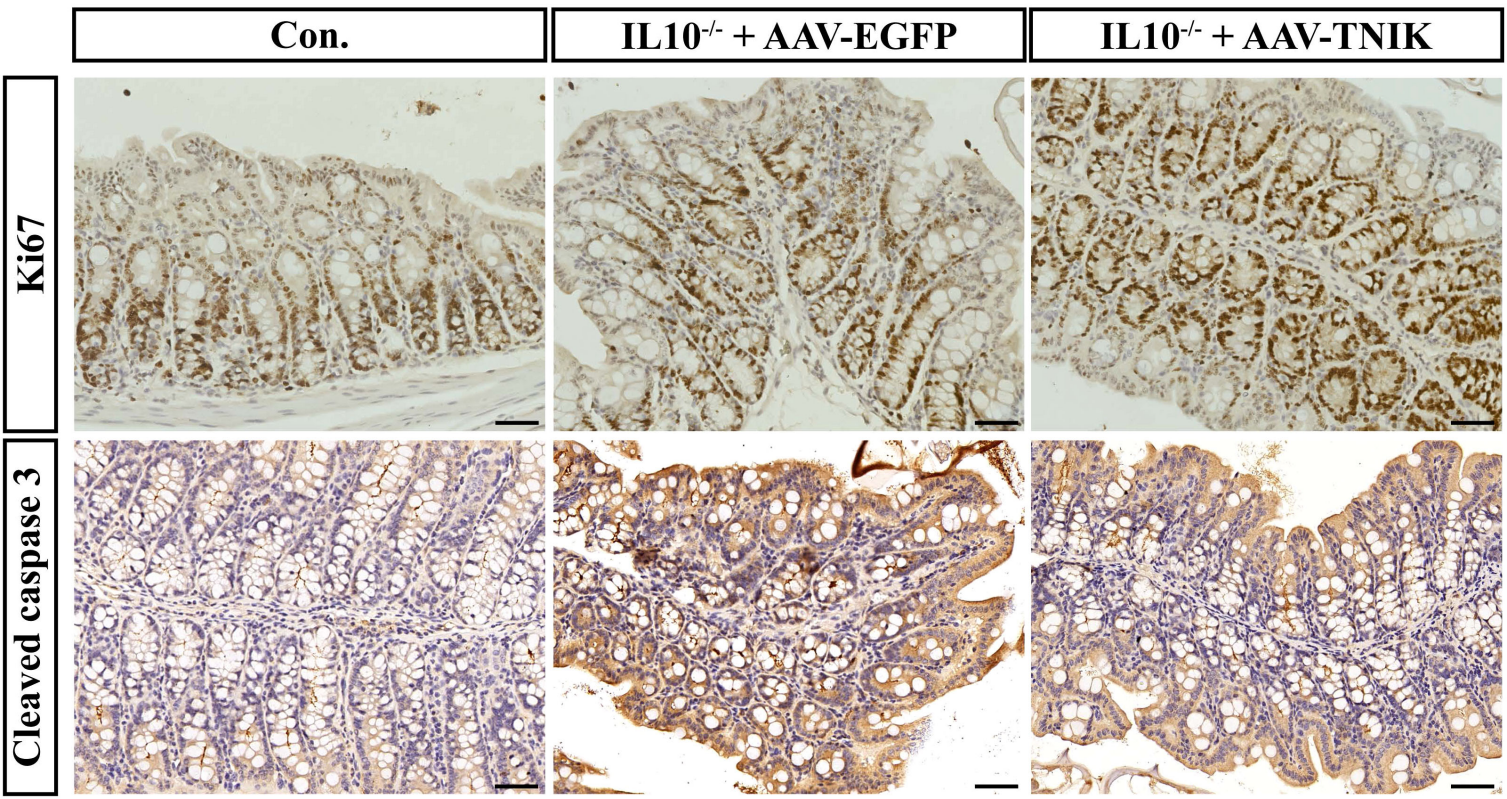
TNIK promotes colonic epithelial cell proliferation and ameliorates apoptosis imbalance. The IHC staining results of mouse colonic tissues showing the expression of Ki67 (top row) and Cleaved caspase-3 (bottom row). Scale bars, 50 µm.

To investigate the role of TNIK in maintaining colonic epithelial homeostasis, immunohistochemical staining for Ki67 and Cleaved caspase3 was performed on mouse colonic tissues (**Figure 10**). In IL-10^-/-^ mice, evident colonic tissue damage was observed, accompanied by increased expression of Cleaved caspase3 and Ki67, indicating enhanced epithelial apoptosis and proliferation. This finding reflects the pathological process of IBD, in which the colon undergoes continuous injury and repair. Notably, AAV-mediated overexpression of TNIK significantly reduced Cleaved caspase 3 expression while further increasing Ki67 levels. Consequently, the extent of colonic tissue injury was markedly alleviated, suggesting that TNIK may restore epithelial homeostasis by promoting epithelial proliferation and suppressing apoptosis, thereby mitigating IBD severity.

## Discussion

Although previous MR studies have established a causal relationship between the GM and IBD, the molecular mechanisms by which GM influences host intestinal pathogenesis remain unclear. In this study, we identified TNIK as a key mediator linking gut microbiota dysbiosis to IBD pathogenesis, with cell type–specific expression patterns and functional validation *in vivo*. It is noteworthy that our study is not the first to recognize the importance of TNIK in IBD. A previous MR analysis identified TNIK as a GM-associated gene significantly related to IBD, predicting that higher TNIK expression exerts a protective effect in patients with IBD (OR = 0.935, 95% CI = 0.915–0.957, *P* < 0.0001), but this observation was not further explored (40). Building on this finding, we provided *in vivo* validation, thereby reinforcing the evidence for the pivotal role of TNIK in IBD.

TNIK encodes a serine/threonine kinase interacting with TRAF2/NCK, known to regulate the Wnt/β-catenin signaling axis and thereby maintain epithelial homeostasis. Previous studies have shown that insufficient Wnt activity in the mucosa of patients with chronic IBD impairs epithelial healing, while exogenous activation of Wnt signaling through R-spondin analogs accelerates epithelial regeneration and ameliorates inflammatory markers of colitis (41). Thus, downregulation of TNIK in colonic epithelial cells may reduce barrier repair capacity, facilitating the translocation of bacteria and their products into the lamina propria and triggering excessive inflammation (42). Although further experimental evidence is required to confirm the role of TNIK in epithelial barrier restoration, our findings demonstrate that TNIK overexpression significantly attenuates colonic inflammation in IL-10^-/-^ mice.

In addition, TNIK also participates in the regulation of inflammatory signaling pathways, further underscoring its multifaceted role in IBD pathogenesis. It has been shown that TNIK deficiency results in an excessive bias of T lymphocytes toward the effector cell lineage, a significant upregulation of inflammatory signaling pathways in damaged epithelial cells, and an increase in apoptosis (43, 44). This suggests that TNIK has a role in the intestinal mucosa in suppressing abnormal inflammatory responses and maintaining immune and barrier homeostasis. Moreover, our single-cell transcriptomic analysis revealed cell type–specific differences in TNIK expression. In colonic epithelial cells (including colonocytes and goblet cells), TNIK expression was markedly downregulated, whereas in T/NK cells, it was upregulated. This suggests that TNIK may exert distinct biological functions in different cellular contexts: reduced expression in epithelial cells could compromise mucosal repair and barrier integrity, thereby facilitating bacterial translocation and triggering inflammation, while elevated expression in immune cells may represent a compensatory mechanism aimed at restraining excessive immune activation (44). Such bidirectional regulation underscores the complexity of TNIK’s role in IBD pathogenesis and highlights the importance of considering cell type–specific contexts when dissecting its precise biological functions.

The strengths of this study are the following. (1) Compared with previous studies, we focused more specifically on identifying key nodal genes mediating the effects of GM on IBD, and further validated their importance using an *in vivo* animal model. (2) We constructed a nomogram model based on the key nodes TMEM163 and TNIK, and demonstrated its favorable predictive performance in the training cohort GSE87473. These findings suggest that our results also possess potential clinical predictive value.

However, several limitations of this study should be acknowledged. (1) We employed a unidirectional MR analysis, which has certain inherent limitations. Nevertheless, multiple sensitivity tests were performed, which to some extent strengthened the robustness of the causal inference between GM and IBD. (2) We did not directly demonstrate whether GM influences IBD by modulating TNIK expression, an important question that warrants broader investigation. (3) Our validation was restricted to the IL-10^-/-^ mouse model, which limits the generalizability of the findings. Future studies should incorporate additional models as well as *in vitro* cellular experiments for further validation. (4) As this work was primarily based on inference and animal experiments, further clinical validation will be of great value to substantiate these findings.

In summary, we combined MR analysis, multi-omics data and animal experimental results, and found that TNIK is a potential target of the intestinal flora to influence the development of IBD, and may be involved in the disease process through the modulation of immune cell function, cytokine secretion and intestinal barrier stability, and other mechanisms together. This finding offers novel insights into the pathogenesis of IBD and may serve as a foundation for future studies exploring its underlying mechanisms. However, more clinical trials and data are still needed to support how to use TNIK to treat or predict IBD.

## Data Availability

The datasets presented in this study can be found in online repositories. The names of the repository/repositories and accession number(s) can be found in the article/**Supplementary Material**.
