# Supplementary material for: Mendelian randomization integrated with multi-omics analysis identifies TNIK as a key gene in gut microbiota-induced IBD development
Source: Front Immunol. 2025 Nov 18;16:1678444. doi: 10.3389/fimmu.2025.1678444 (PMC12669205; doi:10.3389/fimmu.2025.1678444)
Supplement: Supplementary file 1 [file DataSheet1.pdf]

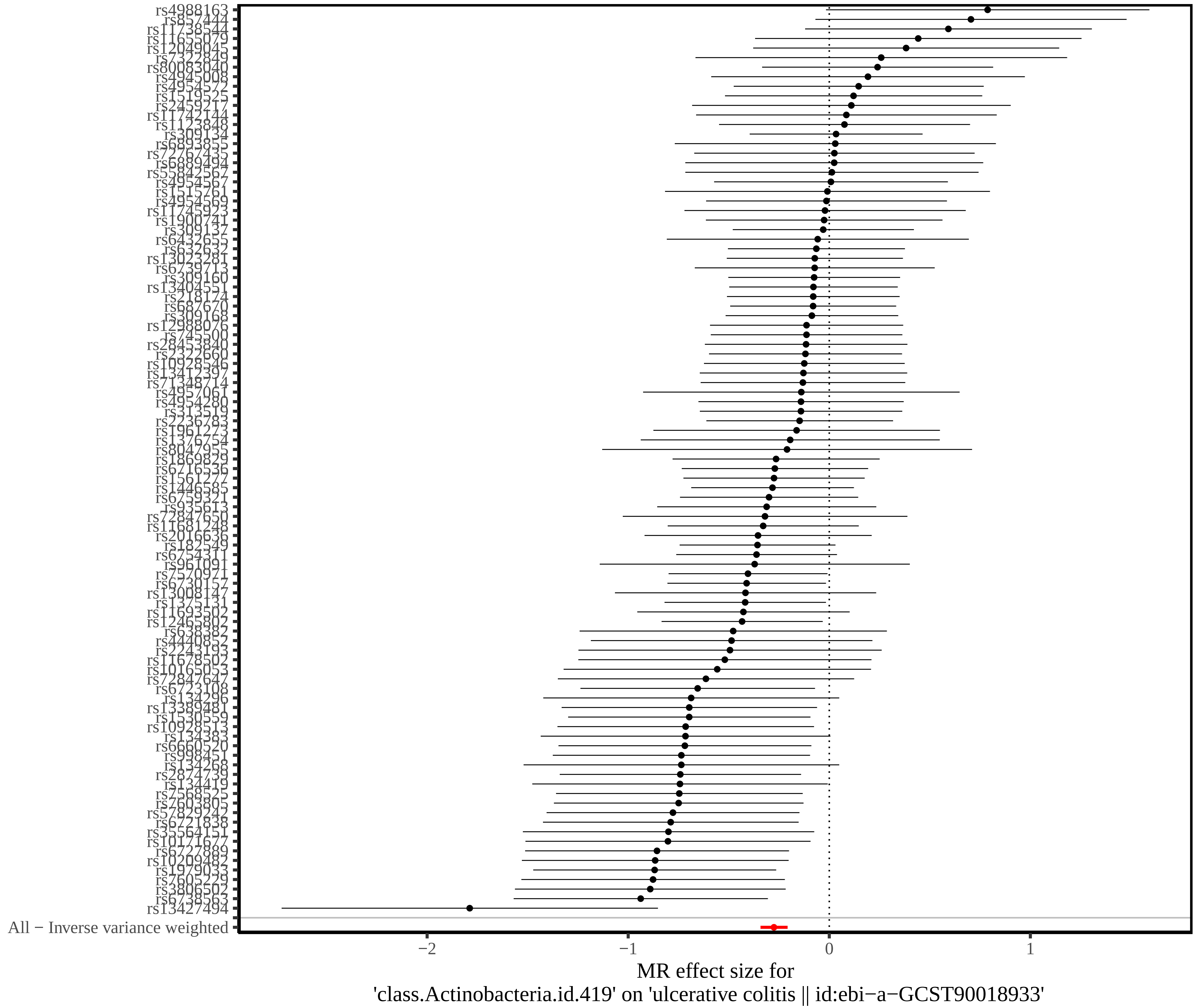
rs4988163
rs13427494
All − Inverse variance weighted
−2
−1
0
1
MR effect size for
'class.Actinobacteria.id.419' on 'ulcerative colitis || id:ebi−a−GCST90018933'

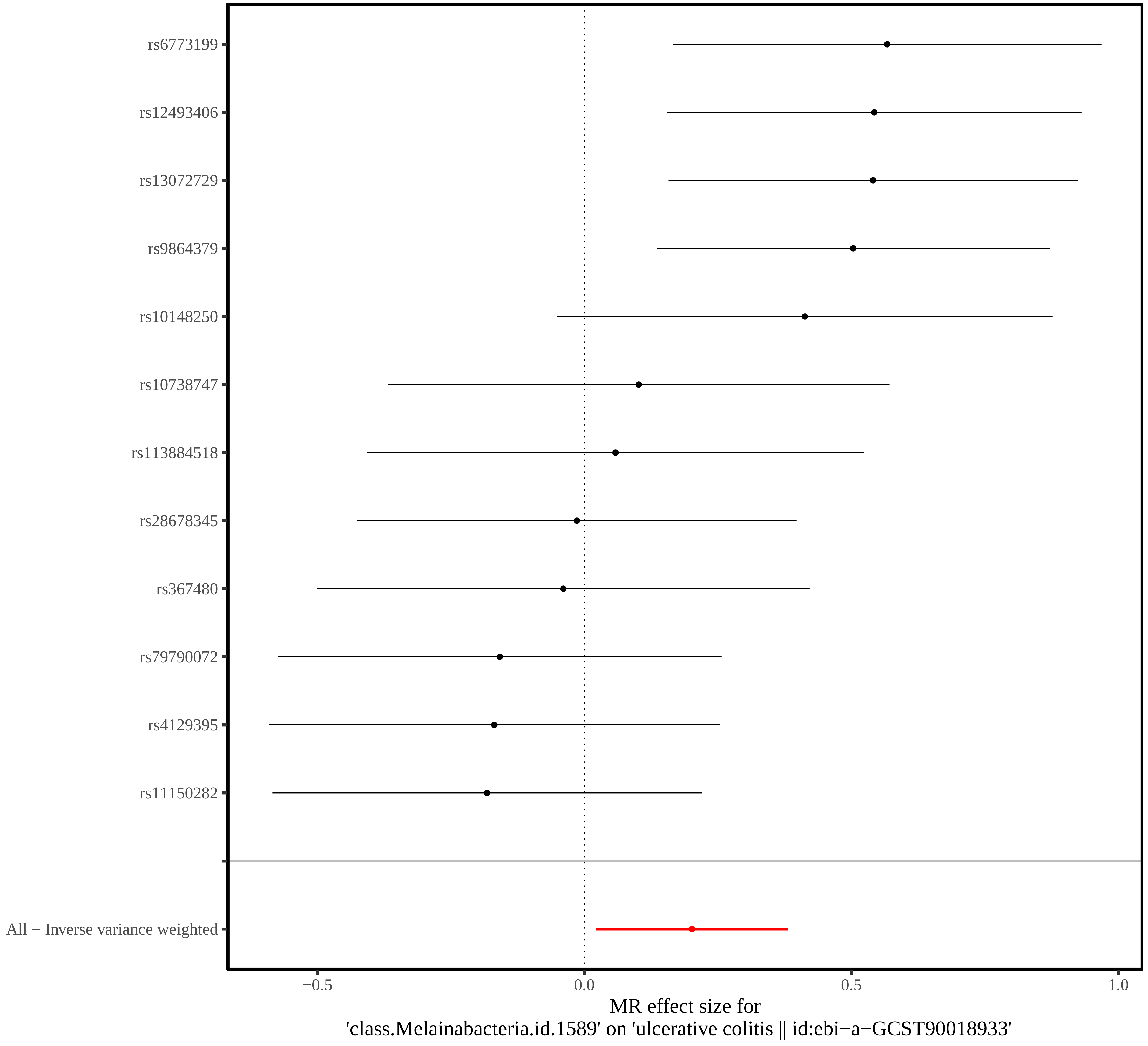

rs6773199
rs12493406
rs13072729
rs9864379
rs10148250
rs10738747
rs113884518
rs28678345
rs367480
rs79790072
rs4129395
rs11150282
All − Inverse variance weighted
−0.5
0.0
0.5
1.0
MR effect size for
'class.Melainabacteria.id.1589' on 'ulcerative colitis || id:ebi−a−GCST90018933'

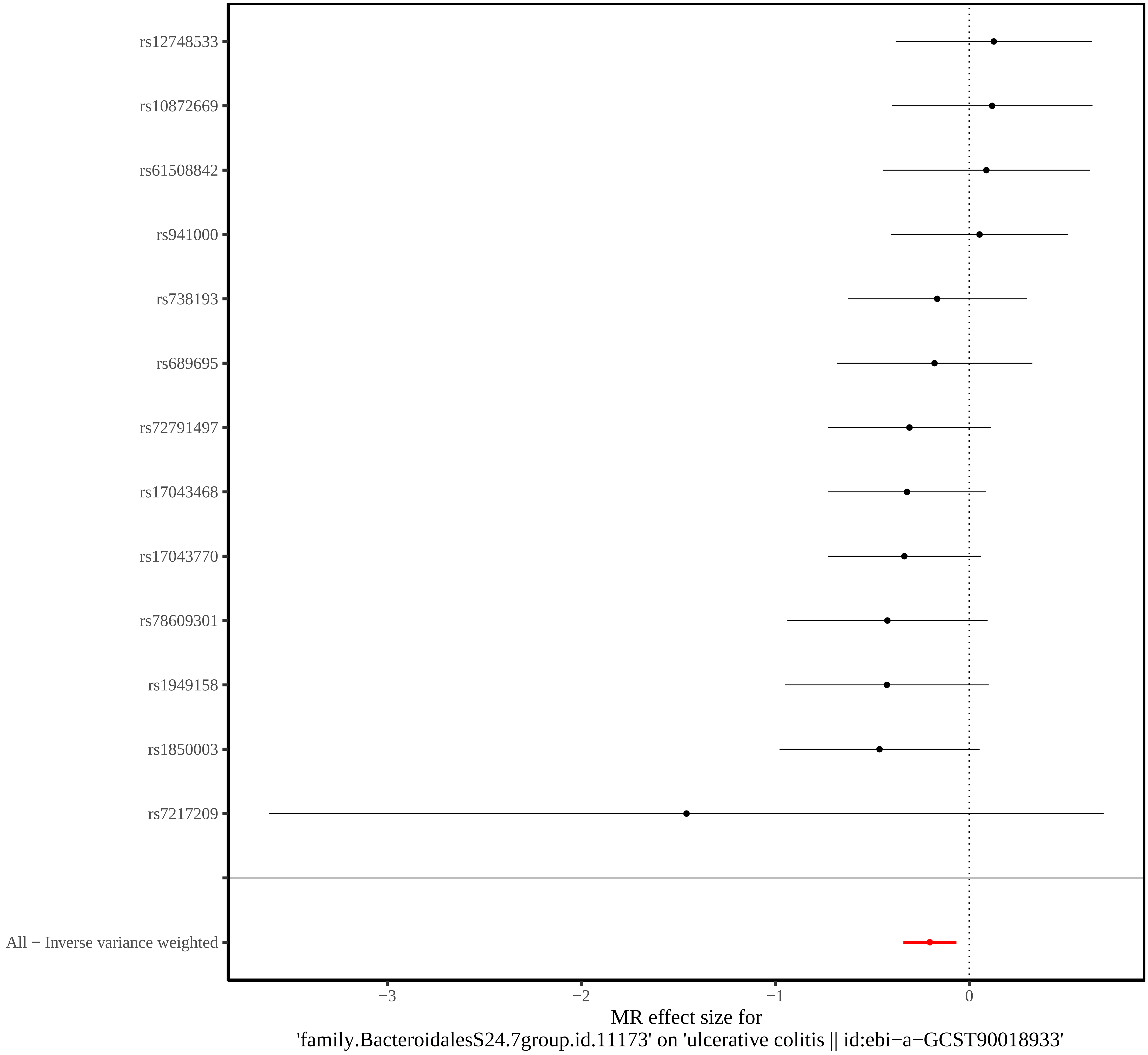

rs12748533
rs10872669
rs61508842
rs941000
rs738193
rs689695
rs72791497
rs17043468
rs17043770
rs78609301
rs1949158
rs1850003
rs7217209
All − Inverse variance weighted
−3
−2
−1
0
MR effect size for
'family.BacteroidalesS24.7group.id.11173' on 'ulcerative colitis || id:ebi−a−GCST90018933'

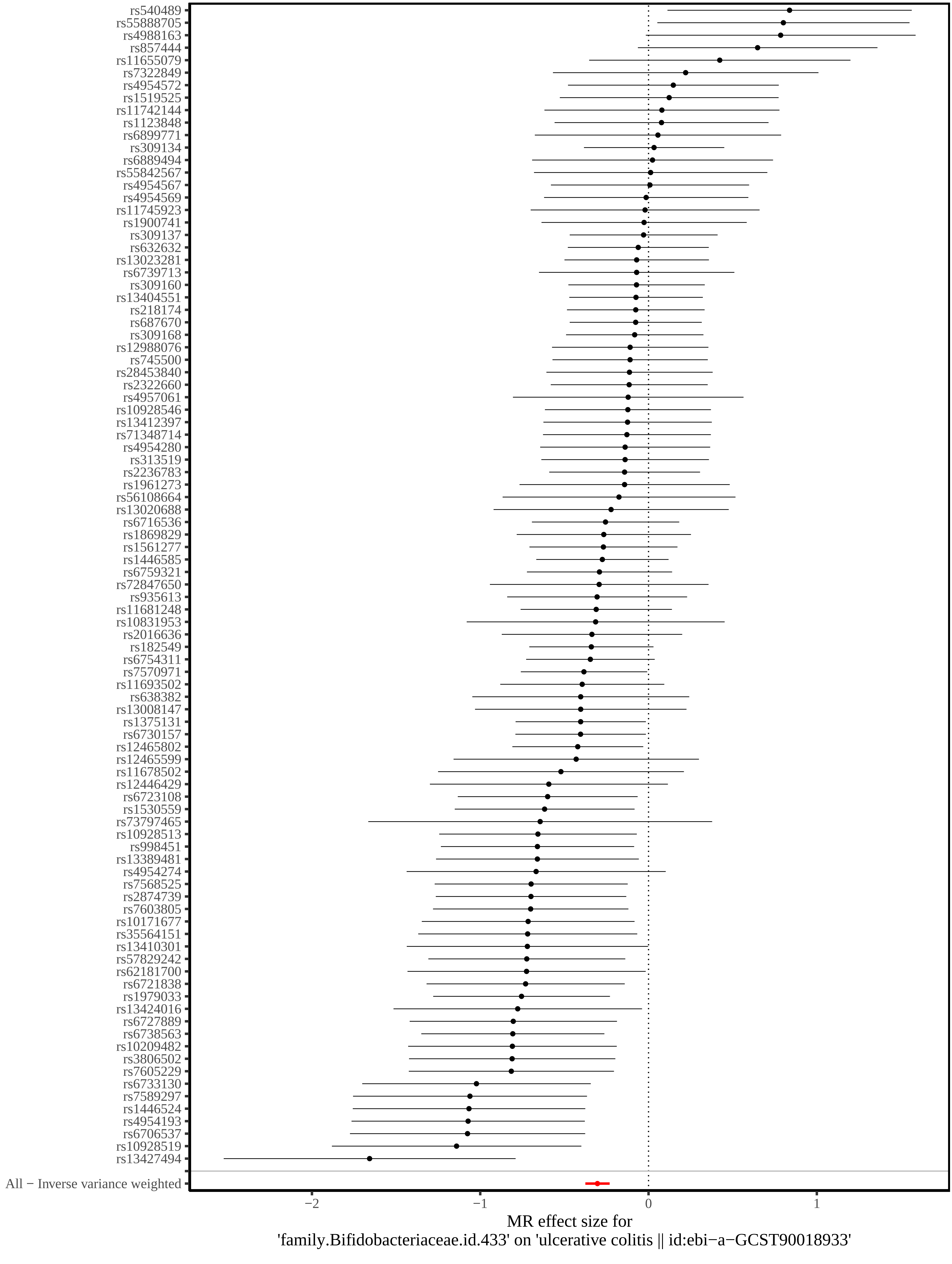

rs540489
rs55888705
rs4988163
rs857444
rs11655079
rs7322849
rs4954572
rs1519525
rs11742144
rs1123848
rs6899771
rs309134
rs6889494
rs55842567
rs4954567
rs4954569
rs11745923
rs1900741
rs309137
rs632632
rs13023281
rs6739713
rs309160
rs13404551
rs218174
rs687670
rs309168
rs12988076
rs745500
rs28453840
rs2322660
rs4957061
rs10928546
rs13412397
rs71348714
rs4954280
rs313519
rs2236783
rs1961273
rs56108664
rs13020688
rs6716536
rs1869829
rs1561277
rs1446585
rs6759321
rs72847650
rs935613
rs11681248
rs10831953
rs2016636
rs182549
rs6754311
rs7570971
rs11693502
rs638382
rs13008147
rs1375131
rs6730157
rs12465802
rs12465599
rs11678502
rs12446429
rs6723108
rs1530559
rs73797465
rs10928513
rs998451
rs13389481
rs4954274
rs7568525
rs2874739
rs7603805
rs10171677
rs35564151
rs13410301
rs57829242
rs62181700
rs6721838
rs1979033
rs13424016
rs6727889
rs6738563
rs10209482
rs3806502
rs7605229
rs6733130
rs7589297
rs1446524
rs4954193
rs6706537
rs10928519
rs13427494
All − Inverse variance weighted
−2
−1
0
1
MR effect size for
'family.Bifidobacteriaceae.id.433' on 'ulcerative colitis || id:ebi−a−GCST90018933'

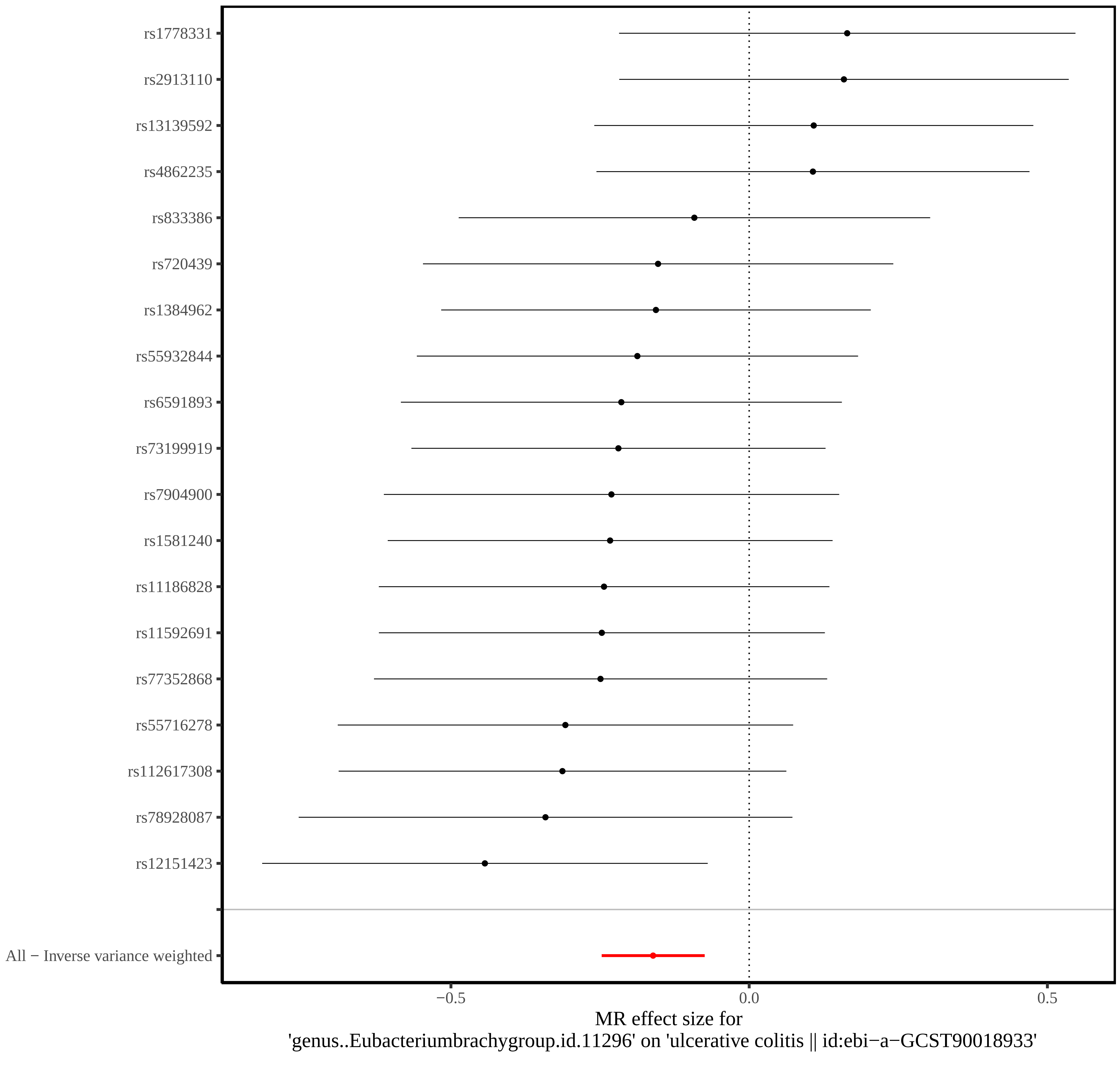

rs1778331
rs2913110
rs13139592
rs4862235
rs833386
rs720439
rs1384962
rs55932844
rs6591893
rs73199919
rs7904900
rs1581240
rs11186828
rs11592691
rs77352868
rs55716278
rs112617308
rs78928087
rs12151423
All − Inverse variance weighted
−0.5
0.0
0.5
MR effect size for
'genus..Eubacteriumbrachygroup.id.11296' on 'ulcerative colitis || id:ebi−a−GCST90018933'

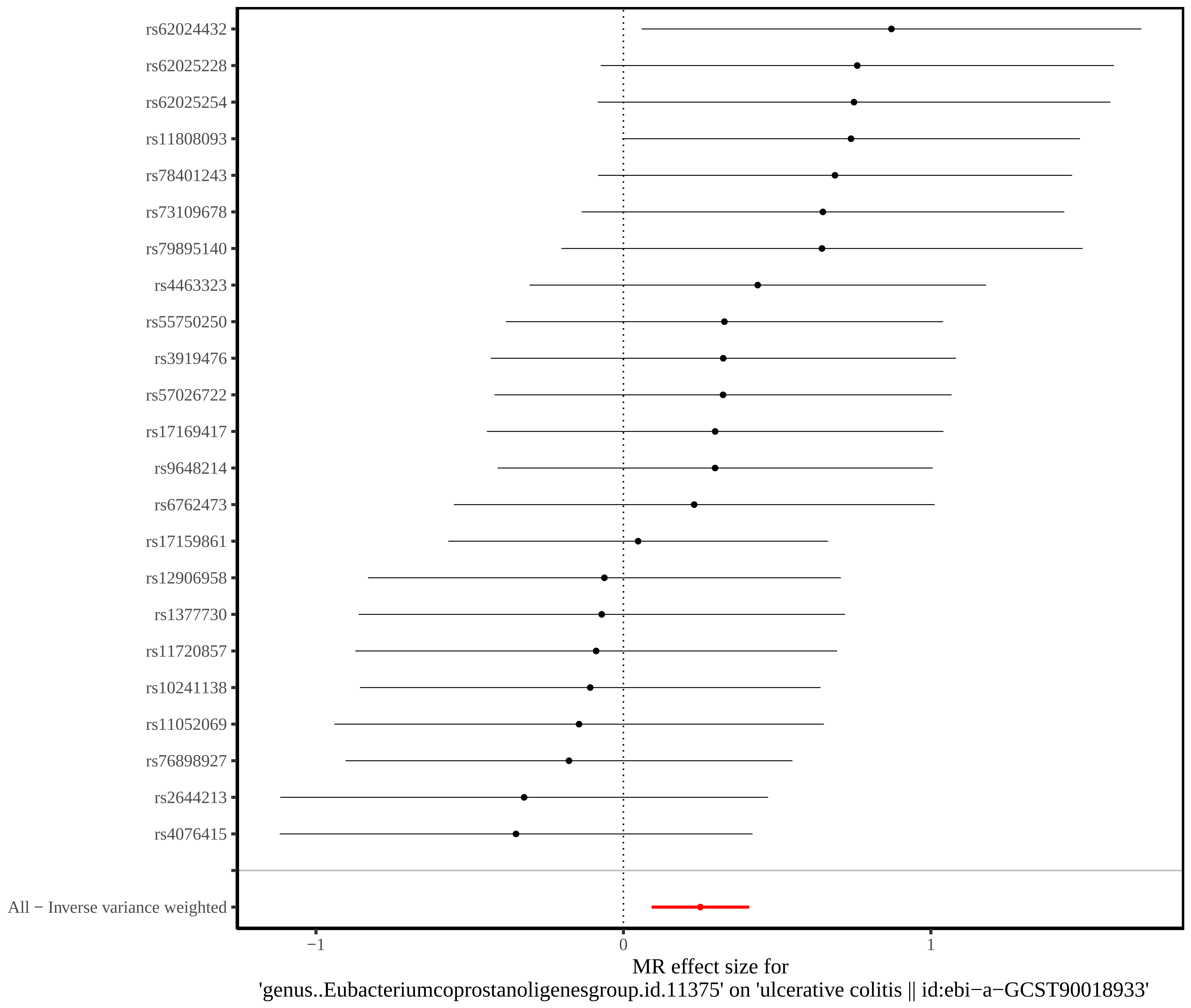
rs62024432
rs62025228
rs62025254
rs11808093
rs78401243
rs73109678
rs79895140
rs4463323
rs55750250
rs3919476
rs57026722
rs17169417
rs9648214
rs6762473
rs17159861
rs12906958
rs1377730
rs11720857
rs10241138
rs11052069
rs76898927
rs2644213
rs4076415
All − Inverse variance weighted
−1
0
1
MR effect size for
'genus..Eubacteriumcoprostanoligenesgroup.id.11375' on 'ulcerative colitis || id:ebi−a−GCST90018933'

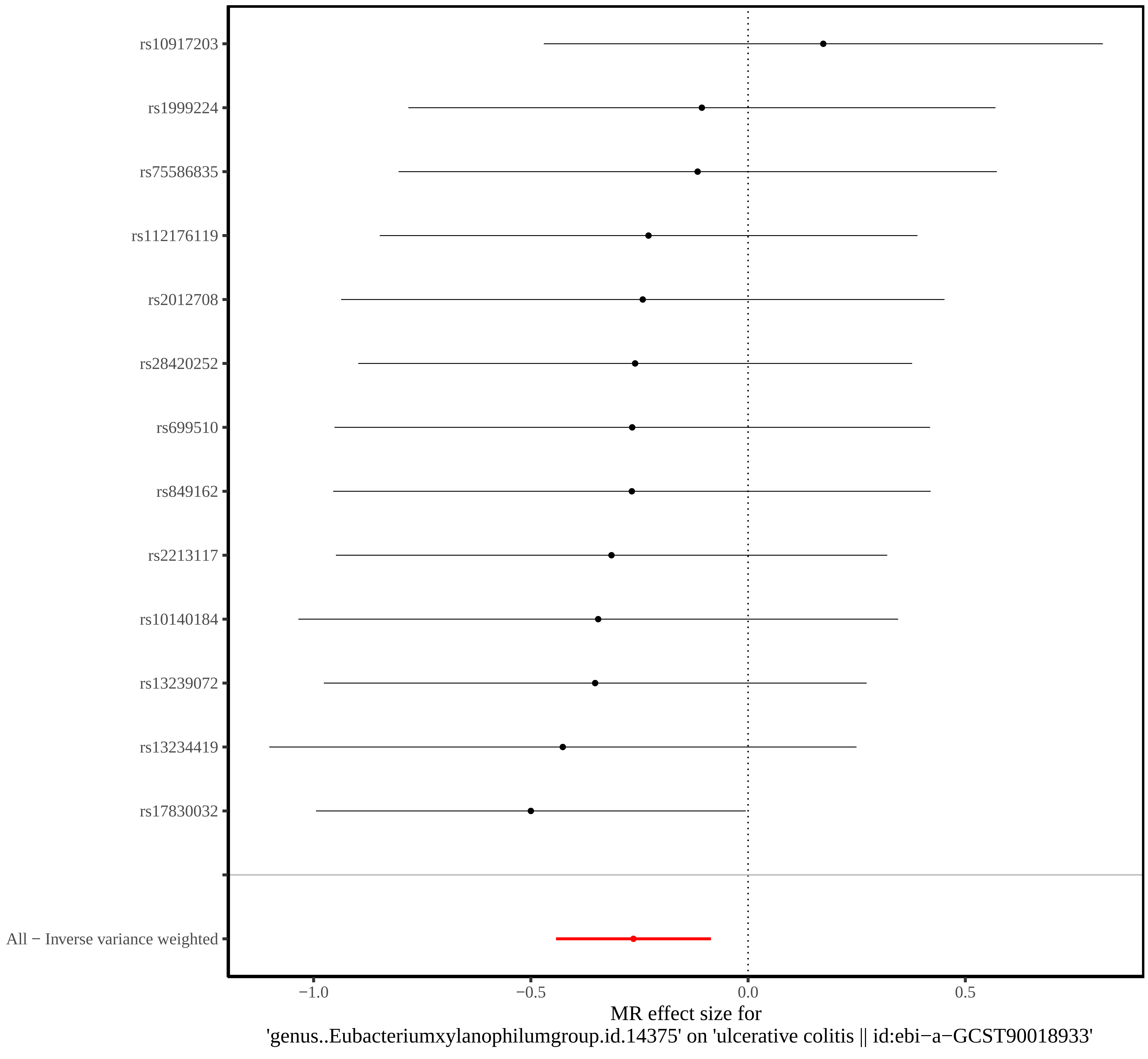
rs10917203
rs1999224
rs75586835
rs112176119
rs2012708
rs28420252
rs699510
rs849162
rs2213117
rs10140184
rs13239072
rs13234419
rs17830032
All − Inverse variance weighted
−1.0
−0.5
0.0
0.5
MR effect size for
'genus..Eubacteriumxylanophilumgroup.id.14375' on 'ulcerative colitis || id:ebi−a−GCST90018933'

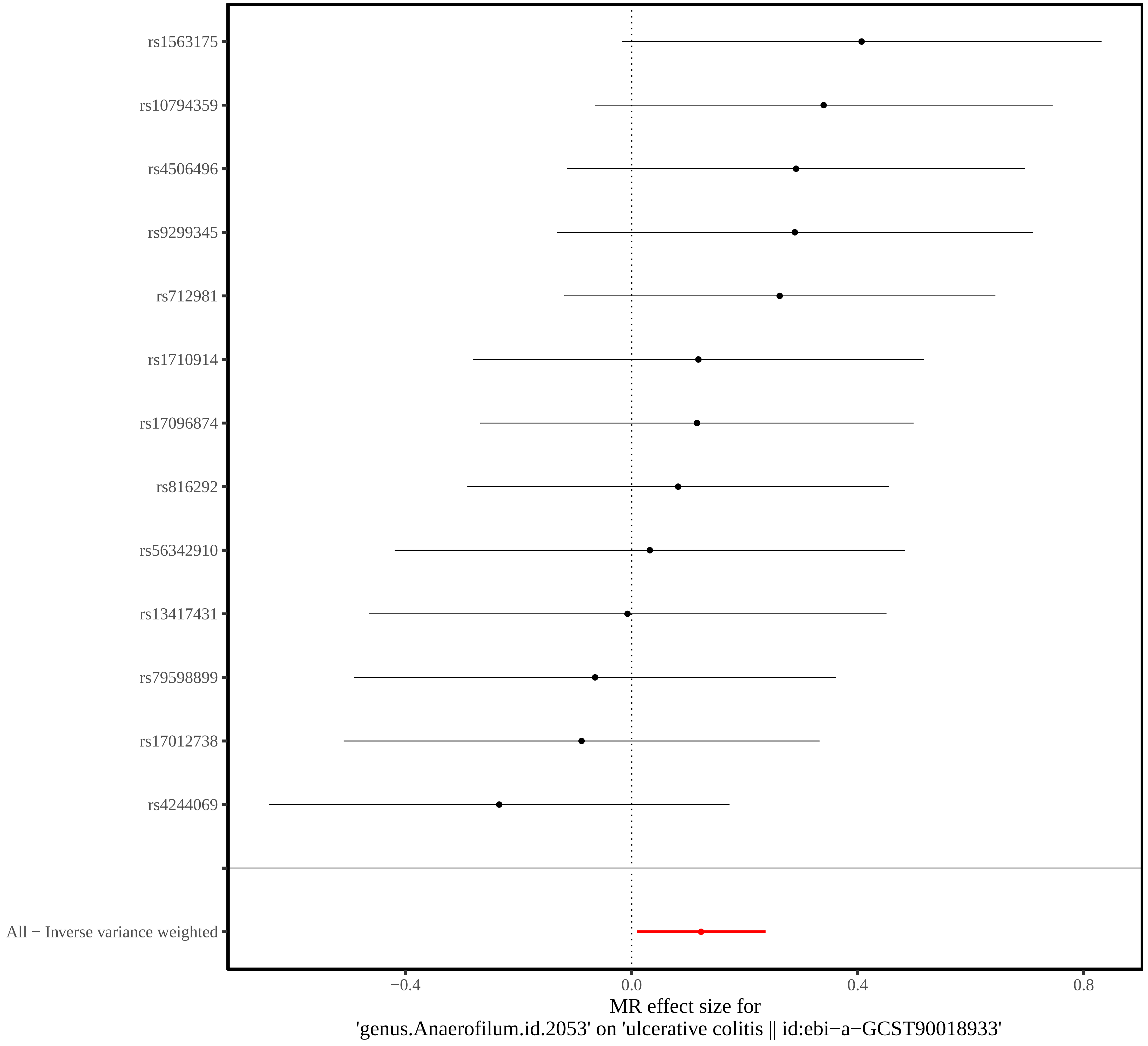
rs1563175
rs10794359
rs4506496
rs9299345
rs712981
rs1710914
rs17096874
rs816292
rs56342910
rs13417431
rs79598899
rs17012738
rs4244069
All − Inverse variance weighted
−0.4
0.0
0.4
0.8
MR effect size for
'genus.Anaerofilum.id.2053' on 'ulcerative colitis || id:ebi−a−GCST90018933'

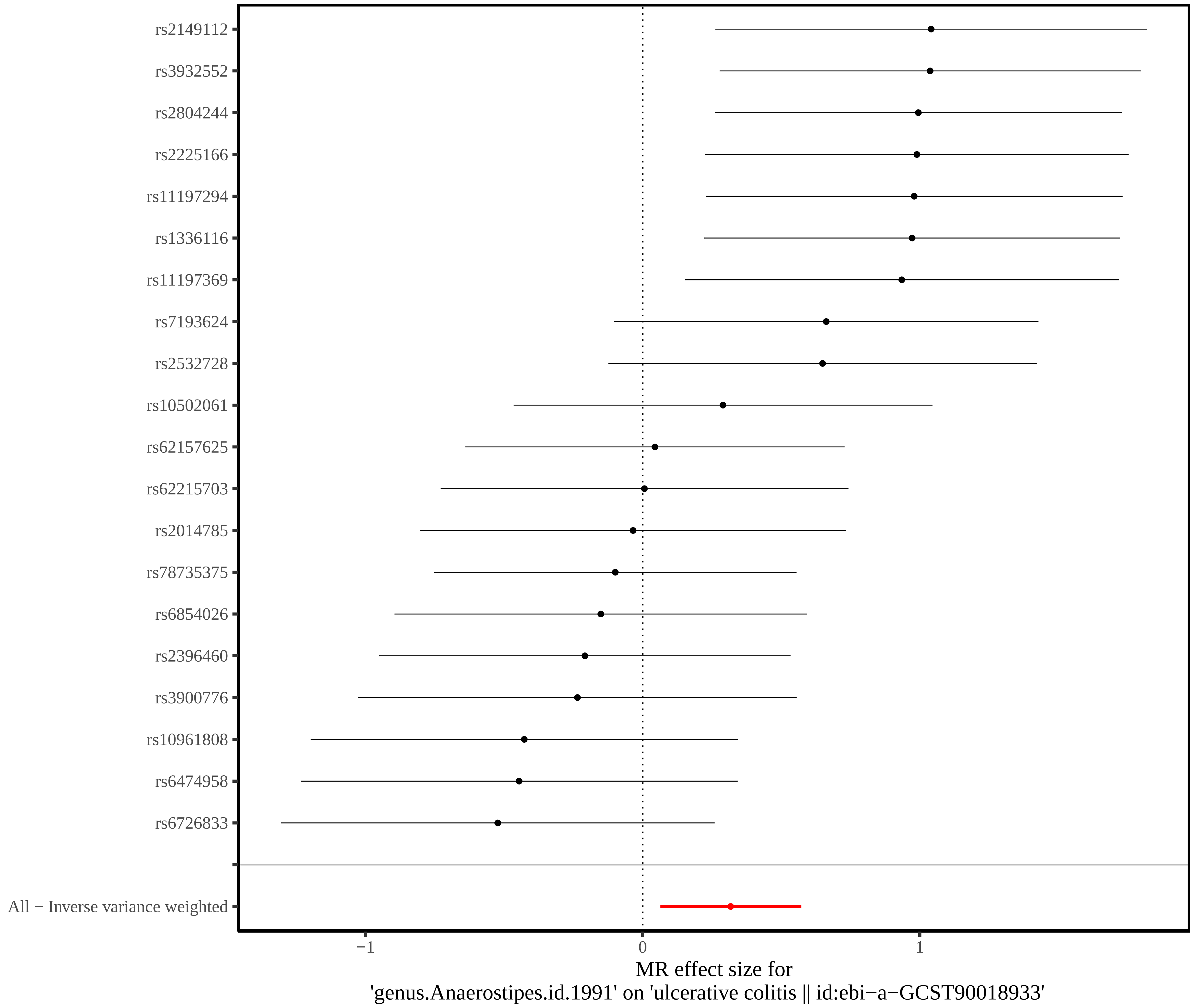
rs2149112
rs3932552
rs2804244
rs2225166
rs11197294
rs1336116
rs11197369
rs7193624
rs2532728
rs10502061
rs62157625
rs62215703
rs2014785
rs78735375
rs6854026
rs2396460
rs3900776
rs10961808
rs6474958
rs6726833
All − Inverse variance weighted
−1
0
1
MR effect size for
'genus.Anaerostipes.id.1991' on 'ulcerative colitis || id:ebi−a−GCST90018933'

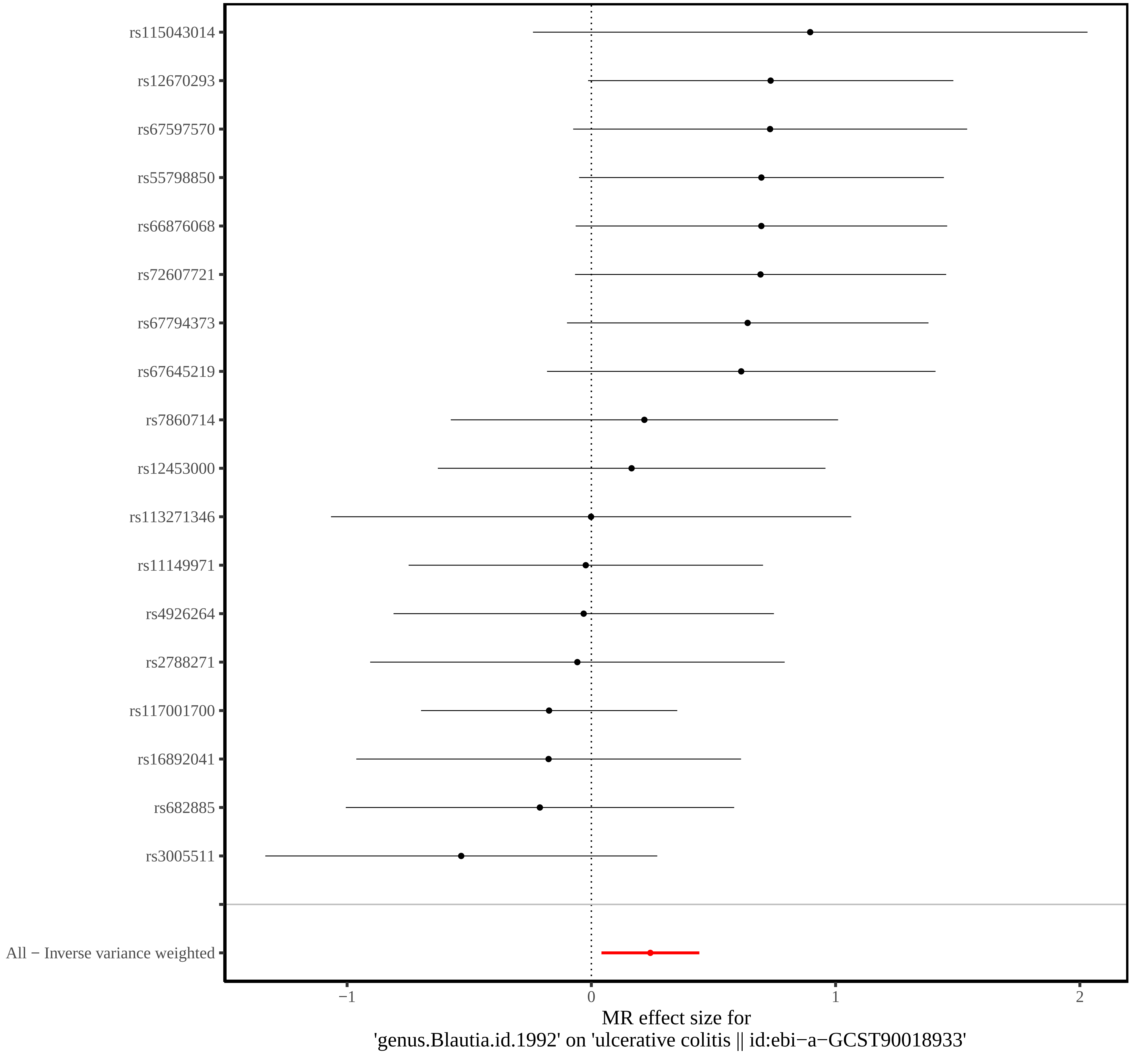
rs115043014
rs12670293
rs67597570
rs55798850
rs66876068
rs72607721
rs67794373
rs67645219
rs7860714
rs12453000
rs113271346
rs11149971
rs4926264
rs2788271
rs117001700
rs16892041
rs682885
rs3005511
All − Inverse variance weighted
−1
0
1
2
MR effect size for
'genus.Blautia.id.1992' on 'ulcerative colitis || id:ebi−a−GCST90018933'

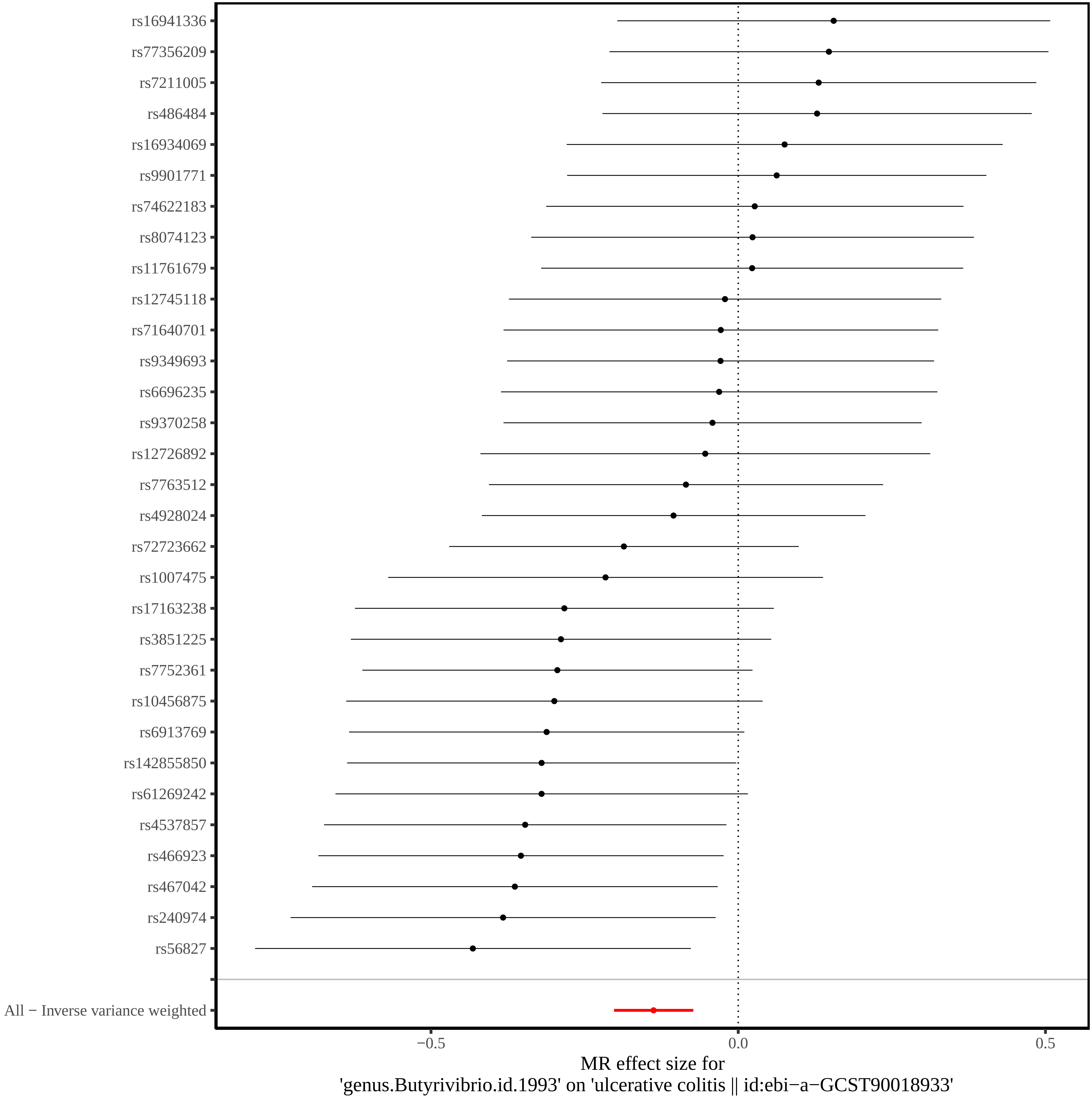
rs16941336
rs77356209
rs7211005
rs486484
rs16934069
rs9901771
rs74622183
rs8074123
rs11761679
rs12745118
rs71640701
rs9349693
rs6696235
rs9370258
rs12726892
rs7763512
rs4928024
rs72723662
rs1007475
rs17163238
rs3851225
rs7752361
rs10456875
rs6913769
rs142855850
rs61269242
rs4537857
rs466923
rs467042
rs240974
rs56827
All − Inverse variance weighted
−0.5
0.0
0.5
MR effect size for
'genus.Butyrivibrio.id.1993' on 'ulcerative colitis || id:ebi−a−GCST90018933'

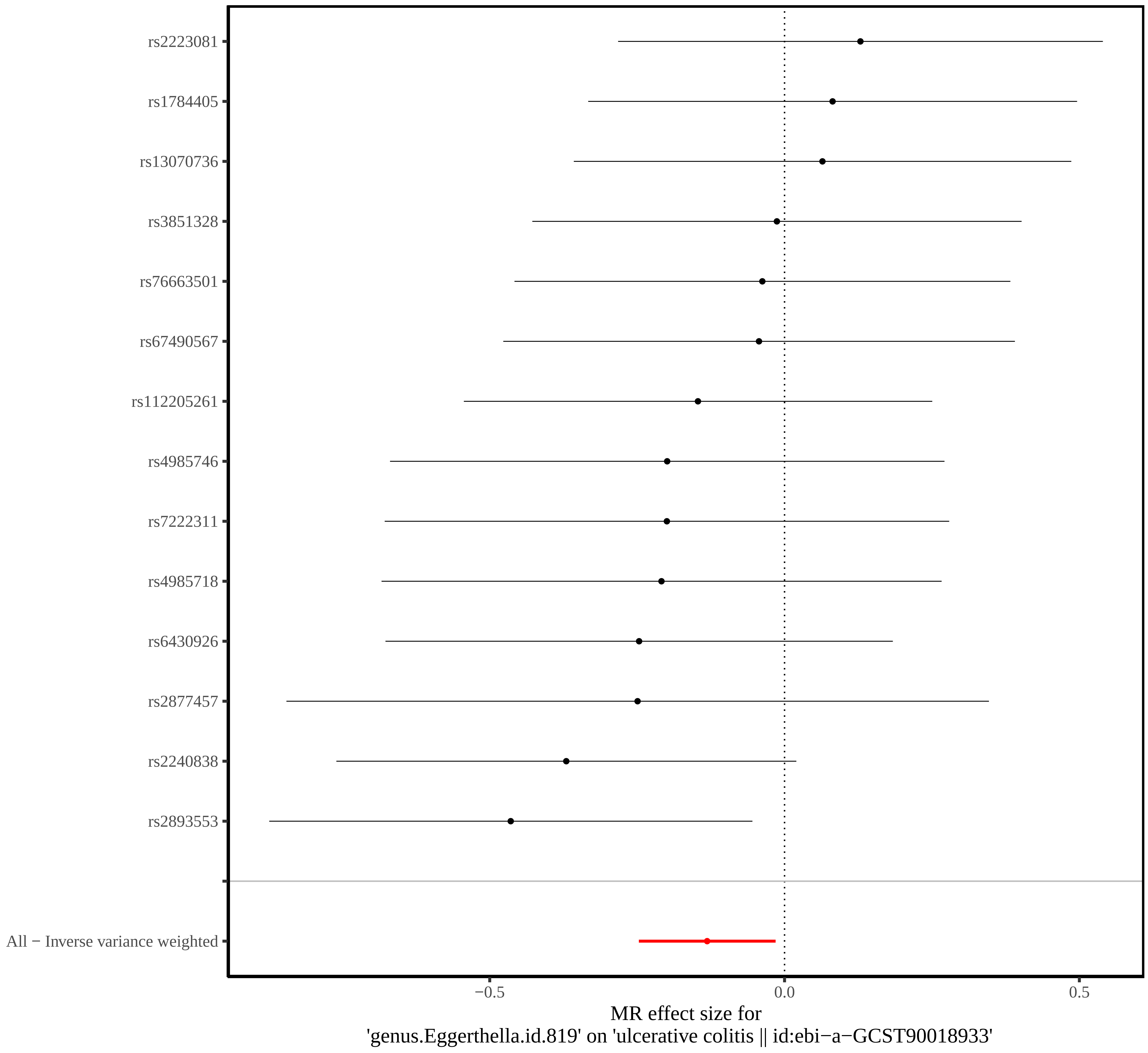
rs2223081
rs1784405
rs13070736
rs3851328
rs76663501
rs67490567
rs112205261
rs4985746
rs7222311
rs4985718
rs6430926
rs2877457
rs2240838
rs2893553
All − Inverse variance weighted
−0.5
0.0
0.5
MR effect size for
'genus.Eggerthella.id.819' on 'ulcerative colitis || id:ebi−a−GCST90018933'

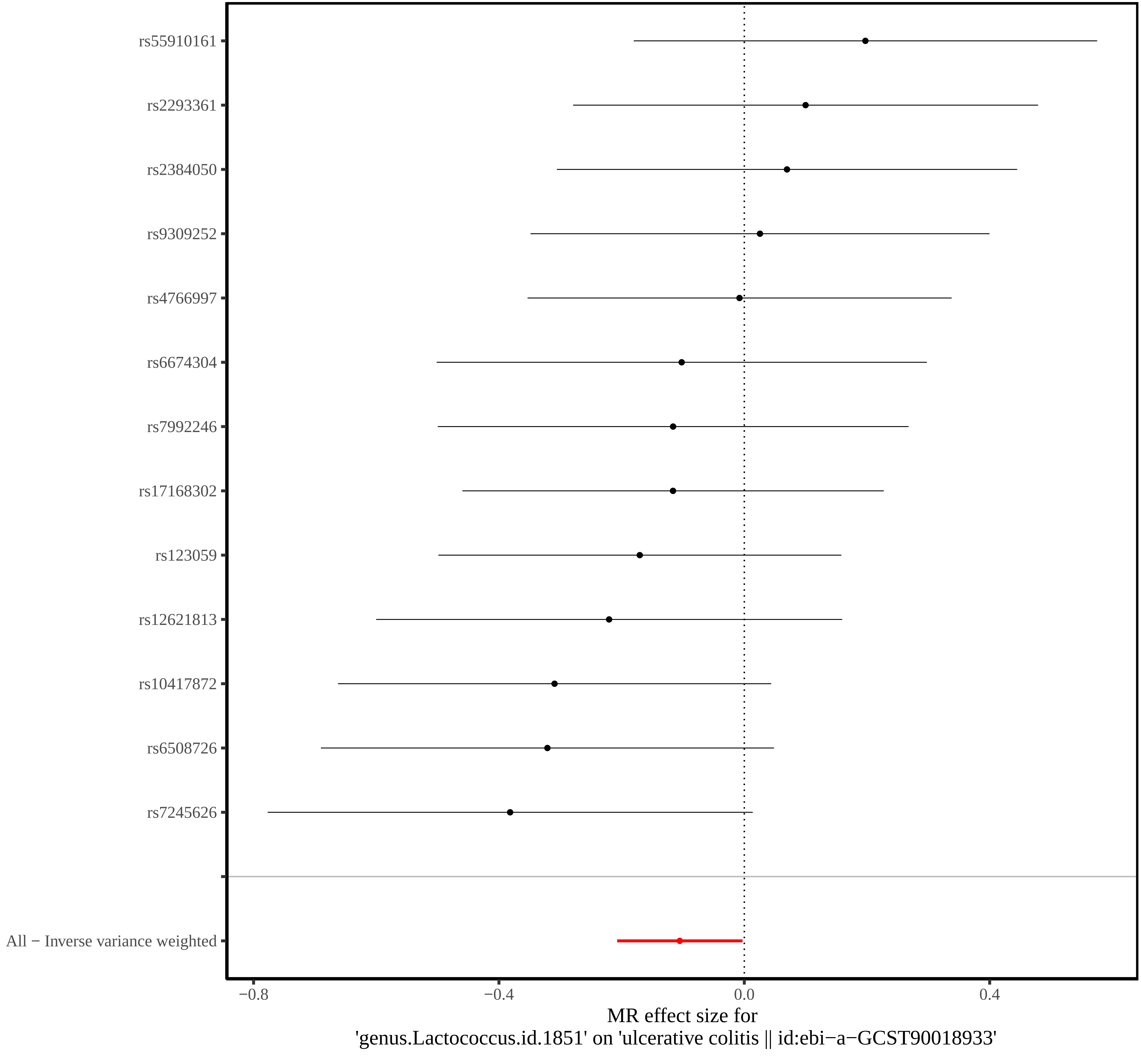
rs55910161
rs2293361
rs2384050
rs9309252
rs4766997
rs6674304
rs7992246
rs17168302
rs123059
rs12621813
rs10417872
rs6508726
rs7245626
All − Inverse variance weighted
−0.8
−0.4
0.0
0.4
MR effect size for
'genus.Lactococcus.id.1851' on 'ulcerative colitis || id:ebi−a−GCST90018933'

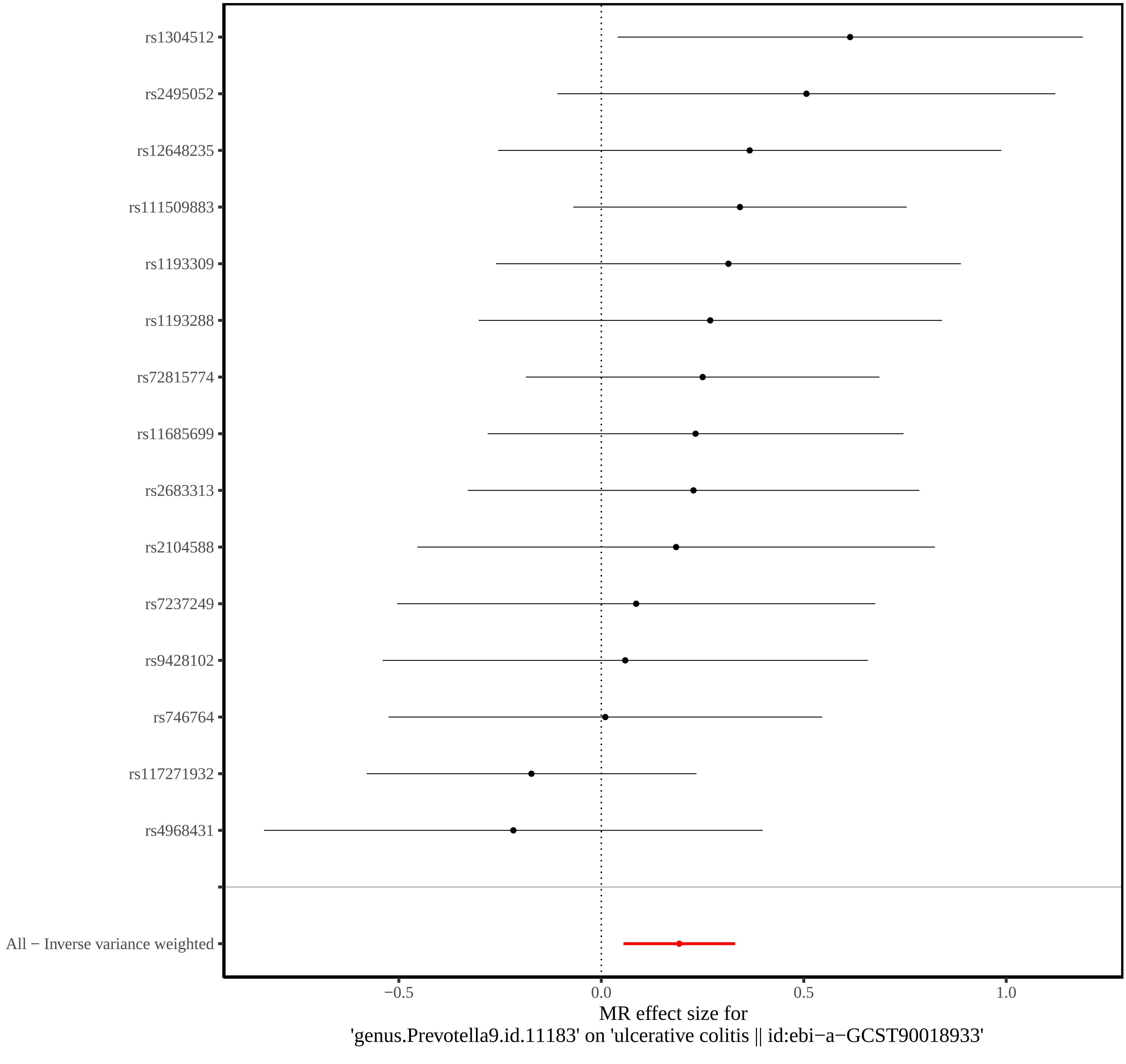

rs1304512
rs2495052
rs12648235
rs111509883
rs1193309
rs1193288
rs72815774
rs11685699
rs2683313
rs2104588
rs7237249
rs9428102
rs746764
rs117271932
rs4968431
All − Inverse variance weighted
−0.5
0.0
0.5
1.0
MR effect size for
'genus.Prevotella9.id.11183' on 'ulcerative colitis || id:ebi−a−GCST90018933'

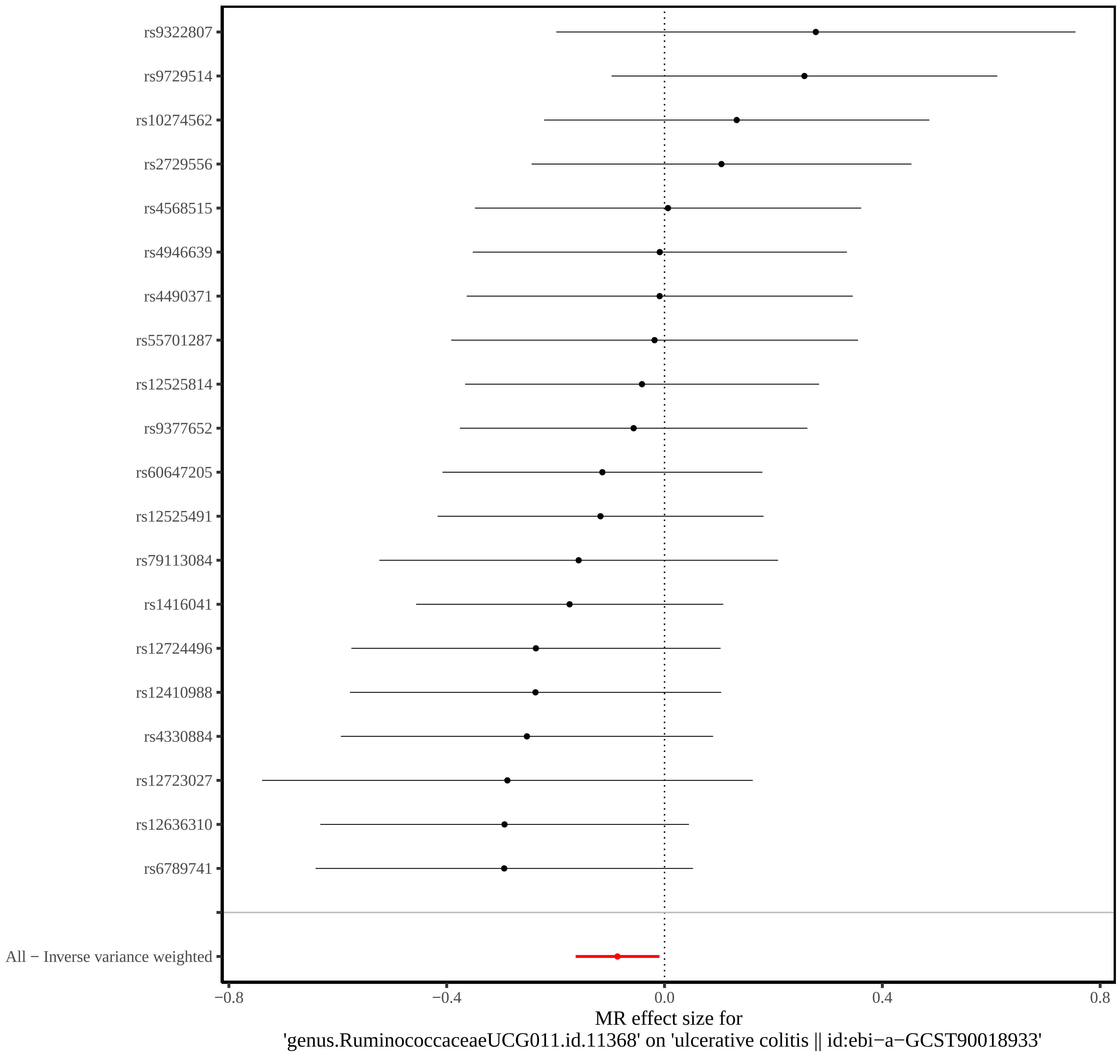
rs9322807
rs9729514
rs10274562
rs2729556
rs4568515
rs4946639
rs4490371
rs55701287
rs12525814
rs9377652
rs60647205
rs12525491
rs79113084
rs1416041
rs12724496
rs12410988
rs4330884
rs12723027
rs12636310
rs6789741
All − Inverse variance weighted
−0.8
−0.4
0.0
0.4
0.8
MR effect size for
'genus.RuminococcaceaeUCG011.id.11368' on 'ulcerative colitis || id:ebi−a−GCST90018933'

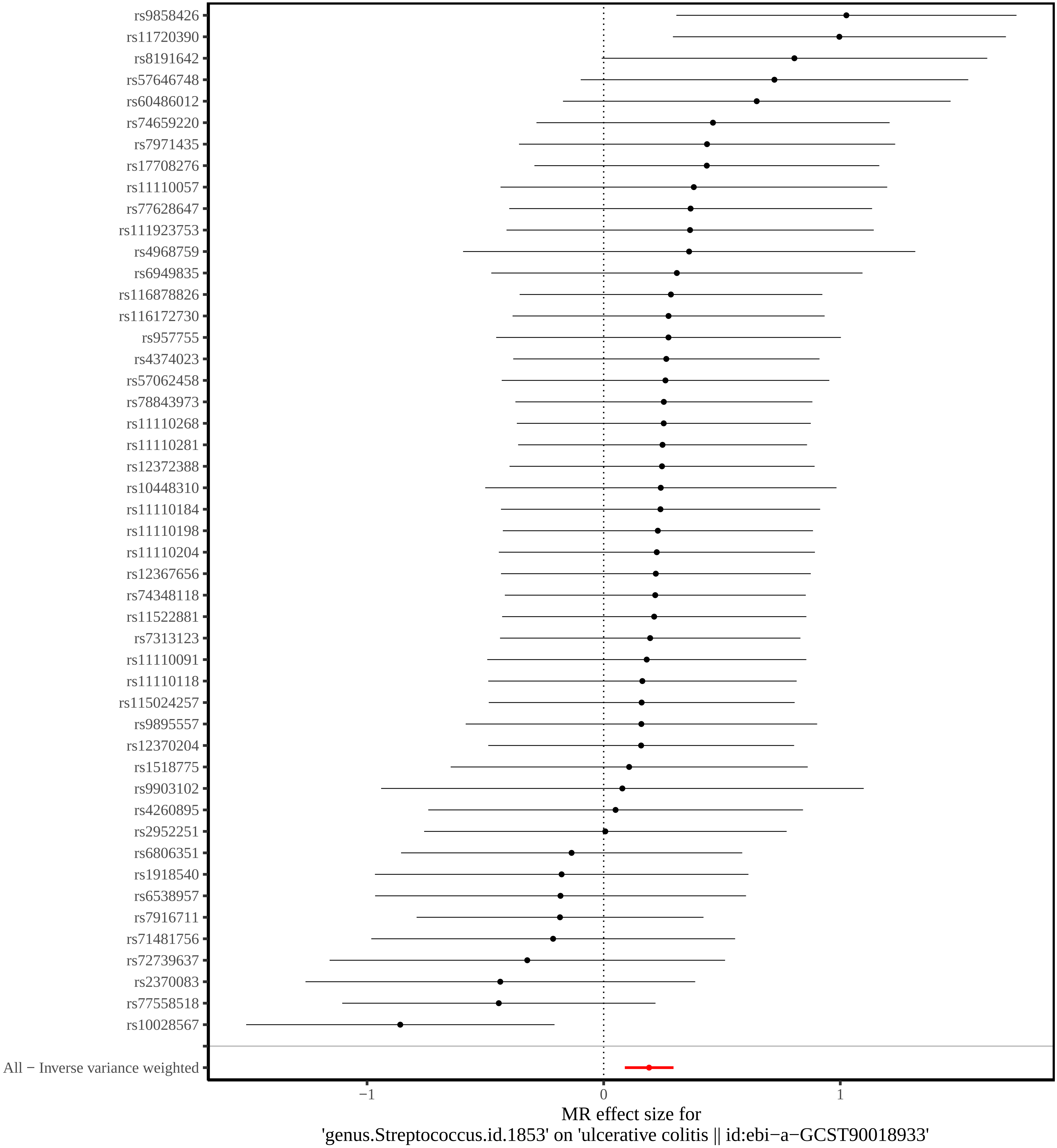

rs9858426
rs11720390
rs8191642
rs57646748
rs60486012
rs74659220
rs7971435
rs17708276
rs11110057
rs77628647
rs111923753
rs4968759
rs6949835
rs116878826
rs116172730
rs957755
rs4374023
rs57062458
rs78843973
rs11110268
rs11110281
rs12372388
rs10448310
rs11110184
rs11110198
rs11110204
rs12367656
rs74348118
rs11522881
rs7313123
rs11110091
rs11110118
rs115024257
rs9895557
rs12370204
rs1518775
rs9903102
rs4260895
rs2952251
rs6806351
rs1918540
rs6538957
rs7916711
rs71481756
rs72739637
rs2370083
rs77558518
rs10028567
All − Inverse variance weighted
−1
0
1
MR effect size for
'genus.Streptococcus.id.1853' on 'ulcerative colitis || id:ebi−a−GCST90018933'

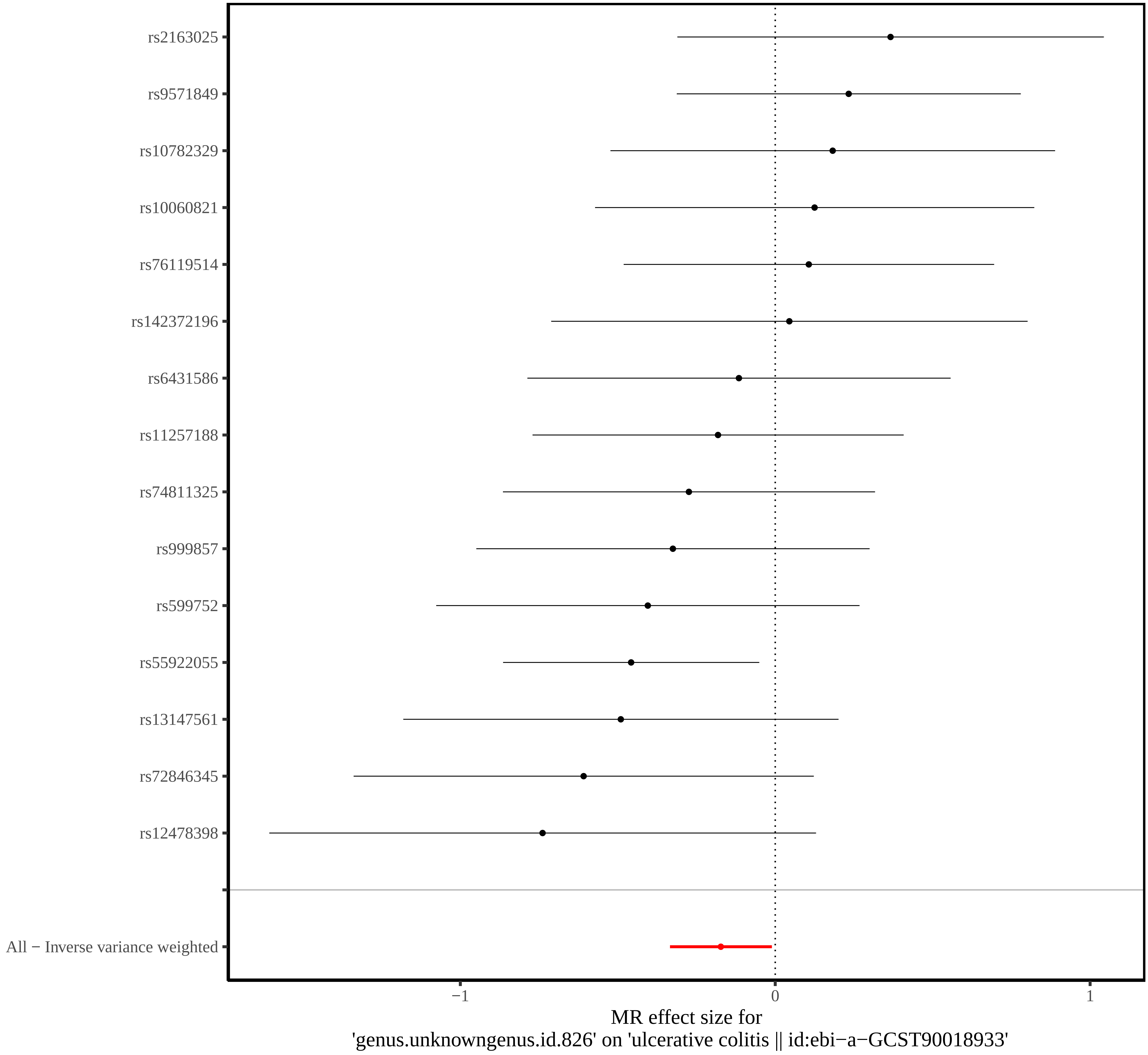

rs2163025
rs9571849
rs10782329
rs10060821
rs76119514
rs142372196
rs6431586
rs11257188
rs74811325
rs999857
rs599752
rs55922055
rs13147561
rs72846345
rs12478398
All − Inverse variance weighted
−1
0
1
MR effect size for
'genus.unknowngenus.id.826' on 'ulcerative colitis || id:ebi−a−GCST90018933'

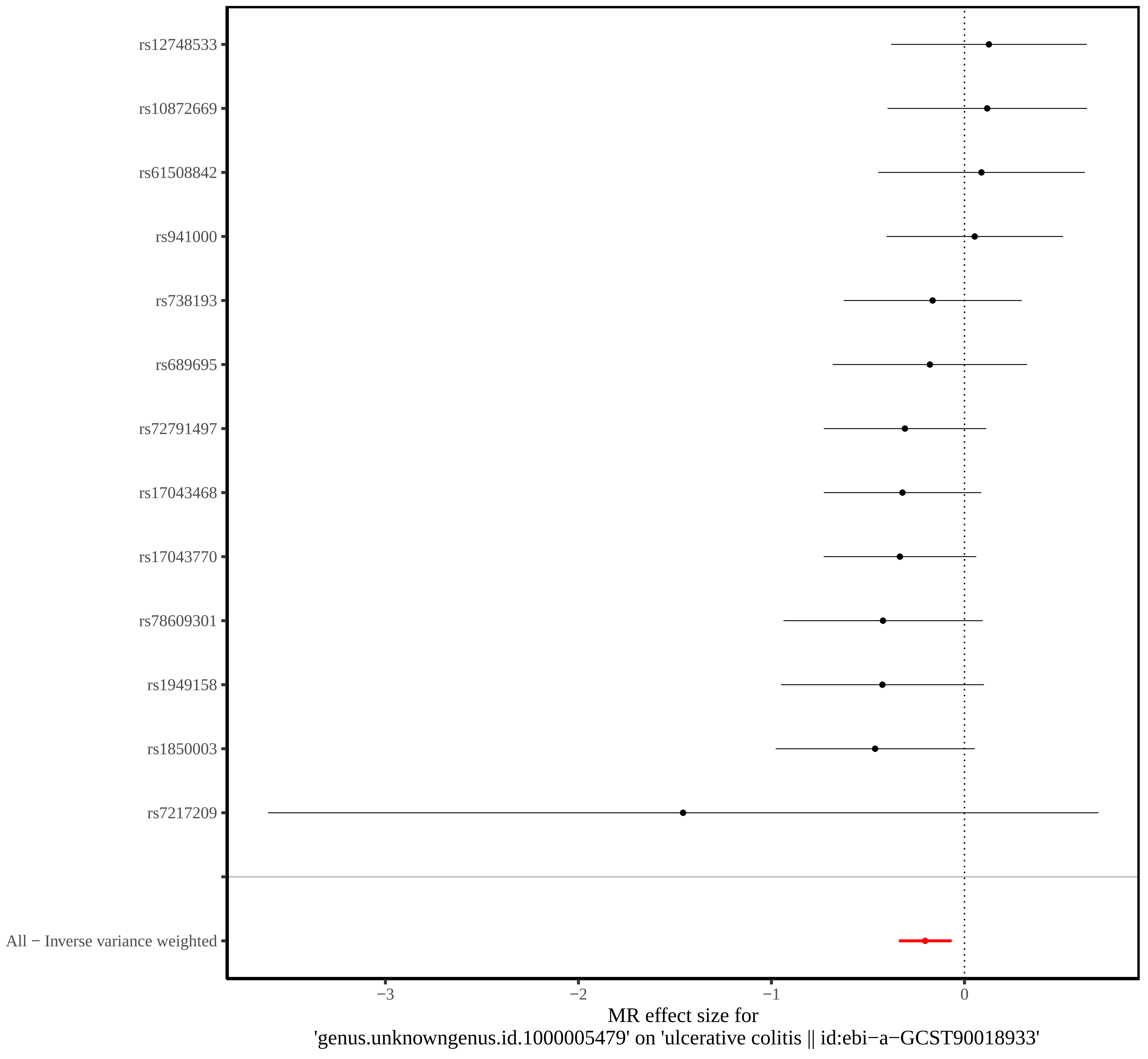
rs12748533
rs10872669
rs61508842
rs941000
rs738193
rs689695
rs72791497
rs17043468
rs17043770
rs78609301
rs1949158
rs1850003
rs7217209
All − Inverse variance weighted
−3
−2
−1
0
MR effect size for
'genus.unknowngenus.id.1000005479' on 'ulcerative colitis || id:ebi−a−GCST90018933'

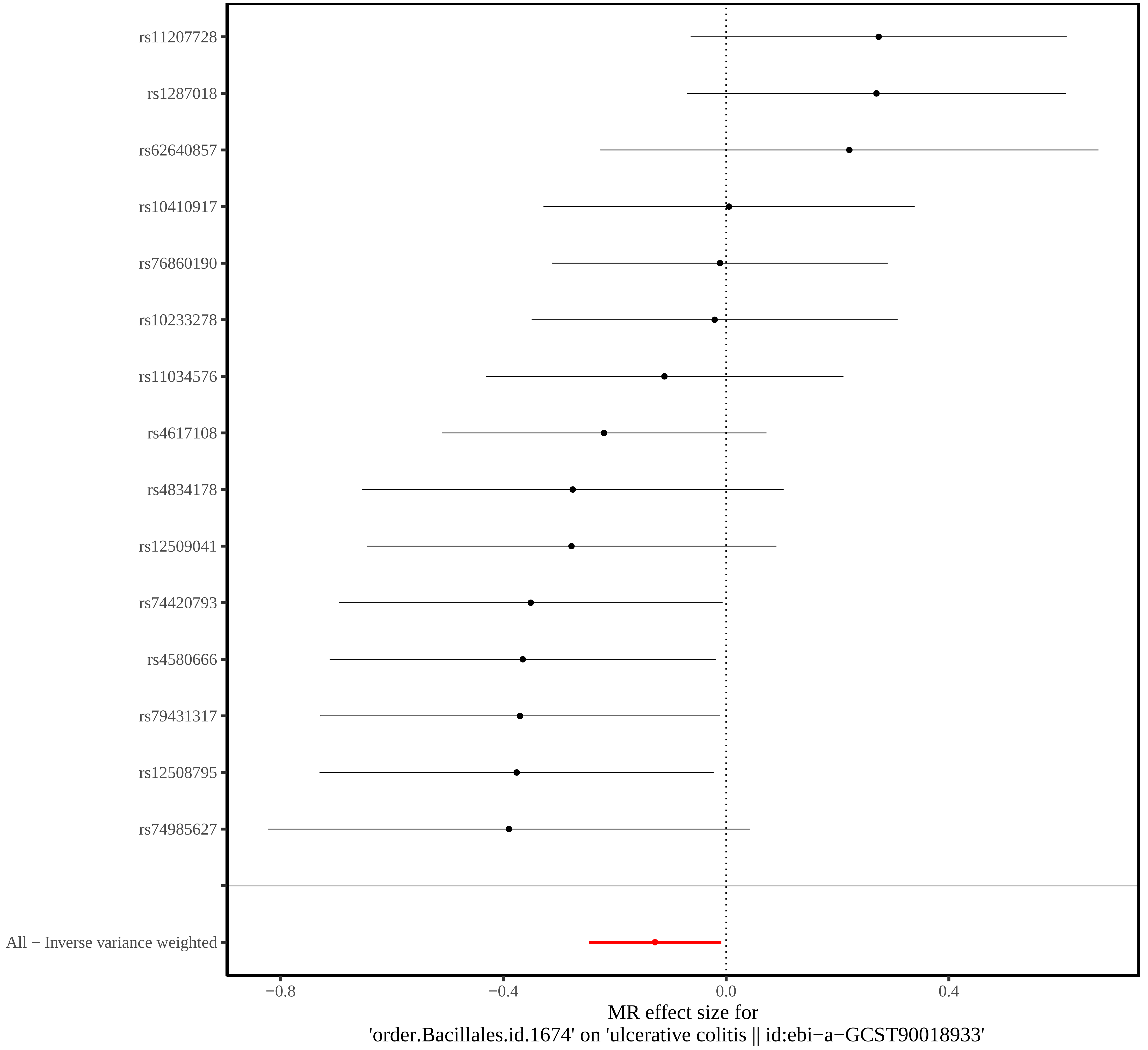

rs11207728
rs1287018
rs62640857
rs10410917
rs76860190
rs10233278
rs11034576
rs4617108
rs4834178
rs12509041
rs74420793
rs4580666
rs79431317
rs12508795
rs74985627
All − Inverse variance weighted
−0.8
−0.4
0.0
0.4
MR effect size for
'order.Bacillales.id.1674' on 'ulcerative colitis || id:ebi−a−GCST90018933'

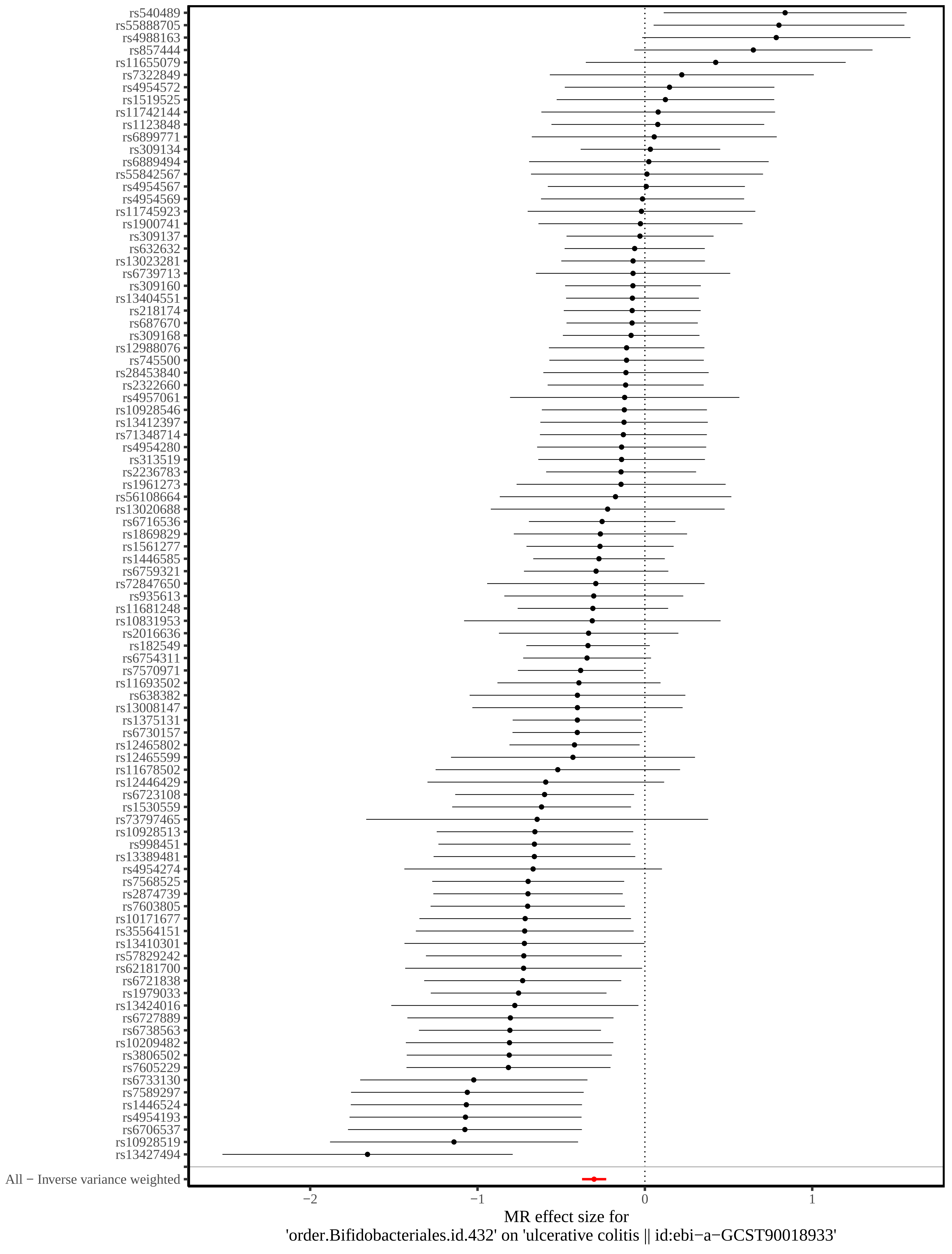

rs540489
rs55888705
rs4988163
rs857444
rs11655079
rs7322849
rs4954572
rs1519525
rs11742144
rs1123848
rs6899771
rs309134
rs6889494
rs55842567
rs4954567
rs4954569
rs11745923
rs1900741
rs309137
rs632632
rs13023281
rs6739713
rs309160
rs13404551
rs218174
rs687670
rs309168
rs12988076
rs745500
rs28453840
rs2322660
rs4957061
rs10928546
rs13412397
rs71348714
rs4954280
rs313519
rs2236783
rs1961273
rs56108664
rs13020688
rs6716536
rs1869829
rs1561277
rs1446585
rs6759321
rs72847650
rs935613
rs11681248
rs10831953
rs2016636
rs182549
rs6754311
rs7570971
rs11693502
rs638382
rs13008147
rs1375131
rs6730157
rs12465802
rs12465599
rs11678502
rs12446429
rs6723108
rs1530559
rs73797465
rs10928513
rs998451
rs13389481
rs4954274
rs7568525
rs2874739
rs7603805
rs10171677
rs35564151
rs13410301
rs57829242
rs62181700
rs6721838
rs1979033
rs13424016
rs6727889
rs6738563
rs10209482
rs3806502
rs7605229
rs6733130
rs7589297
rs1446524
rs4954193
rs6706537
rs10928519
rs13427494
All − Inverse variance weighted
−2
−1
0
1
MR effect size for
'order.Bifidobacteriales.id.432' on 'ulcerative colitis || id:ebi−a−GCST90018933'

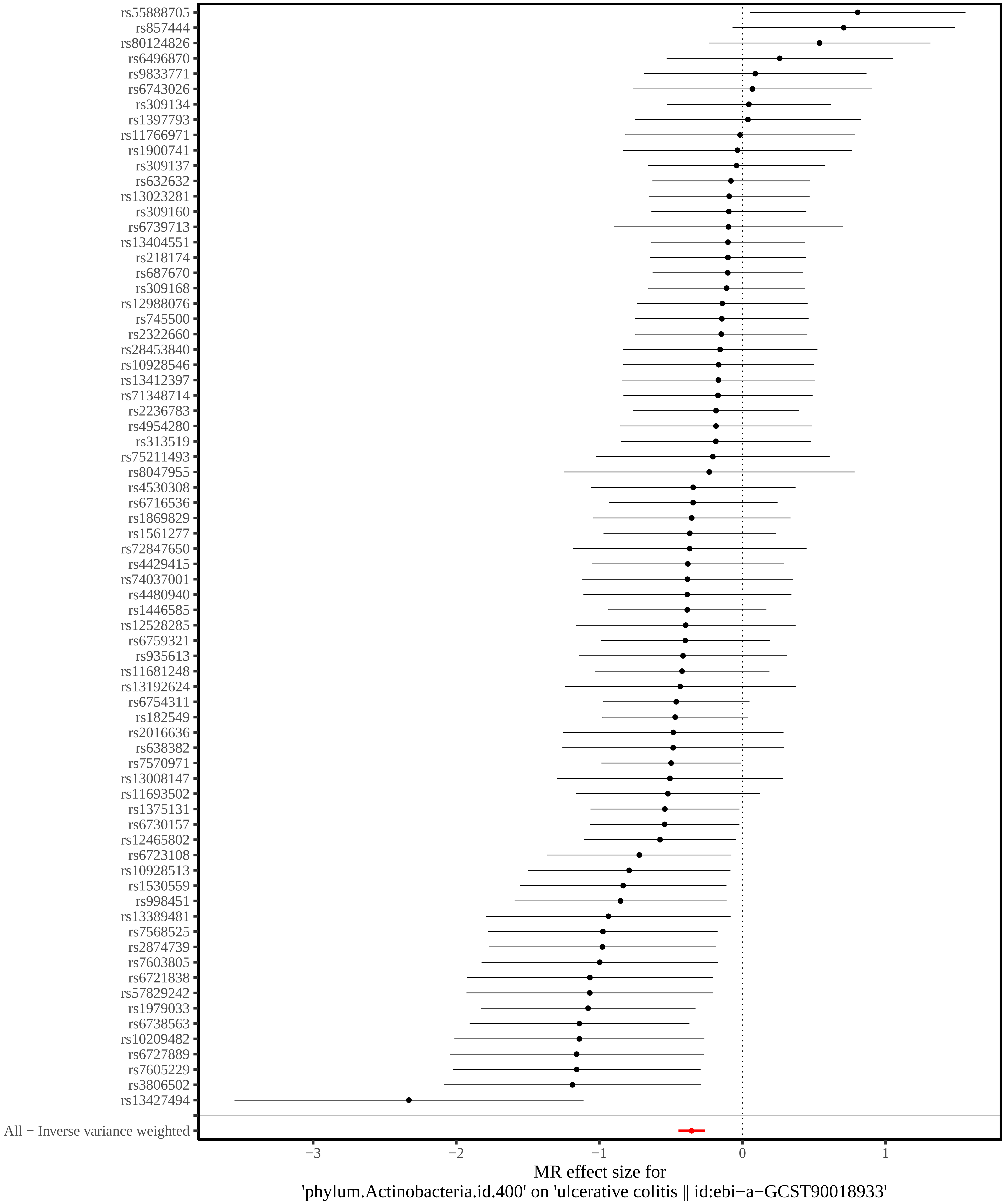

rs55888705
rs857444
rs80124826
rs6496870
rs9833771
rs6743026
rs309134
rs1397793
rs11766971
rs1900741
rs309137
rs632632
rs13023281
rs309160
rs6739713
rs13404551
rs218174
rs687670
rs309168
rs12988076
rs745500
rs2322660
rs28453840
rs10928546
rs13412397
rs71348714
rs2236783
rs4954280
rs313519
rs75211493
rs8047955
rs4530308
rs6716536
rs1869829
rs1561277
rs72847650
rs4429415
rs74037001
rs4480940
rs1446585
rs12528285
rs6759321
rs935613
rs11681248
rs13192624
rs6754311
rs182549
rs2016636
rs638382
rs7570971
rs13008147
rs11693502
rs1375131
rs6730157
rs12465802
rs6723108
rs10928513
rs1530559
rs998451
rs13389481
rs7568525
rs2874739
rs7603805
rs6721838
rs57829242
rs1979033
rs6738563
rs10209482
rs6727889
rs7605229
rs3806502
rs13427494
All − Inverse variance weighted
−3
−2
−1
0
1
MR effect size for
'phylum.Actinobacteria.id.400' on 'ulcerative colitis || id:ebi−a−GCST90018933'

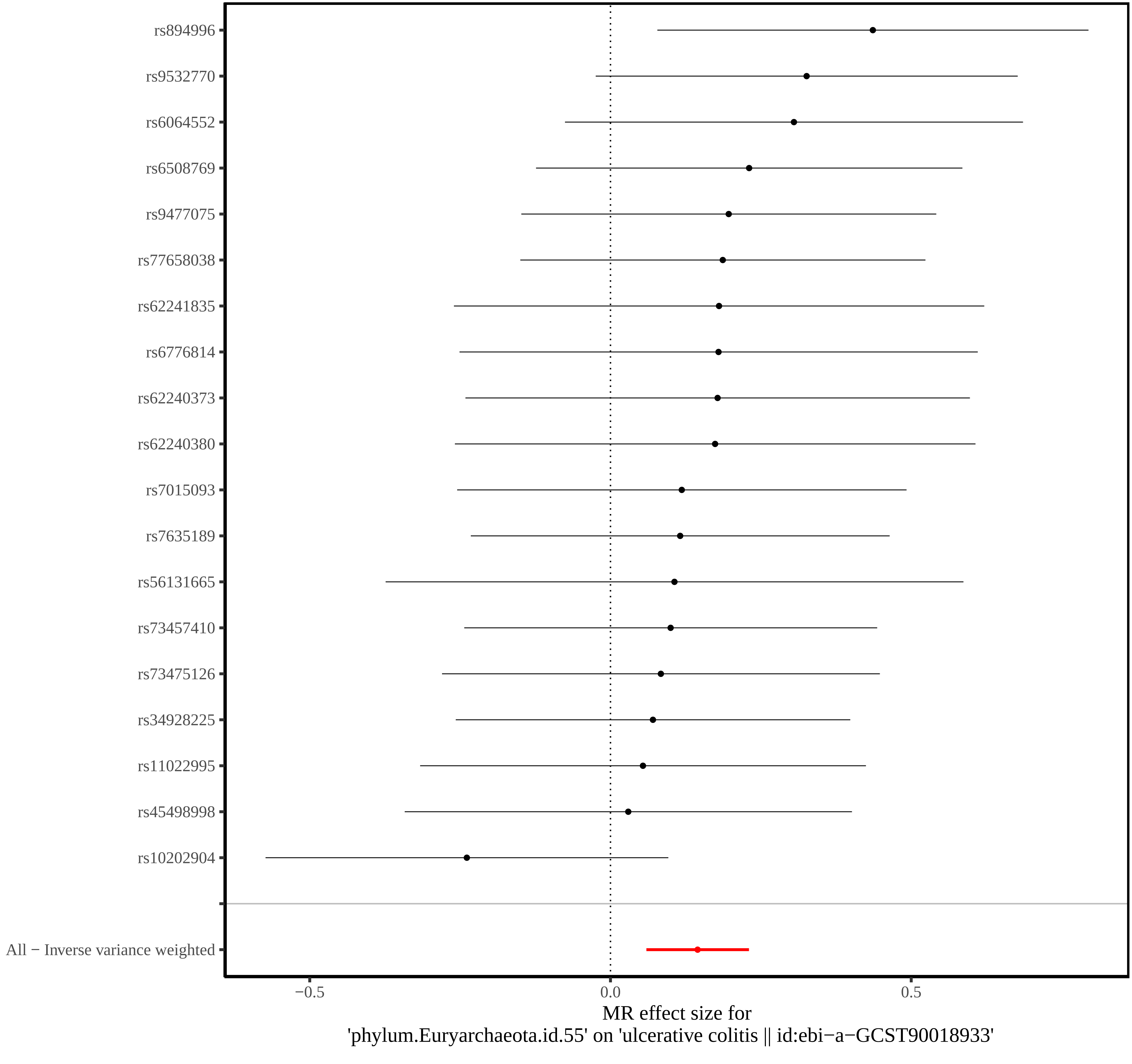

rs894996
rs9532770
rs6064552
rs6508769
rs9477075
rs77658038
rs62241835
rs6776814
rs62240373
rs62240380
rs7015093
rs7635189
rs56131665
rs73457410
rs73475126
rs34928225
rs11022995
rs45498998
rs10202904
All − Inverse variance weighted
−0.5
0.0
0.5
MR effect size for
'phylum.Euryarchaeota.id.55' on 'ulcerative colitis || id:ebi−a−GCST90018933'

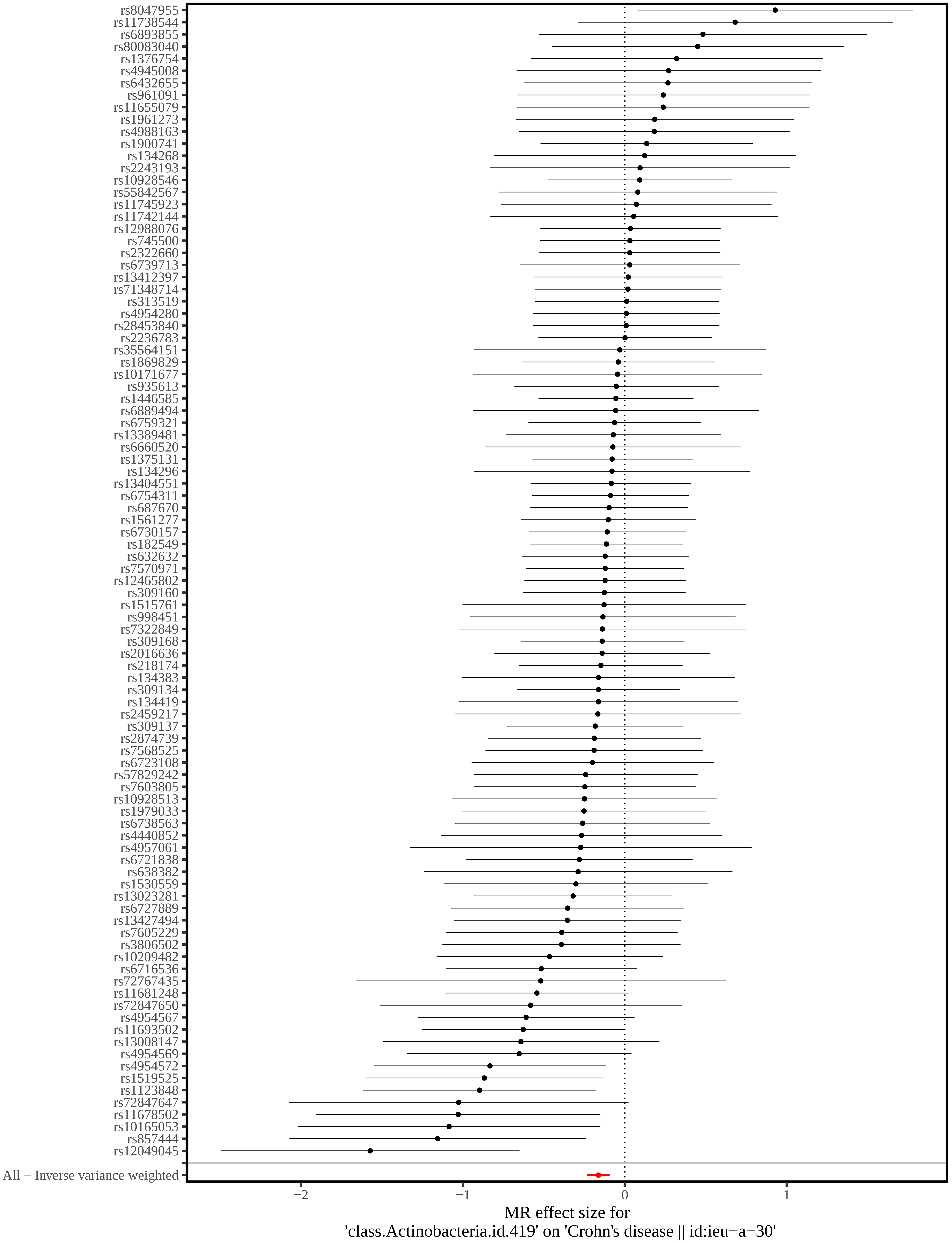
rs8047955
rs11738544
rs6893855
rs80083040
rs1376754
rs4945008
rs6432655
rs961091
rs11655079
rs1961273
rs4988163
rs1900741
rs134268
rs2243193
rs10928546
rs55842567
rs11745923
rs11742144
rs12988076
rs745500
rs2322660
rs6739713
rs13412397
rs71348714
rs313519
rs4954280
rs28453840
rs2236783
rs35564151
rs1869829
rs10171677
rs935613
rs1446585
rs6889494
rs6759321
rs13389481
rs6660520
rs1375131
rs134296
rs13404551
rs6754311
rs687670
rs1561277
rs6730157
rs182549
rs632632
rs7570971
rs12465802
rs309160
rs1515761
rs998451
rs7322849
rs309168
rs2016636
rs218174
rs134383
rs309134
rs134419
rs2459217
rs309137
rs2874739
rs7568525
rs6723108
rs57829242
rs7603805
rs10928513
rs1979033
rs6738563
rs4440852
rs4957061
rs6721838
rs638382
rs1530559
rs13023281
rs6727889
rs13427494
rs7605229
rs3806502
rs10209482
rs6716536
rs72767435
rs11681248
rs72847650
rs4954567
rs11693502
rs13008147
rs4954569
rs4954572
rs1519525
rs1123848
rs72847647
rs11678502
rs10165053
rs857444
rs12049045
All − Inverse variance weighted
−2
−1
0
1
MR effect size for
'class.Actinobacteria.id.419' on 'Crohn's disease || id:ieu−a−30'

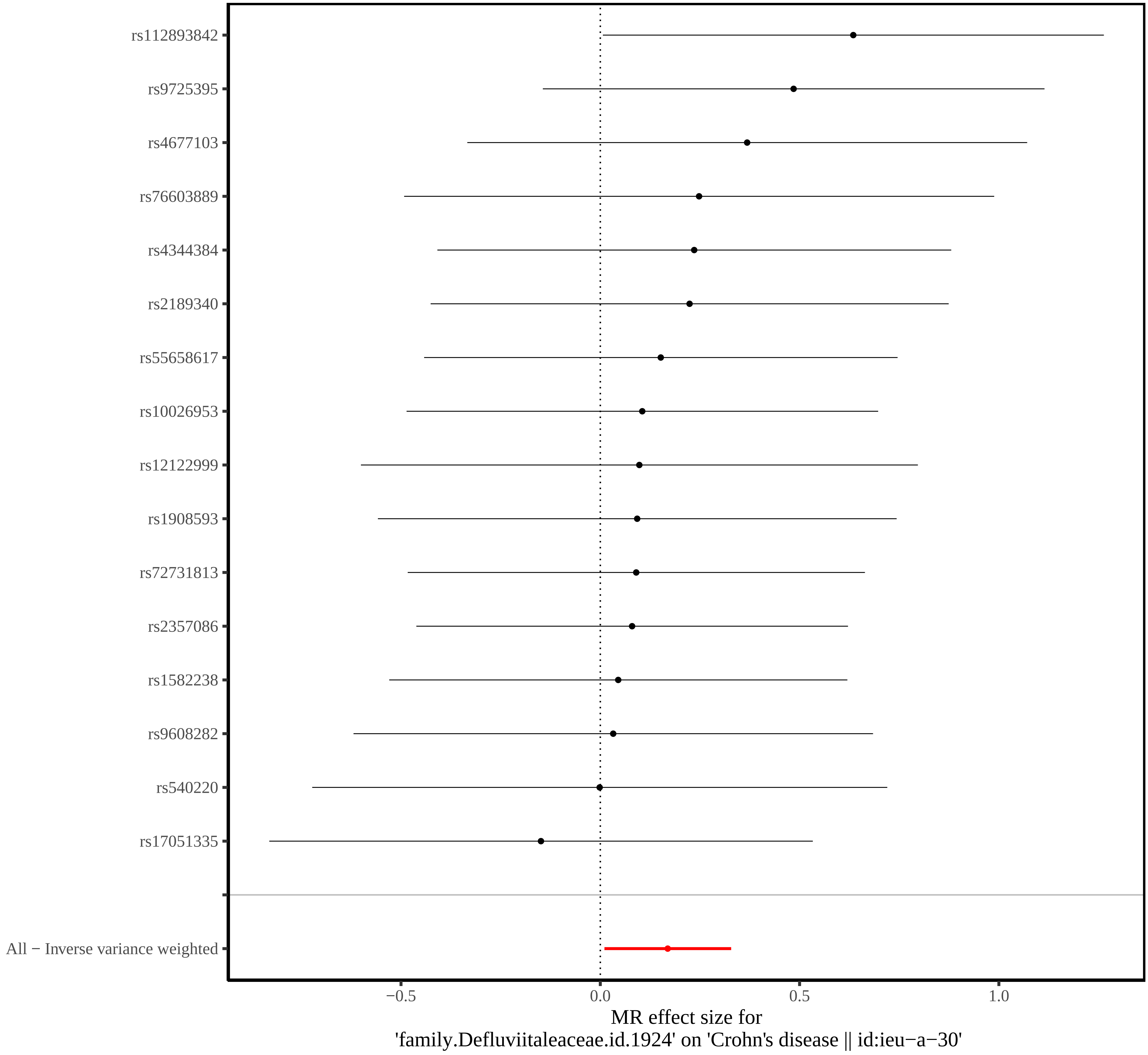
rs112893842
rs9725395
rs4677103
rs76603889
rs4344384
rs2189340
rs55658617
rs10026953
rs12122999
rs1908593
rs72731813
rs2357086
rs1582238
rs9608282
rs540220
rs17051335
All − Inverse variance weighted
−0.5
0.0
0.5
1.0
MR effect size for
'family.Defluviitaleaceae.id.1924' on 'Crohn's disease || id:ieu−a−30'

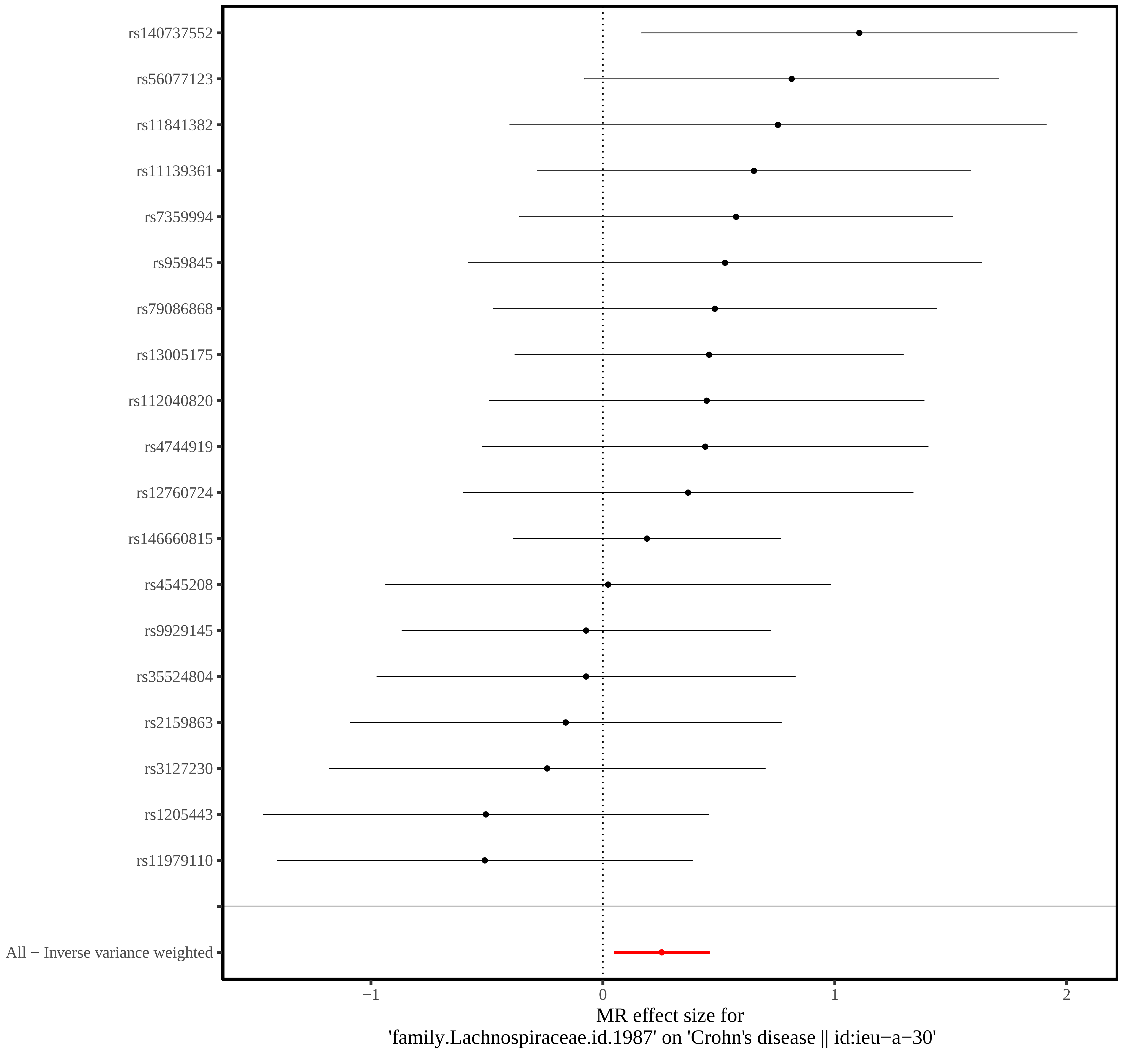

rs140737552
rs56077123
rs11841382
rs11139361
rs7359994
rs959845
rs79086868
rs13005175
rs112040820
rs4744919
rs12760724
rs146660815
rs4545208
rs9929145
rs35524804
rs2159863
rs3127230
rs1205443
rs11979110
All − Inverse variance weighted
−1
0
1
2
MR effect size for
'family.Lachnospiraceae.id.1987' on 'Crohn's disease || id:ieu−a−30'

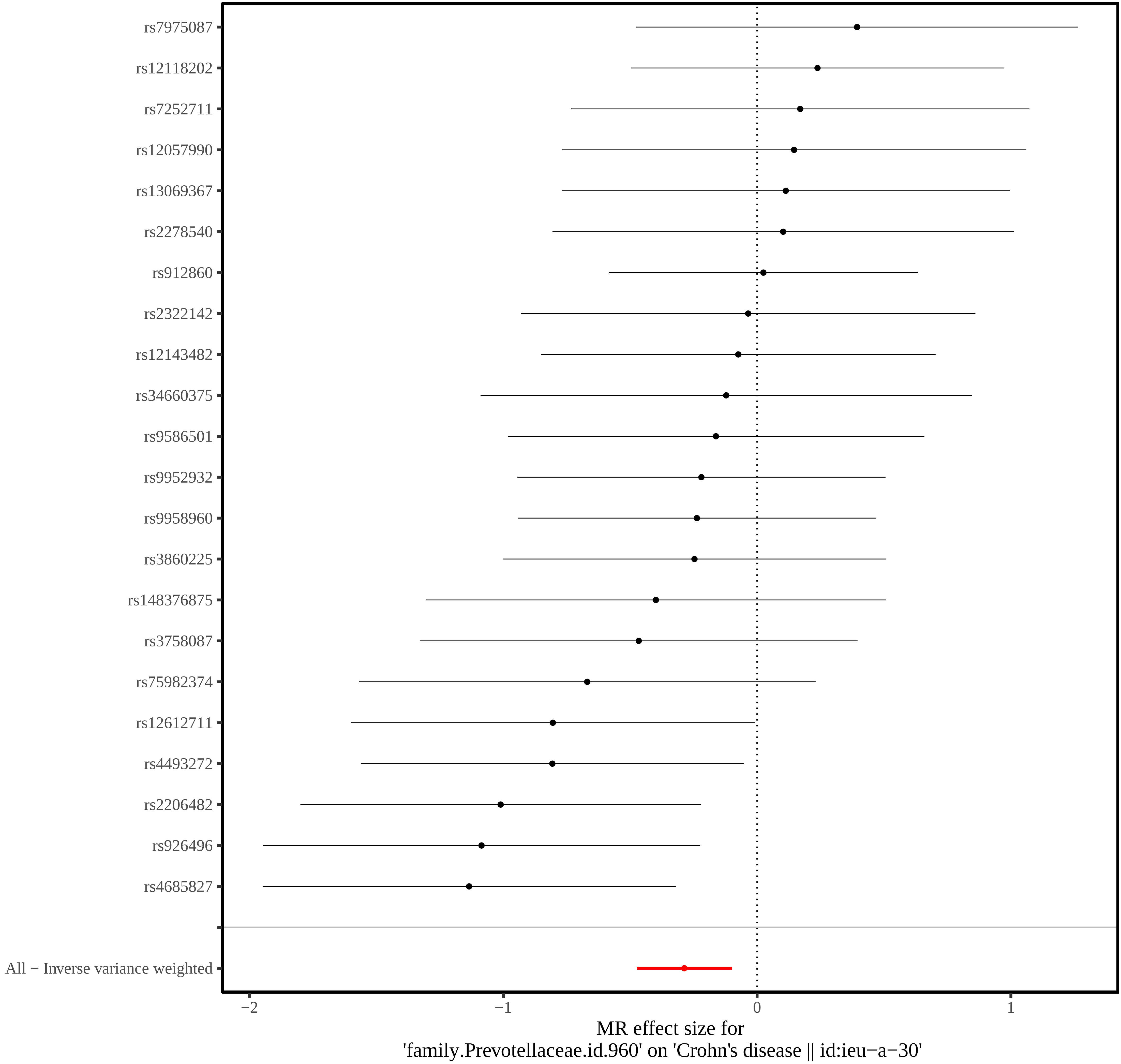
rs7975087
rs12118202
rs7252711
rs12057990
rs13069367
rs2278540
rs912860
rs2322142
rs12143482
rs34660375
rs9586501
rs9952932
rs9958960
rs3860225
rs148376875
rs3758087
rs75982374
rs12612711
rs4493272
rs2206482
rs926496
rs4685827
All − Inverse variance weighted
−2
−1
0
1
MR effect size for
'family.Prevotellaceae.id.960' on 'Crohn's disease || id:ieu−a−30'

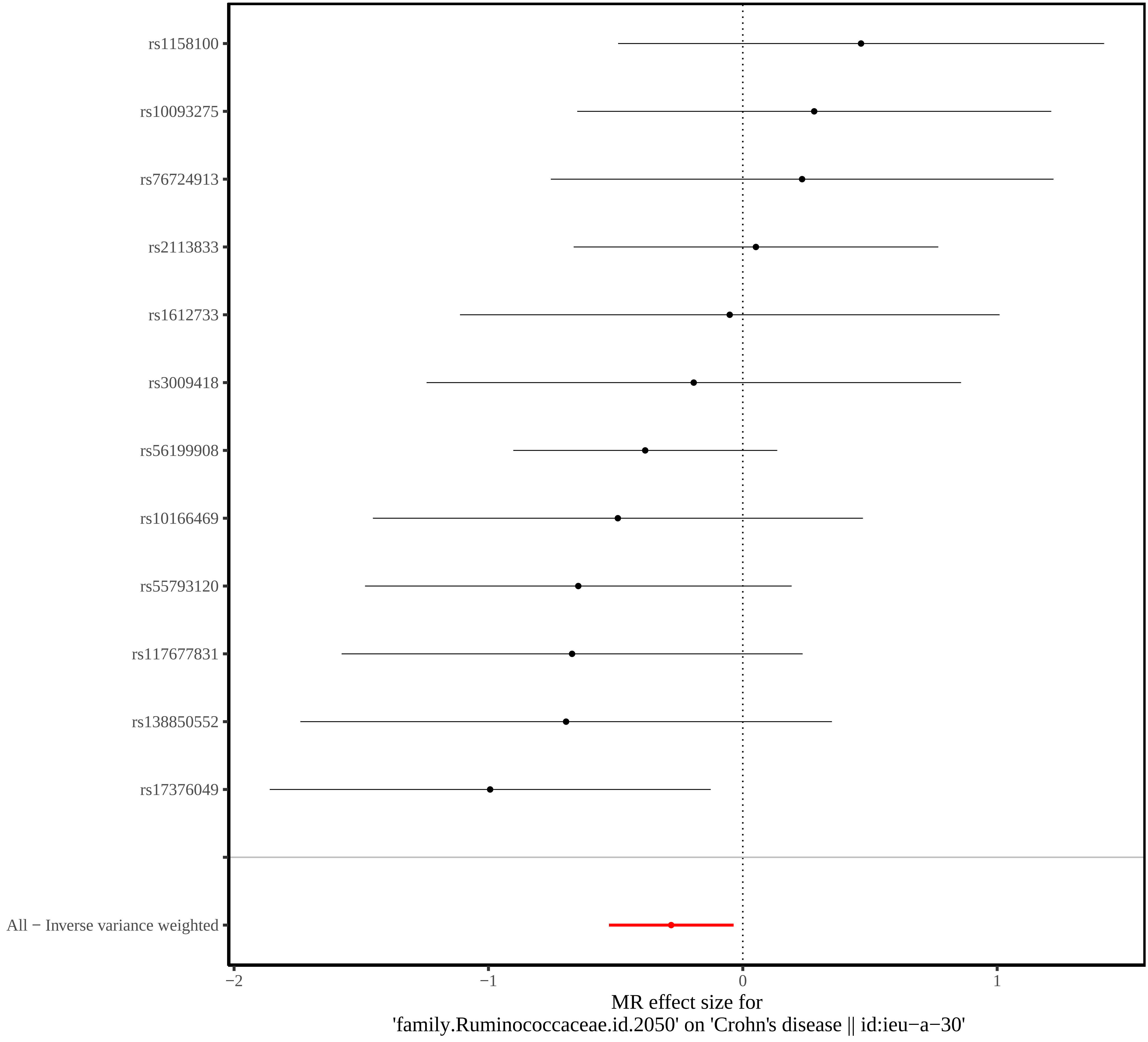

rs1158100
rs10093275
rs76724913
rs2113833
rs1612733
rs3009418
rs56199908
rs10166469
rs55793120
rs117677831
rs138850552
rs17376049
All − Inverse variance weighted
−2
−1
0
1
MR effect size for
'family.Ruminococcaceae.id.2050' on 'Crohn's disease || id:ieu−a−30'

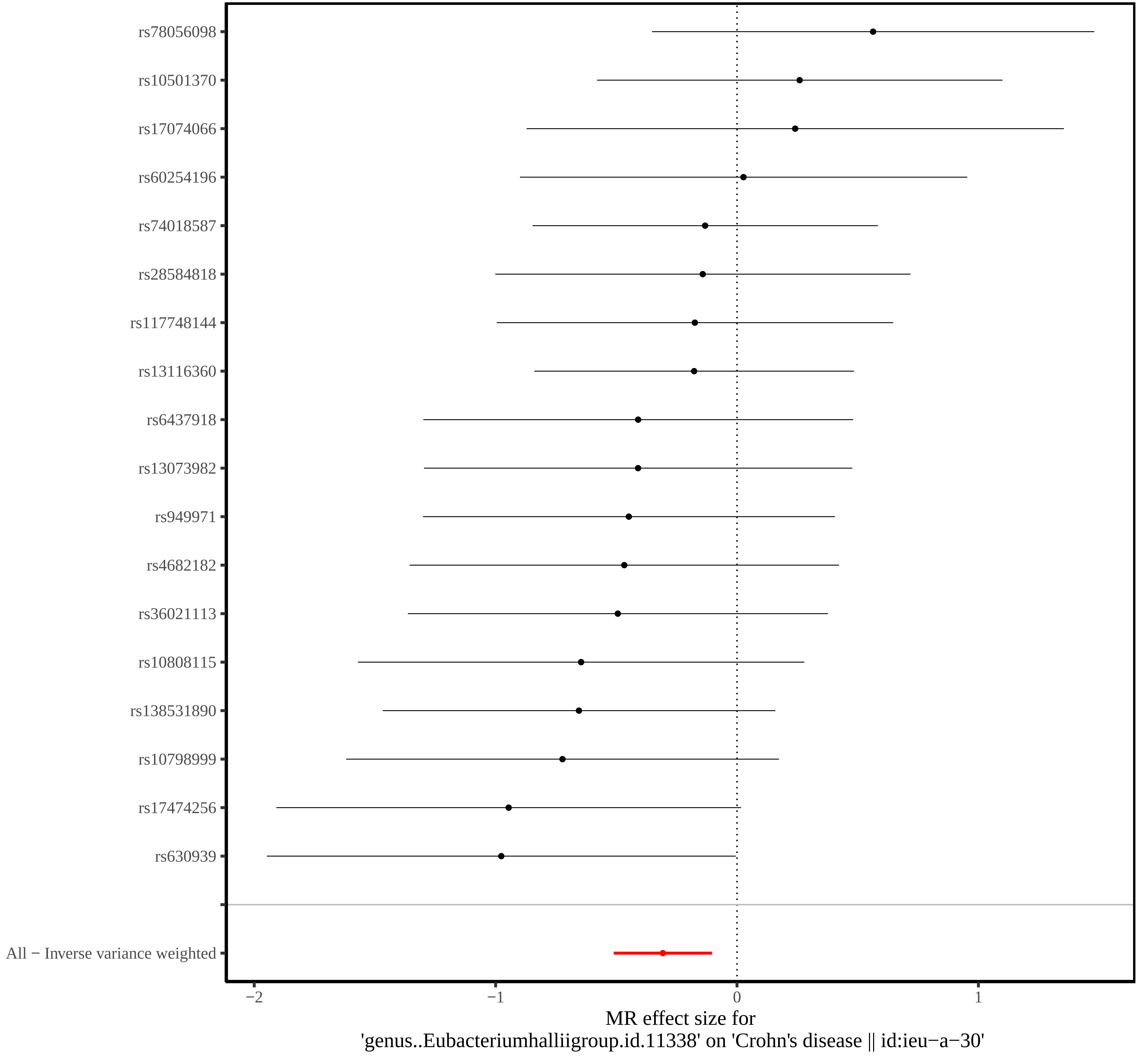
rs78056098
rs10501370
rs17074066
rs60254196
rs74018587
rs28584818
rs117748144
rs13116360
rs6437918
rs13073982
rs949971
rs4682182
rs36021113
rs10808115
rs138531890
rs10798999
rs17474256
rs630939
All − Inverse variance weighted
−2
−1
0
1
MR effect size for
'genus..Eubacteriumhalliigroup.id.11338' on 'Crohn's disease || id:ieu−a−30'

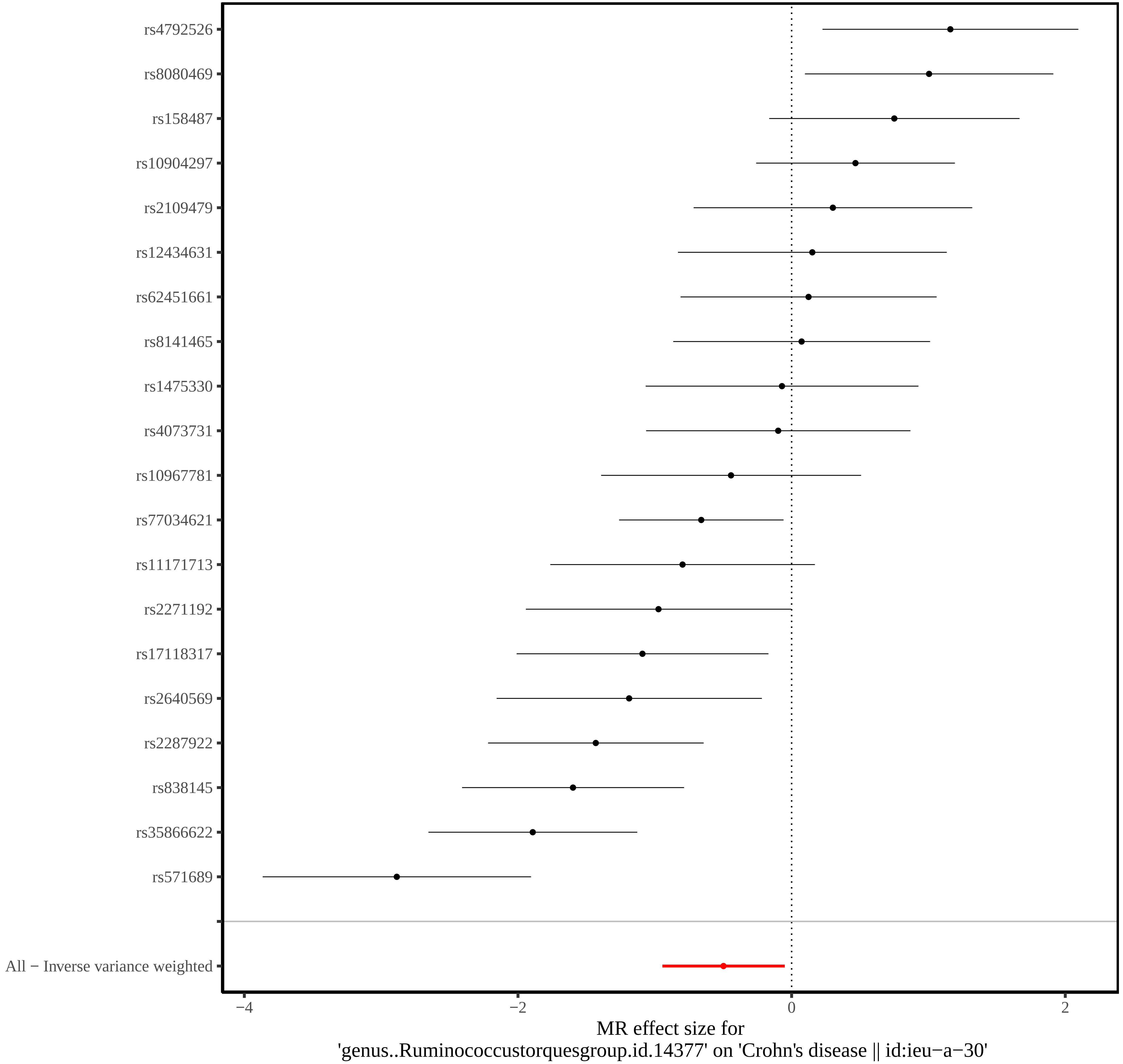

rs4792526
rs8080469
rs158487
rs10904297
rs2109479
rs12434631
rs62451661
rs8141465
rs1475330
rs4073731
rs10967781
rs77034621
rs11171713
rs2271192
rs17118317
rs2640569
rs2287922
rs838145
rs35866622
rs571689
All − Inverse variance weighted
−4
−2
0
2
MR effect size for
'genus..Ruminococcustorquesgroup.id.14377' on 'Crohn's disease || id:ieu−a−30'

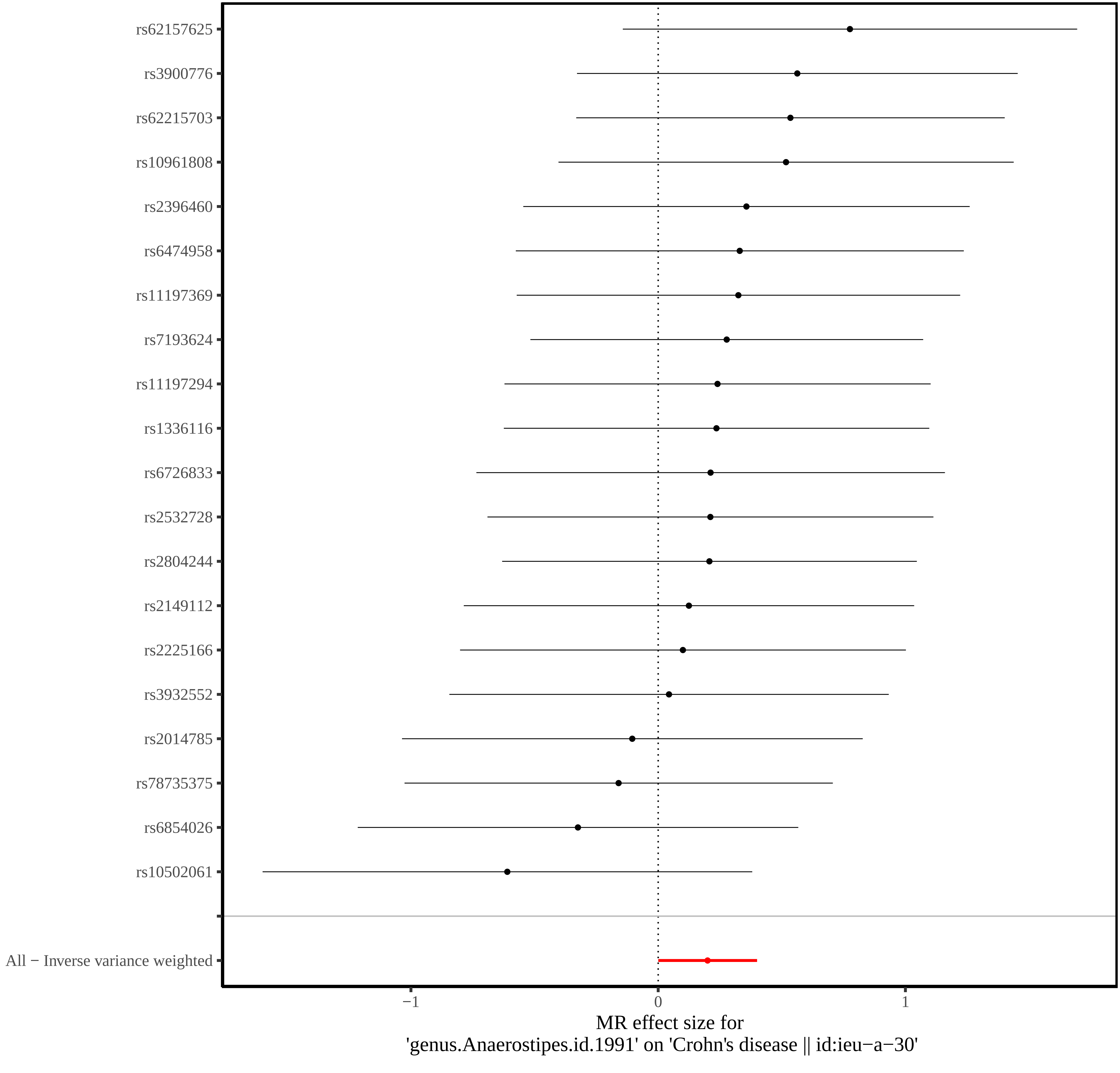
rs62157625
rs3900776
rs62215703
rs10961808
rs2396460
rs6474958
rs11197369
rs7193624
rs11197294
rs1336116
rs6726833
rs2532728
rs2804244
rs2149112
rs2225166
rs3932552
rs2014785
rs78735375
rs6854026
rs10502061
All − Inverse variance weighted
−1
0
1
MR effect size for
'genus.Anaerostipes.id.1991' on 'Crohn's disease || id:ieu−a−30'

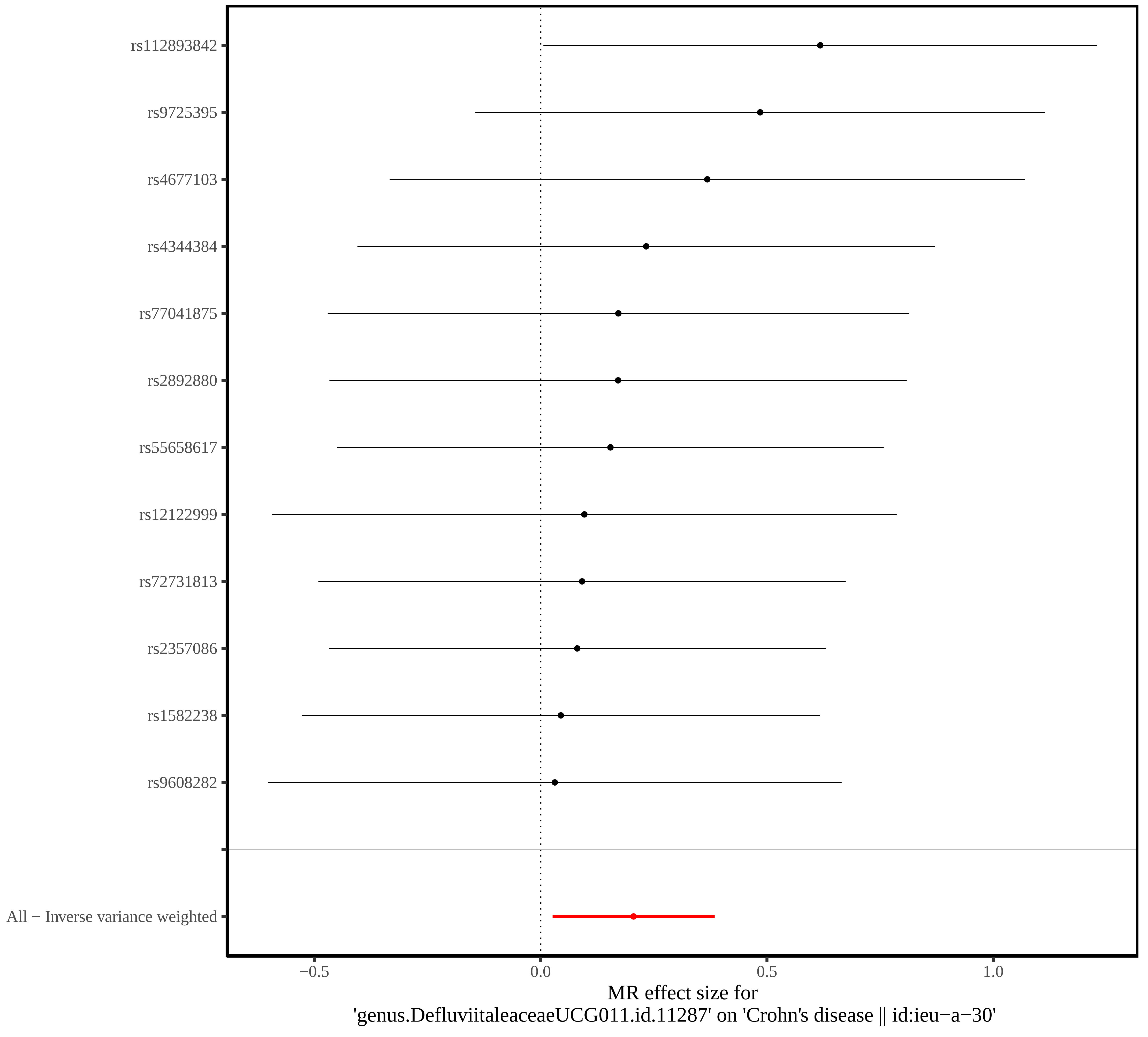

rs112893842
rs9725395
rs4677103
rs4344384
rs77041875
rs2892880
rs55658617
rs12122999
rs72731813
rs2357086
rs1582238
rs9608282
All − Inverse variance weighted
−0.5
0.0
0.5
1.0
MR effect size for
'genus.DefluviitaleaceaeUCG011.id.11287' on 'Crohn's disease || id:ieu−a−30'

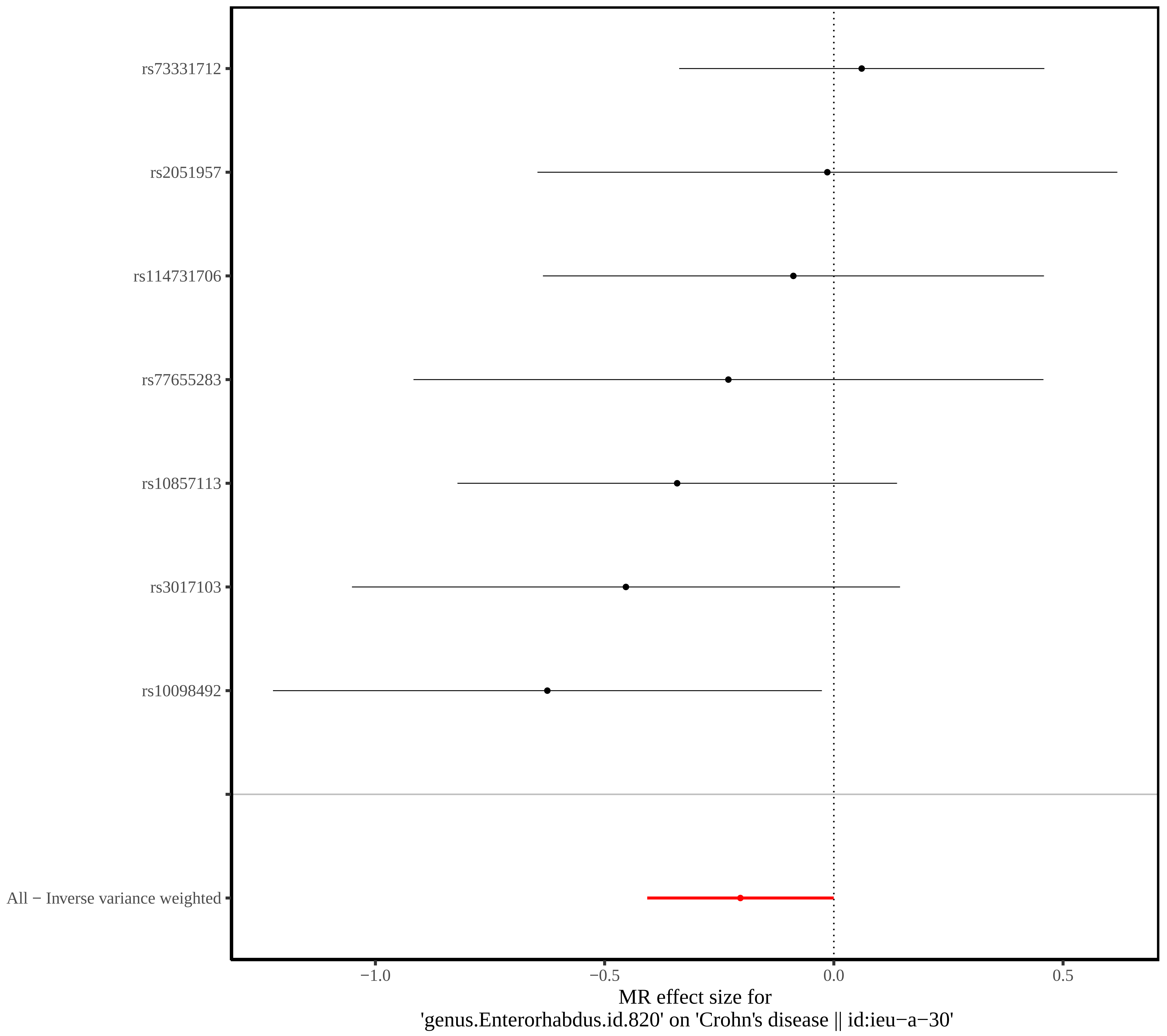
rs73331712
rs2051957
rs114731706
rs77655283
rs10857113
rs3017103
rs10098492
All − Inverse variance weighted
−1.0
−0.5
0.0
0.5
MR effect size for
'genus.Enterorhabdus.id.820' on 'Crohn's disease || id:ieu−a−30'

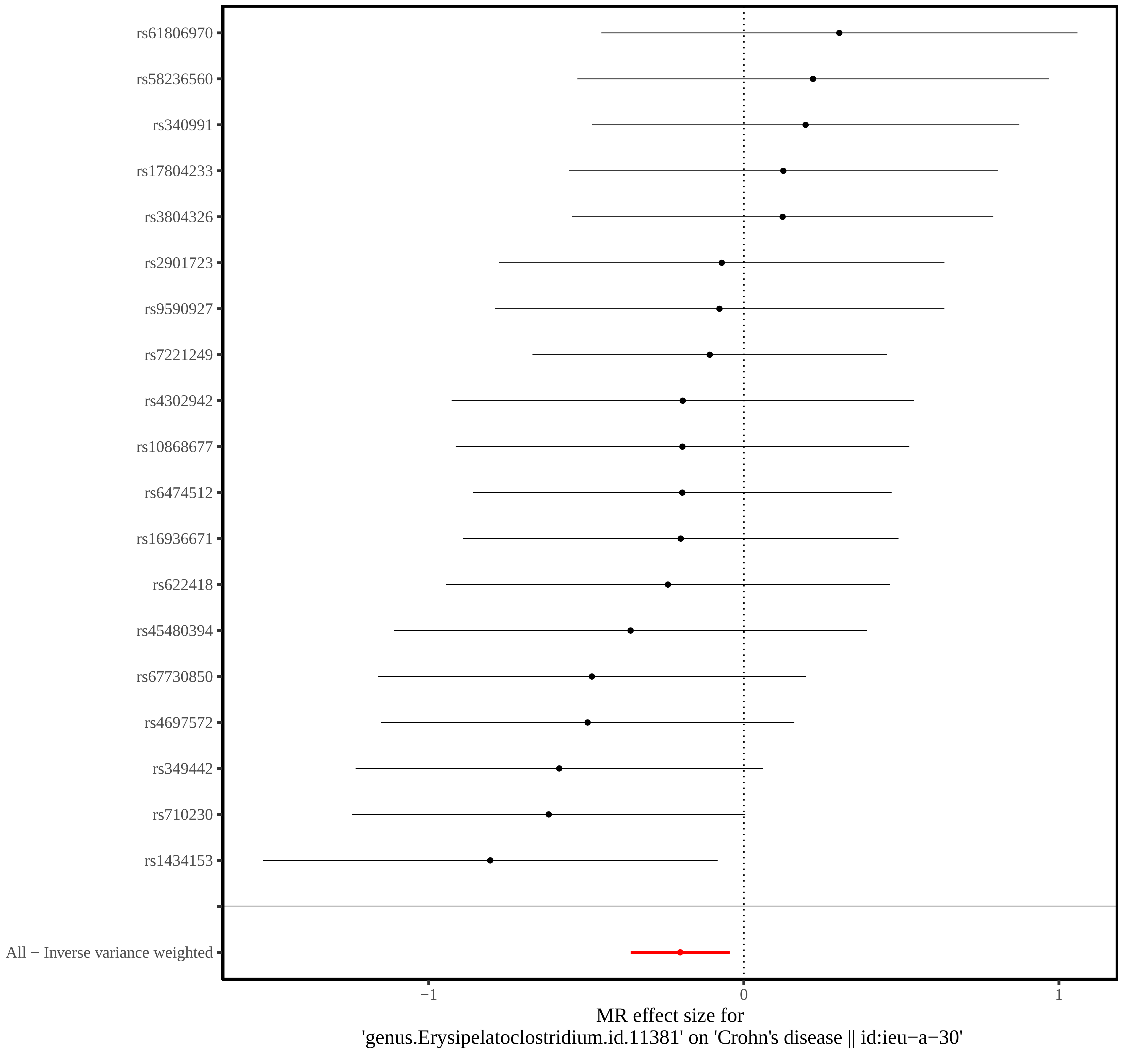
rs61806970
rs58236560
rs340991
rs17804233
rs3804326
rs2901723
rs9590927
rs7221249
rs4302942
rs10868677
rs6474512
rs16936671
rs622418
rs45480394
rs67730850
rs4697572
rs349442
rs710230
rs1434153
All − Inverse variance weighted
−1
0
1
MR effect size for
'genus.Erysipelatoclostridium.id.11381' on 'Crohn's disease || id:ieu−a−30'

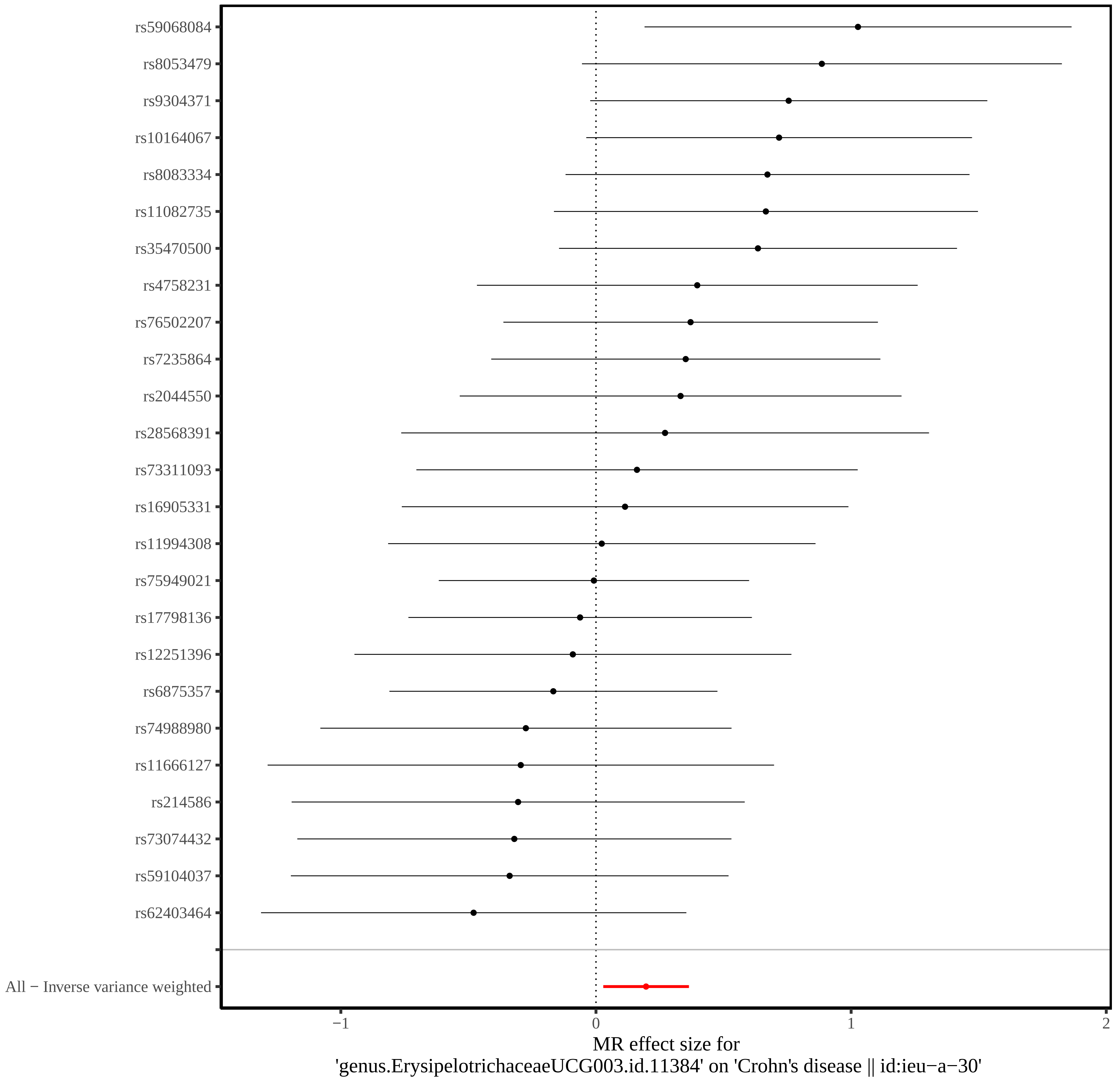
rs59068084
rs8053479
rs9304371
rs10164067
rs8083334
rs11082735
rs35470500
rs4758231
rs76502207
rs7235864
rs2044550
rs28568391
rs73311093
rs16905331
rs11994308
rs75949021
rs17798136
rs12251396
rs6875357
rs74988980
rs11666127
rs214586
rs73074432
rs59104037
rs62403464
All − Inverse variance weighted
−1
0
1
2
MR effect size for
'genus.ErysipelotrichaceaeUCG003.id.11384' on 'Crohn's disease || id:ieu−a−30'

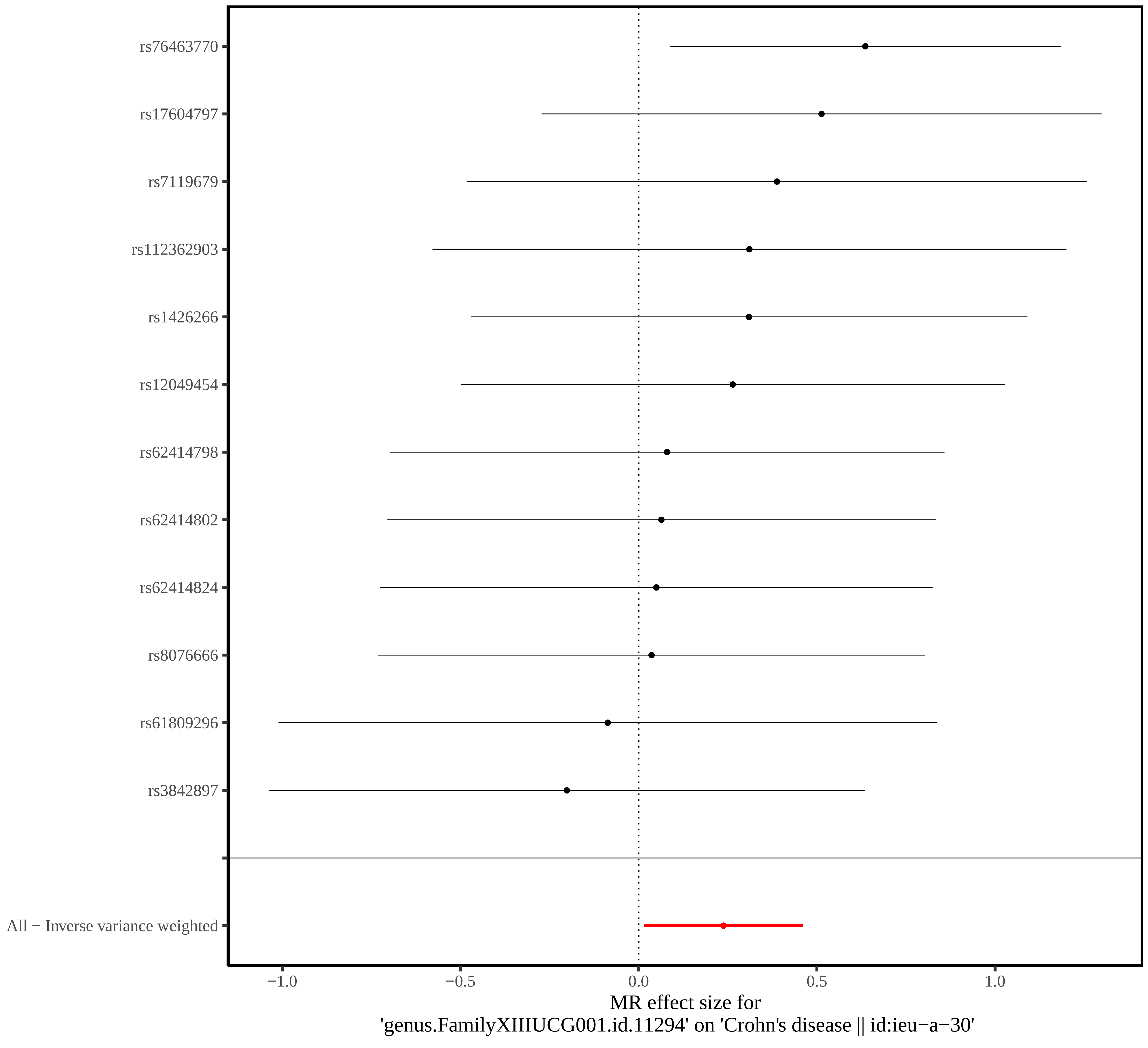

rs76463770
rs17604797
rs7119679
rs112362903
rs1426266
rs12049454
rs62414798
rs62414802
rs62414824
rs8076666
rs61809296
rs3842897
All − Inverse variance weighted
−1.0
−0.5
0.0
0.5
1.0
MR effect size for
'genus.FamilyXIIIUCG001.id.11294' on 'Crohn's disease || id:ieu−a−30'

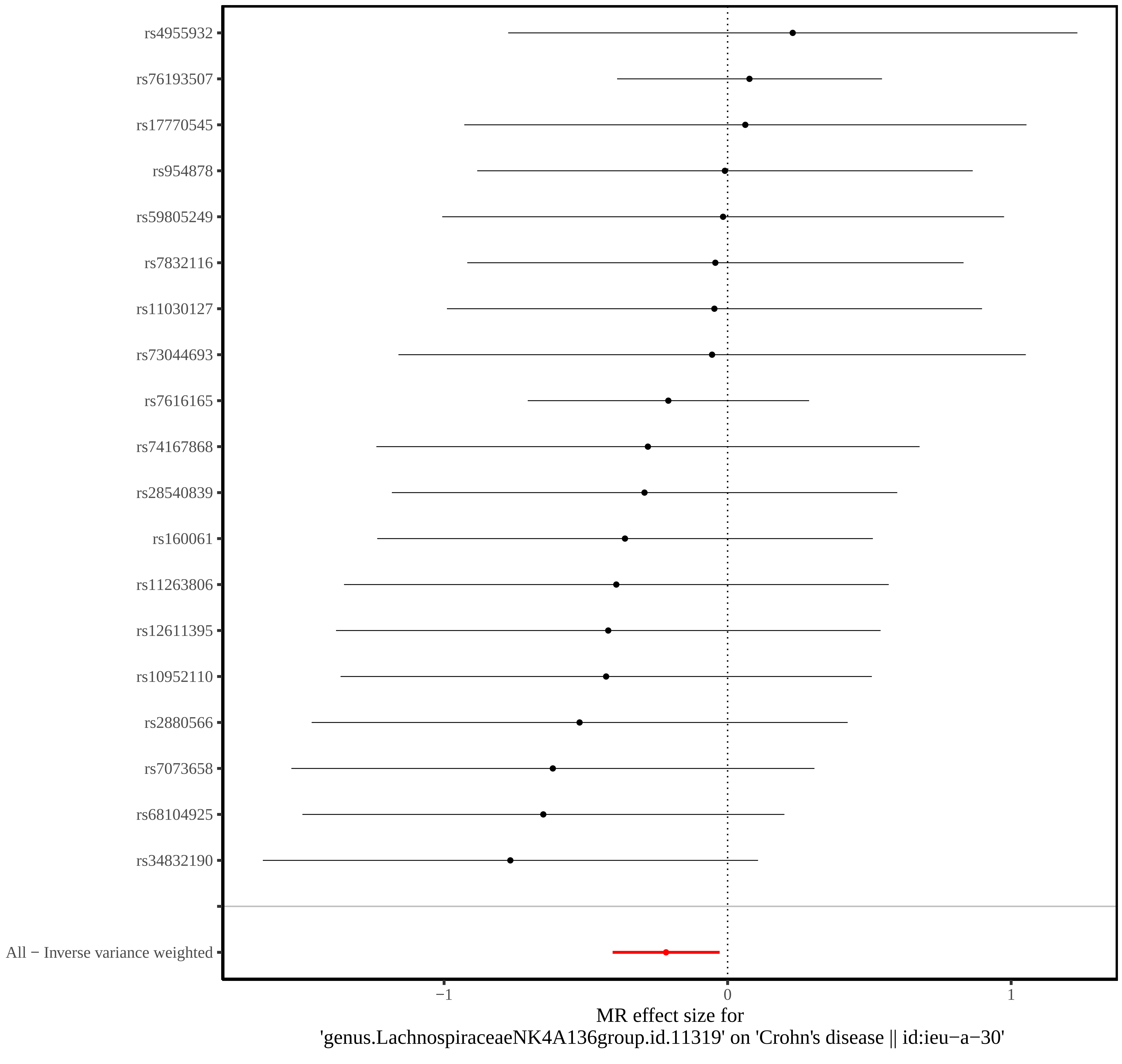

rs4955932
rs76193507
rs17770545
rs954878
rs59805249
rs7832116
rs11030127
rs73044693
rs7616165
rs74167868
rs28540839
rs160061
rs11263806
rs12611395
rs10952110
rs2880566
rs7073658
rs68104925
rs34832190
All − Inverse variance weighted
−1
0
1
MR effect size for
'genus.LachnospiraceaeNK4A136group.id.11319' on 'Crohn's disease || id:ieu−a−30'

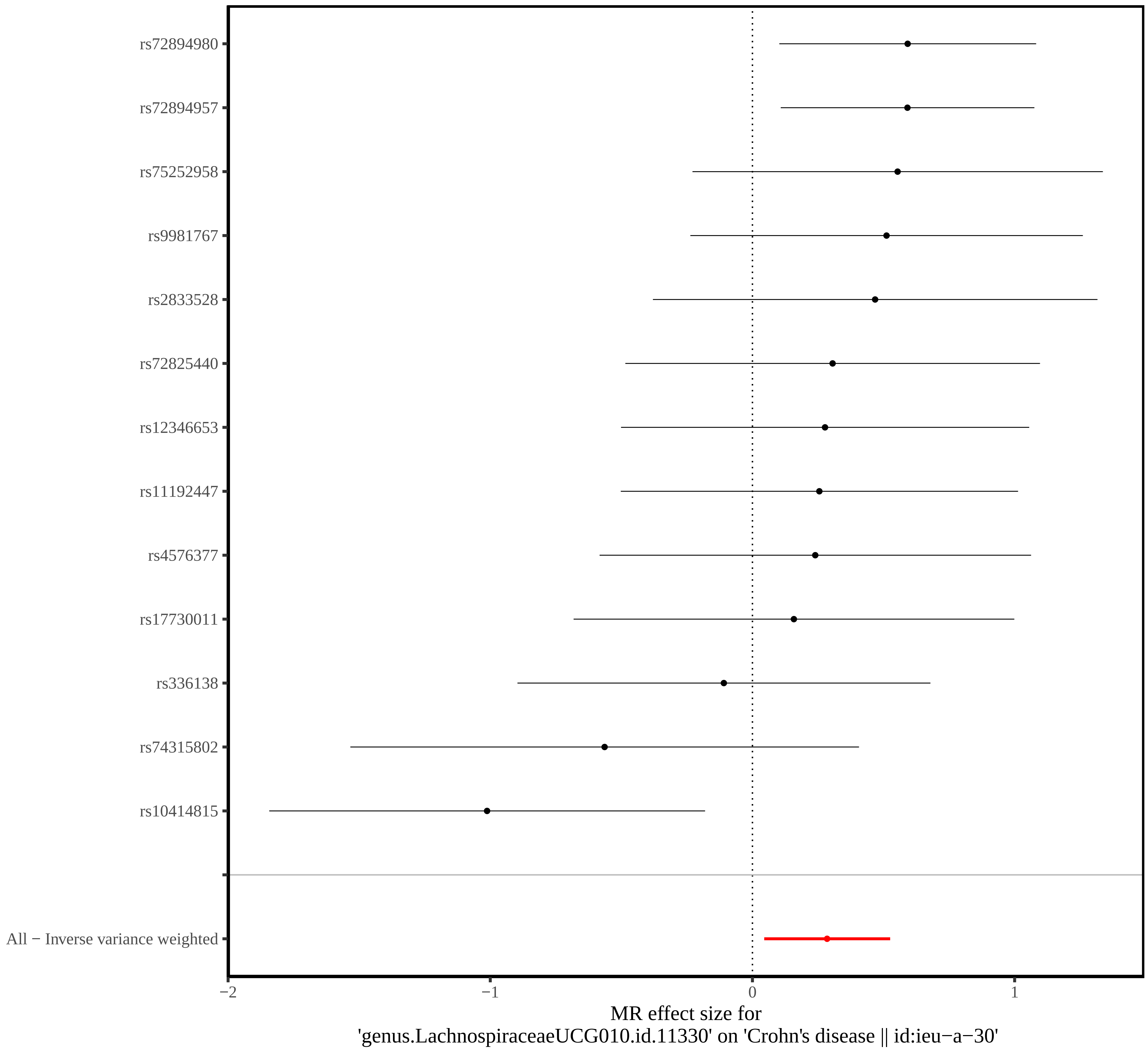
rs72894980
rs72894957
rs75252958
rs9981767
rs2833528
rs72825440
rs12346653
rs11192447
rs4576377
rs17730011
rs336138
rs74315802
rs10414815
All − Inverse variance weighted
−2
−1
0
1
MR effect size for
'genus.LachnospiraceaeUCG010.id.11330' on 'Crohn's disease || id:ieu−a−30'

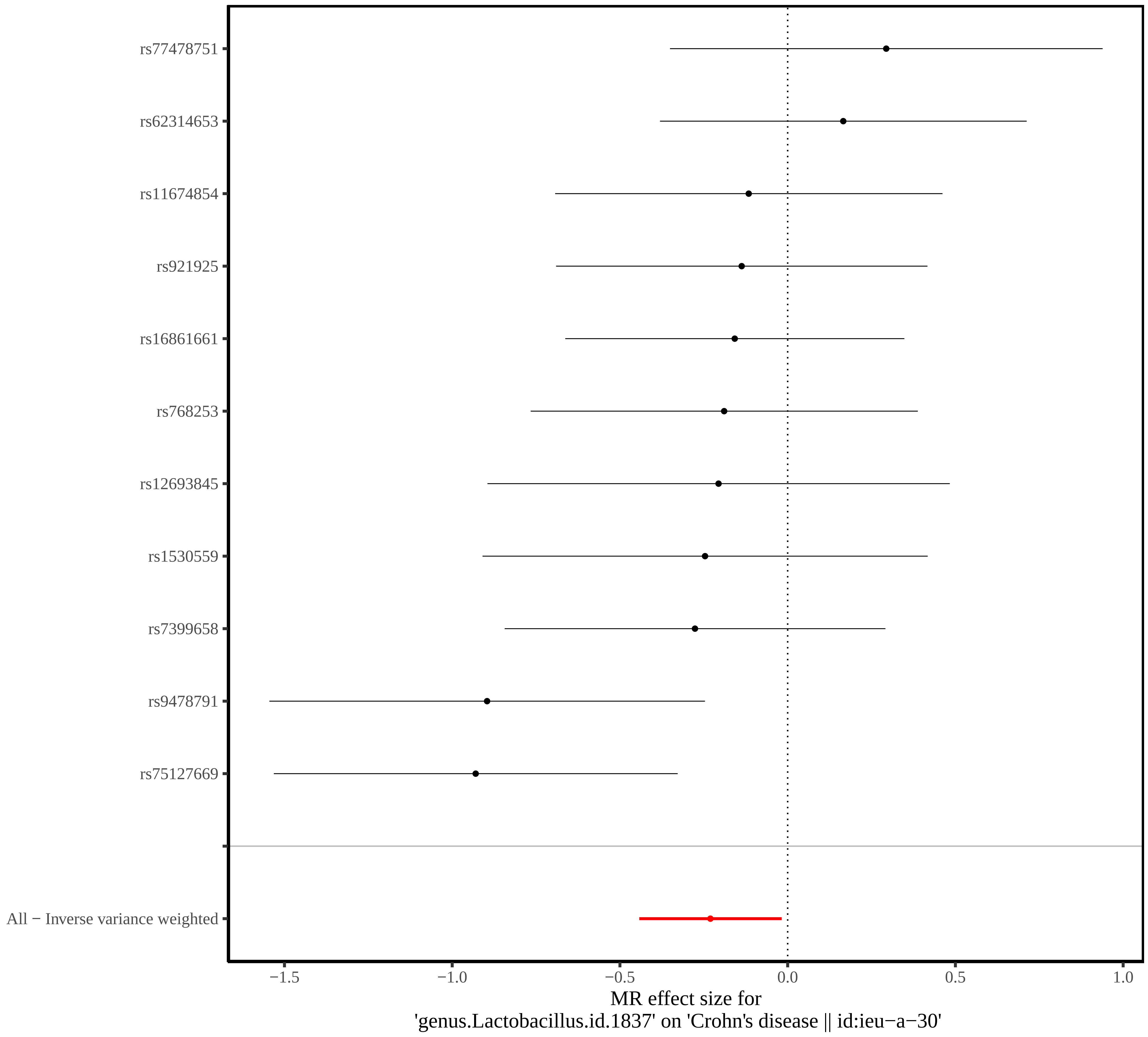

rs77478751
rs62314653
rs11674854
rs921925
rs16861661
rs768253
rs12693845
rs1530559
rs7399658
rs9478791
rs75127669
All − Inverse variance weighted
−1.5
−1.0
−0.5
0.0
0.5
1.0
MR effect size for
'genus.Lactobacillus.id.1837' on 'Crohn's disease || id:ieu−a−30'

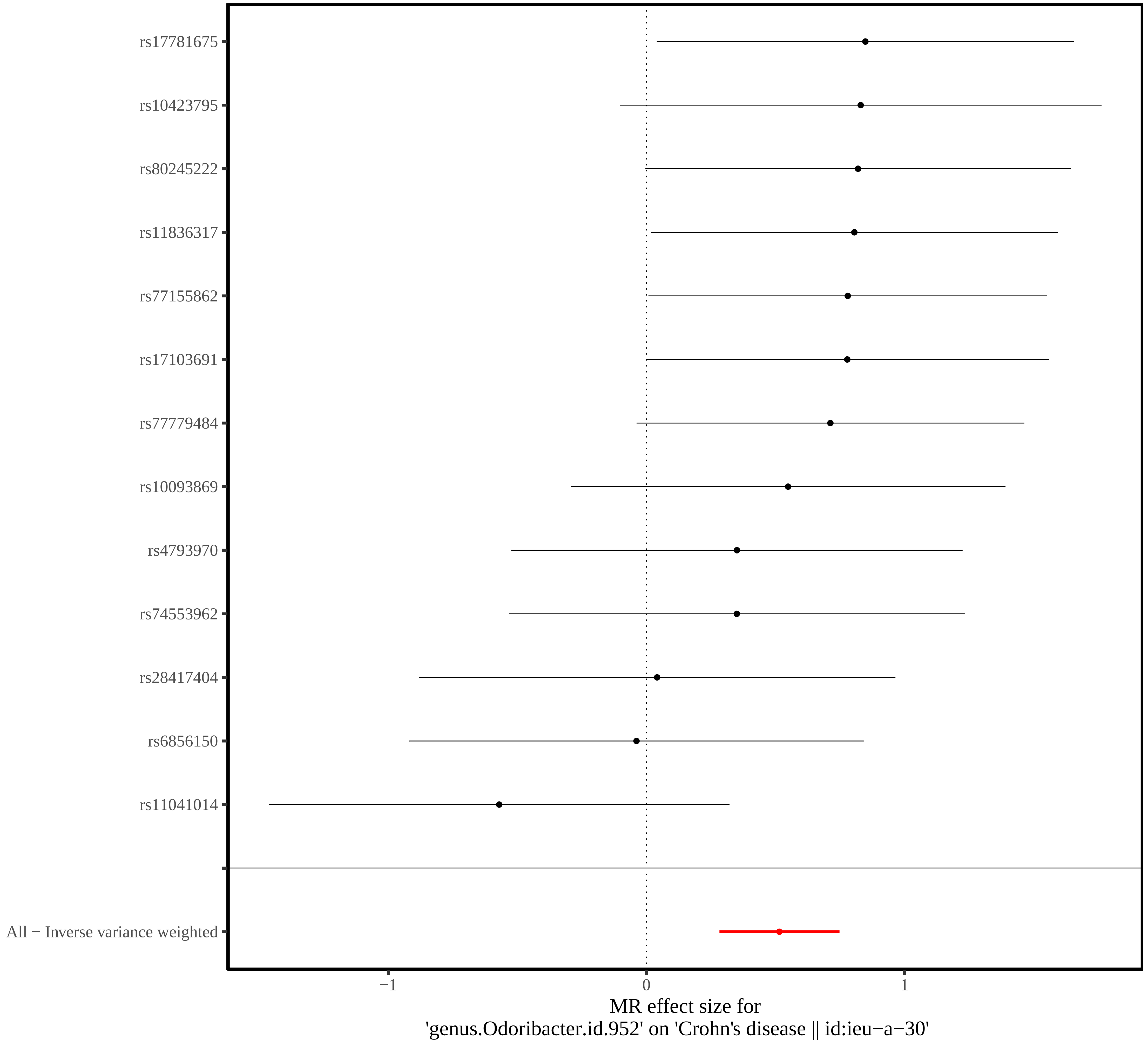

rs17781675
rs10423795
rs80245222
rs11836317
rs77155862
rs17103691
rs77779484
rs10093869
rs4793970
rs74553962
rs28417404
rs6856150
rs11041014
All − Inverse variance weighted
−1
0
1
MR effect size for
'genus.Odoribacter.id.952' on 'Crohn's disease || id:ieu−a−30'

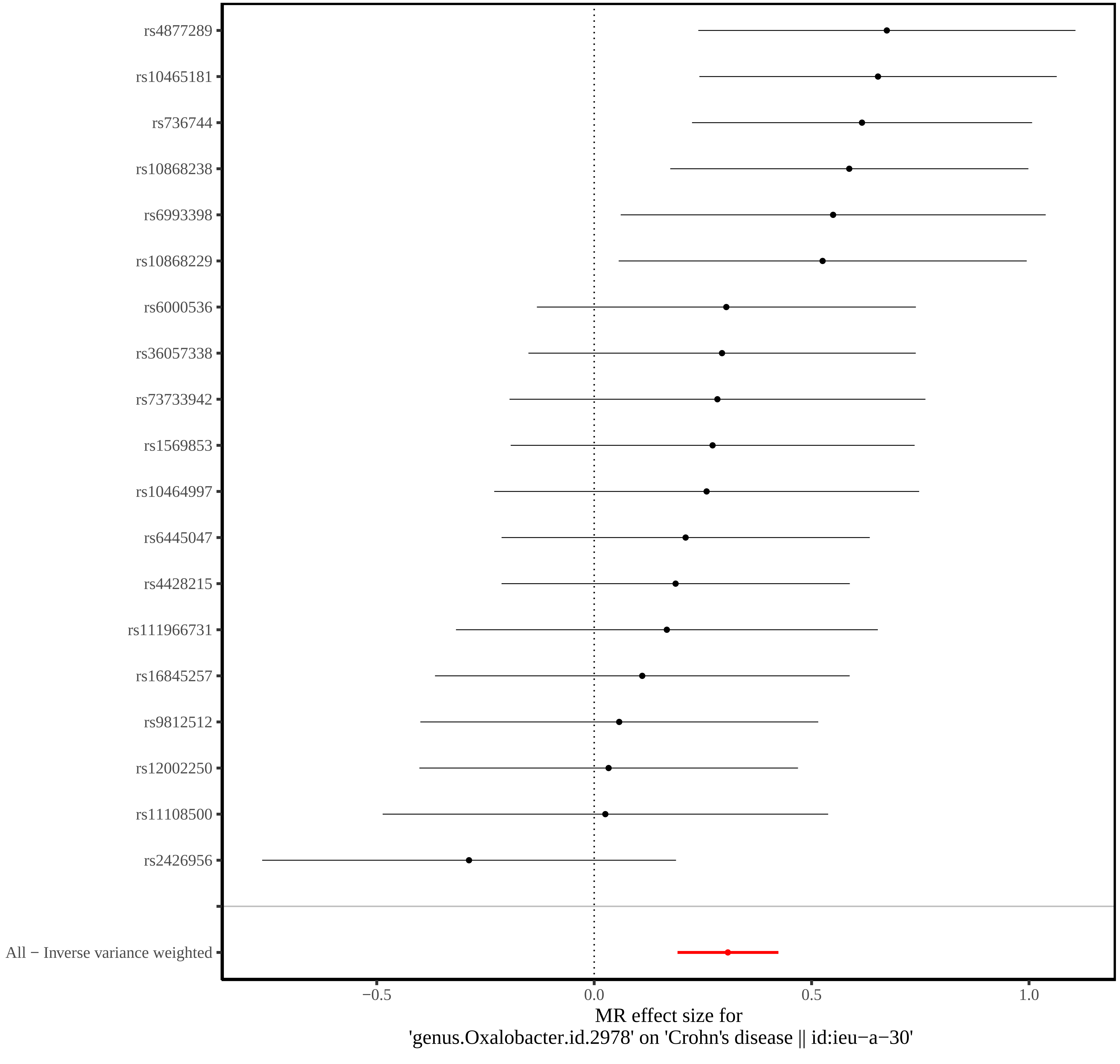
rs4877289
rs10465181
rs736744
rs10868238
rs6993398
rs10868229
rs6000536
rs36057338
rs73733942
rs1569853
rs10464997
rs6445047
rs4428215
rs111966731
rs16845257
rs9812512
rs12002250
rs11108500
rs2426956
All − Inverse variance weighted
−0.5
0.0
0.5
1.0
MR effect size for
'genus.Oxalobacter.id.2978' on 'Crohn's disease || id:ieu−a−30'

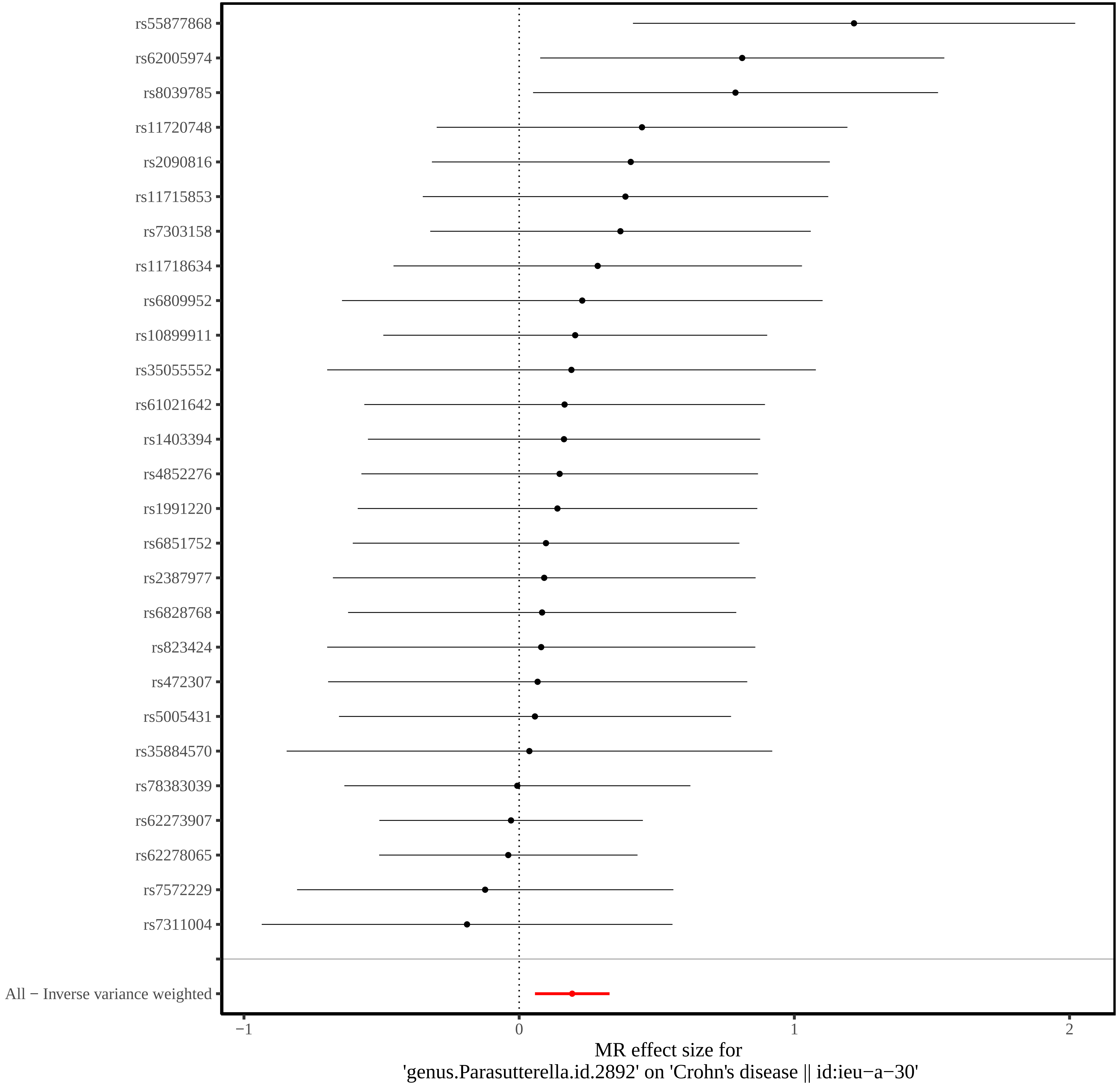

rs55877868
rs62005974
rs8039785
rs11720748
rs2090816
rs11715853
rs7303158
rs11718634
rs6809952
rs10899911
rs35055552
rs61021642
rs1403394
rs4852276
rs1991220
rs6851752
rs2387977
rs6828768
rs823424
rs472307
rs5005431
rs35884570
rs78383039
rs62273907
rs62278065
rs7572229
rs7311004
All − Inverse variance weighted
−1
0
1
2
MR effect size for
'genus.Parasutterella.id.2892' on 'Crohn's disease || id:ieu−a−30'

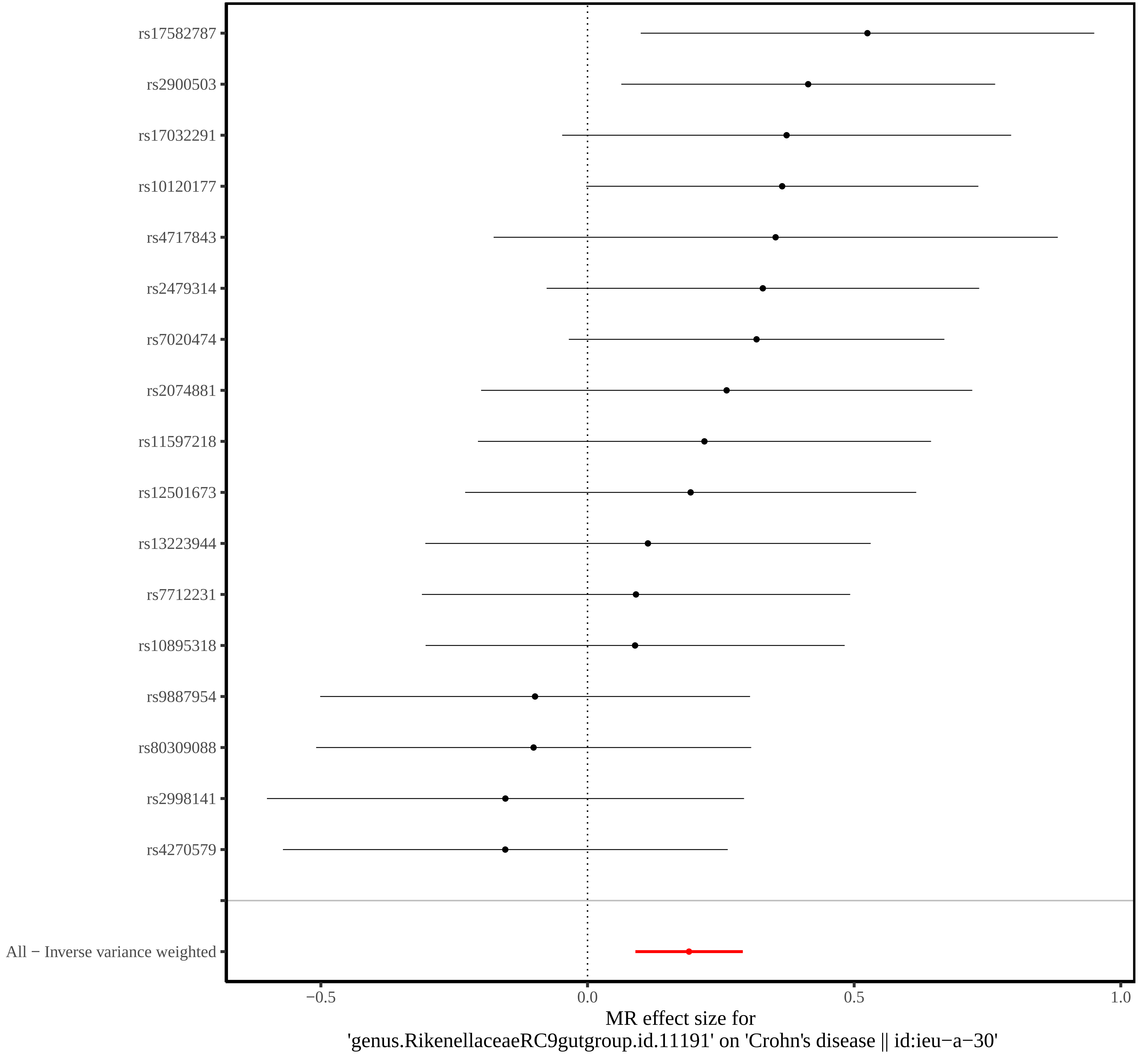
rs17582787
rs2900503
rs17032291
rs10120177
rs4717843
rs2479314
rs7020474
rs2074881
rs11597218
rs12501673
rs13223944
rs7712231
rs10895318
rs9887954
rs80309088
rs2998141
rs4270579
All − Inverse variance weighted
−0.5
0.0
0.5
1.0
MR effect size for
'genus.RikenellaceaeRC9gutgroup.id.11191' on 'Crohn's disease || id:ieu−a−30'

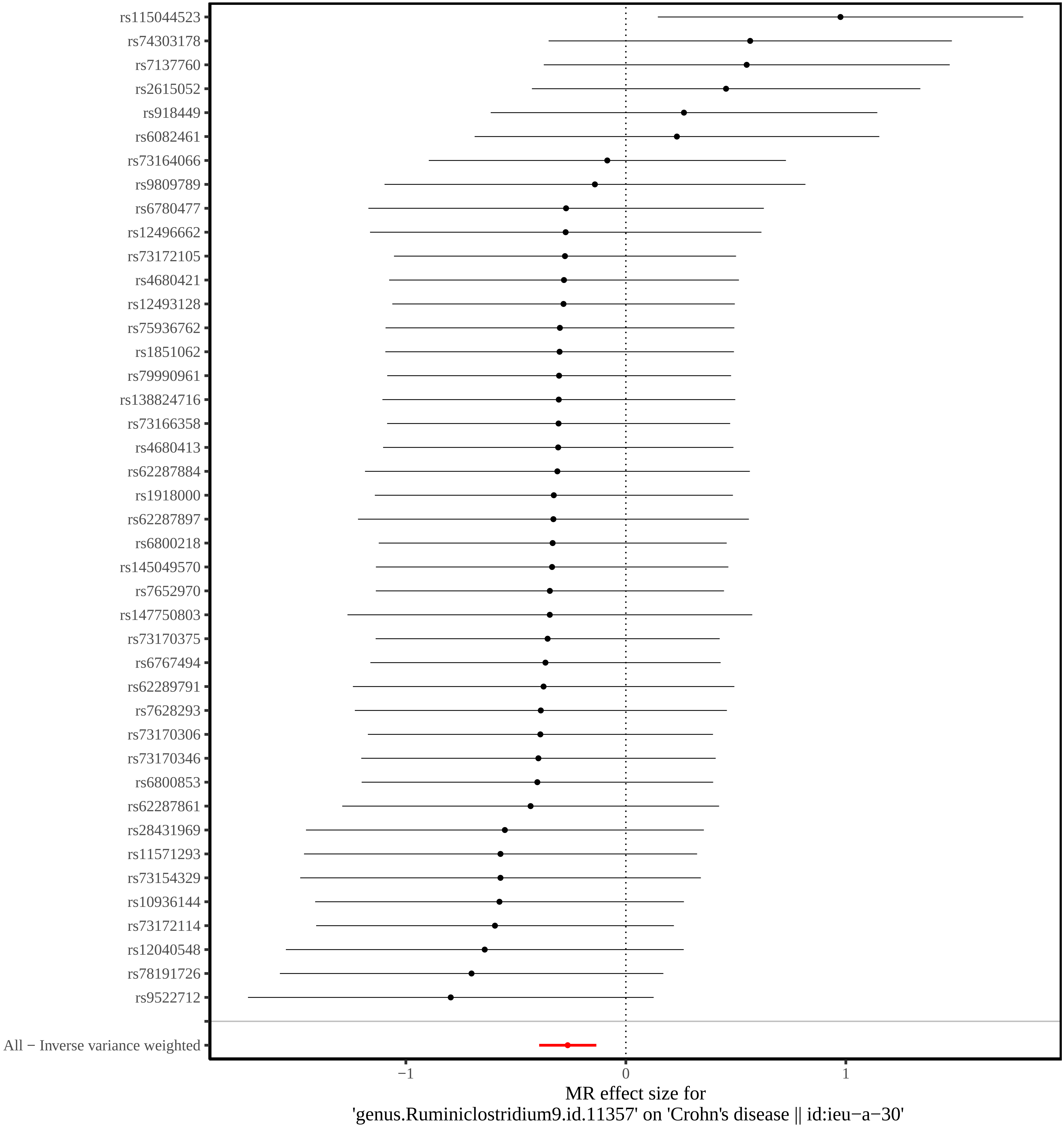
rs115044523
rs74303178
rs7137760
rs2615052
rs918449
rs6082461
rs73164066
rs9809789
rs6780477
rs12496662
rs73172105
rs4680421
rs12493128
rs75936762
rs1851062
rs79990961
rs138824716
rs73166358
rs4680413
rs62287884
rs1918000
rs62287897
rs6800218
rs145049570
rs7652970
rs147750803
rs73170375
rs6767494
rs62289791
rs7628293
rs73170306
rs73170346
rs6800853
rs62287861
rs28431969
rs11571293
rs73154329
rs10936144
rs73172114
rs12040548
rs78191726
rs9522712
All − Inverse variance weighted
−1
0
1
MR effect size for
'genus.Ruminiclostridium9.id.11357' on 'Crohn's disease || id:ieu−a−30'

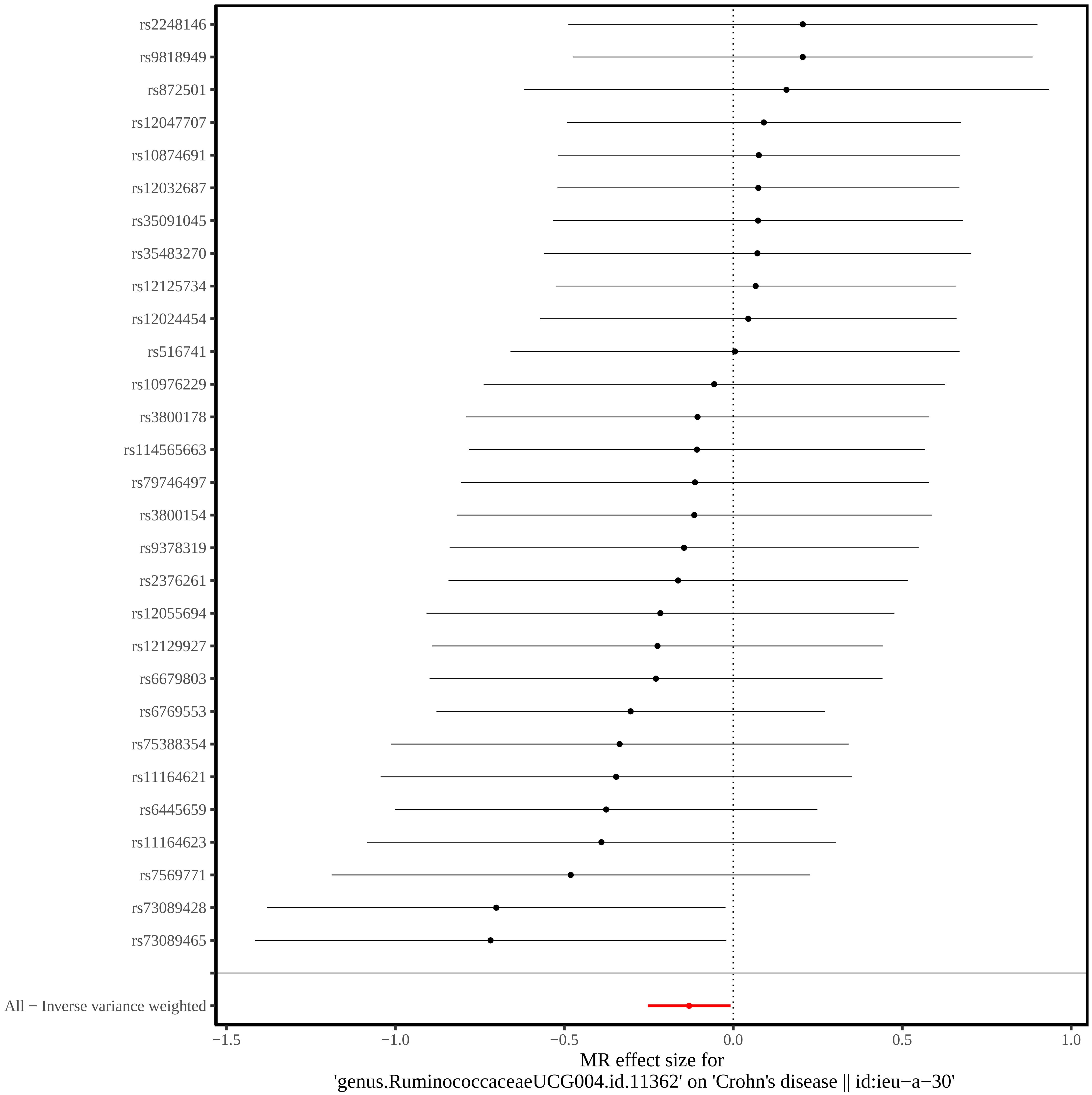

rs2248146
rs9818949
rs872501
rs12047707
rs10874691
rs12032687
rs35091045
rs35483270
rs12125734
rs12024454
rs516741
rs10976229
rs3800178
rs114565663
rs79746497
rs3800154
rs9378319
rs2376261
rs12055694
rs12129927
rs6679803
rs6769553
rs75388354
rs11164621
rs6445659
rs11164623
rs7569771
rs73089428
rs73089465
All − Inverse variance weighted
−1.5
−1.0
−0.5
0.0
0.5
1.0
MR effect size for
'genus.RuminococcaceaeUCG004.id.11362' on 'Crohn's disease || id:ieu−a−30'

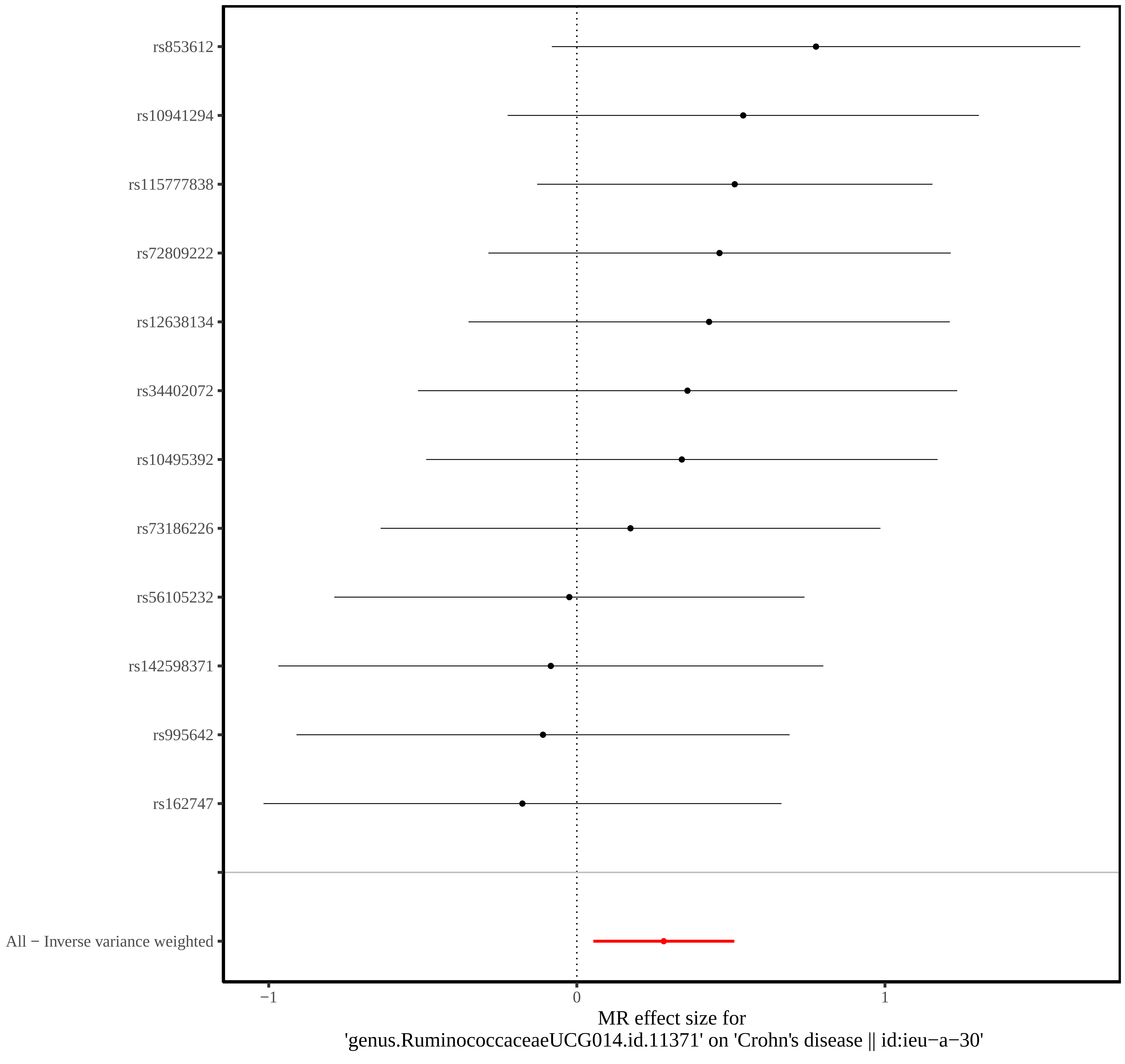
rs853612
rs10941294
rs115777838
rs72809222
rs12638134
rs34402072
rs10495392
rs73186226
rs56105232
rs142598371
rs995642
rs162747
All − Inverse variance weighted
−1
0
1
MR effect size for
'genus.RuminococcaceaeUCG014.id.11371' on 'Crohn's disease || id:ieu−a−30'

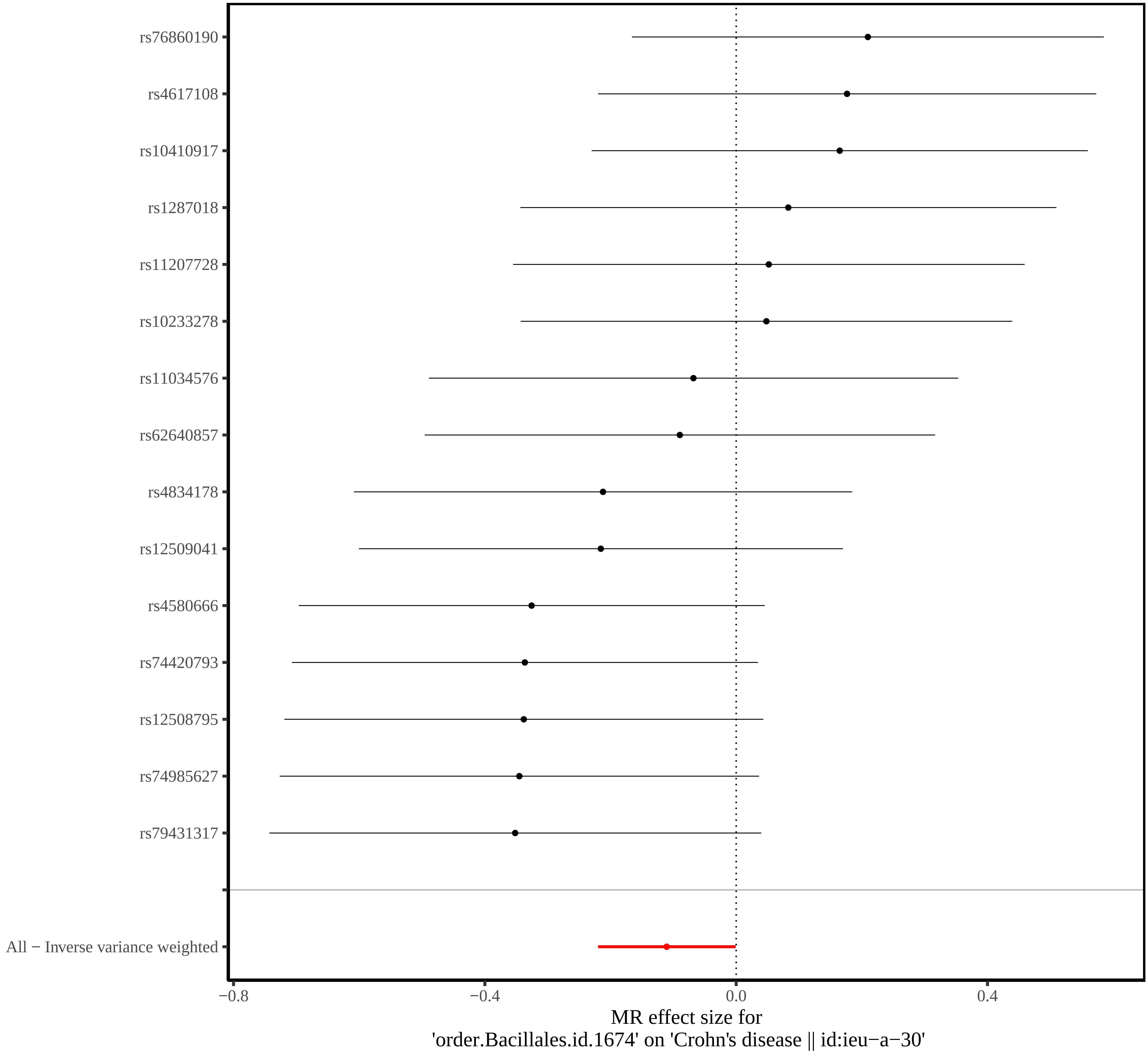

rs76860190
rs4617108
rs10410917
rs1287018
rs11207728
rs10233278
rs11034576
rs62640857
rs4834178
rs12509041
rs4580666
rs74420793
rs12508795
rs74985627
rs79431317
All − Inverse variance weighted
−0.8
−0.4
0.0
0.4
MR effect size for
'order.Bacillales.id.1674' on 'Crohn's disease || id:ieu−a−30'

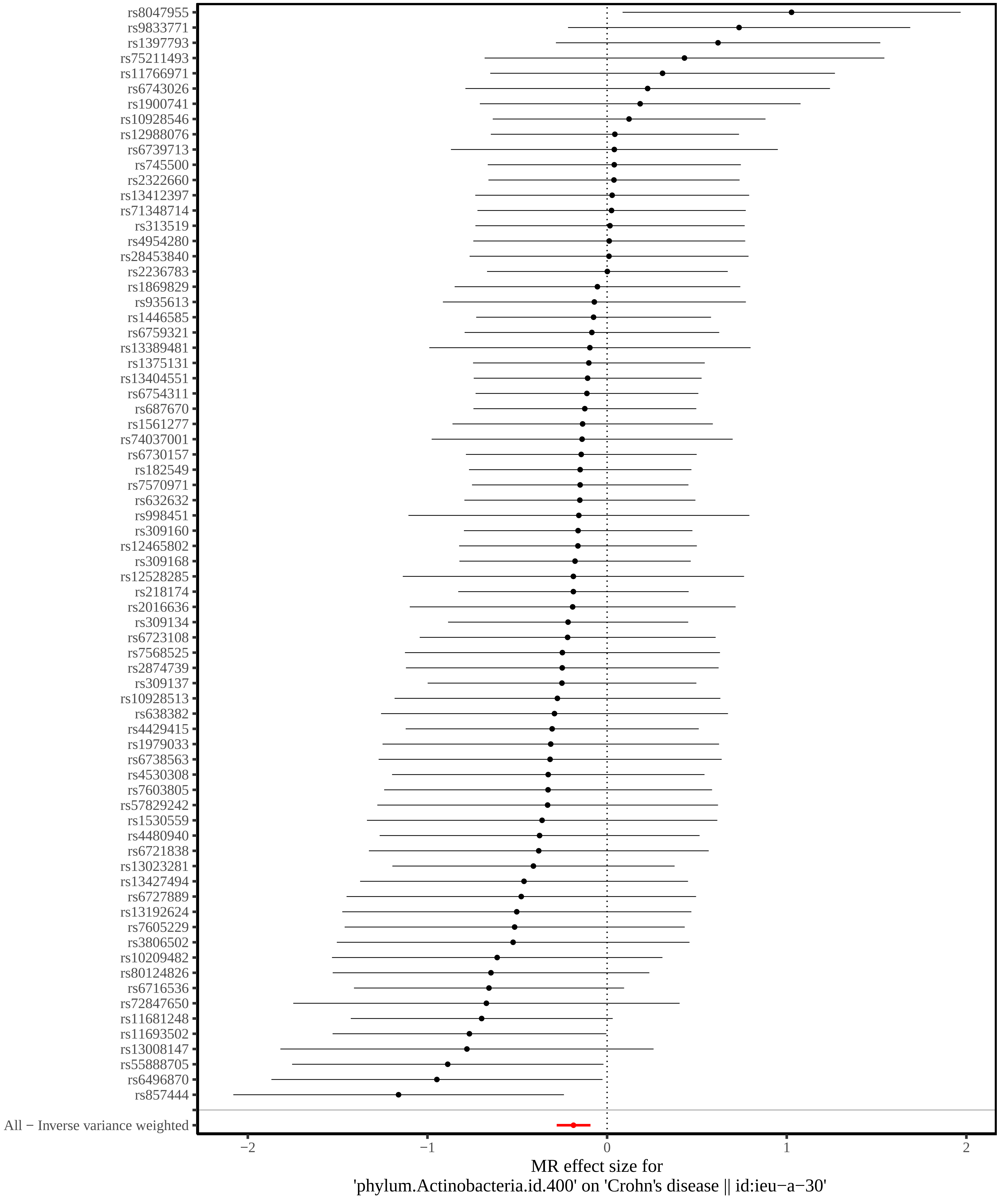
rs8047955
rs9833771
rs1397793
rs75211493
rs11766971
rs6743026
rs1900741
rs10928546
rs12988076
rs6739713
rs745500
rs2322660
rs13412397
rs71348714
rs313519
rs4954280
rs28453840
rs2236783
rs1869829
rs935613
rs1446585
rs6759321
rs13389481
rs1375131
rs13404551
rs6754311
rs687670
rs1561277
rs74037001
rs6730157
rs182549
rs7570971
rs632632
rs998451
rs309160
rs12465802
rs309168
rs12528285
rs218174
rs2016636
rs309134
rs6723108
rs7568525
rs2874739
rs309137
rs10928513
rs638382
rs4429415
rs1979033
rs6738563
rs4530308
rs7603805
rs57829242
rs1530559
rs4480940
rs6721838
rs13023281
rs13427494
rs6727889
rs13192624
rs7605229
rs3806502
rs10209482
rs80124826
rs6716536
rs72847650
rs11681248
rs11693502
rs13008147
rs55888705
rs6496870
rs857444
All − Inverse variance weighted
−2
−1
0
1
2
MR effect size for
'phylum.Actinobacteria.id.400' on 'Crohn's disease || id:ieu−a−30'

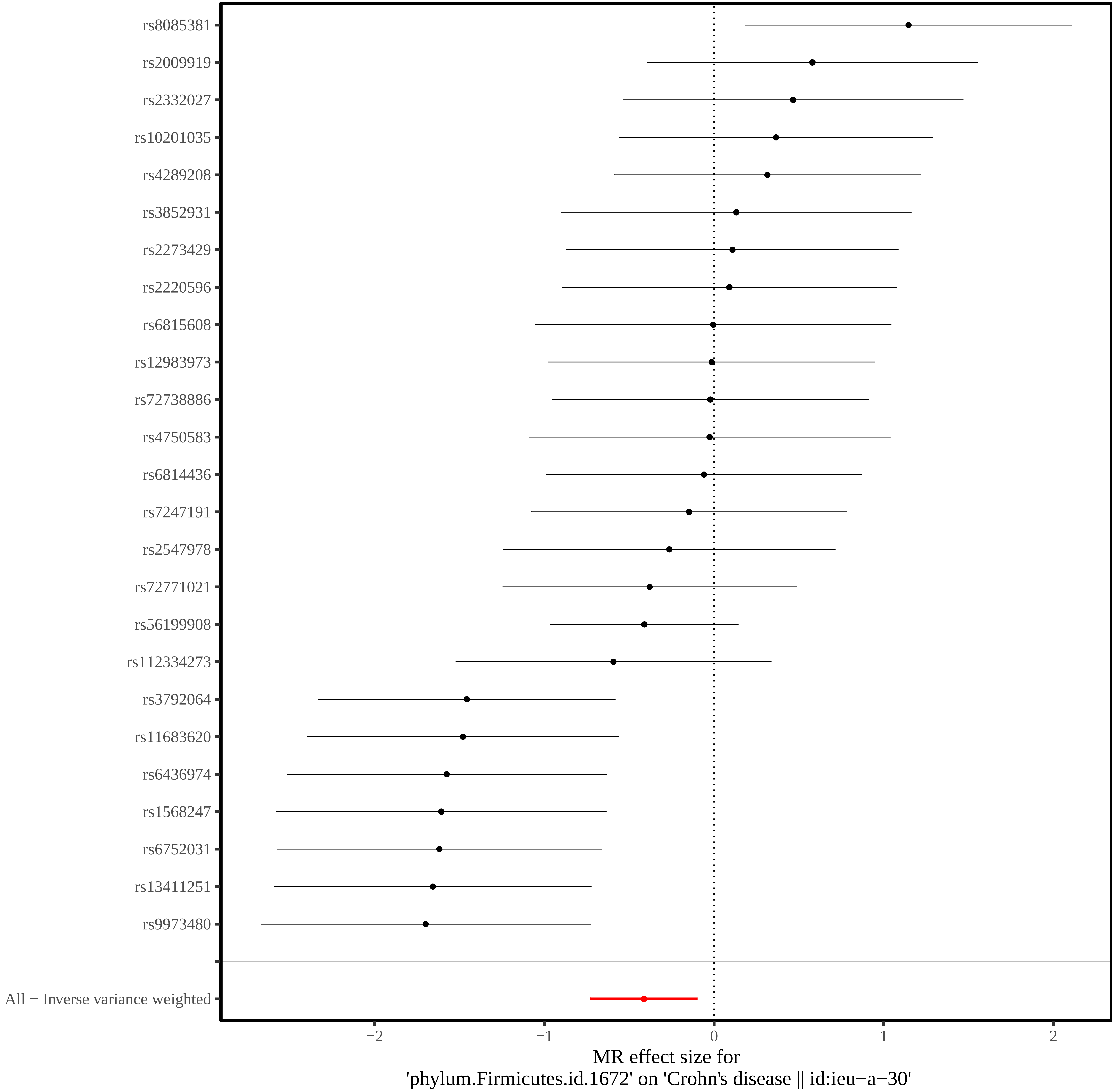
rs8085381
rs2009919
rs2332027
rs10201035
rs4289208
rs3852931
rs2273429
rs2220596
rs6815608
rs12983973
rs72738886
rs4750583
rs6814436
rs7247191
rs2547978
rs72771021
rs56199908
rs112334273
rs3792064
rs11683620
rs6436974
rs1568247
rs6752031
rs13411251
rs9973480
All − Inverse variance weighted
−2
−1
0
1
2
MR effect size for
'phylum.Firmicutes.id.1672' on 'Crohn's disease || id:ieu−a−30'
