# Supplementary material for: Mendelian randomization integrated with multi-omics analysis identifies TNIK as a key gene in gut microbiota-induced IBD development
Source: Front Immunol. 2025 Nov 18;16:1678444. doi: 10.3389/fimmu.2025.1678444 (PMC12669205; doi:10.3389/fimmu.2025.1678444)
Supplement: Supplementary file 2 [file DataSheet2.pdf]

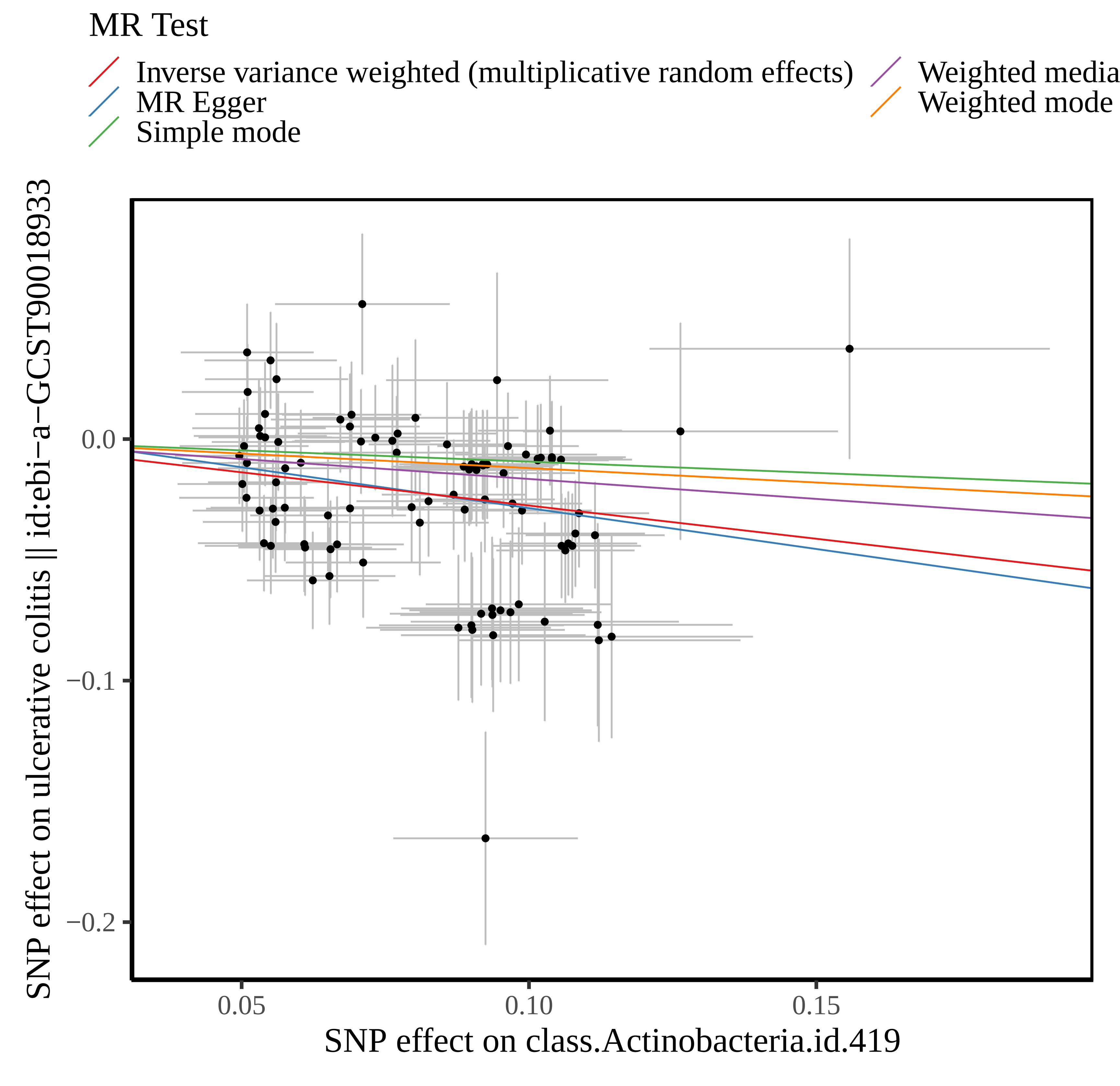

MR Test
Inverse variance weighted (multiplicative random effects)
MR Egger
Simple mode
Weighted media
Weighted mode
SNP effect on ulcerative colitis || id:ebi−a−GCST90018933
0.0
−0.1
−0.2
0.05
0.10
0.15
SNP effect on class.Actinobacteria.id.419

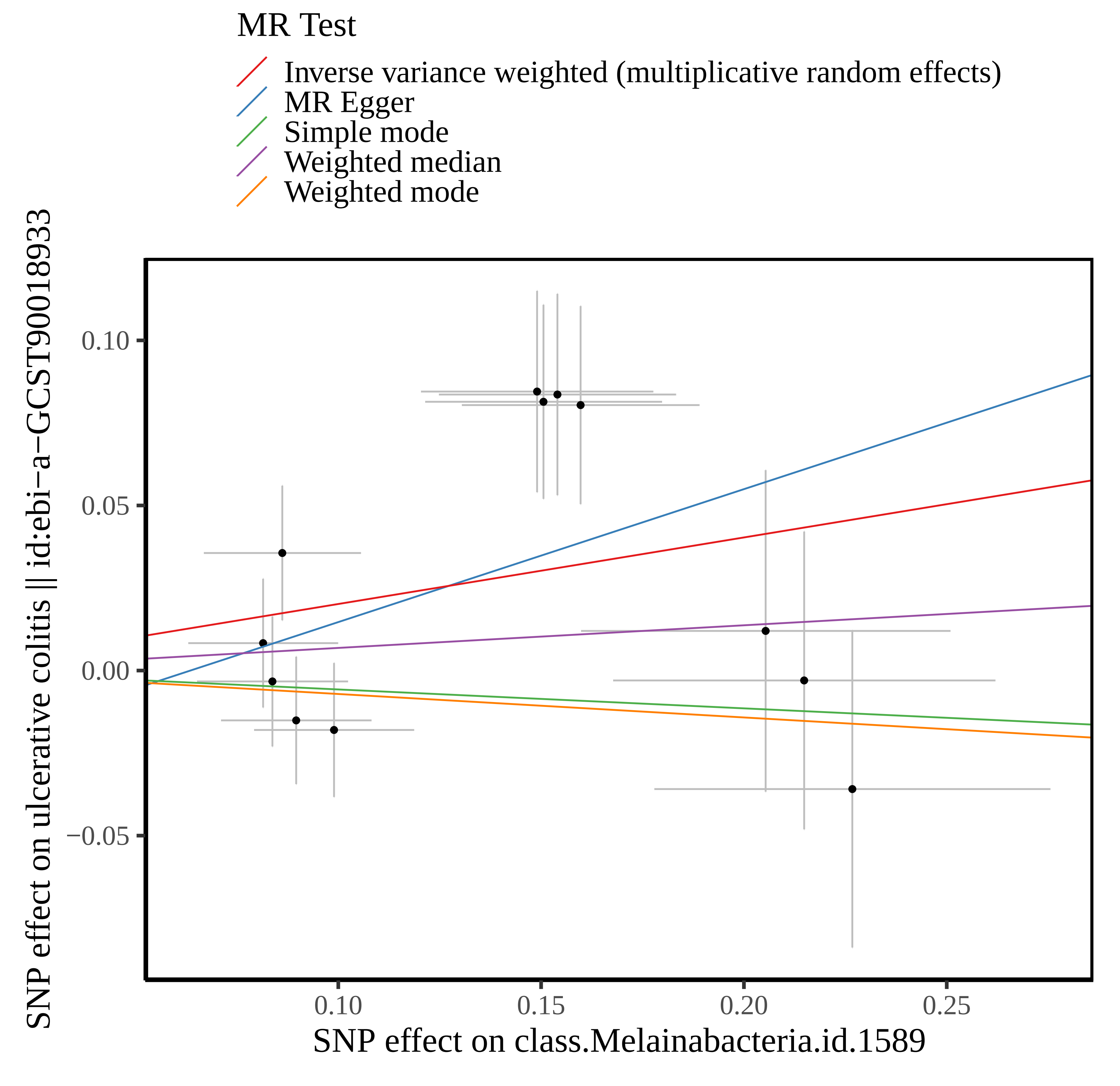

MR Test
Inverse variance weighted (multiplicative random effects)
MR Egger
Simple mode
Weighted median
Weighted mode
SNP effect on ulcerative colitis || id:ebi−a−GCST90018933
0.10
0.05
0.00
−0.05
0.10
0.15
0.20
0.25
SNP effect on class.Melainabacteria.id.1589

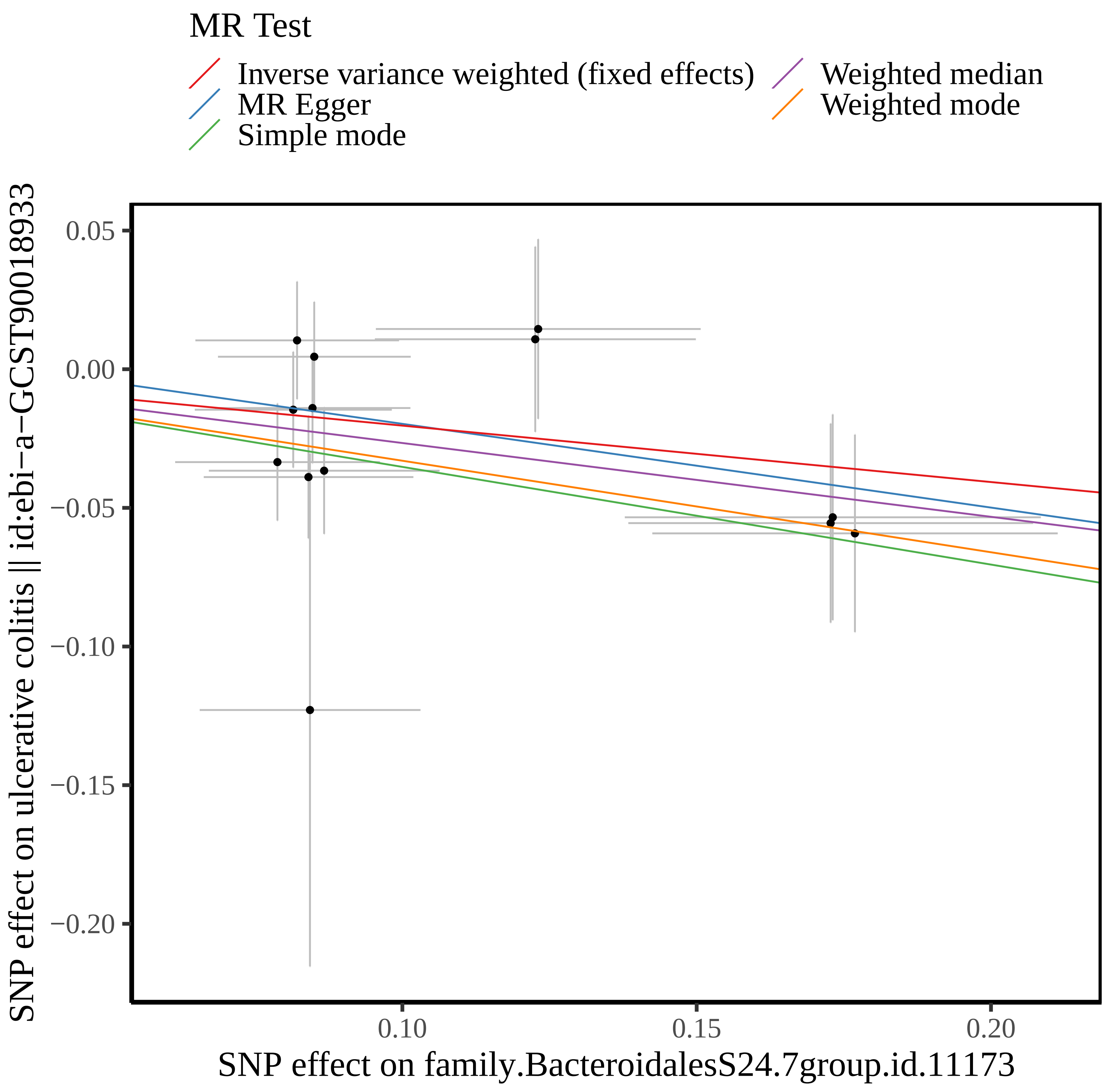

MR Test
Inverse variance weighted (fixed effects)
MR Egger
Simple mode
Weighted median
Weighted mode
0.05
0.00
−0.05
−0.10
−0.15
−0.20
0.10
0.15
0.20
SNP effect on ulcerative colitis || id:ebi−a−GCST90018933
SNP effect on family.BacteroidalesS24.7group.id.11173

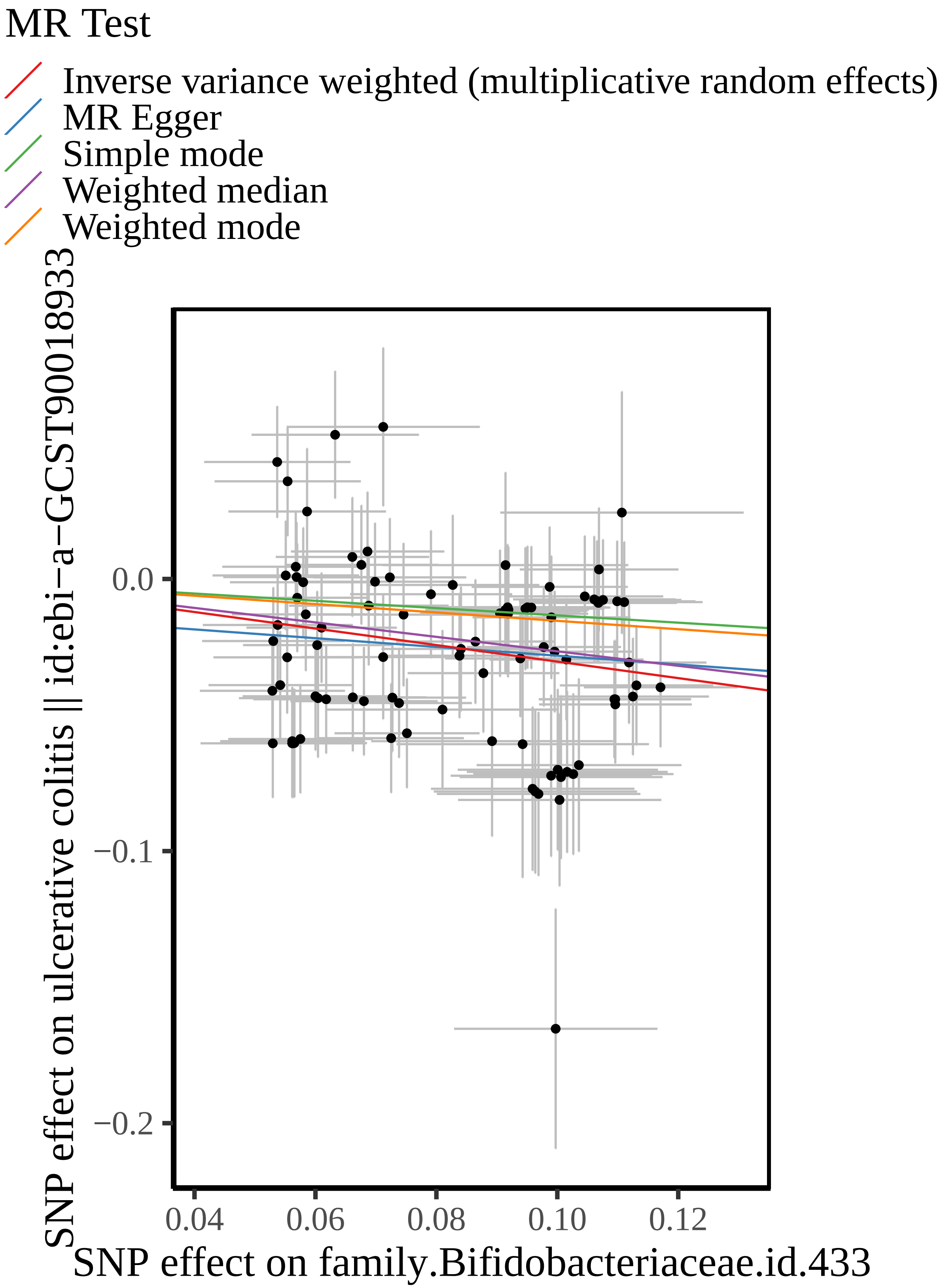

MR Test
Inverse variance weighted (multiplicative random effects)
MR Egger
Simple mode
Weighted median
Weighted mode
SNP effect on ulcerative colitis || id:ebi−a−GCST90018933
0.0
−0.1
−0.2
0.04
0.06
0.08
0.10
0.12
SNP effect on family.Bifidobacteriaceae.id.433

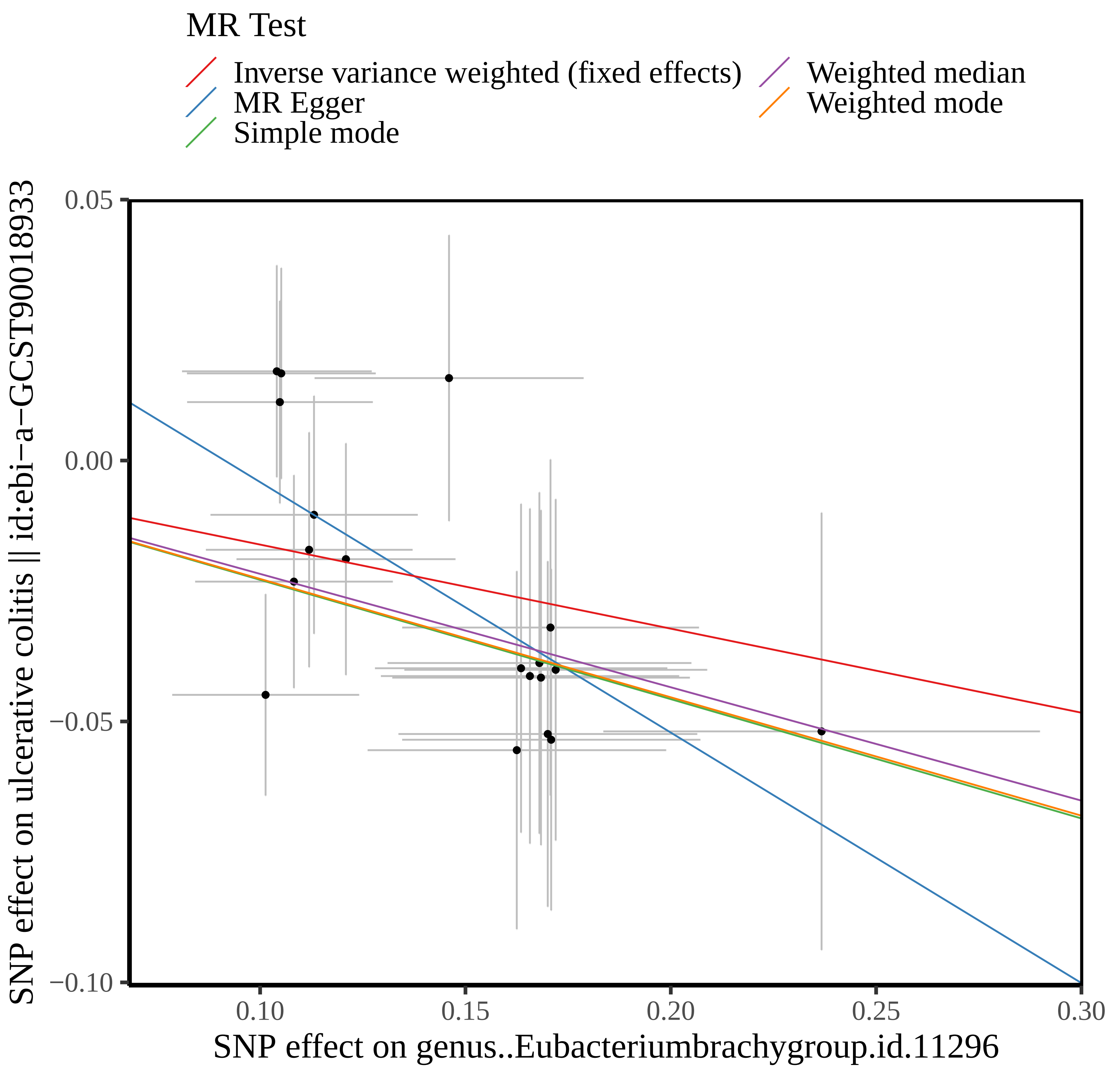

MR Test
Inverse variance weighted (fixed effects)
MR Egger
Simple mode
Weighted median
Weighted mode
SNP effect on ulcerative colitis || id:ebi−a−GCST90018933
0.05
0.00
−0.05
−0.10
0.10
0.15
0.20
0.25
0.30
SNP effect on genus..Eubacteriumbrachygroup.id.11296

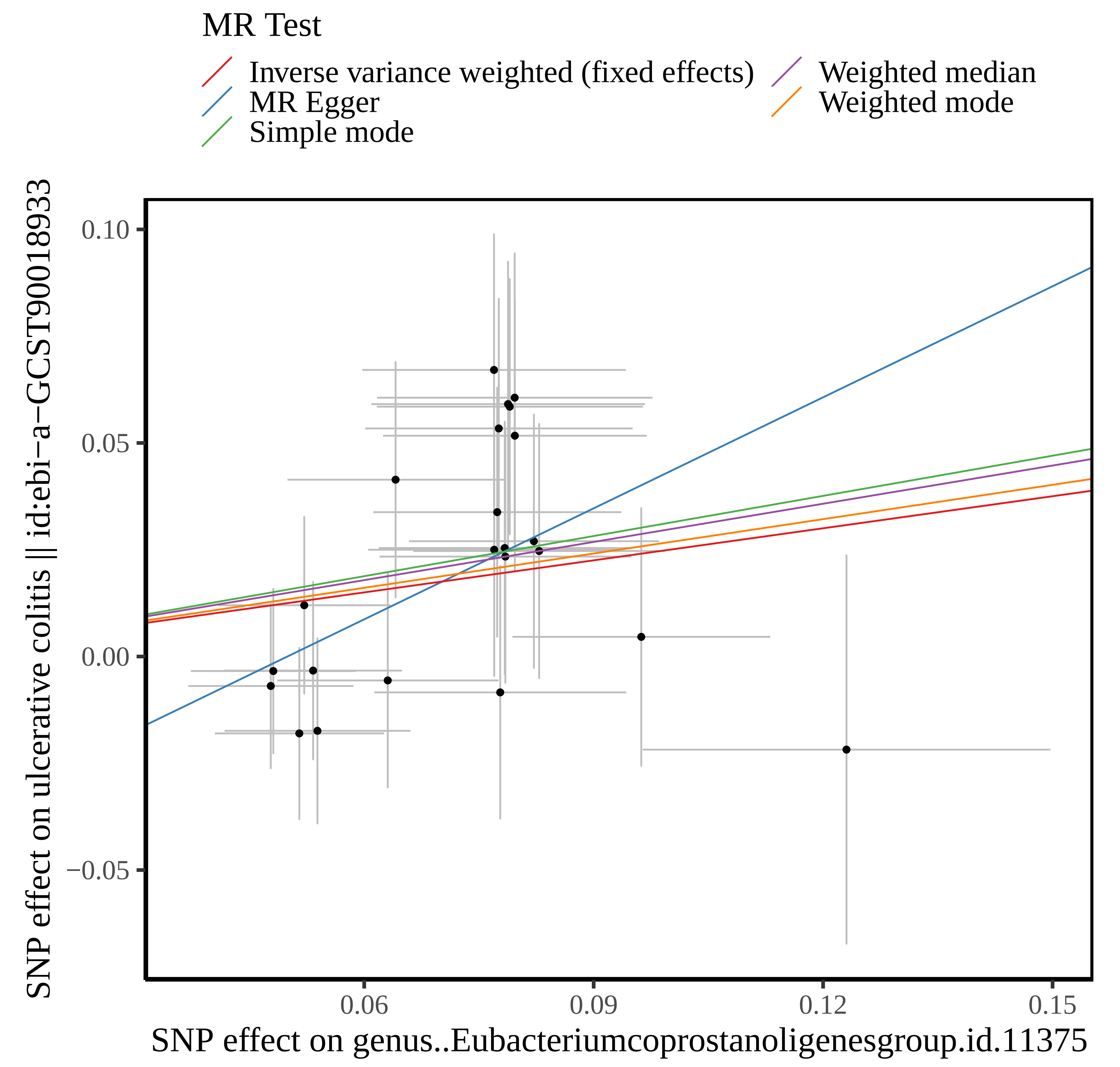

MR Test
Inverse variance weighted (fixed effects)
MR Egger
Simple mode
Weighted median
Weighted mode
0.10
0.05
0.00
−0.05
0.06
0.09
0.12
0.15
SNP effect on ulcerative colitis || id:ebi−a−GCST90018933
SNP effect on genus..Eubacteriumcoprostanoligenesgroup.id.11375

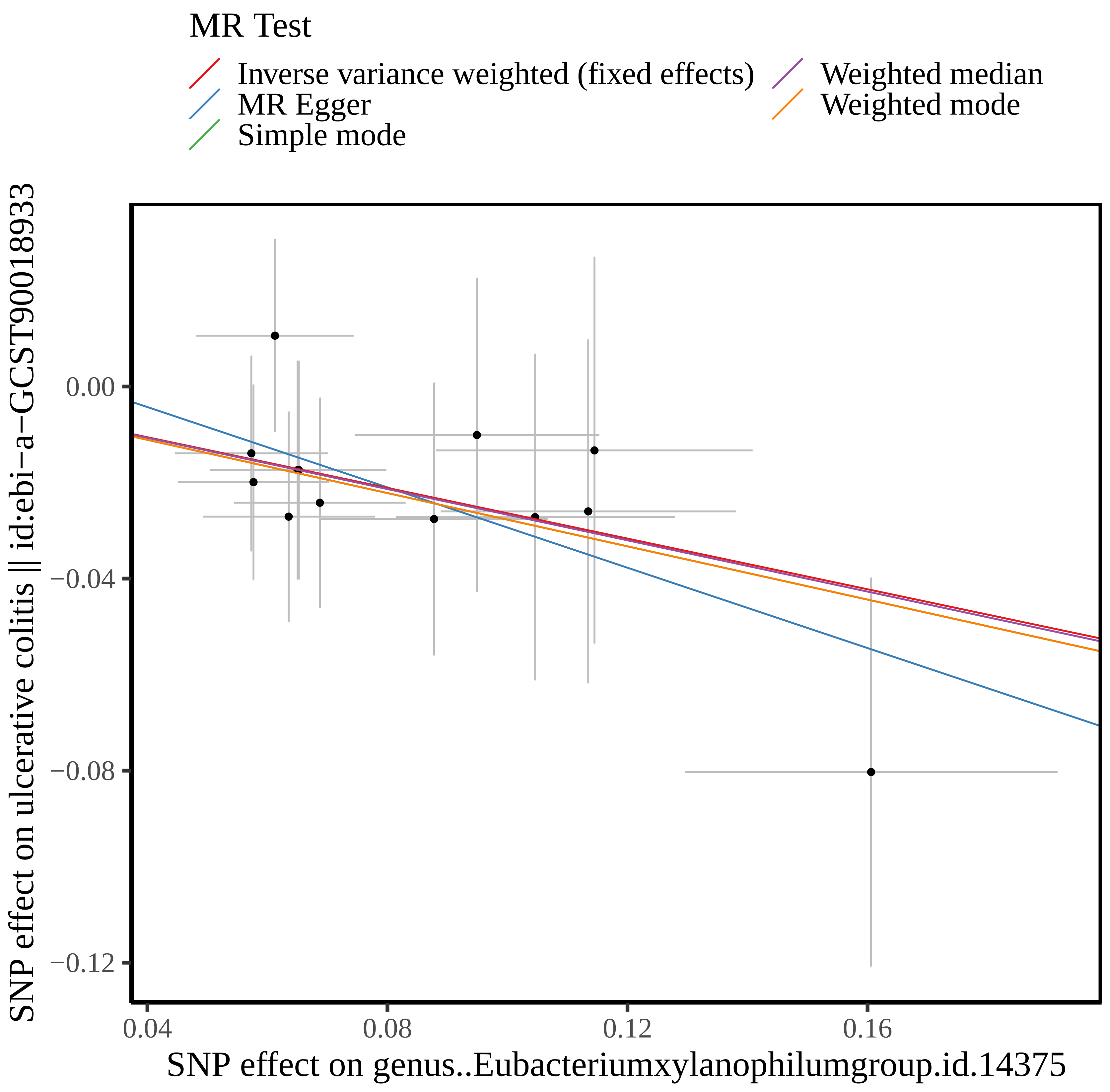
MR Test
Inverse variance weighted (fixed effects)
MR Egger
Simple mode
Weighted median
Weighted mode
SNP effect on ulcerative colitis || id:ebi−a−GCST90018933
0.00
−0.04
−0.08
−0.12
0.04
0.08
0.12
0.16
SNP effect on genus..Eubacteriumxylanophilumgroup.id.14375

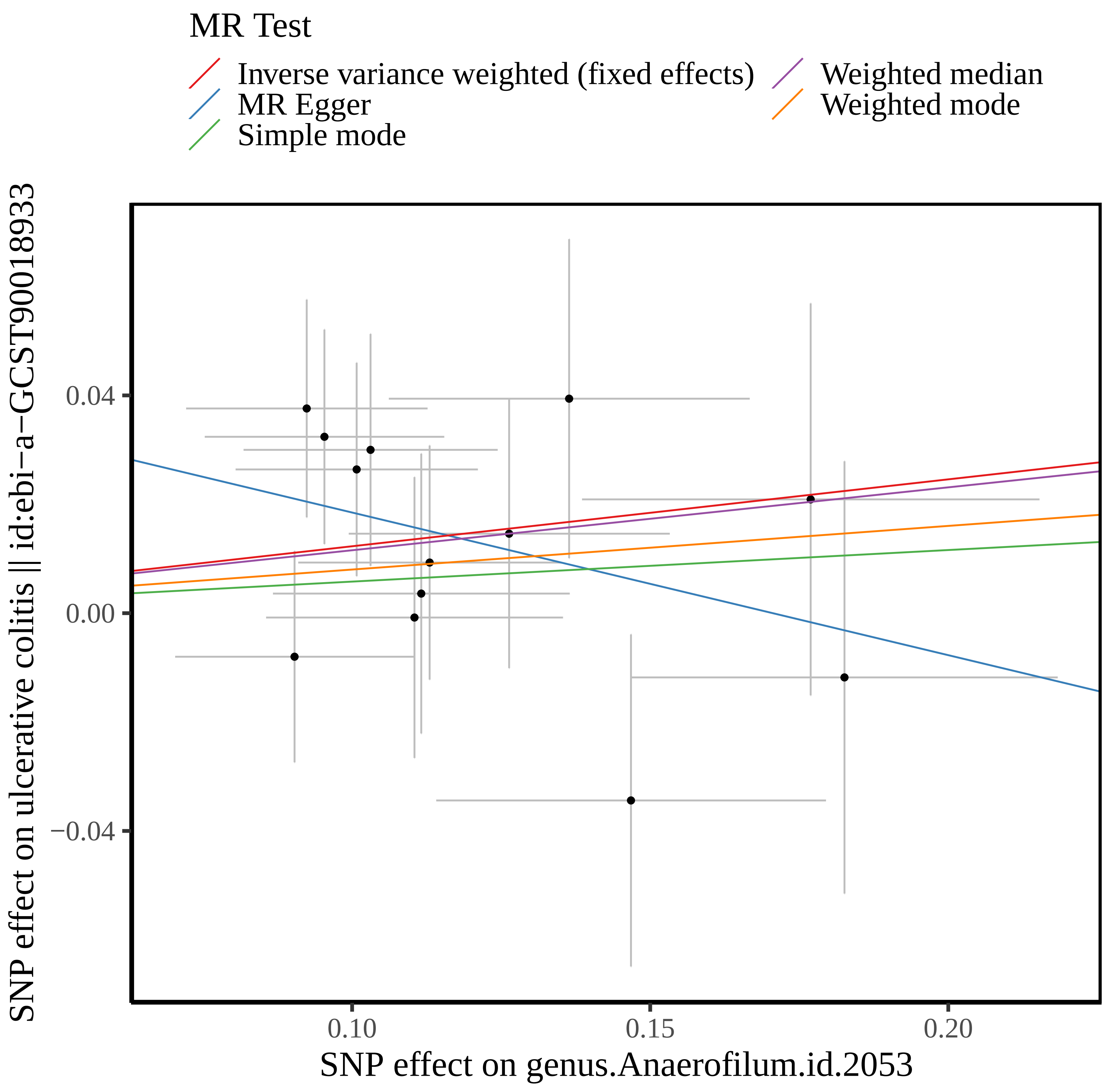

MR Test
Inverse variance weighted (fixed effects)
MR Egger
Simple mode
Weighted median
Weighted mode
SNP effect on ulcerative colitis || id:ebi−a−GCST90018933
0.04
0.00
−0.04
0.10
0.15
0.20
SNP effect on genus.Anaerofilum.id.2053

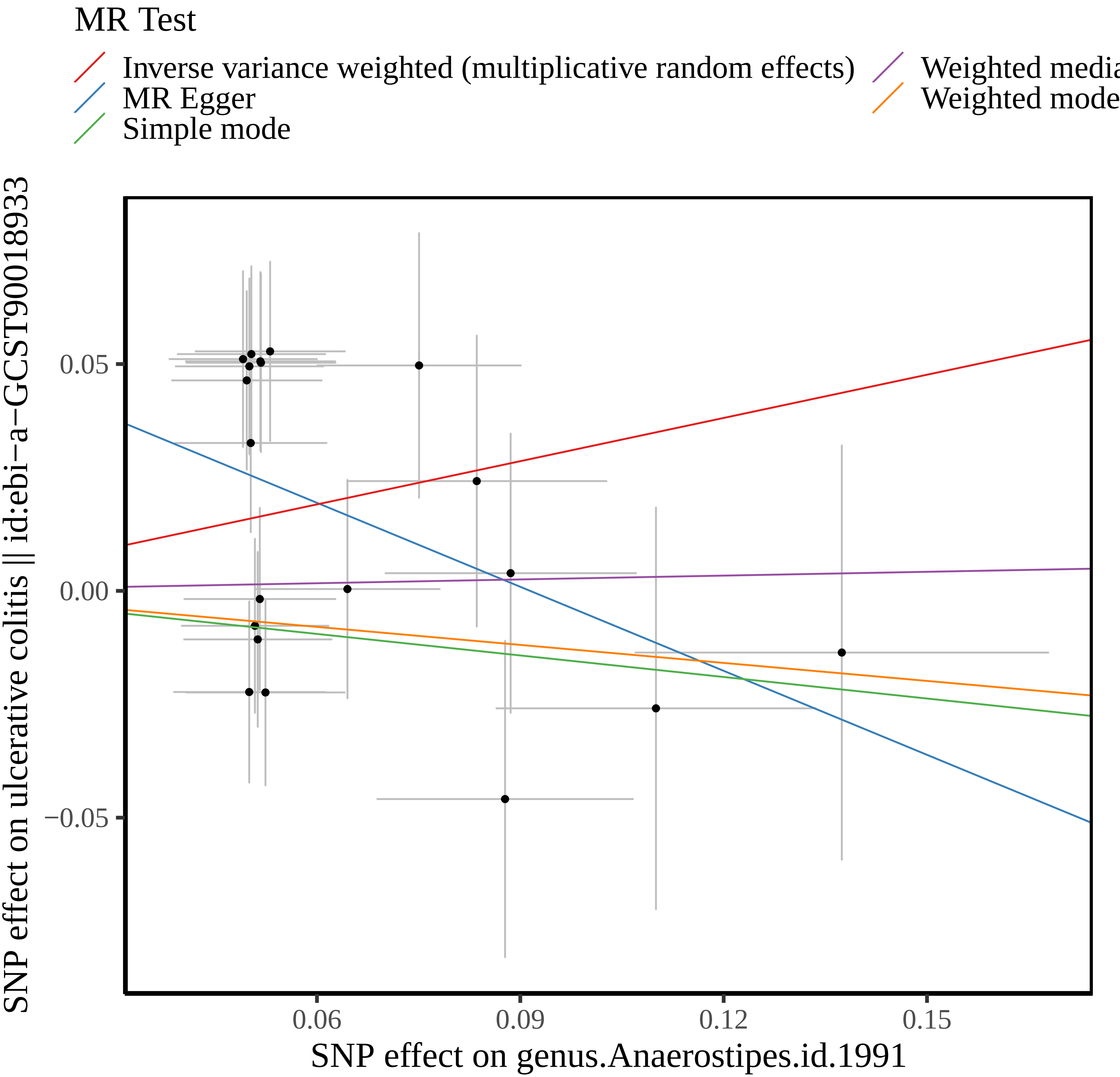
MR Test
Inverse variance weighted (multiplicative random effects)
MR Egger
Simple mode
Weighted media
Weighted mode
SNP effect on ulcerative colitis || id:ebi−a−GCST90018933
0.05
0.00
−0.05
0.06
0.09
0.12
0.15
SNP effect on genus.Anaerostipes.id.1991

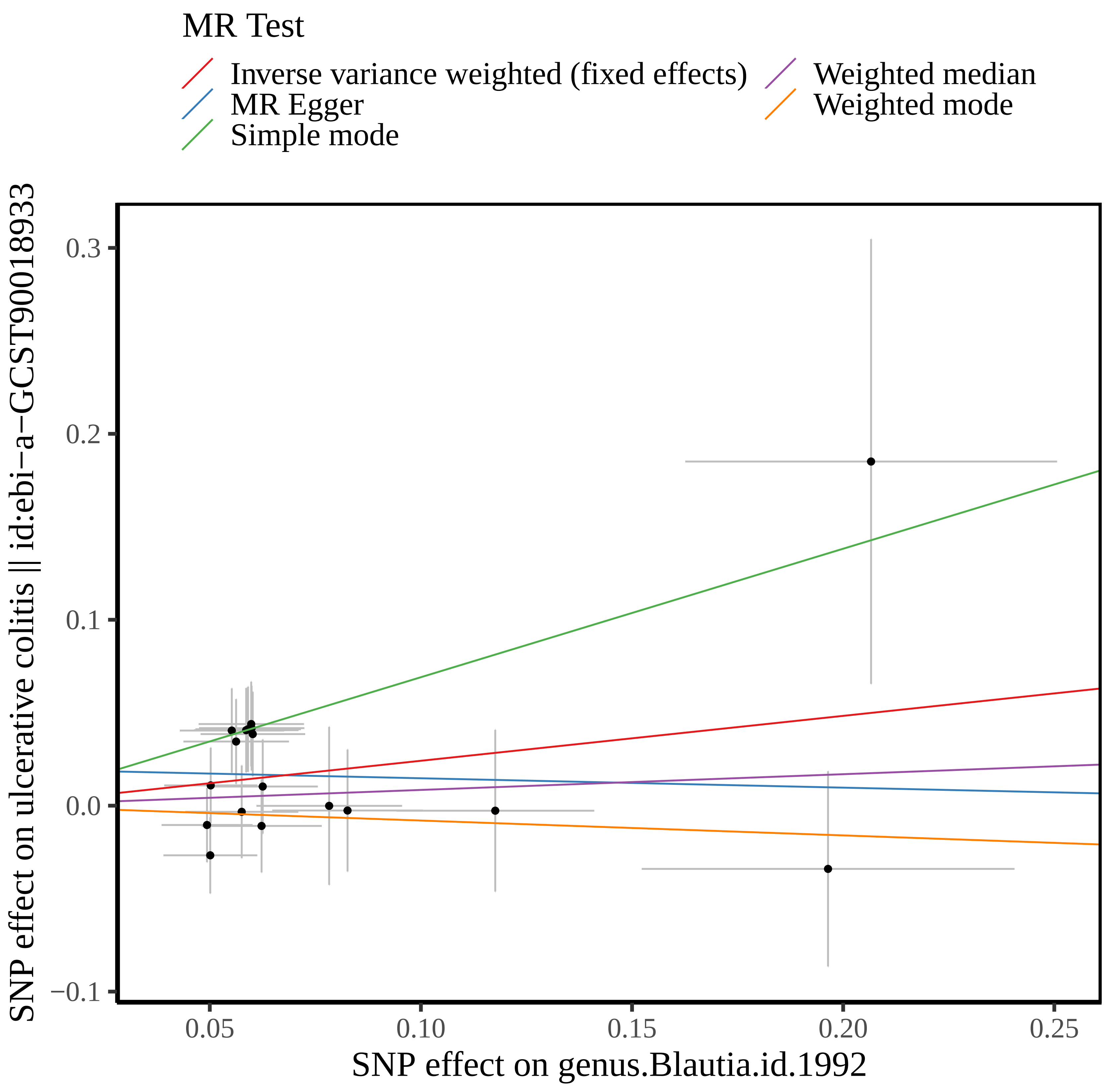
MR Test
Inverse variance weighted (fixed effects)
MR Egger
Simple mode
Weighted median
Weighted mode
SNP effect on ulcerative colitis || id:ebi−a−GCST90018933
0.3
0.2
0.1
0.0
−0.1
0.05
0.10
0.15
0.20
0.25
SNP effect on genus.Blautia.id.1992

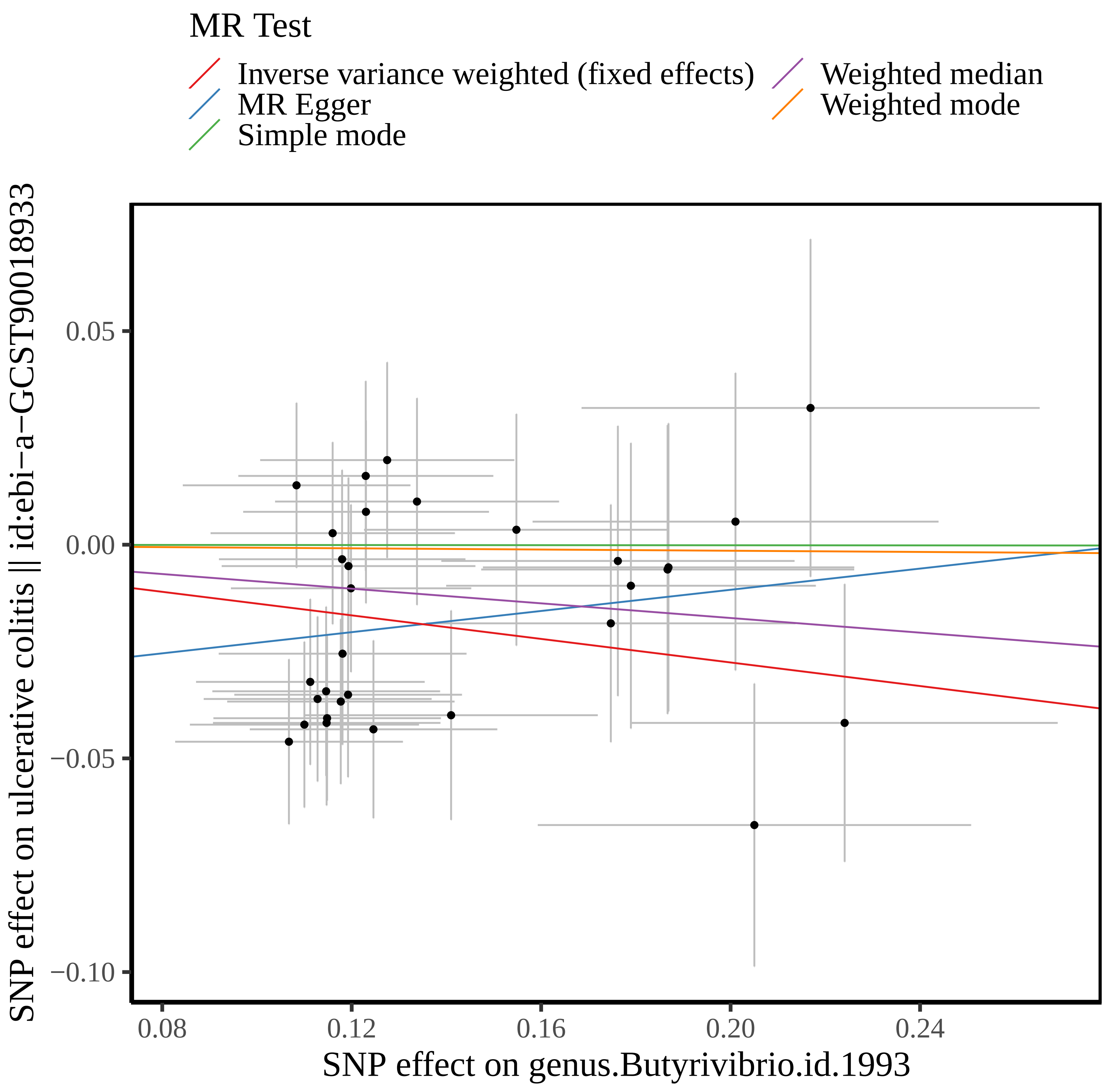

MR Test
Inverse variance weighted (fixed effects)
MR Egger
Simple mode
Weighted median
Weighted mode
SNP effect on ulcerative colitis || id:ebi−a−GCST90018933
0.05
0.00
−0.05
−0.10
0.08
0.12
0.16
0.20
0.24
SNP effect on genus.Butyrivibrio.id.1993

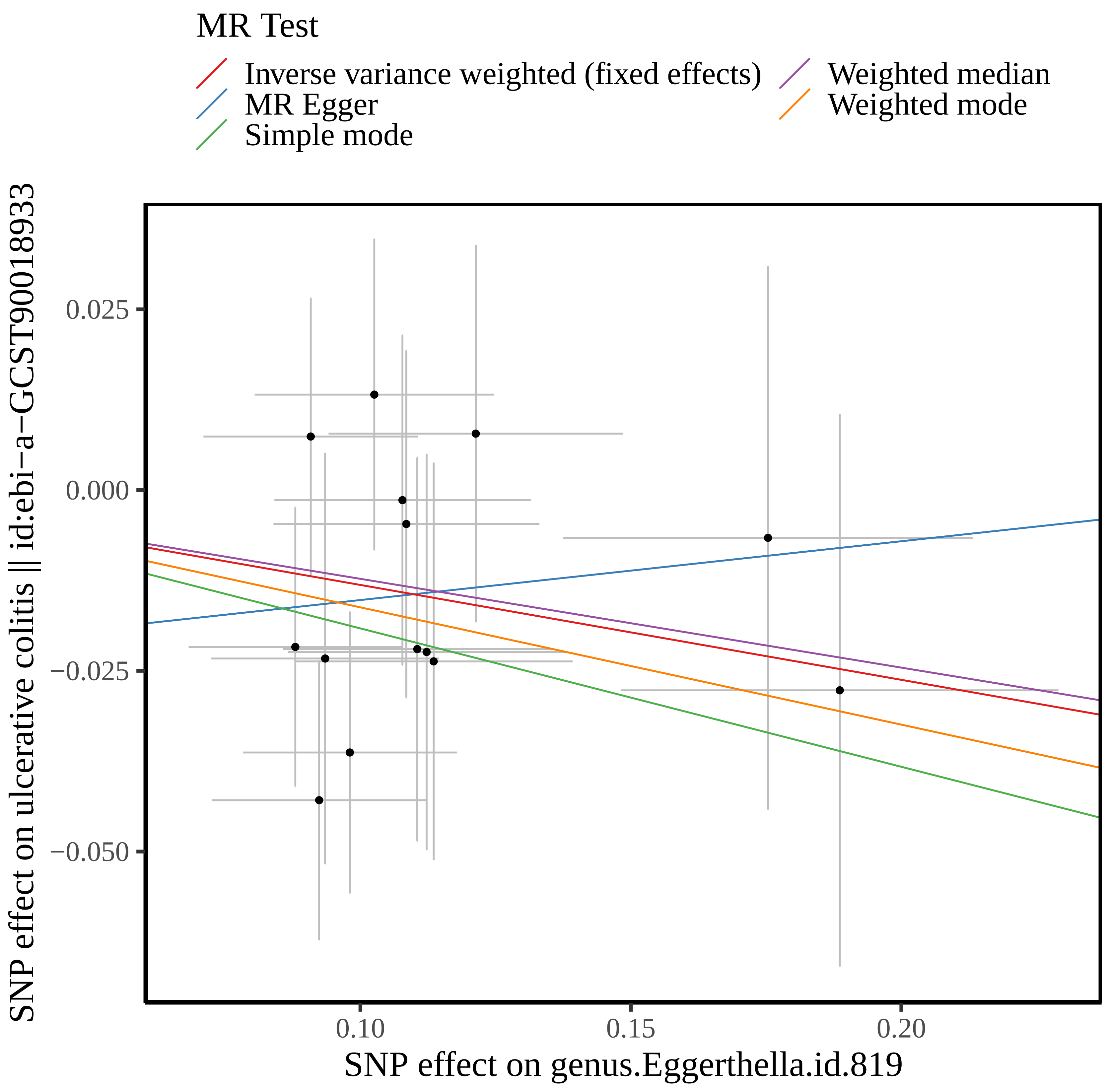

MR Test
Inverse variance weighted (fixed effects)
MR Egger
Simple mode
Weighted median
Weighted mode
SNP effect on ulcerative colitis || id:ebi−a−GCST90018933
0.025
0.000
−0.025
−0.050
0.10
0.15
0.20
SNP effect on genus.Eggerthella.id.819

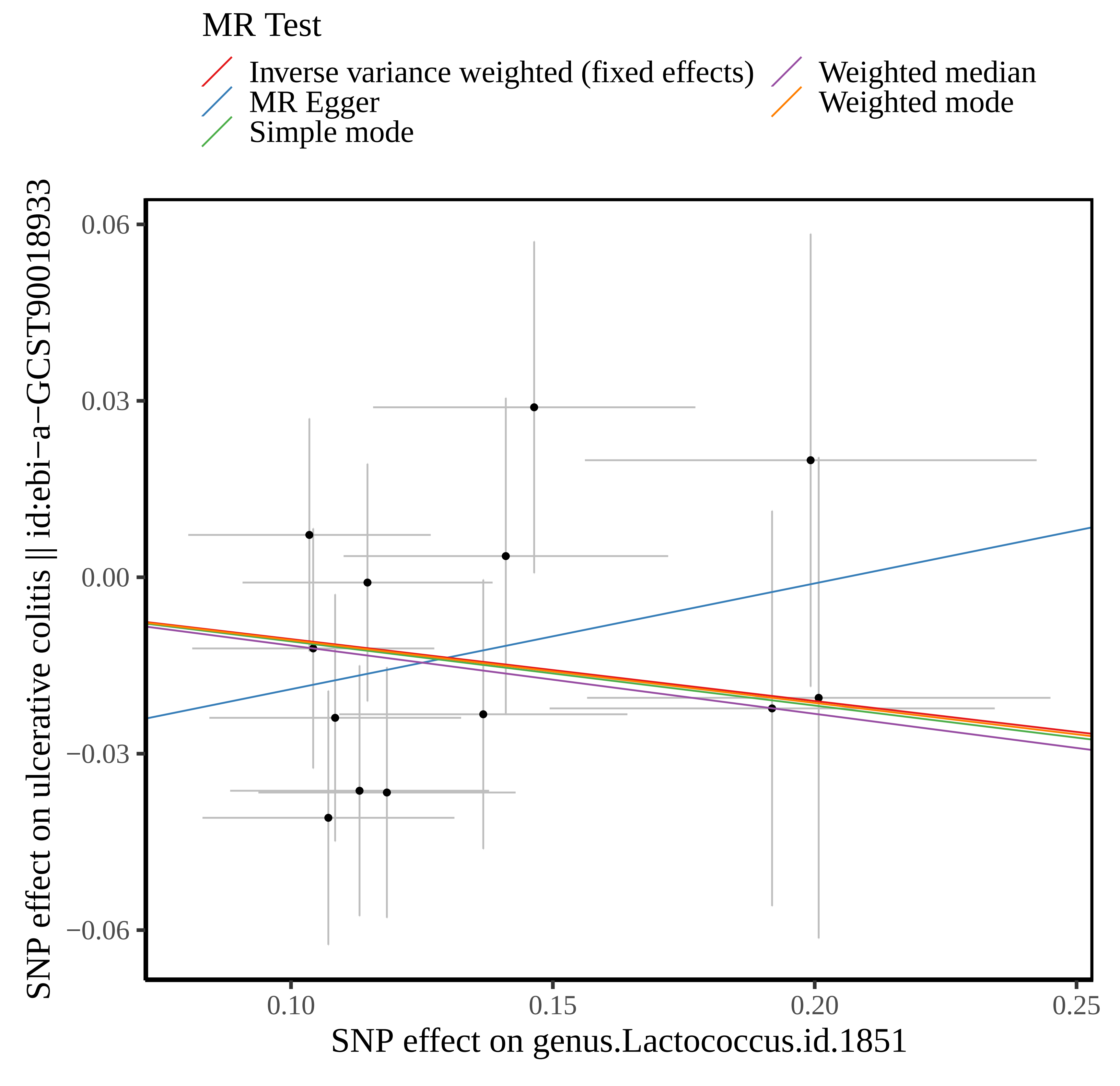
MR Test
Inverse variance weighted (fixed effects)
MR Egger
Simple mode
Weighted median
Weighted mode
SNP effect on ulcerative colitis || id:ebi−a−GCST90018933
0.06
0.03
0.00
−0.03
−0.06
0.10
0.15
0.20
0.25
SNP effect on genus.Lactococcus.id.1851

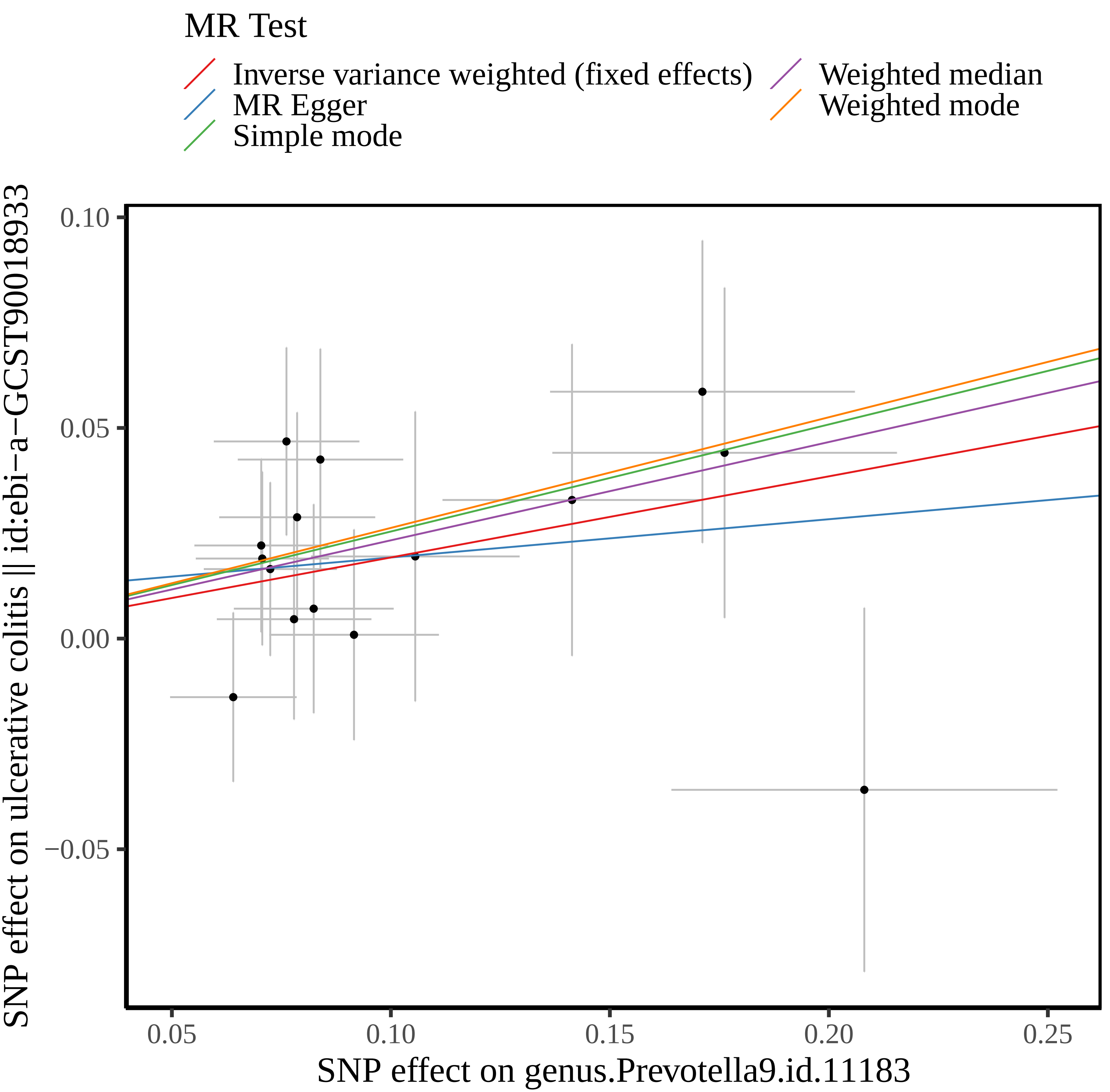
MR Test
Inverse variance weighted (fixed effects)
MR Egger
Simple mode
Weighted median
Weighted mode
0.10
0.05
0.00
−0.05
0.05
0.10
0.15
0.20
0.25
SNP effect on ulcerative colitis || id:ebi−a−GCST90018933
SNP effect on genus.Prevotella9.id.11183

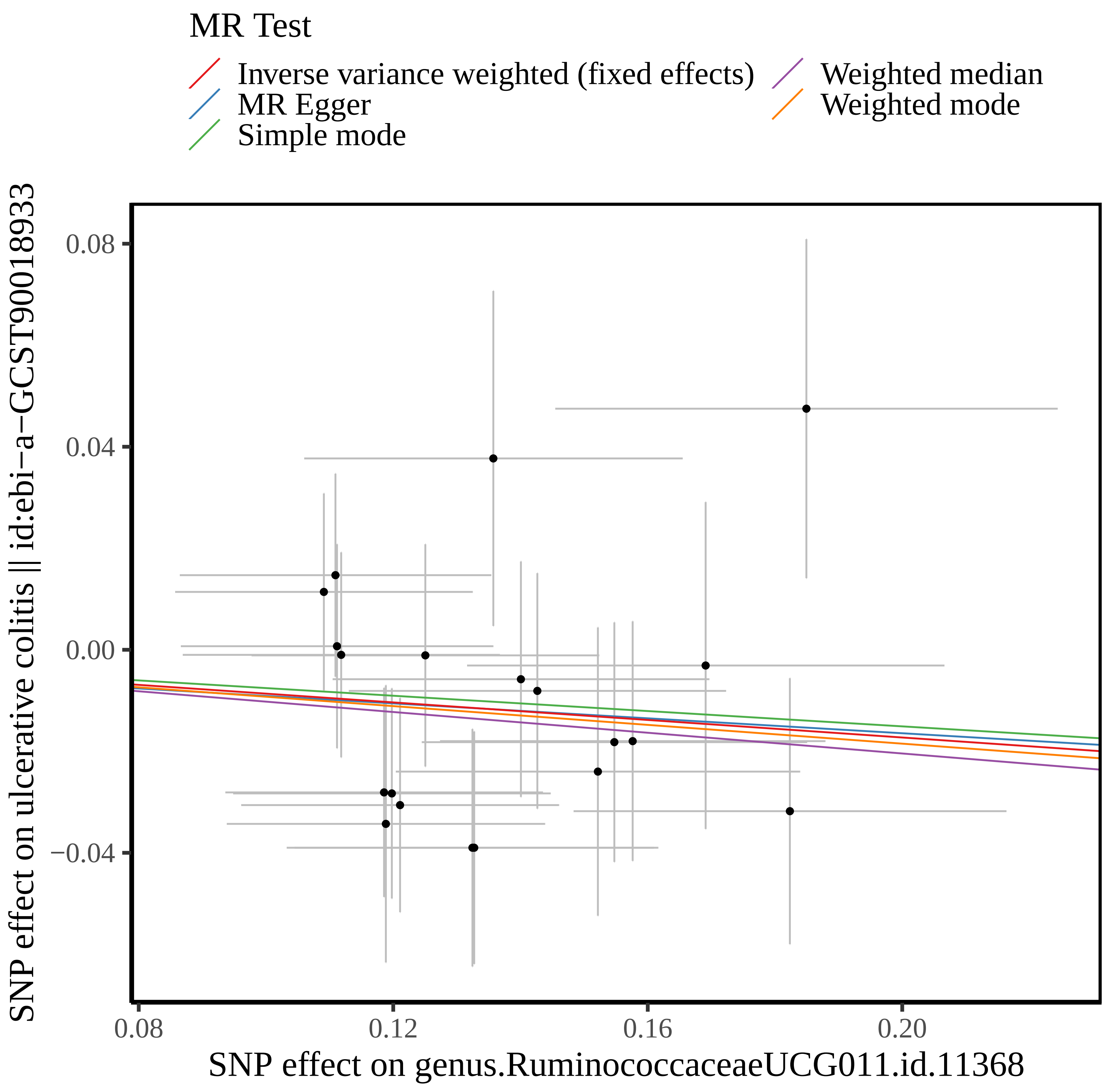
MR Test
Inverse variance weighted (fixed effects)
MR Egger
Simple mode
Weighted median
Weighted mode
SNP effect on ulcerative colitis || id:ebi−a−GCST90018933
0.08
0.04
0.00
−0.04
0.08
0.12
0.16
0.20
SNP effect on genus.RuminococcaceaeUCG011.id.11368

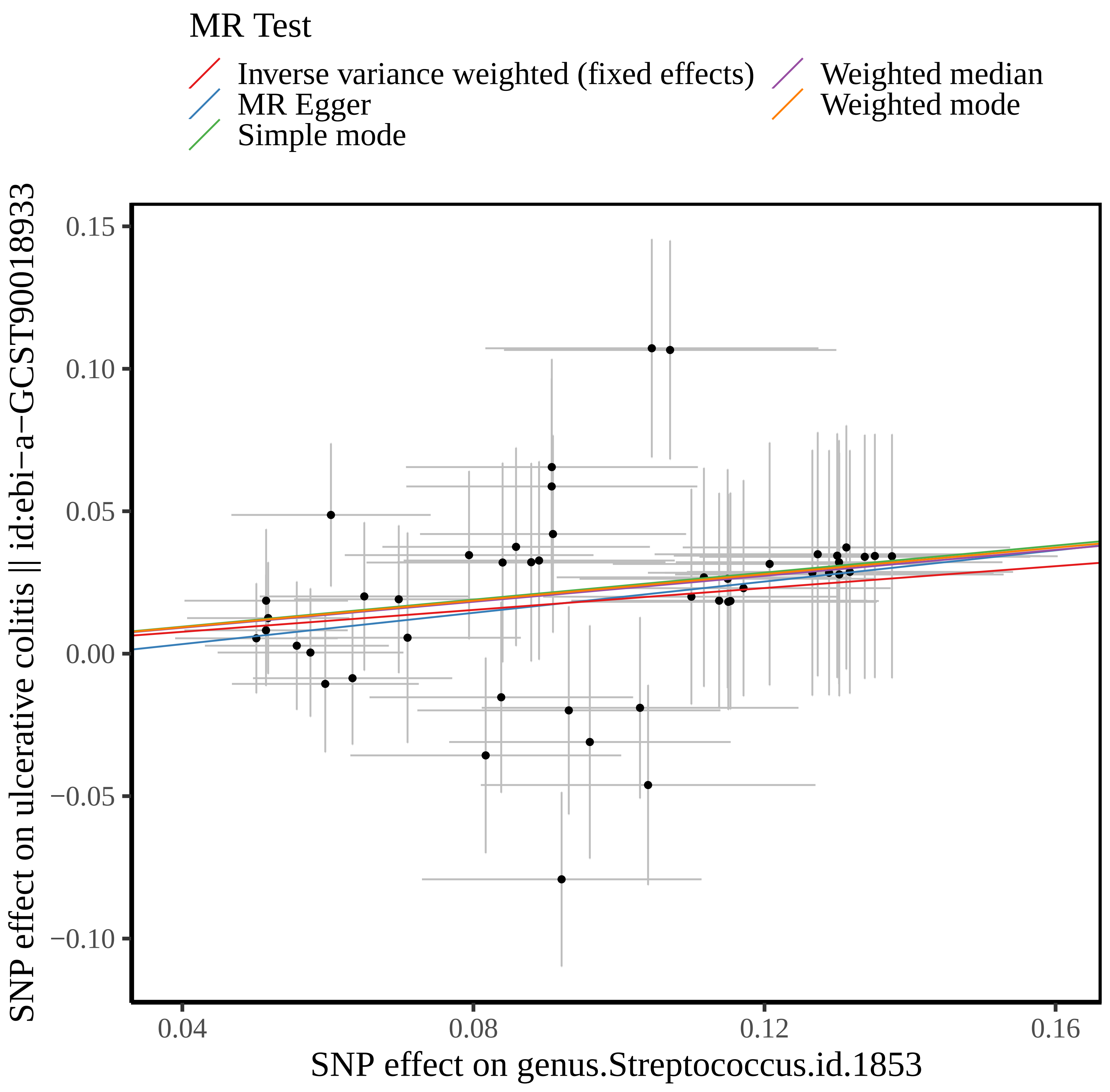
MR Test
Inverse variance weighted (fixed effects)
MR Egger
Simple mode
Weighted median
Weighted mode
SNP effect on ulcerative colitis || id:ebi−a−GCST90018933
0.15
0.10
0.05
0.00
−0.05
−0.10
0.04
0.08
0.12
0.16
SNP effect on genus.Streptococcus.id.1853

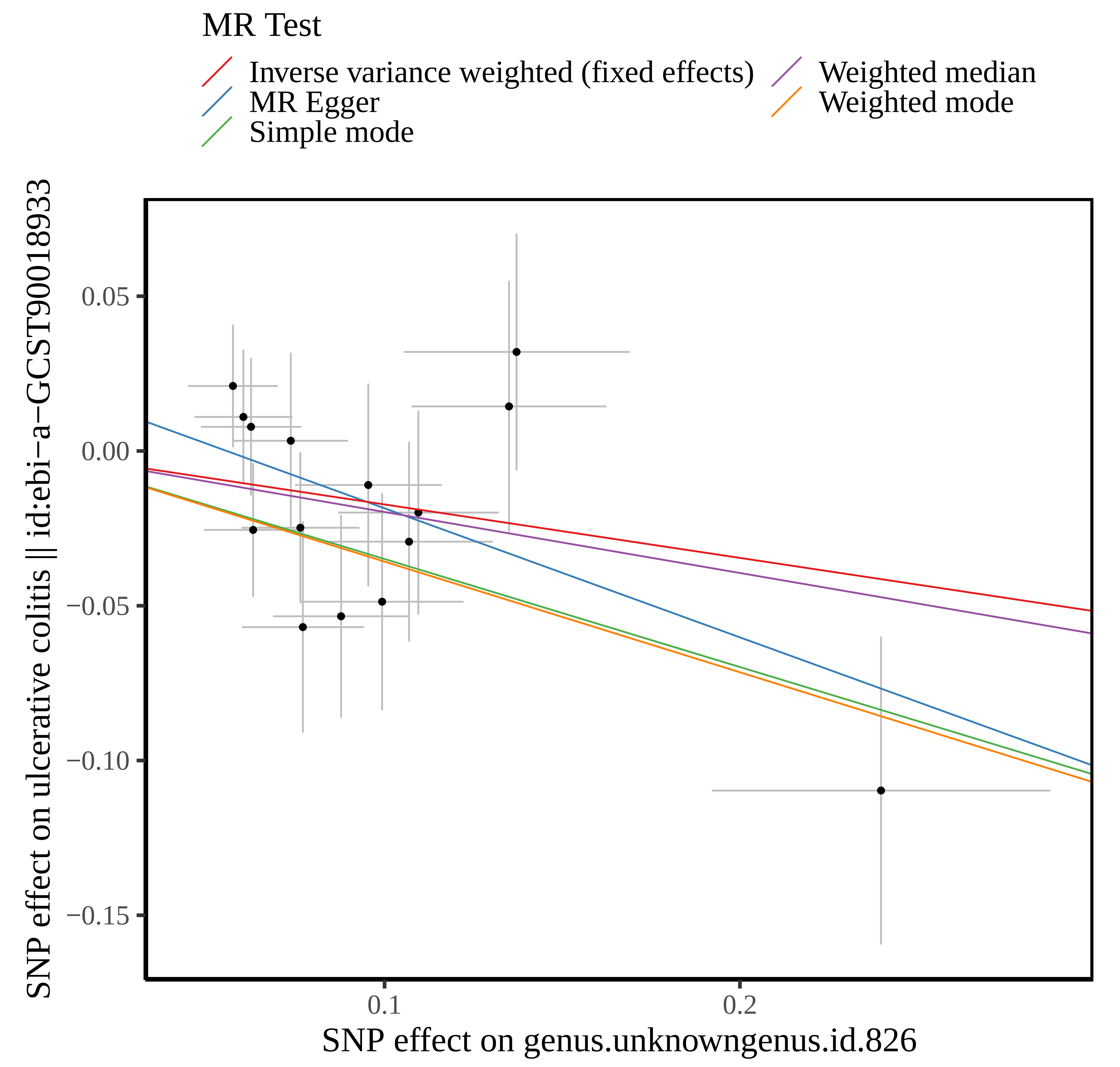

MR Test
Inverse variance weighted (fixed effects)
MR Egger
Simple mode
Weighted median
Weighted mode
SNP effect on ulcerative colitis || id:ebi−a−GCST90018933
0.05
0.00
−0.05
−0.10
−0.15
0.1
0.2
SNP effect on genus.unknowngenus.id.826

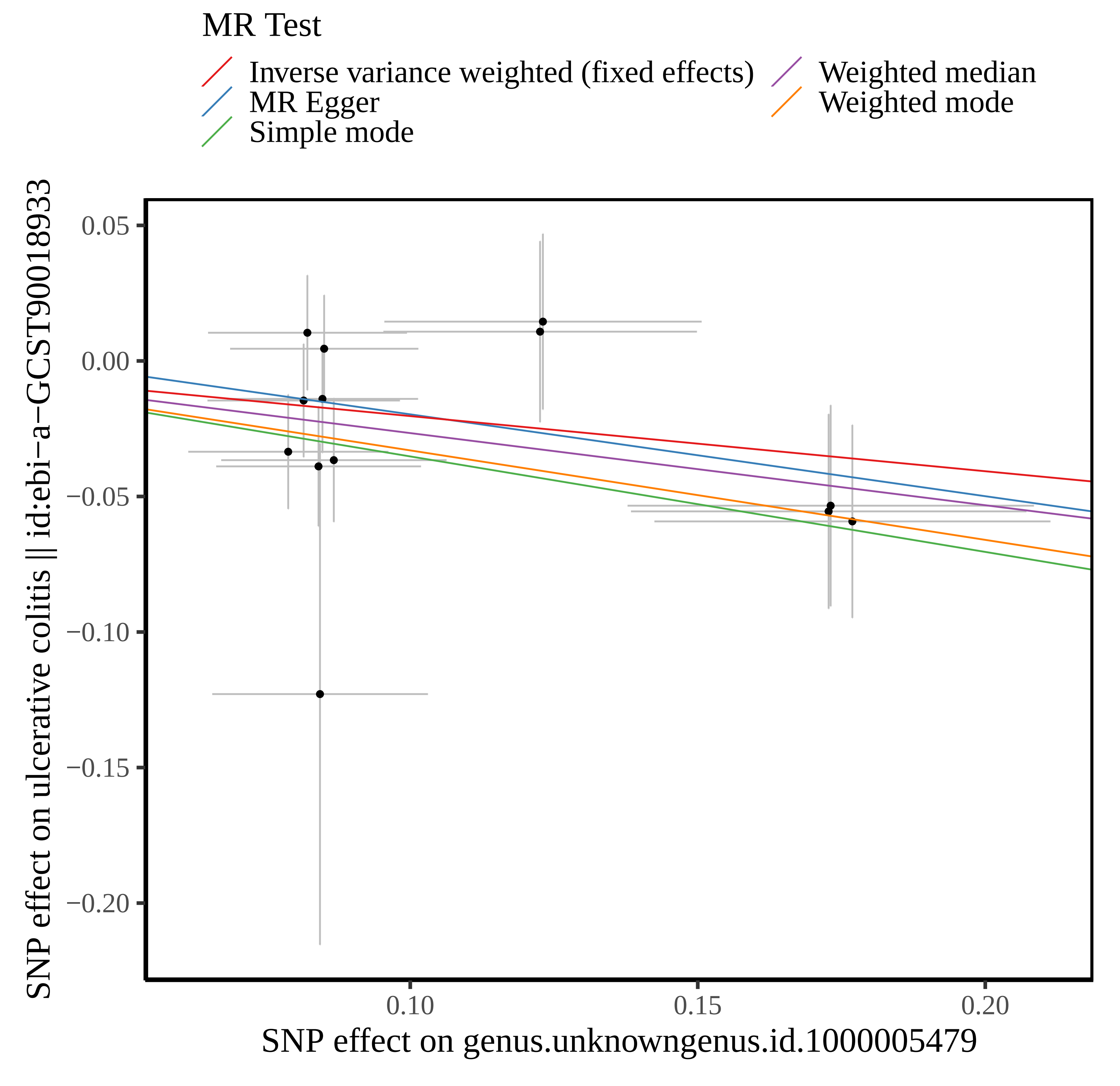

MR Test
Inverse variance weighted (fixed effects)
MR Egger
Simple mode
Weighted median
Weighted mode
SNP effect on ulcerative colitis || id:ebi−a−GCST90018933
0.05
0.00
−0.05
−0.10
−0.15
−0.20
0.10
0.15
0.20
SNP effect on genus.unknowngenus.id.1000005479

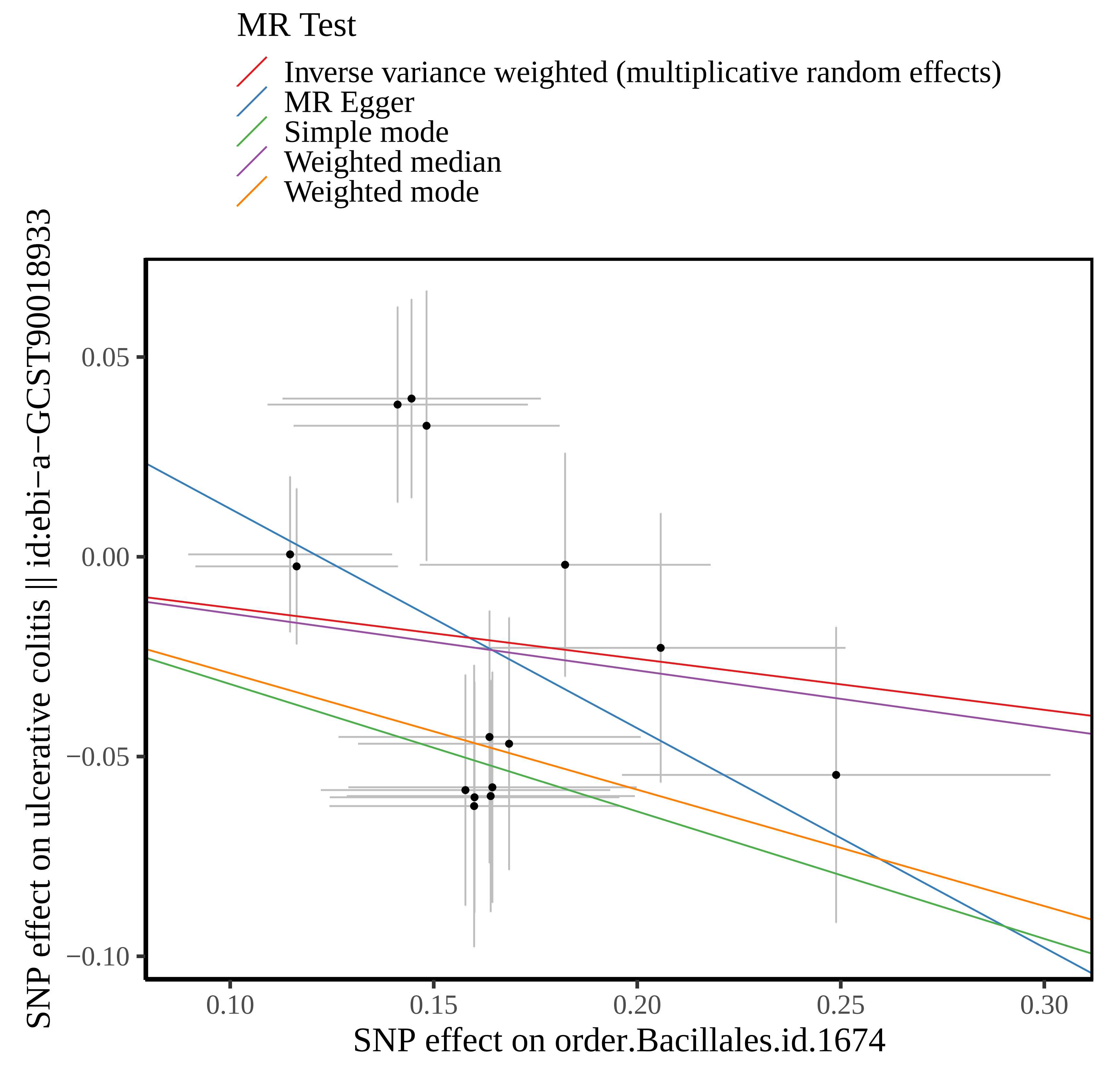

MR Test
Inverse variance weighted (multiplicative random effects)
MR Egger
Simple mode
Weighted median
Weighted mode
0.05
0.00
−0.05
−0.10
0.10
0.15
0.20
0.25
0.30
SNP effect on order.Bacillales.id.1674
SNP effect on ulcerative colitis || id:ebi−a−GCST90018933

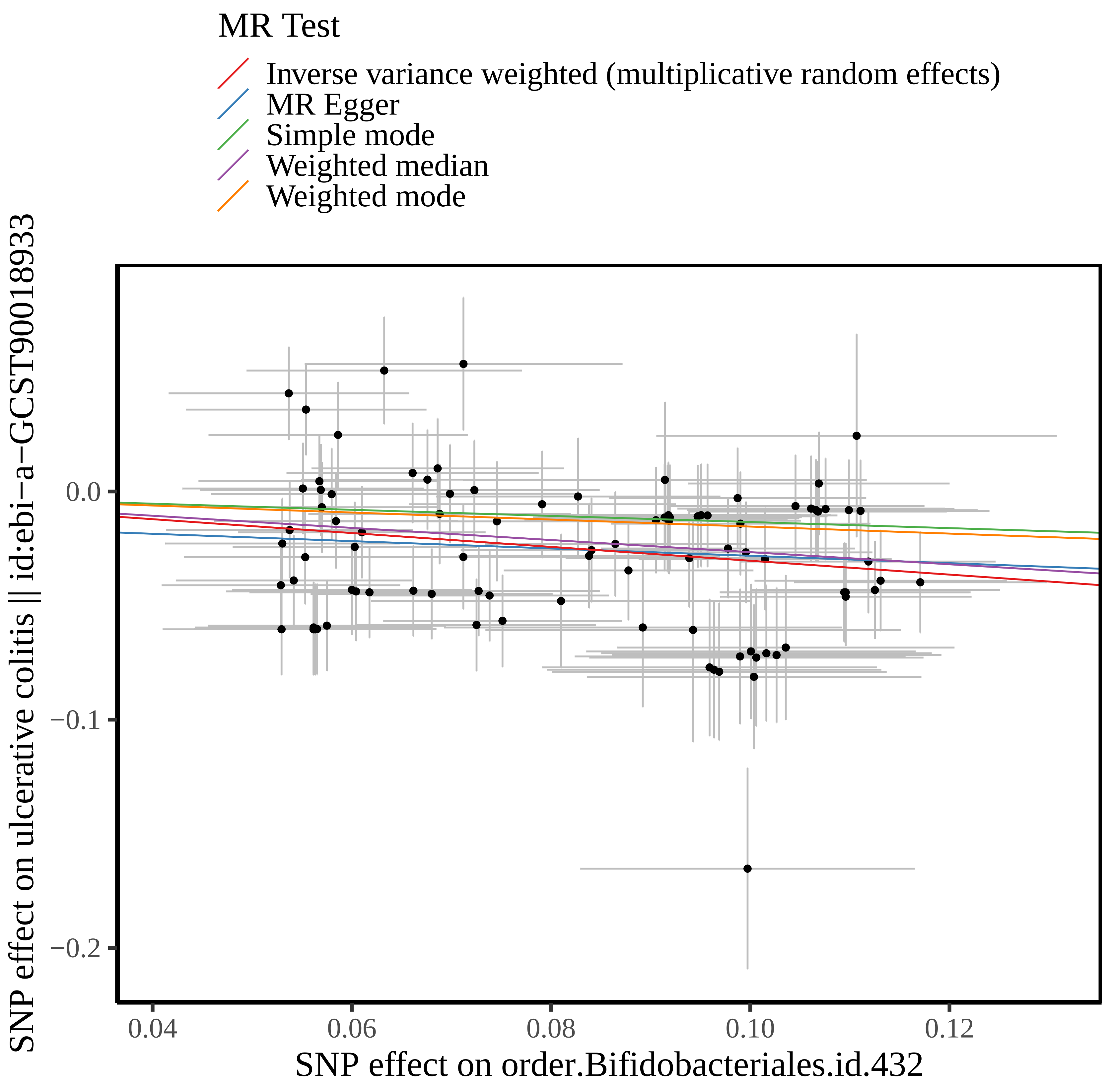

MR Test
Inverse variance weighted (multiplicative random effects)
MR Egger
Simple mode
Weighted median
Weighted mode
SNP effect on ulcerative colitis || id:ebi−a−GCST90018933
0.0
−0.1
−0.2
0.04
0.06
0.08
0.10
0.12
SNP effect on order.Bifidobacteriales.id.432

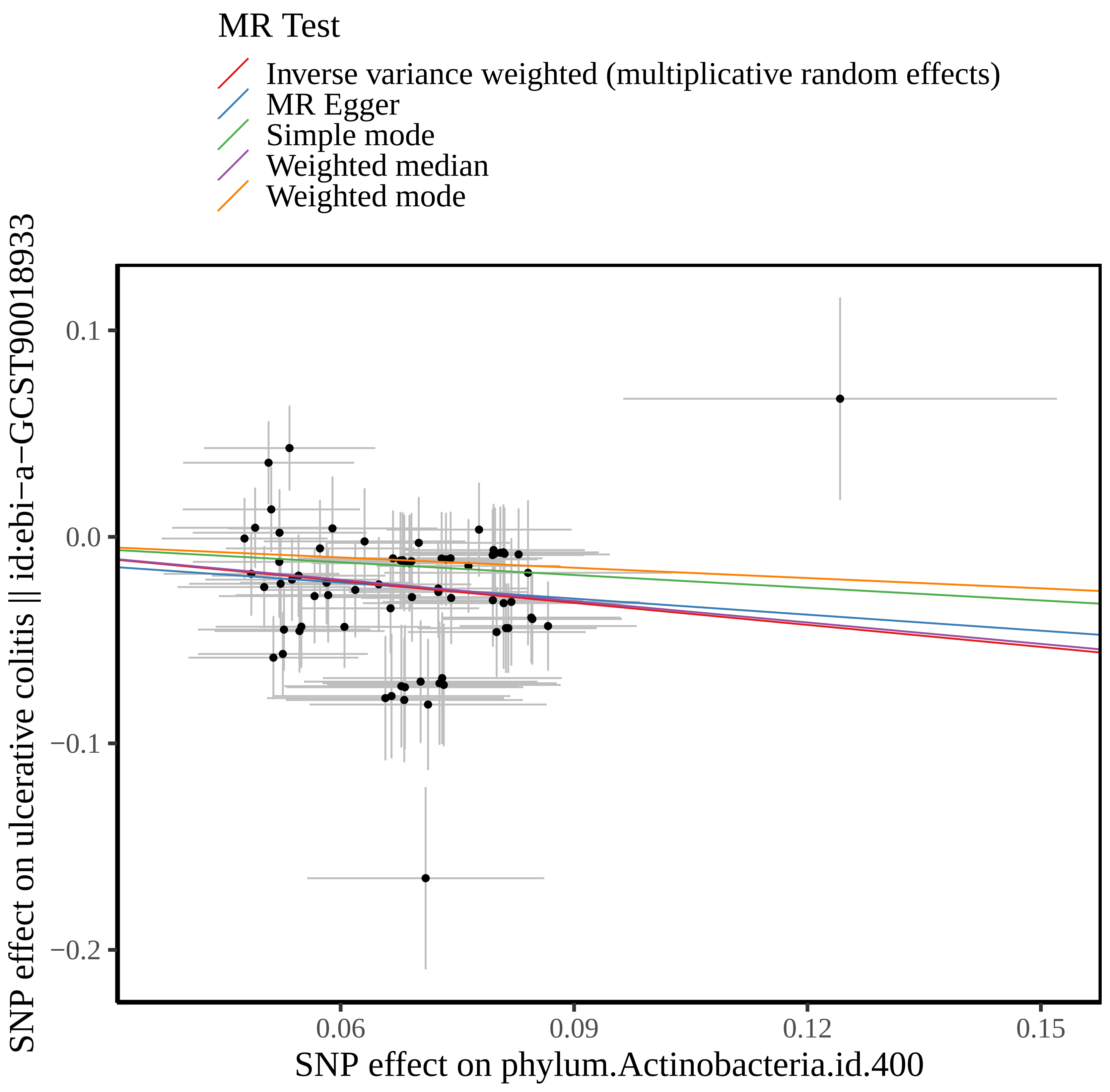
MR Test
Inverse variance weighted (multiplicative random effects)
MR Egger
Simple mode
Weighted median
Weighted mode
SNP effect on ulcerative colitis || id:ebi−a−GCST90018933
0.1
0.0
−0.1
−0.2
0.06
0.09
0.12
0.15
SNP effect on phylum.Actinobacteria.id.400

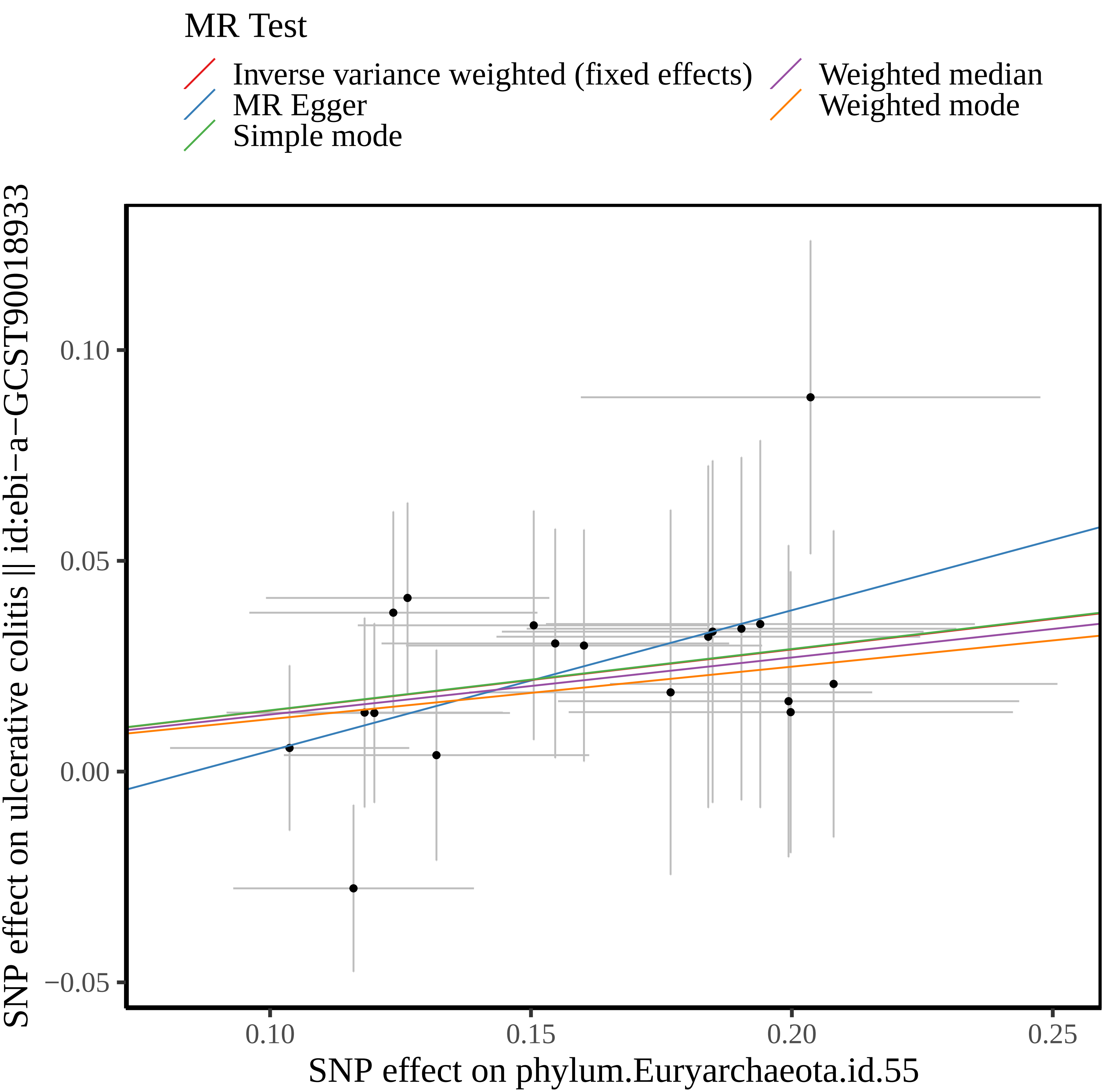
MR Test
Inverse variance weighted (fixed effects)
MR Egger
Simple mode
Weighted median
Weighted mode
SNP effect on ulcerative colitis || id:ebi−a−GCST90018933
0.10
0.05
0.00
−0.05
0.10
0.15
0.20
0.25
SNP effect on phylum.Euryarchaeota.id.55

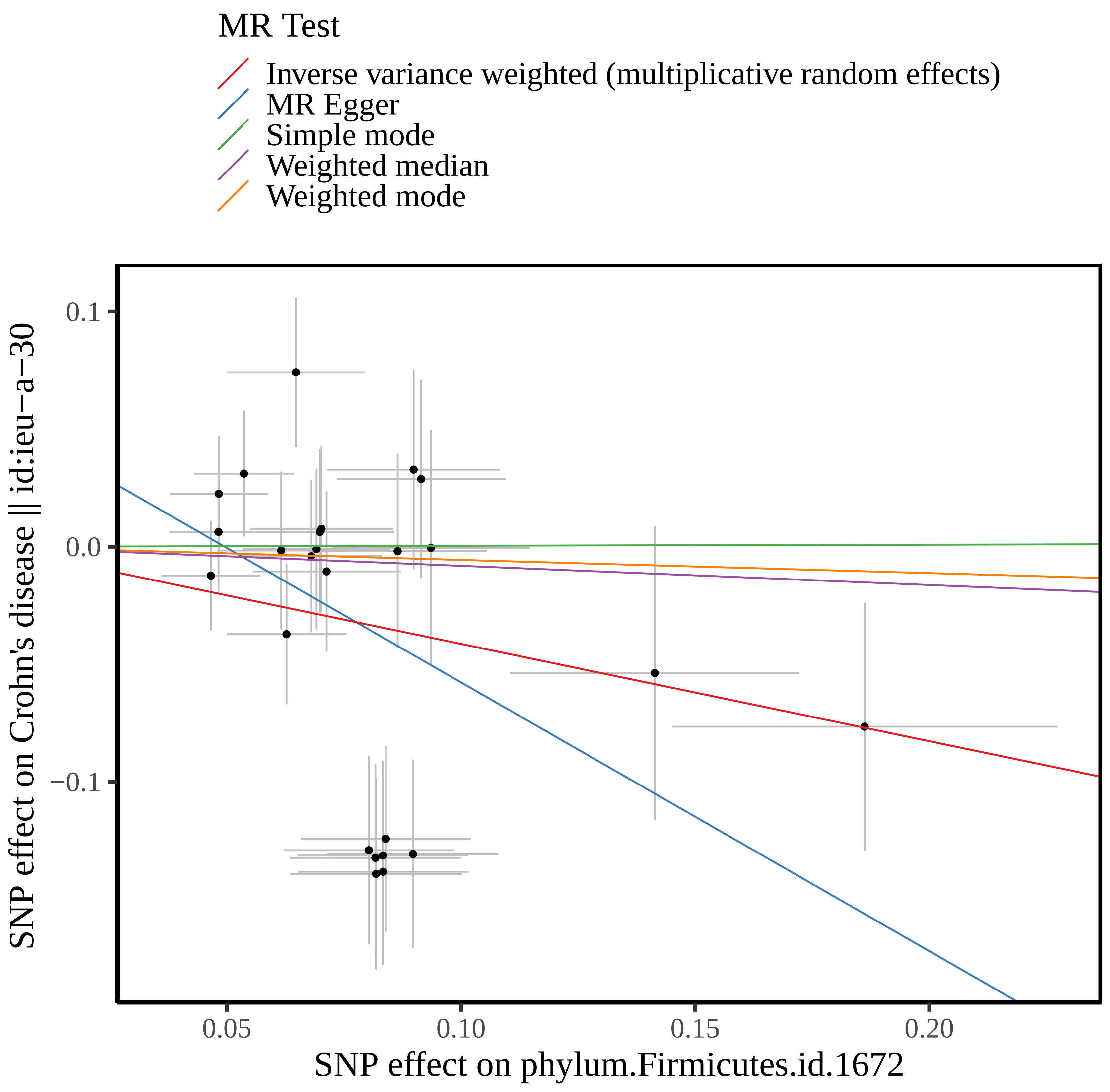
MR Test
Inverse variance weighted (multiplicative random effects)
MR Egger
Simple mode
Weighted median
Weighted mode
0.1
0.0
−0.1
0.05
0.10
0.15
0.20
SNP effect on Crohn's disease || id:ieu−a−30
SNP effect on phylum.Firmicutes.id.1672

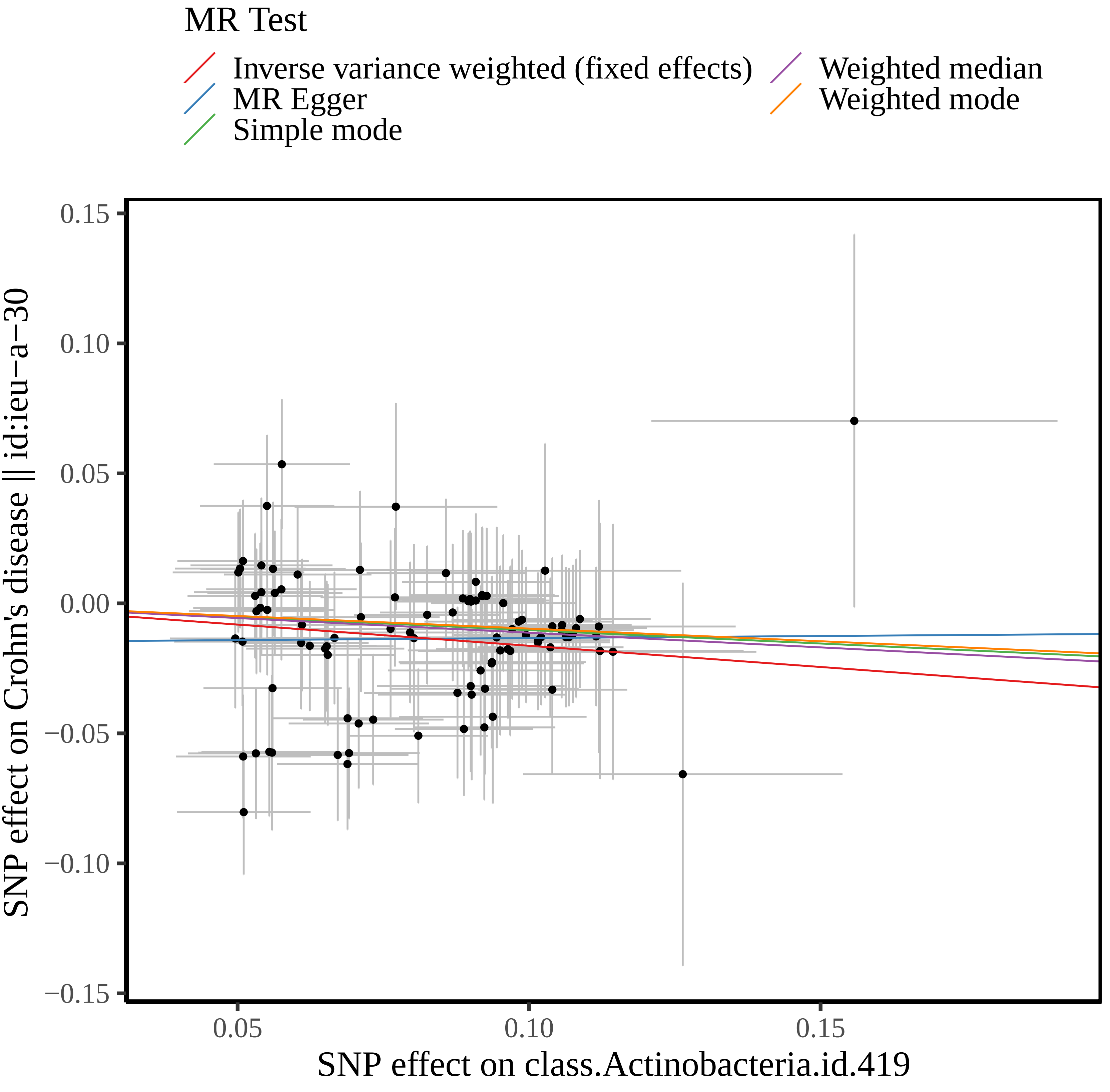

MR Test
Inverse variance weighted (fixed effects)
MR Egger
Simple mode
Weighted median
Weighted mode
0.15
0.10
0.05
0.00
−0.05
−0.10
−0.15
0.05
0.10
0.15
SNP effect on Crohn's disease || id:ieu−a−30
SNP effect on class.Actinobacteria.id.419

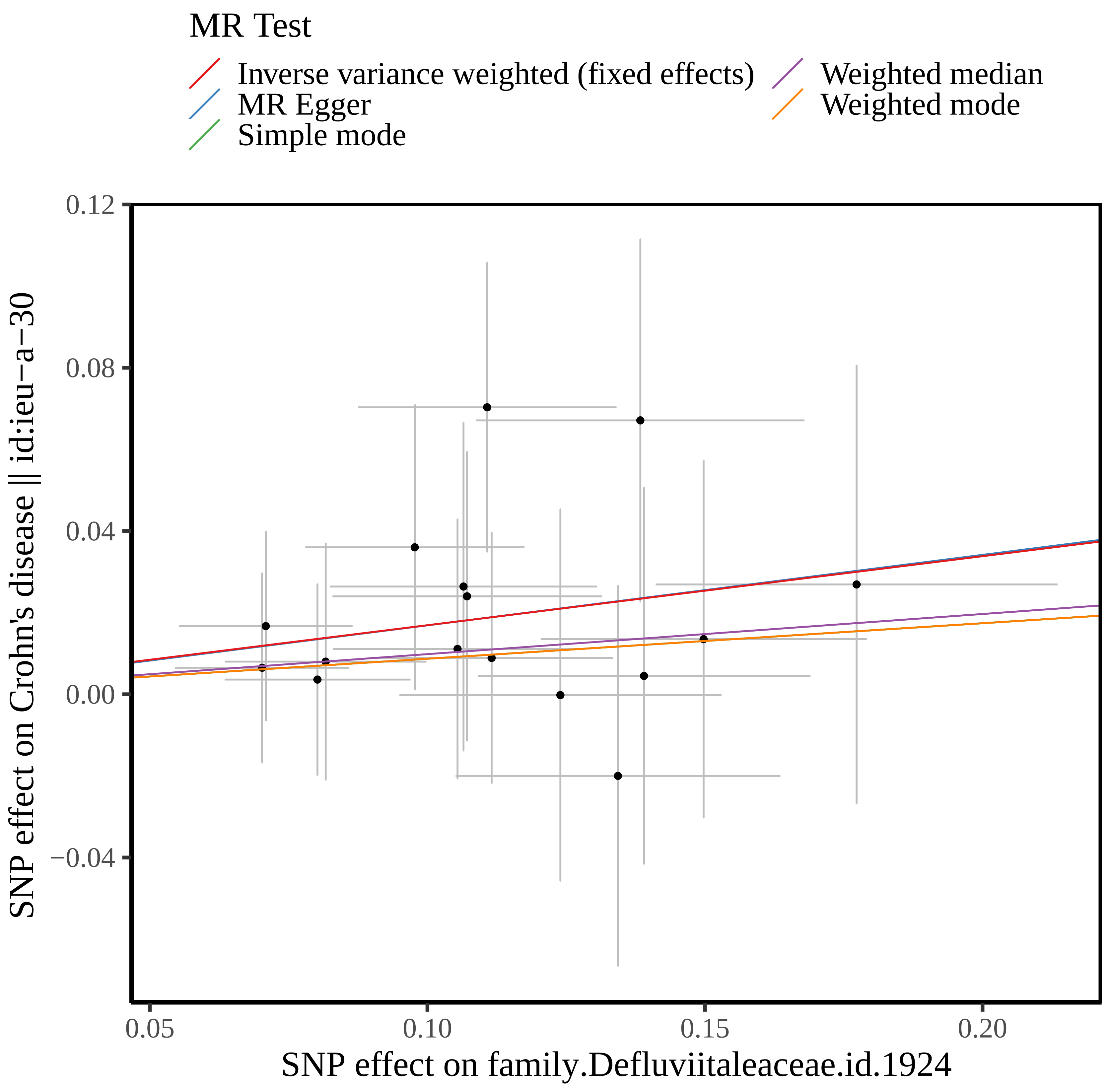
MR Test
Inverse variance weighted (fixed effects)
MR Egger
Simple mode
Weighted median
Weighted mode
0.12
0.08
0.04
0.00
−0.04
0.05
0.10
0.15
0.20
SNP effect on Crohn's disease || id:ieu−a−30
SNP effect on family.Defluviitaleaceae.id.1924

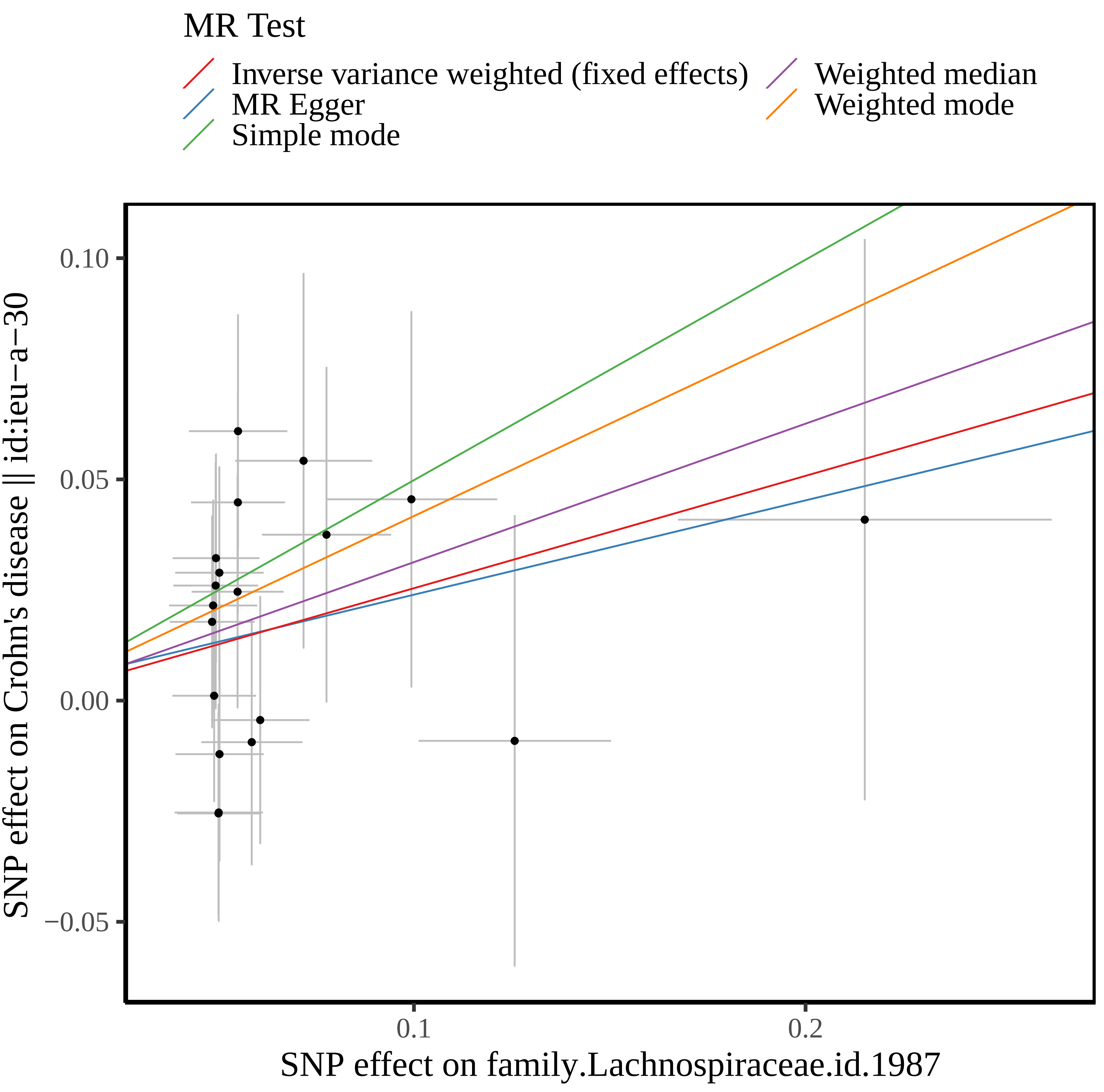

MR Test
Inverse variance weighted (fixed effects)
MR Egger
Simple mode
Weighted median
Weighted mode
0.10
0.05
0.00
−0.05
SNP effect on Crohn's disease || id:ieu−a−30
0.1
0.2
SNP effect on family.Lachnospiraceae.id.1987

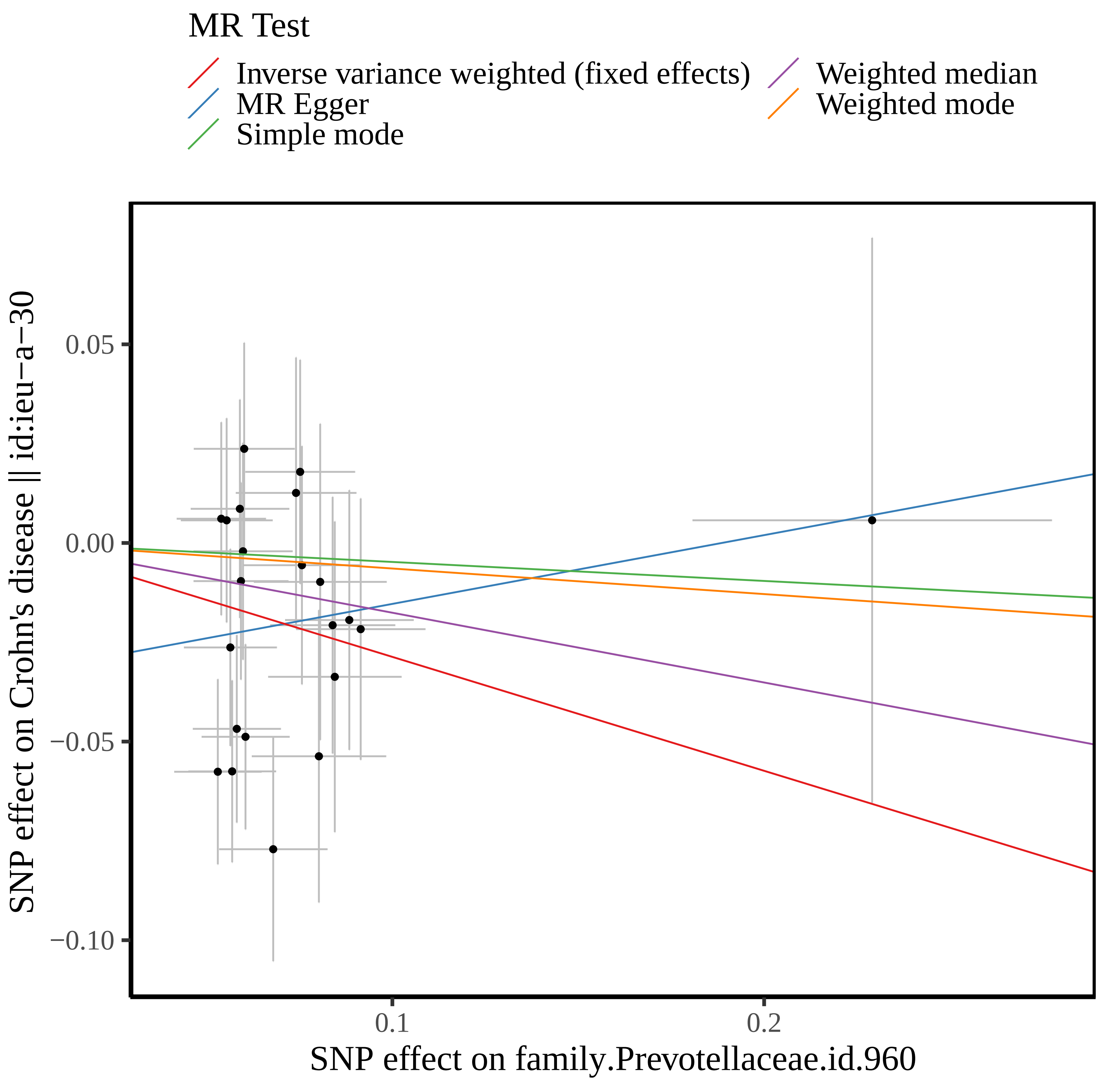
MR Test
Inverse variance weighted (fixed effects)
MR Egger
Simple mode
Weighted median
Weighted mode
SNP effect on Crohn's disease || id:ieu−a−30
0.05
0.00
−0.05
−0.10
0.1
0.2
SNP effect on family.Prevotellaceae.id.960

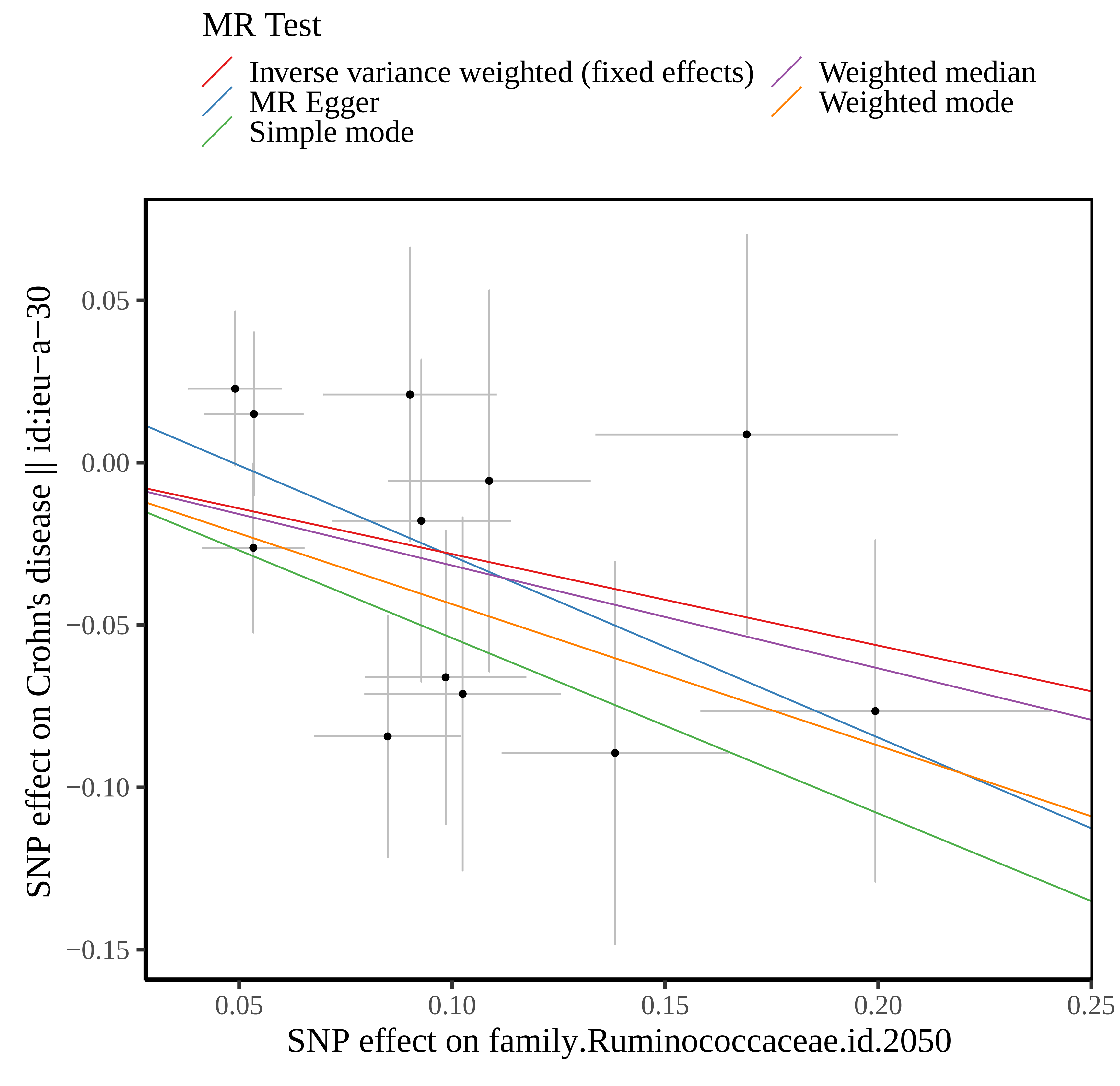
MR Test
Inverse variance weighted (fixed effects)
MR Egger
Simple mode
Weighted median
Weighted mode
SNP effect on Crohn's disease || id:ieu−a−30
0.05
0.00
−0.05
−0.10
−0.15
0.05
0.10
0.15
0.20
0.25
SNP effect on family.Ruminococcaceae.id.2050

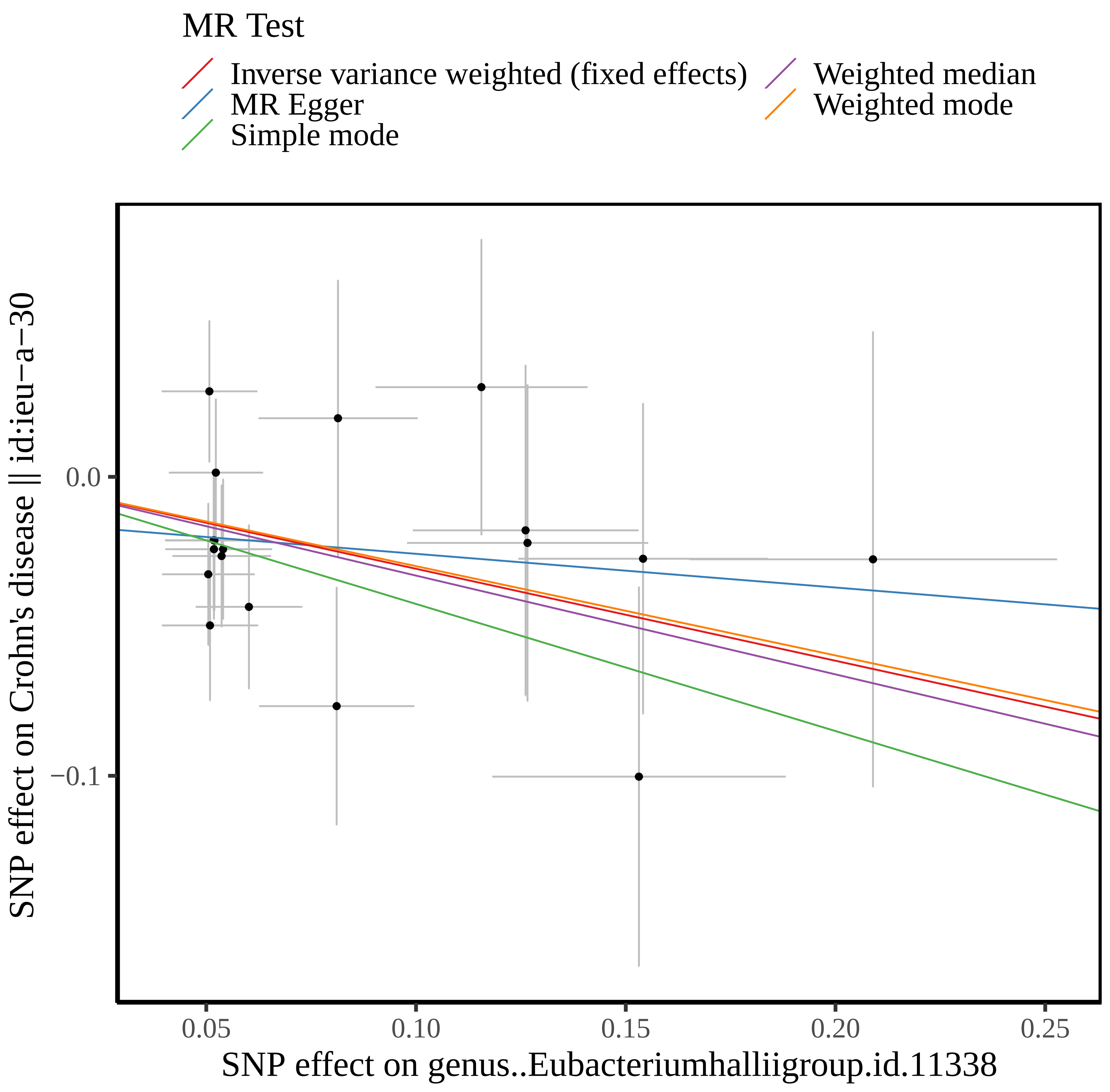

MR Test
Inverse variance weighted (fixed effects)
MR Egger
Simple mode
Weighted median
Weighted mode
0.0
−0.1
0.05
0.10
0.15
0.20
0.25
SNP effect on Crohn's disease || id:ieu−a−30
SNP effect on genus..Eubacteriumhalliigroup.id.11338

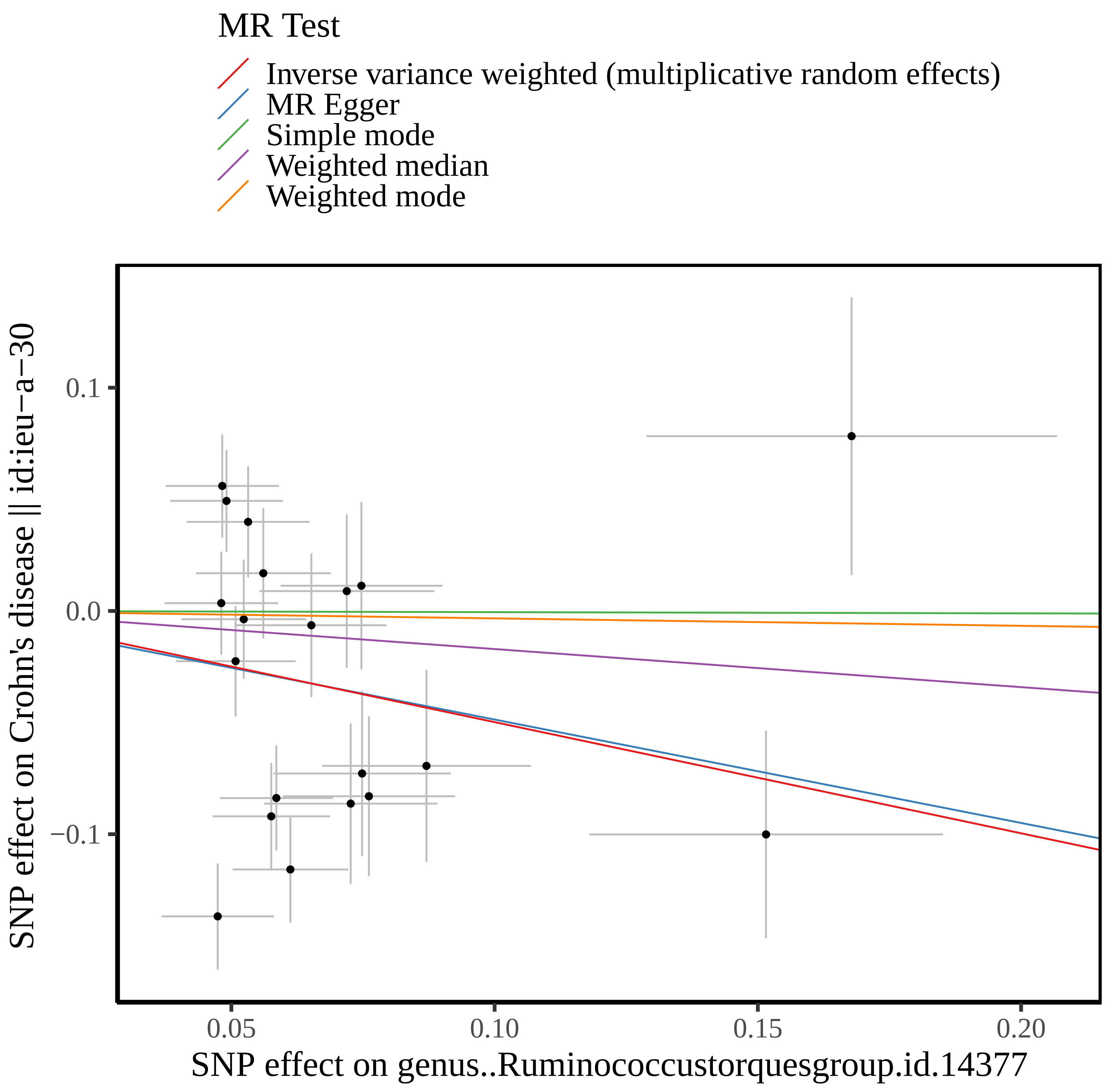

MR Test
Inverse variance weighted (multiplicative random effects)
MR Egger
Simple mode
Weighted median
Weighted mode
0.1
0.0
−0.1
0.05
0.10
0.15
0.20
SNP effect on Crohn's disease || id:ieu−a−30
SNP effect on genus..Ruminococcustorquesgroup.id.14377

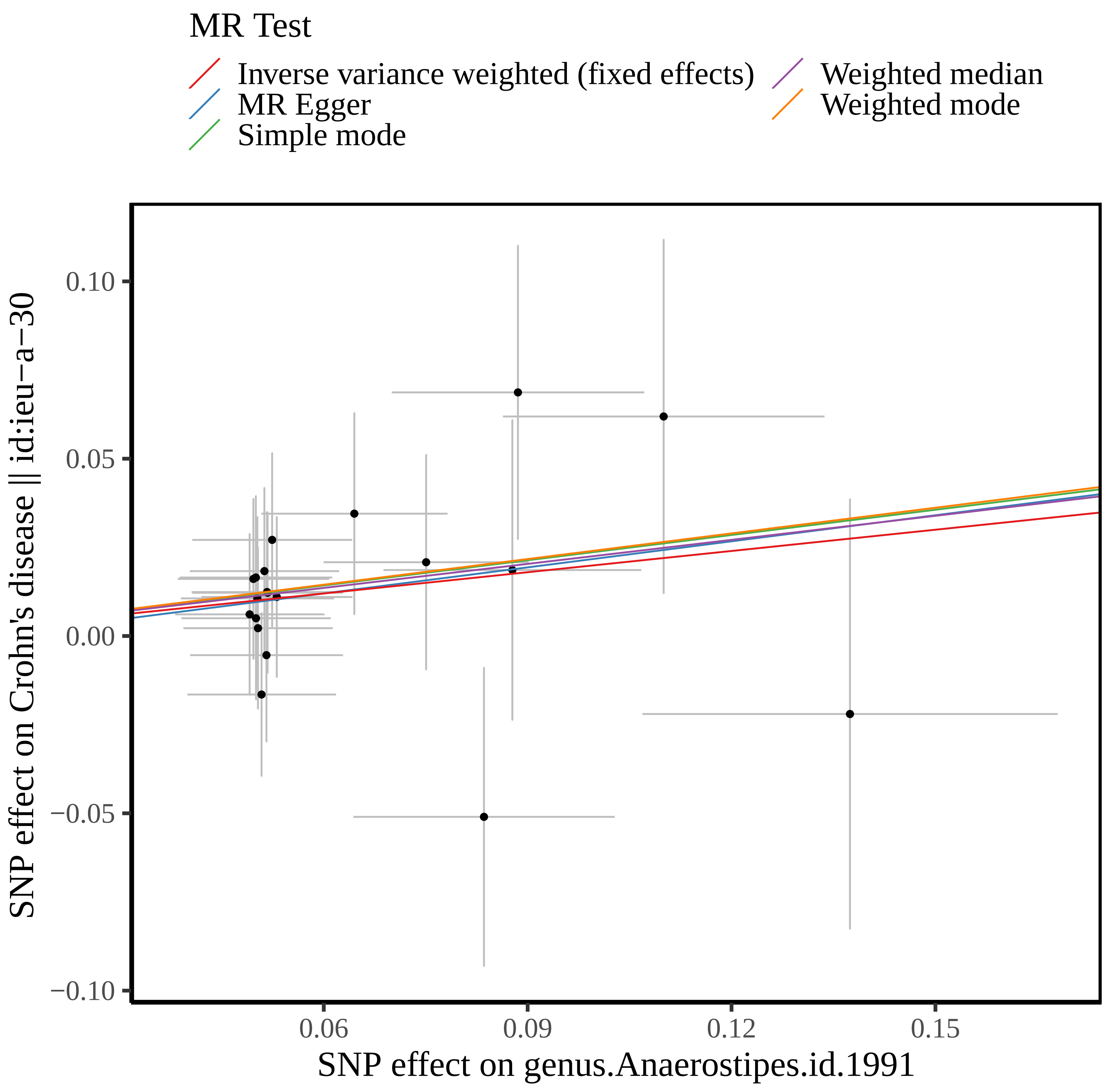
MR Test
Inverse variance weighted (fixed effects)
MR Egger
Simple mode
Weighted median
Weighted mode
0.10
0.05
0.00
−0.05
−0.10
0.06
0.09
0.12
0.15
SNP effect on Crohn's disease || id:ieu−a−30
SNP effect on genus.Anaerostipes.id.1991

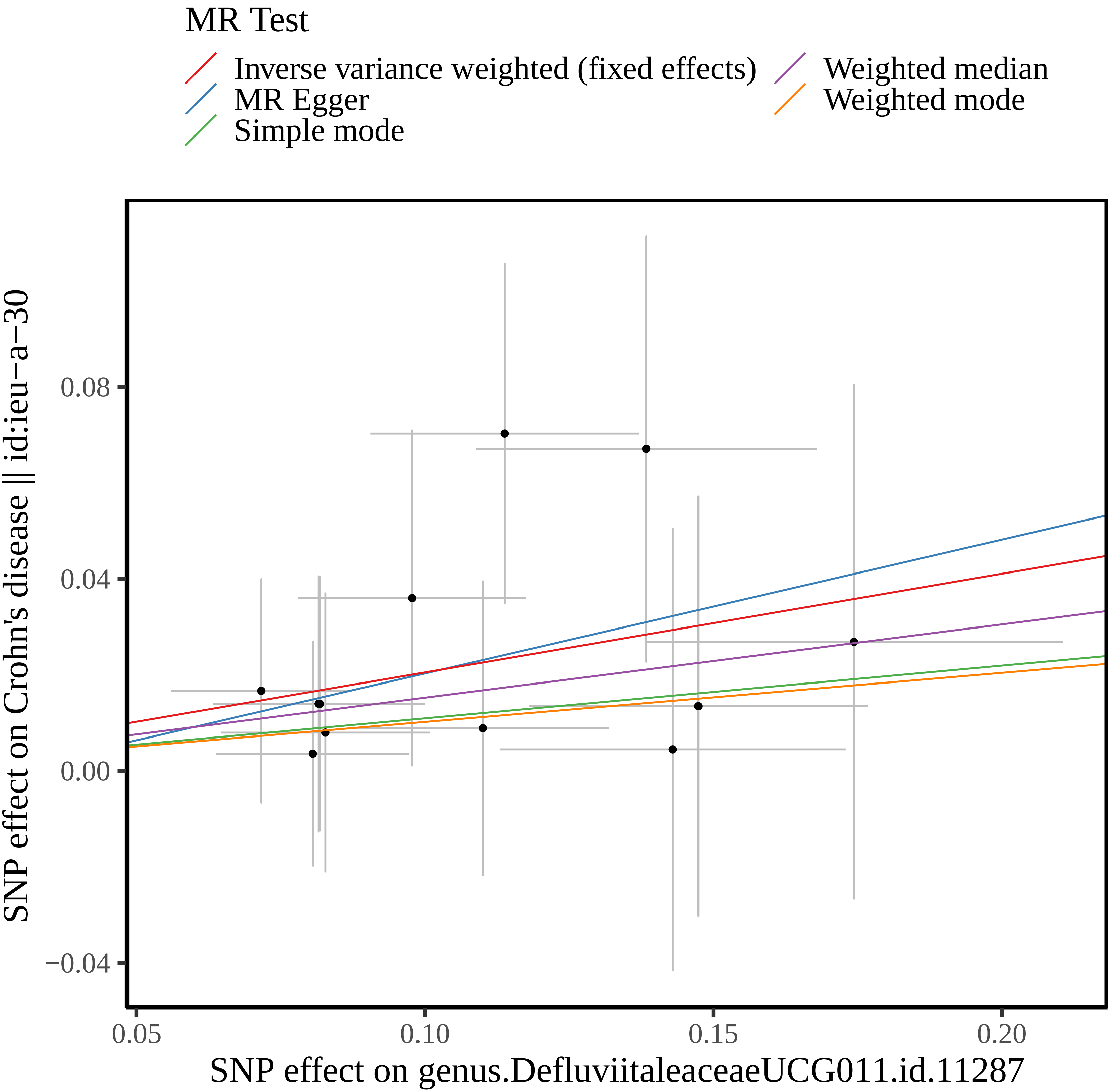
MR Test
Inverse variance weighted (fixed effects)
MR Egger
Simple mode
Weighted median
Weighted mode
SNP effect on Crohn's disease || id:ieu−a−30
0.08
0.04
0.00
−0.04
0.05
0.10
0.15
0.20
SNP effect on genus.DefluviitaleaceaeUCG011.id.11287

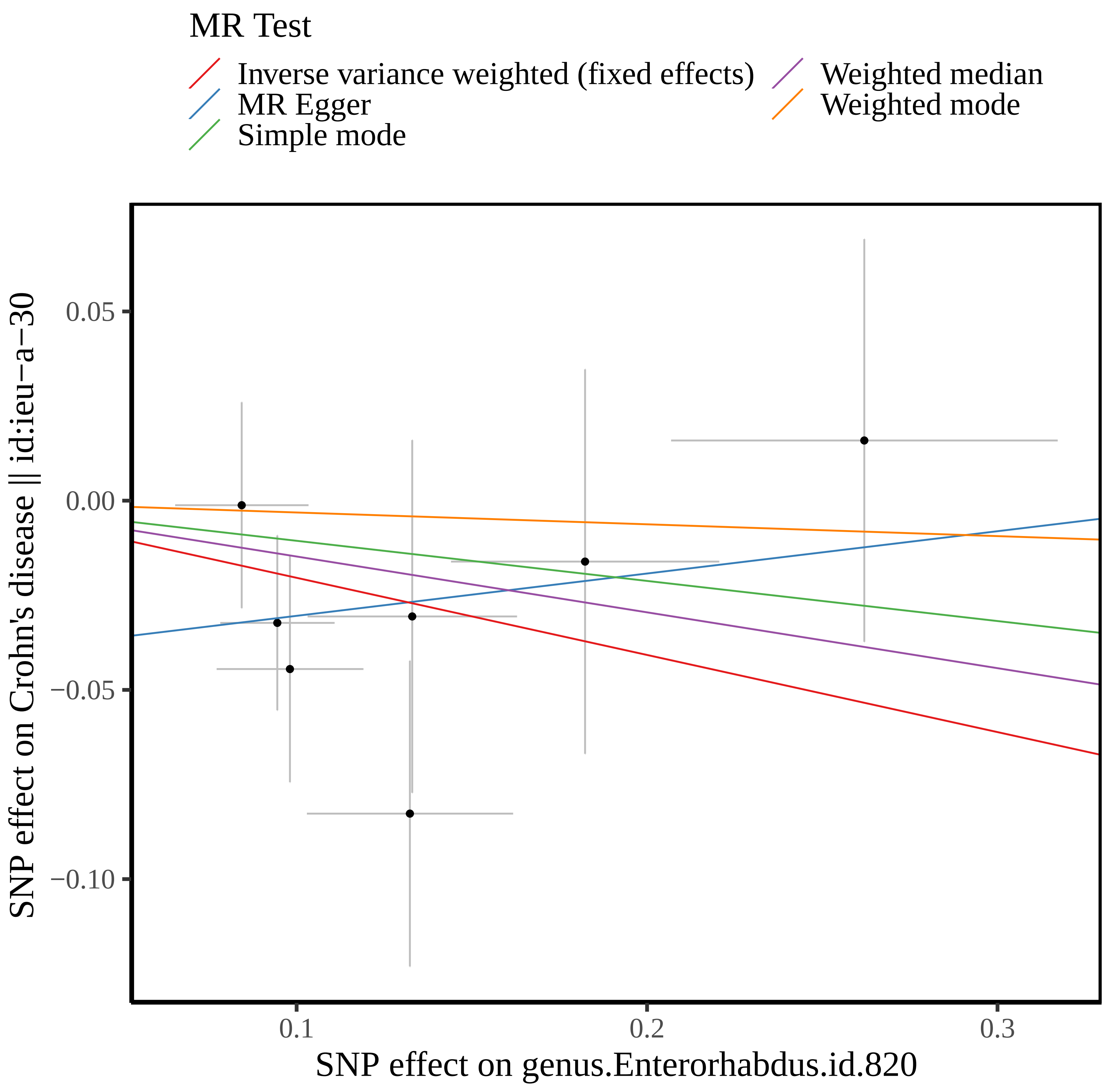

MR Test
Inverse variance weighted (fixed effects)
MR Egger
Simple mode
Weighted median
Weighted mode
SNP effect on Crohn's disease || id:ieu−a−30
0.05
0.00
−0.05
−0.10
0.1
0.2
0.3
SNP effect on genus.Enterorhabdus.id.820

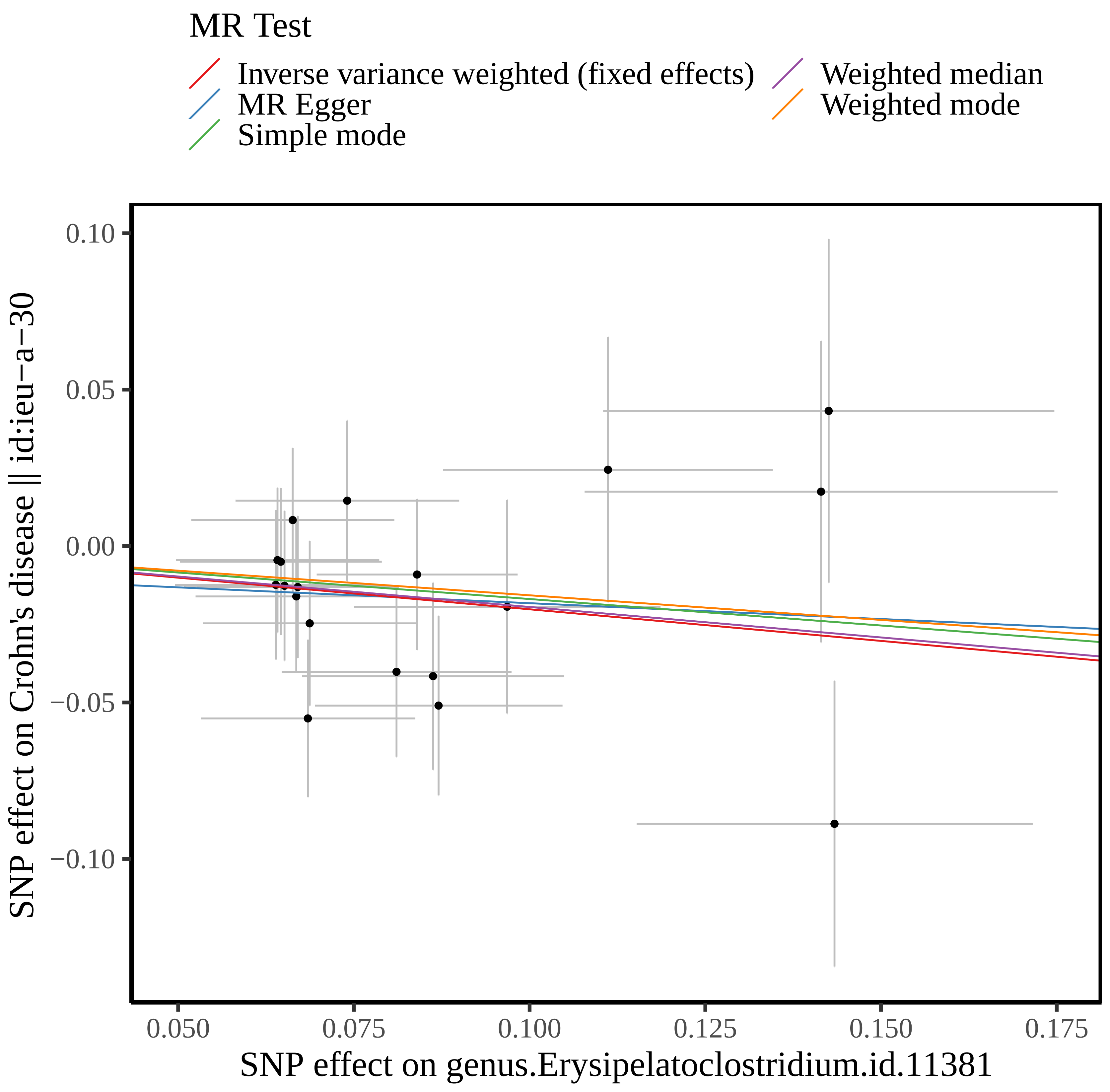
MR Test
Inverse variance weighted (fixed effects)
MR Egger
Simple mode
Weighted median
Weighted mode
0.10
0.05
0.00
−0.05
−0.10
0.050
0.075
0.100
0.125
0.150
0.175
SNP effect on Crohn's disease || id:ieu−a−30
SNP effect on genus.Erysipelatoclostridium.id.11381

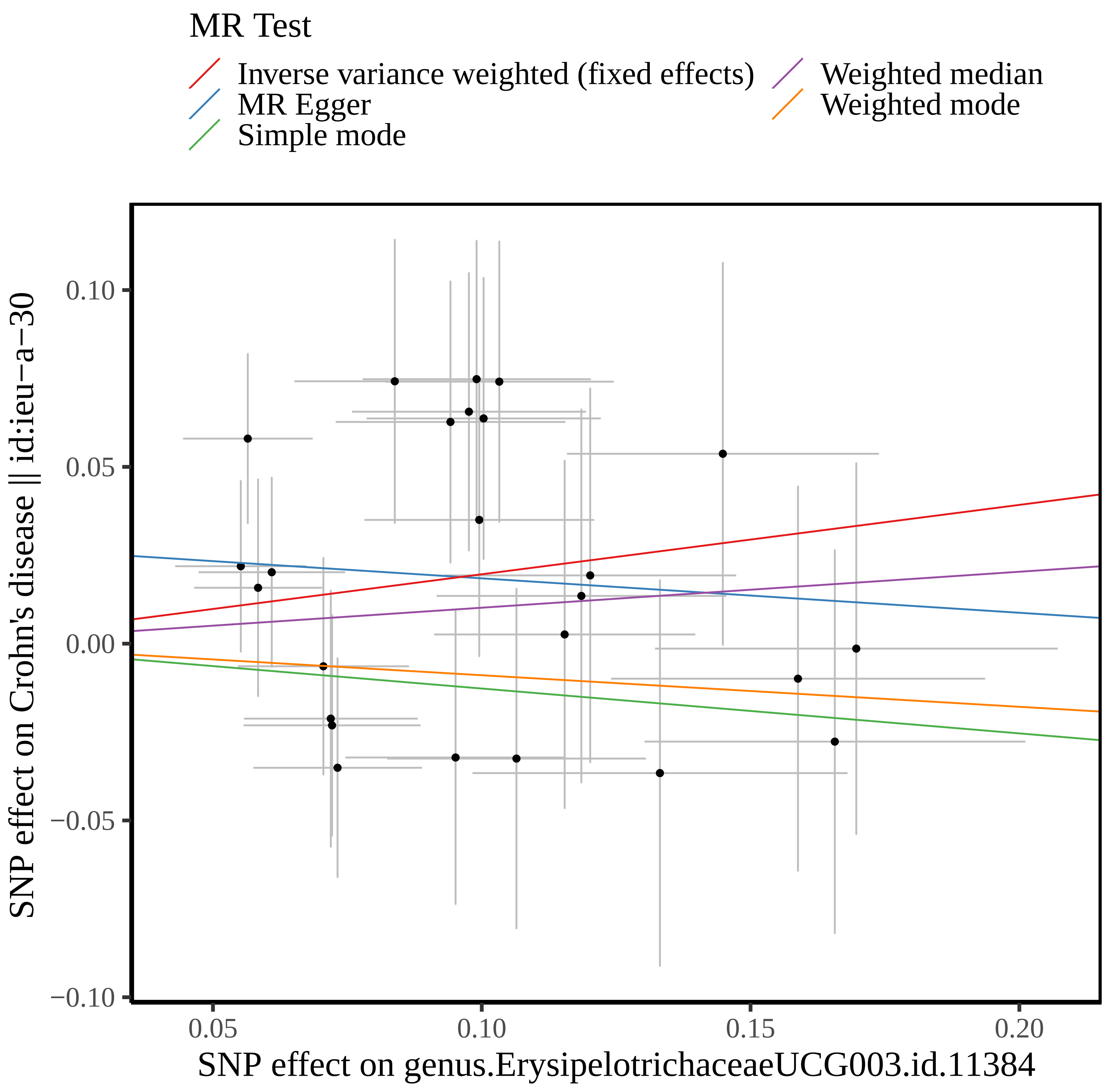
MR Test
Inverse variance weighted (fixed effects)
MR Egger
Simple mode
Weighted median
Weighted mode
SNP effect on Crohn's disease || id:ieu−a−30
0.10
0.05
0.00
−0.05
−0.10
0.05
0.10
0.15
0.20
SNP effect on genus.ErysipelotrichaceaeUCG003.id.11384

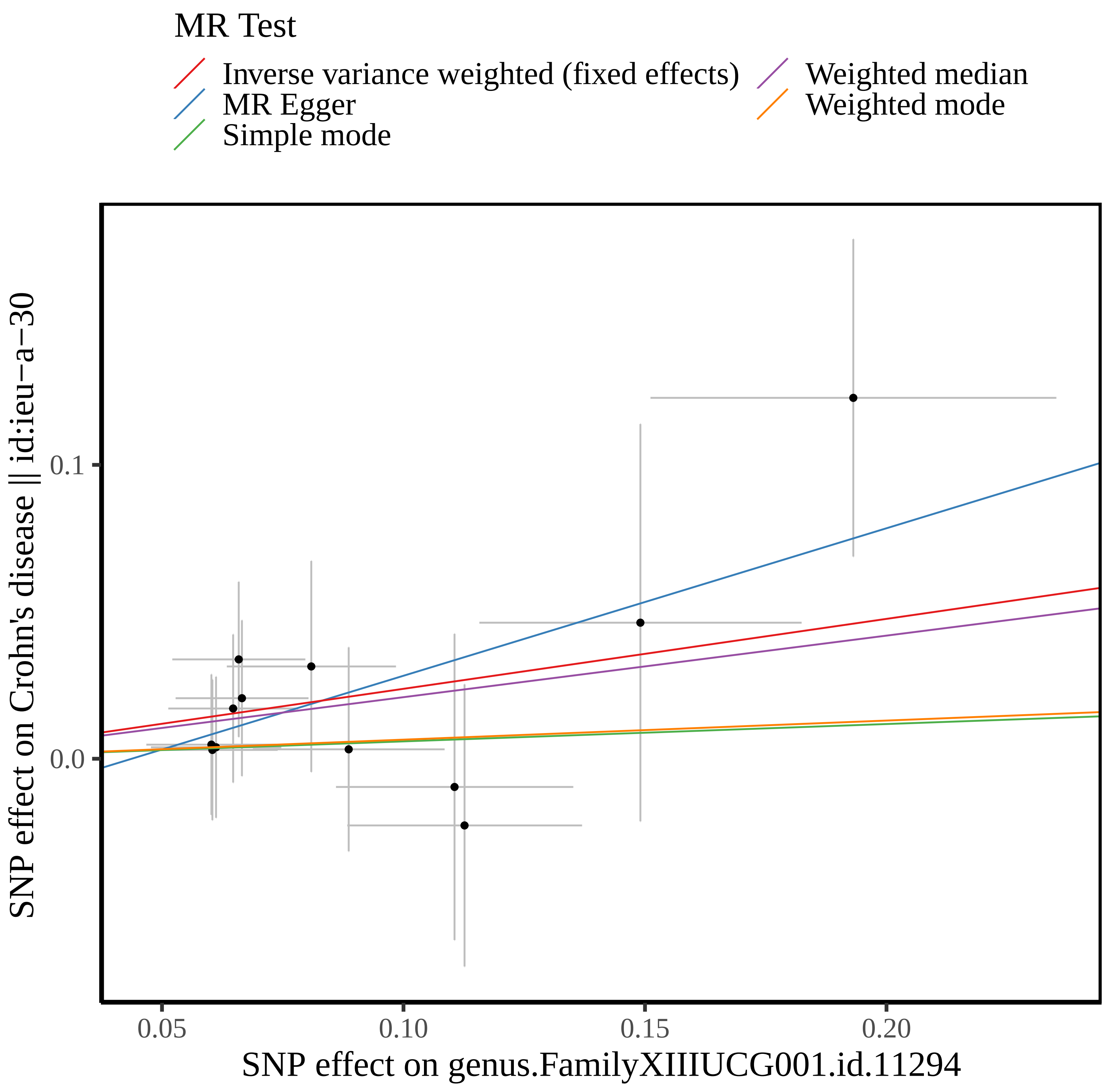

MR Test
Inverse variance weighted (fixed effects)
MR Egger
Simple mode
Weighted median
Weighted mode
SNP effect on Crohn's disease || id:ieu−a−30
0.1
0.0
0.05
0.10
0.15
0.20
SNP effect on genus.FamilyXIIIUCG001.id.11294

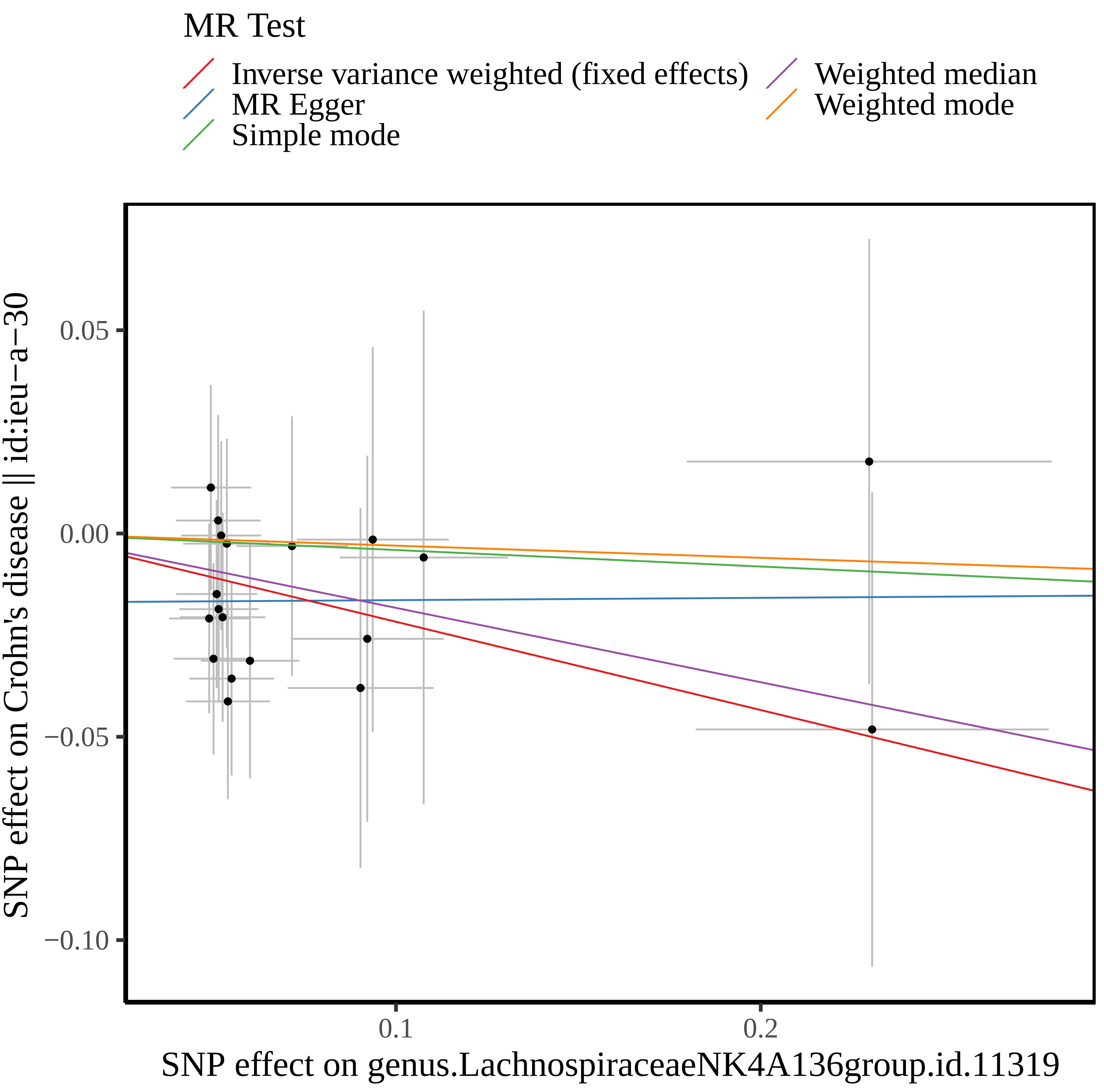

MR Test
Inverse variance weighted (fixed effects)
MR Egger
Simple mode
Weighted median
Weighted mode
0.05
0.00
−0.05
−0.10
0.1
0.2
SNP effect on Crohn's disease || id:ieu−a−30
SNP effect on genus.LachnospiraceaeNK4A136group.id.11319

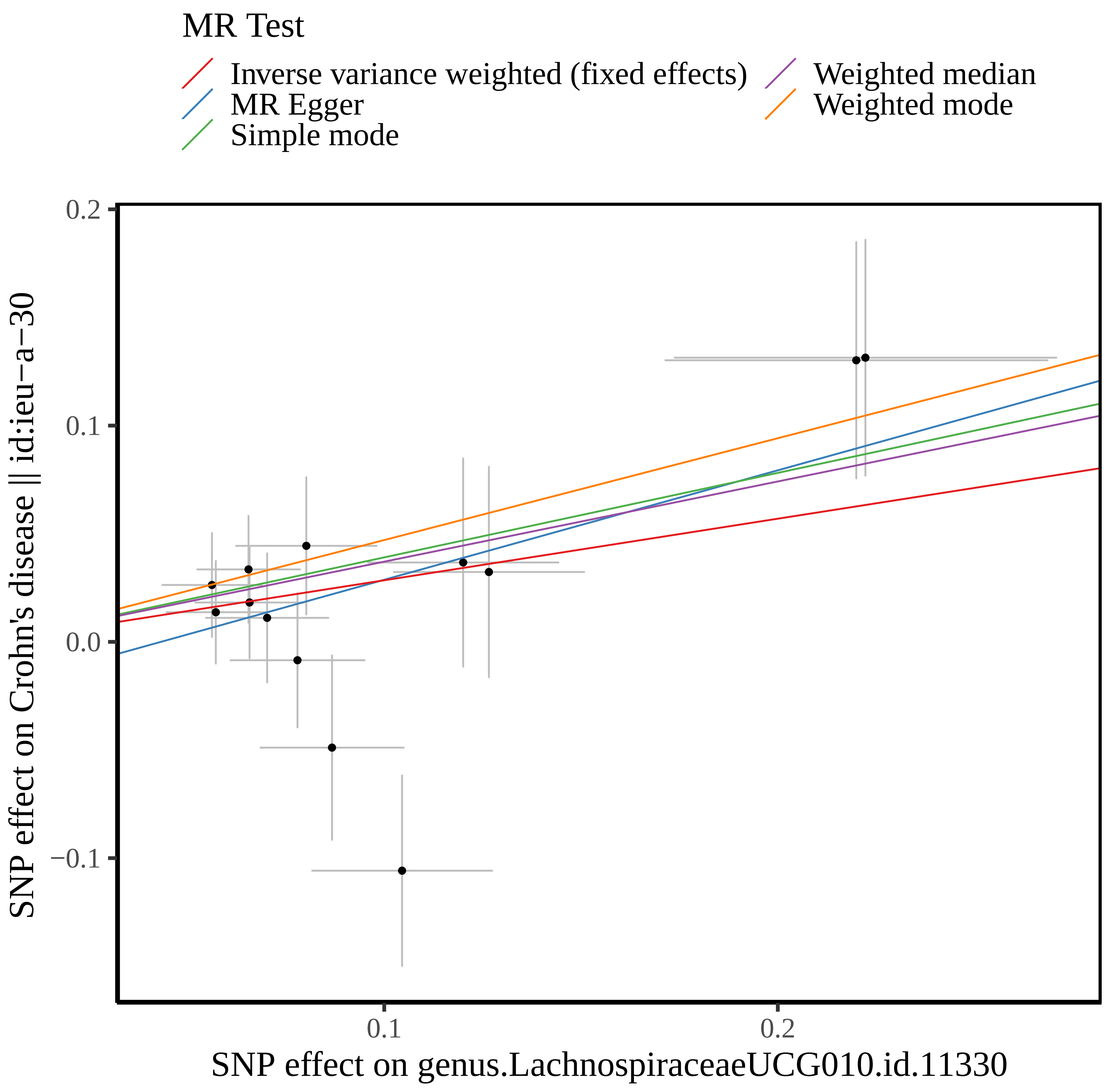

MR Test
Inverse variance weighted (fixed effects)
MR Egger
Simple mode
Weighted median
Weighted mode
0.2
0.1
0.0
−0.1
0.1
0.2
SNP effect on Crohn's disease || id:ieu−a−30
SNP effect on genus.LachnospiraceaeUCG010.id.11330

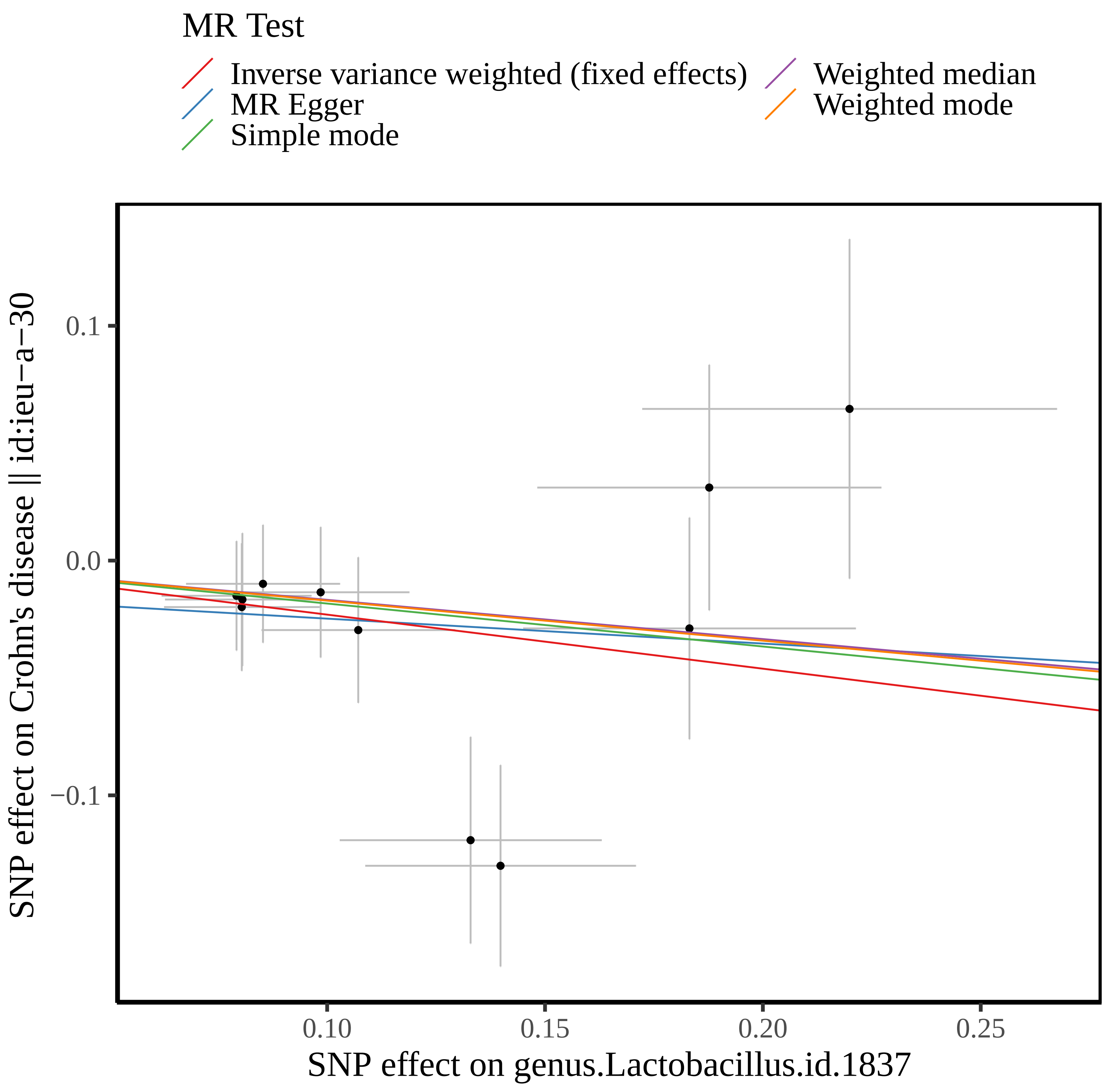
MR Test
Inverse variance weighted (fixed effects)
MR Egger
Simple mode
Weighted median
Weighted mode
SNP effect on Crohn's disease || id:ieu−a−30
0.1
0.0
−0.1
0.10
0.15
0.20
0.25
SNP effect on genus.Lactobacillus.id.1837

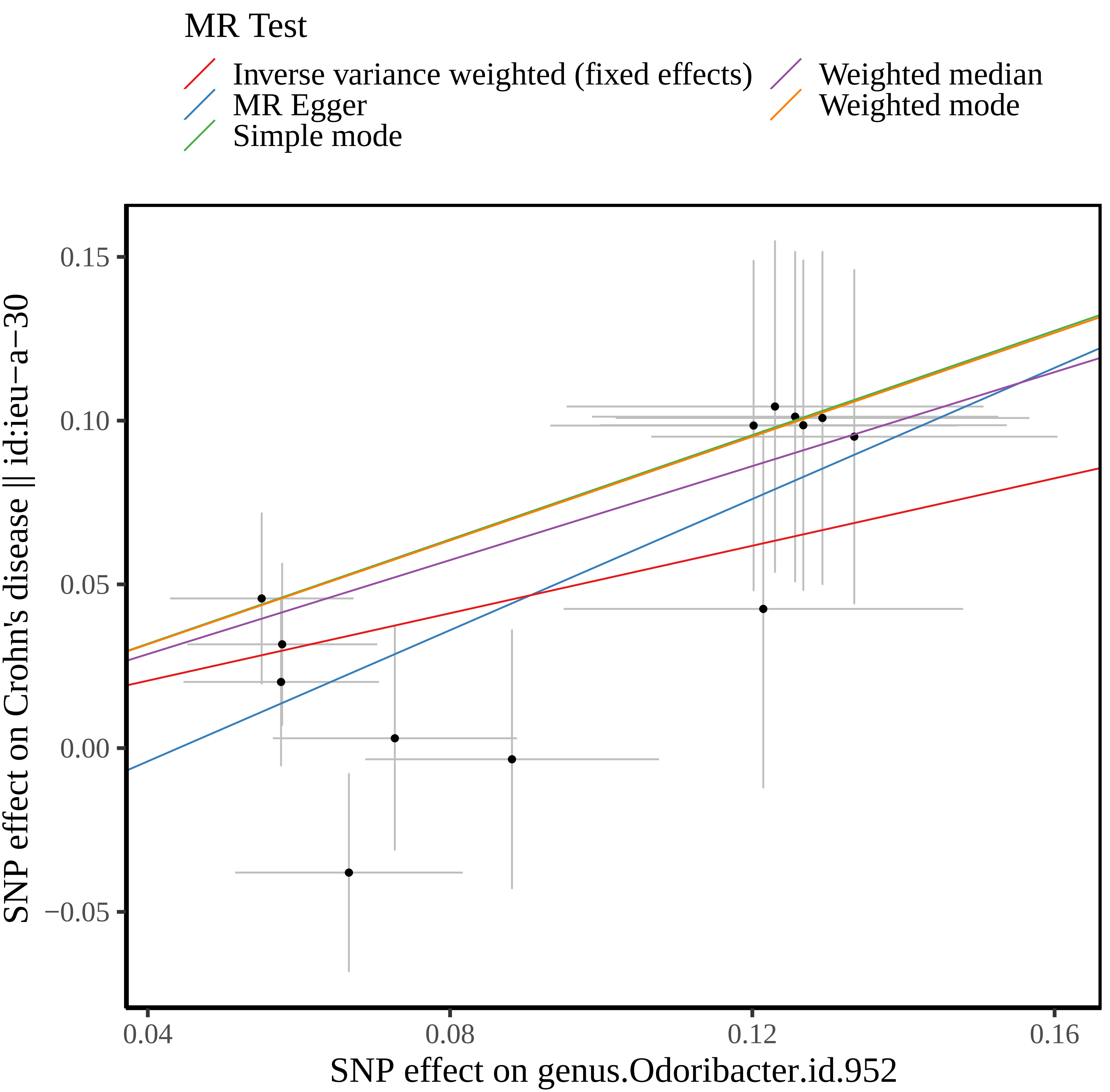

MR Test
Inverse variance weighted (fixed effects)
MR Egger
Simple mode
Weighted median
Weighted mode
0.15
0.10
0.05
0.00
−0.05
0.04
0.08
0.12
0.16
SNP effect on Crohn's disease || id:ieu−a−30
SNP effect on genus.Odoribacter.id.952

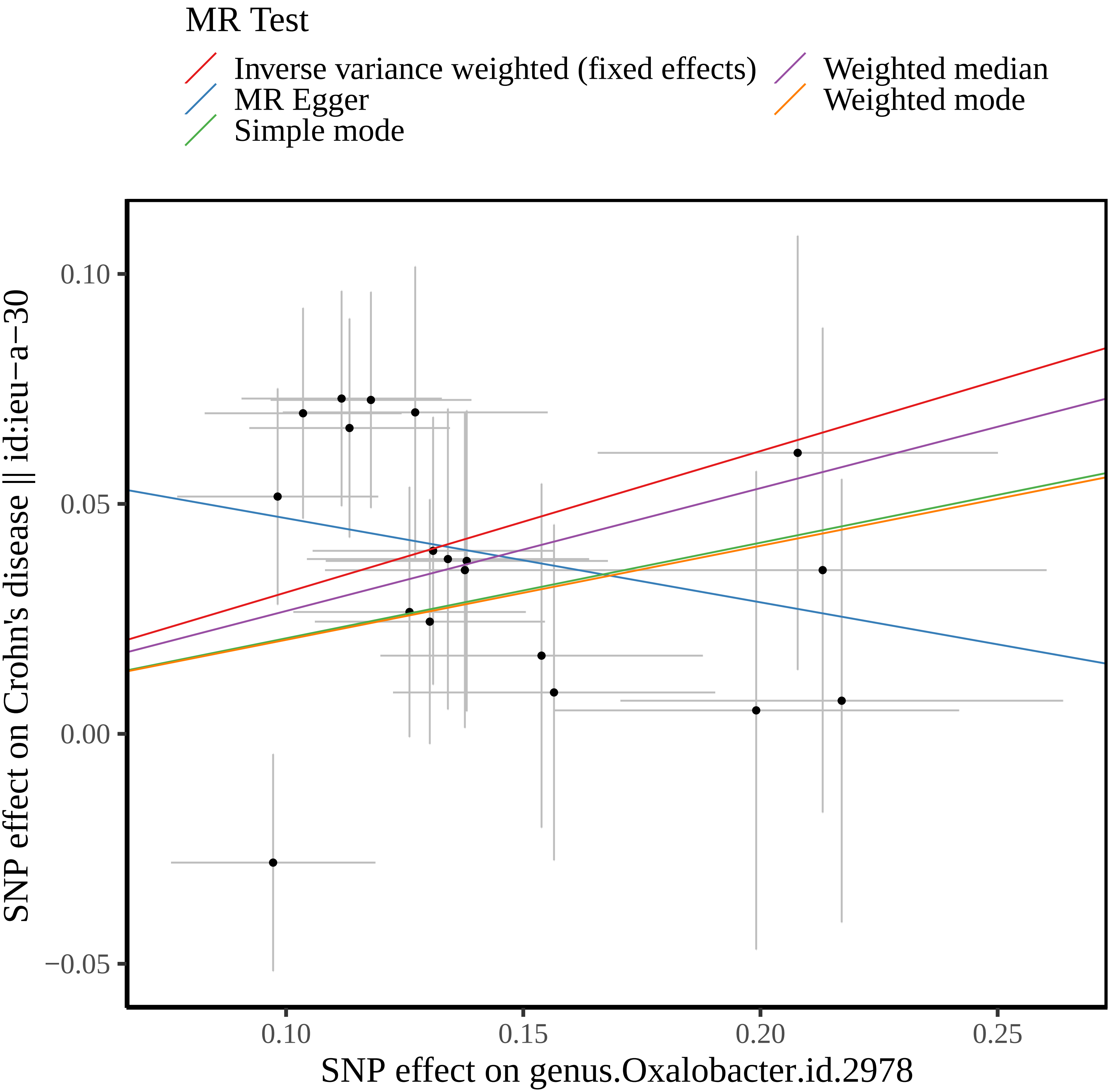

MR Test
Inverse variance weighted (fixed effects)
MR Egger
Simple mode
Weighted median
Weighted mode
SNP effect on Crohn's disease || id:ieu−a−30
0.10
0.05
0.00
−0.05
0.10
0.15
0.20
0.25
SNP effect on genus.Oxalobacter.id.2978

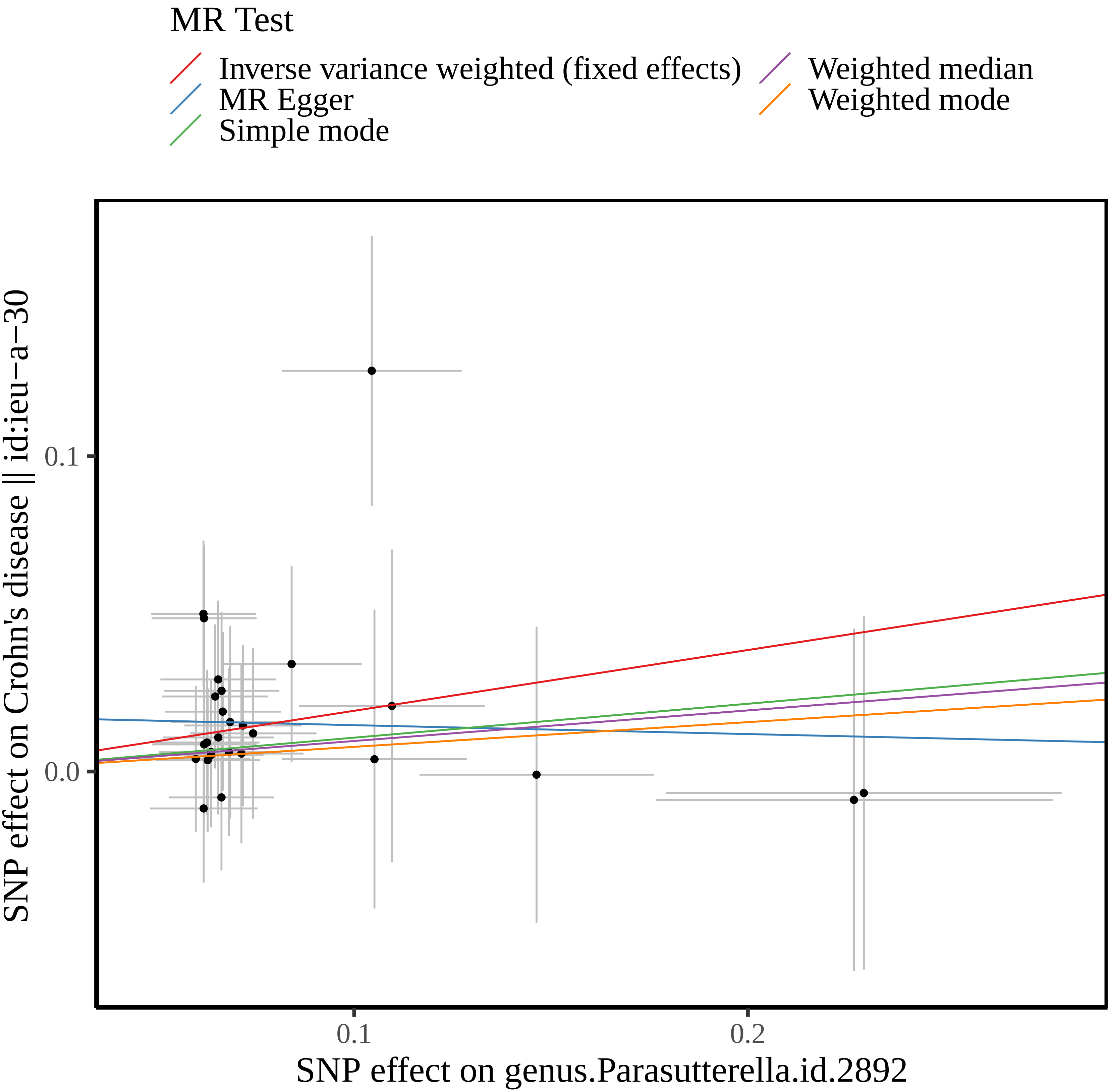

MR Test
Inverse variance weighted (fixed effects)
MR Egger
Simple mode
Weighted median
Weighted mode
SNP effect on Crohn's disease || id:ieu−a−30
0.1
0.0
0.1
0.2
SNP effect on genus.Parasutterella.id.2892

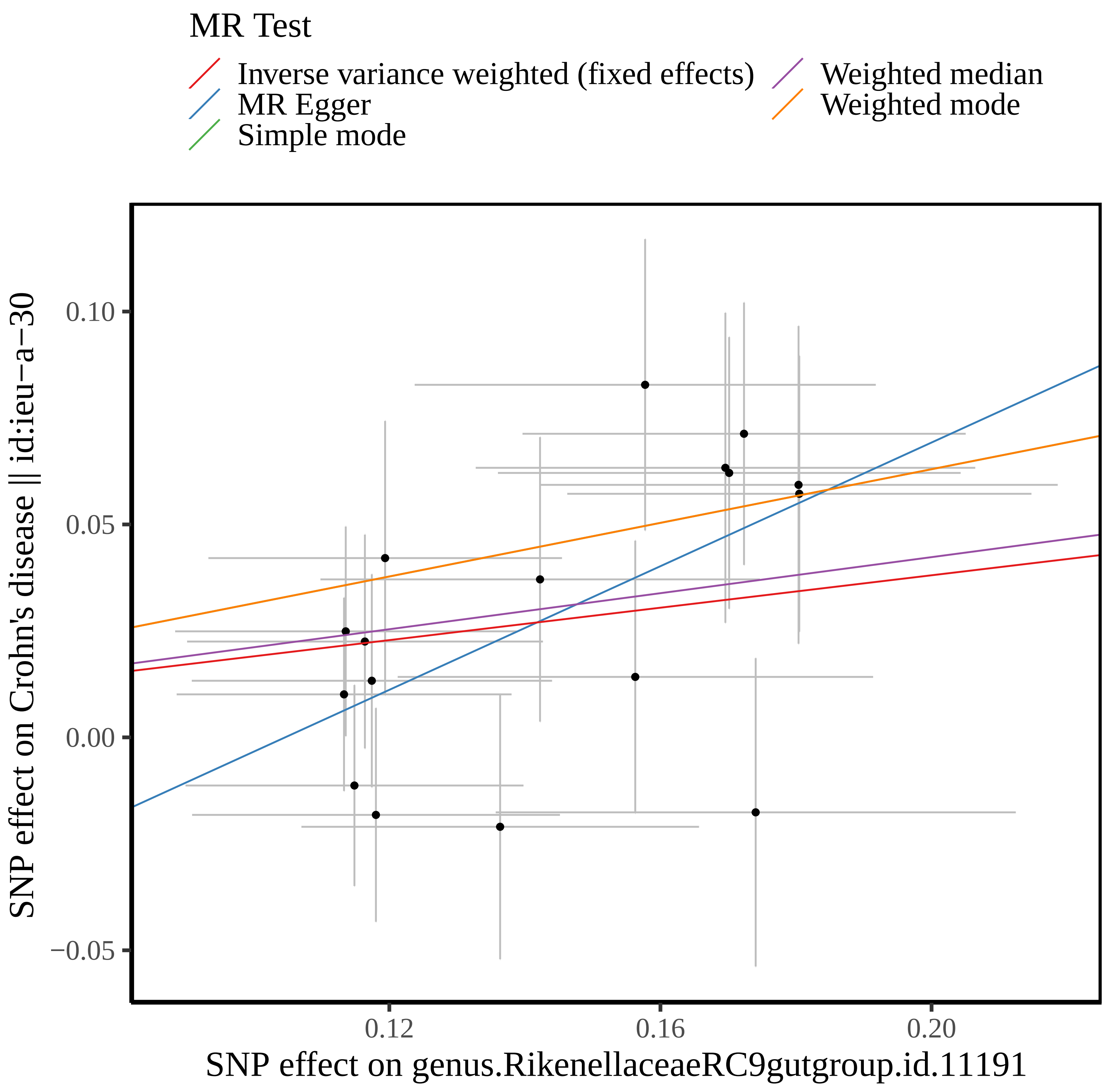
MR Test
Inverse variance weighted (fixed effects)
MR Egger
Simple mode
Weighted median
Weighted mode
0.10
0.05
0.00
−0.05
SNP effect on Crohn's disease || id:ieu−a−30
0.12
0.16
0.20
SNP effect on genus.RikenellaceaeRC9gutgroup.id.11191

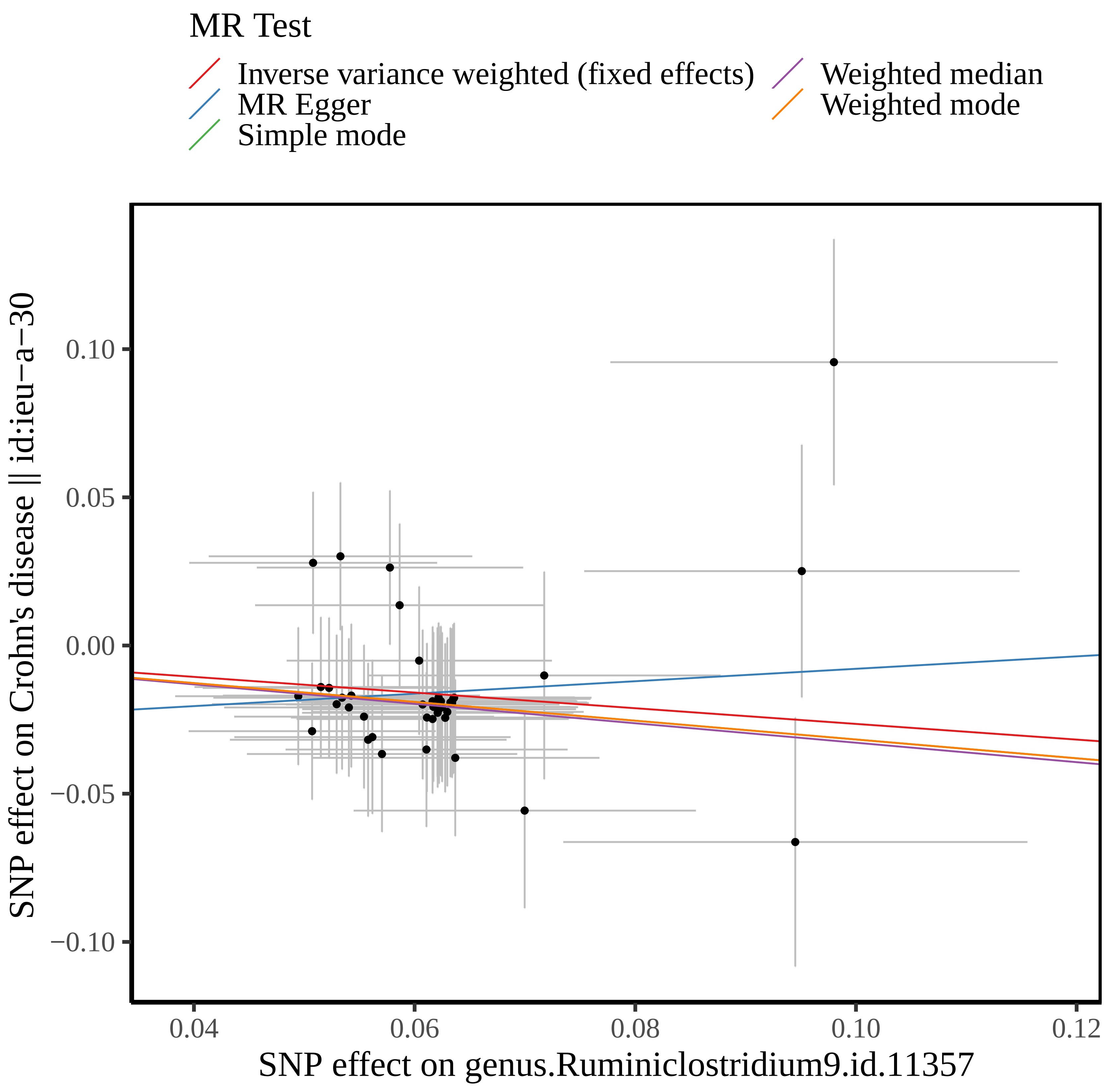
MR Test
Inverse variance weighted (fixed effects)
MR Egger
Simple mode
Weighted median
Weighted mode
SNP effect on Crohn's disease || id:ieu−a−30
0.10
0.05
0.00
−0.05
−0.10
0.04
0.06
0.08
0.10
0.12
SNP effect on genus.Ruminiclostridium9.id.11357

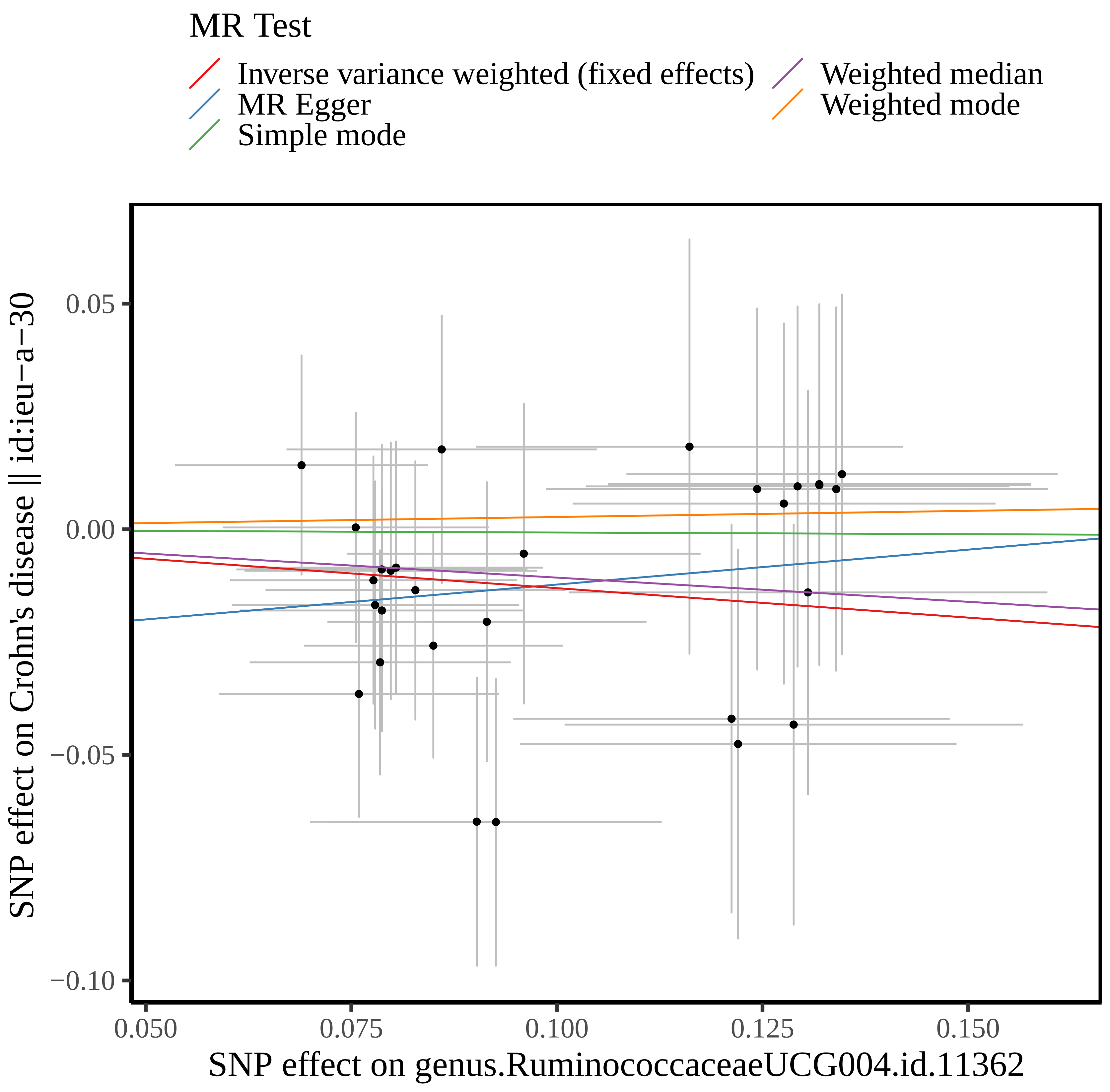
MR Test
Inverse variance weighted (fixed effects)
MR Egger
Simple mode
Weighted median
Weighted mode
SNP effect on Crohn's disease || id:ieu−a−30
0.05
0.00
−0.05
−0.10
0.050
0.075
0.100
0.125
0.150
SNP effect on genus.RuminococcaceaeUCG004.id.11362

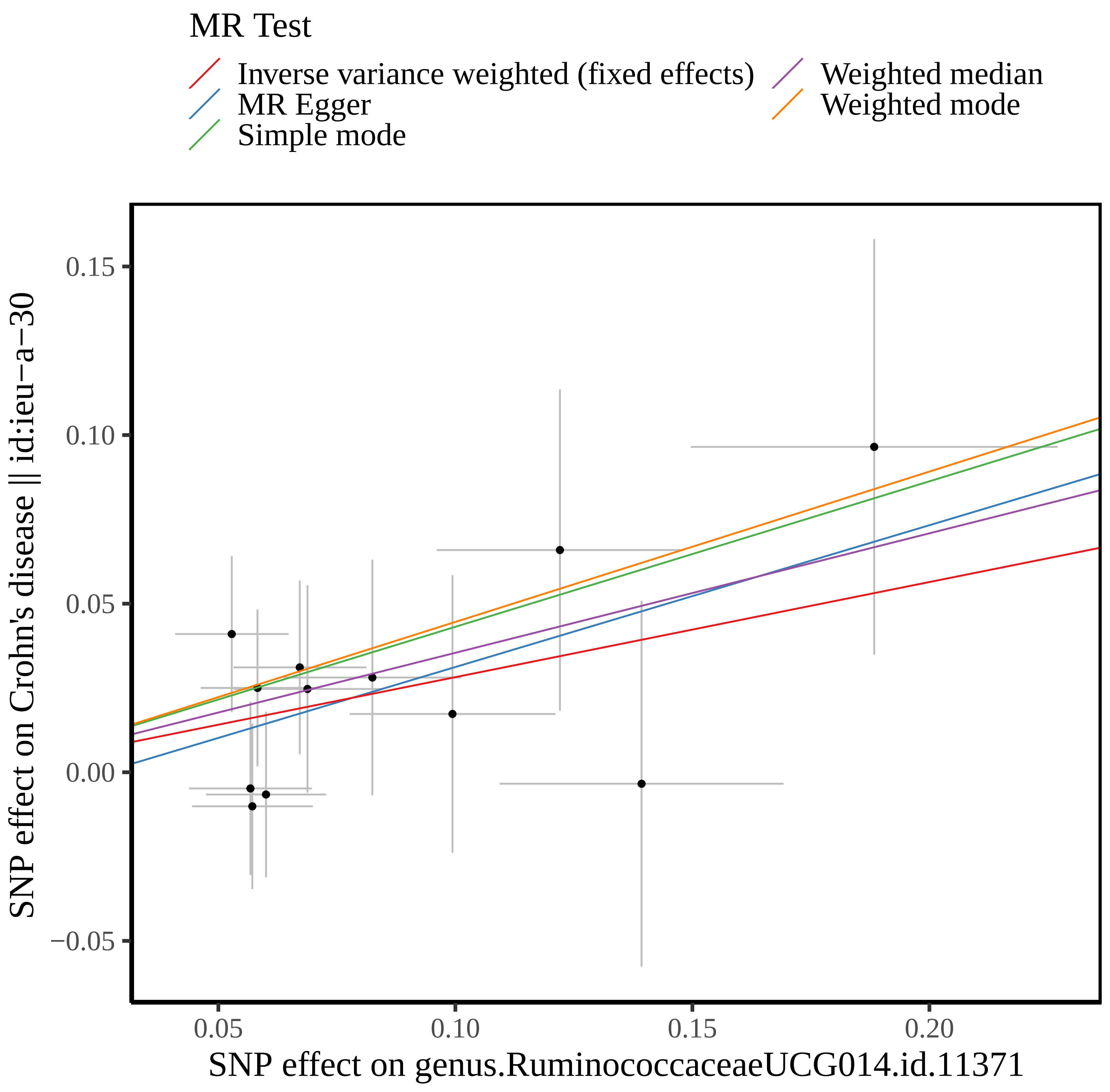
MR Test
Inverse variance weighted (fixed effects)
MR Egger
Simple mode
Weighted median
Weighted mode
0.15
0.10
0.05
0.00
−0.05
0.05
0.10
0.15
0.20
SNP effect on Crohn's disease || id:ieu−a−30
SNP effect on genus.RuminococcaceaeUCG014.id.11371

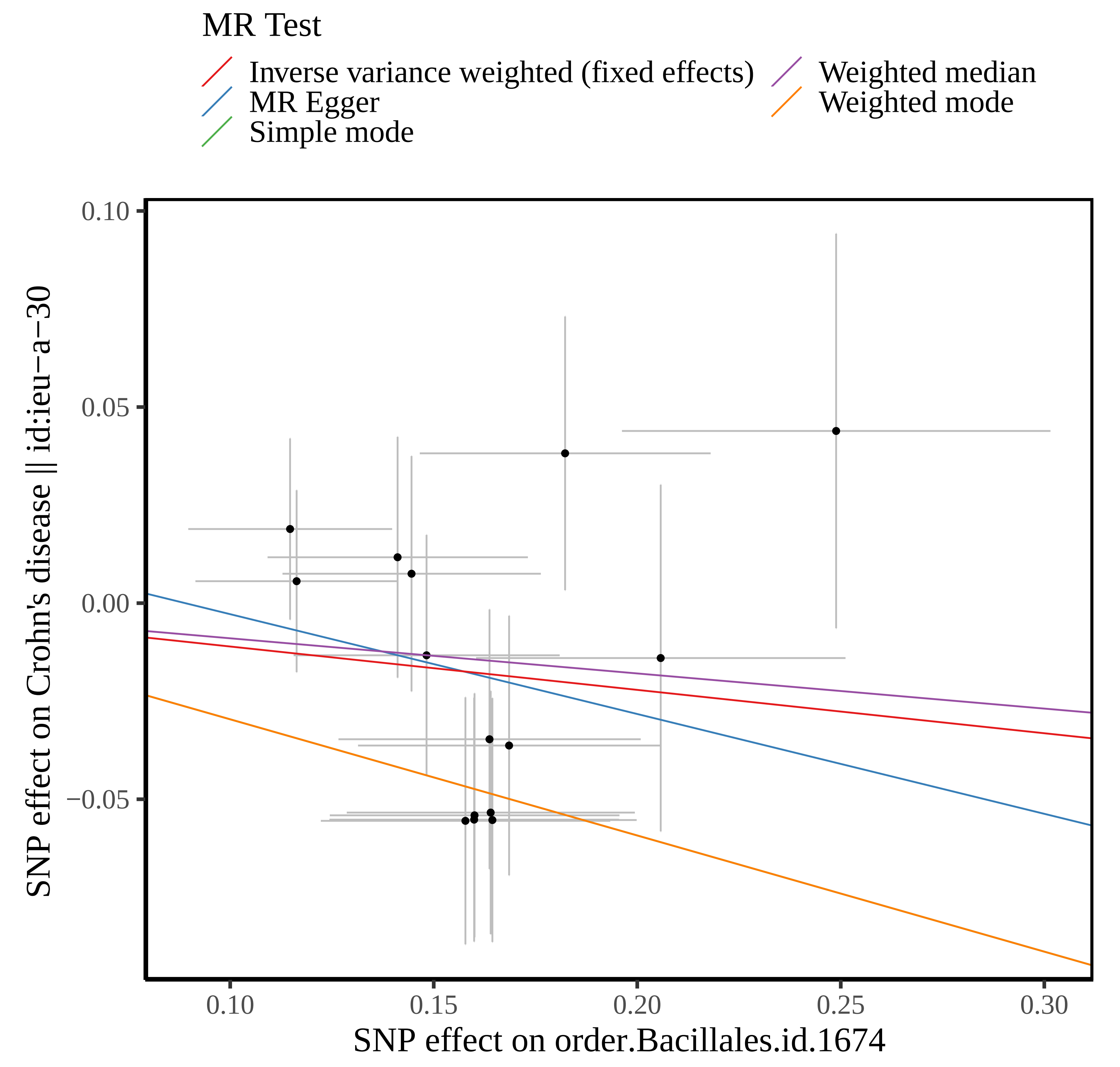

MR Test
Inverse variance weighted (fixed effects)
MR Egger
Simple mode
Weighted median
Weighted mode
0.10
0.05
0.00
−0.05
0.10
0.15
0.20
0.25
0.30
SNP effect on Crohn's disease || id:ieu−a−30
SNP effect on order.Bacillales.id.1674

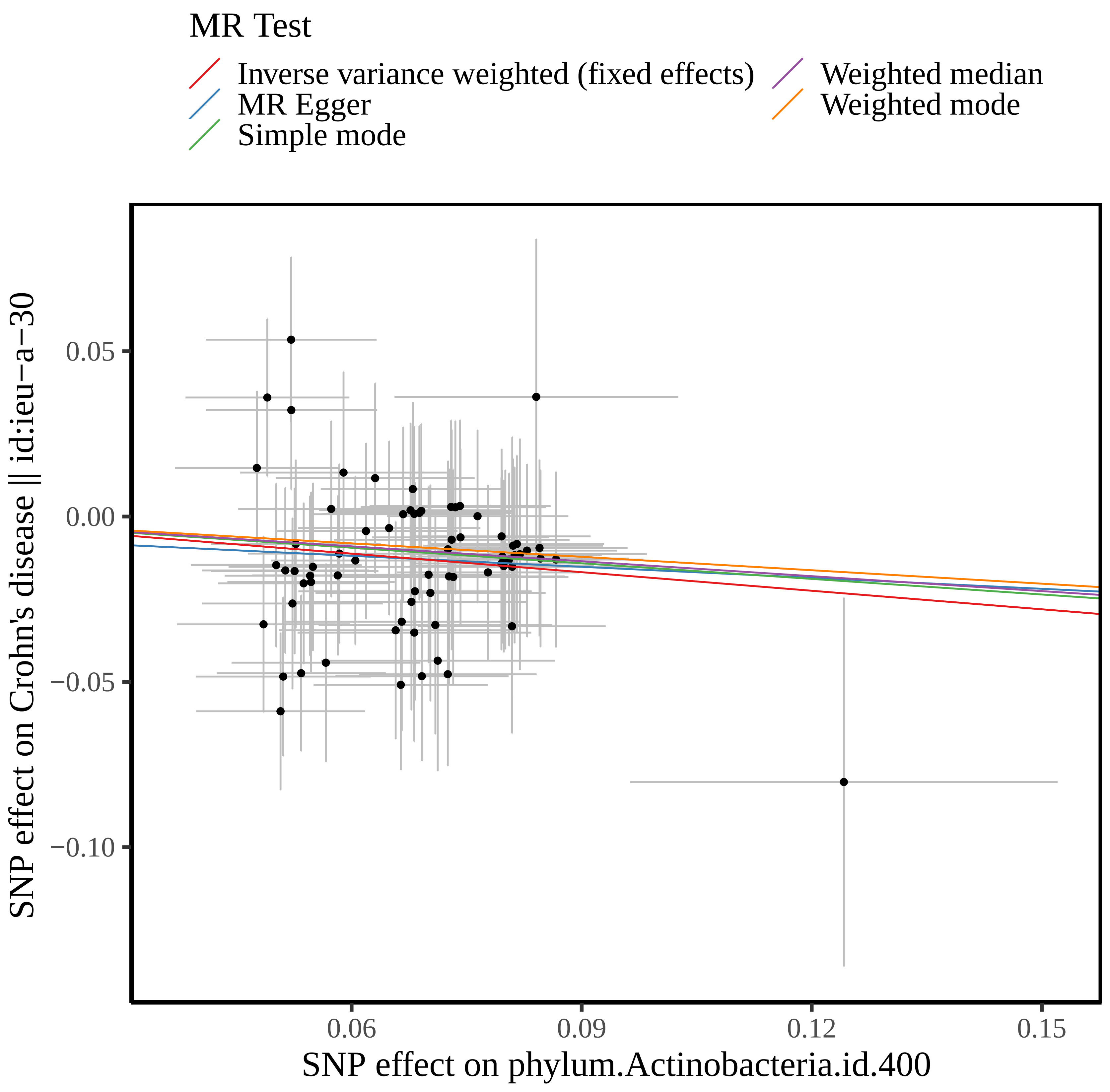
MR Test
Inverse variance weighted (fixed effects)
MR Egger
Simple mode
Weighted median
Weighted mode
SNP effect on Crohn's disease || id:ieu−a−30
0.05
0.00
−0.05
−0.10
0.06
0.09
0.12
0.15
SNP effect on phylum.Actinobacteria.id.400
