# Supplementary material for: Mendelian randomization integrated with multi-omics analysis identifies TNIK as a key gene in gut microbiota-induced IBD development
Source: Front Immunol. 2025 Nov 18;16:1678444. doi: 10.3389/fimmu.2025.1678444 (PMC12669205; doi:10.3389/fimmu.2025.1678444)
Supplement: Supplementary file 3 [file DataSheet3.pdf]

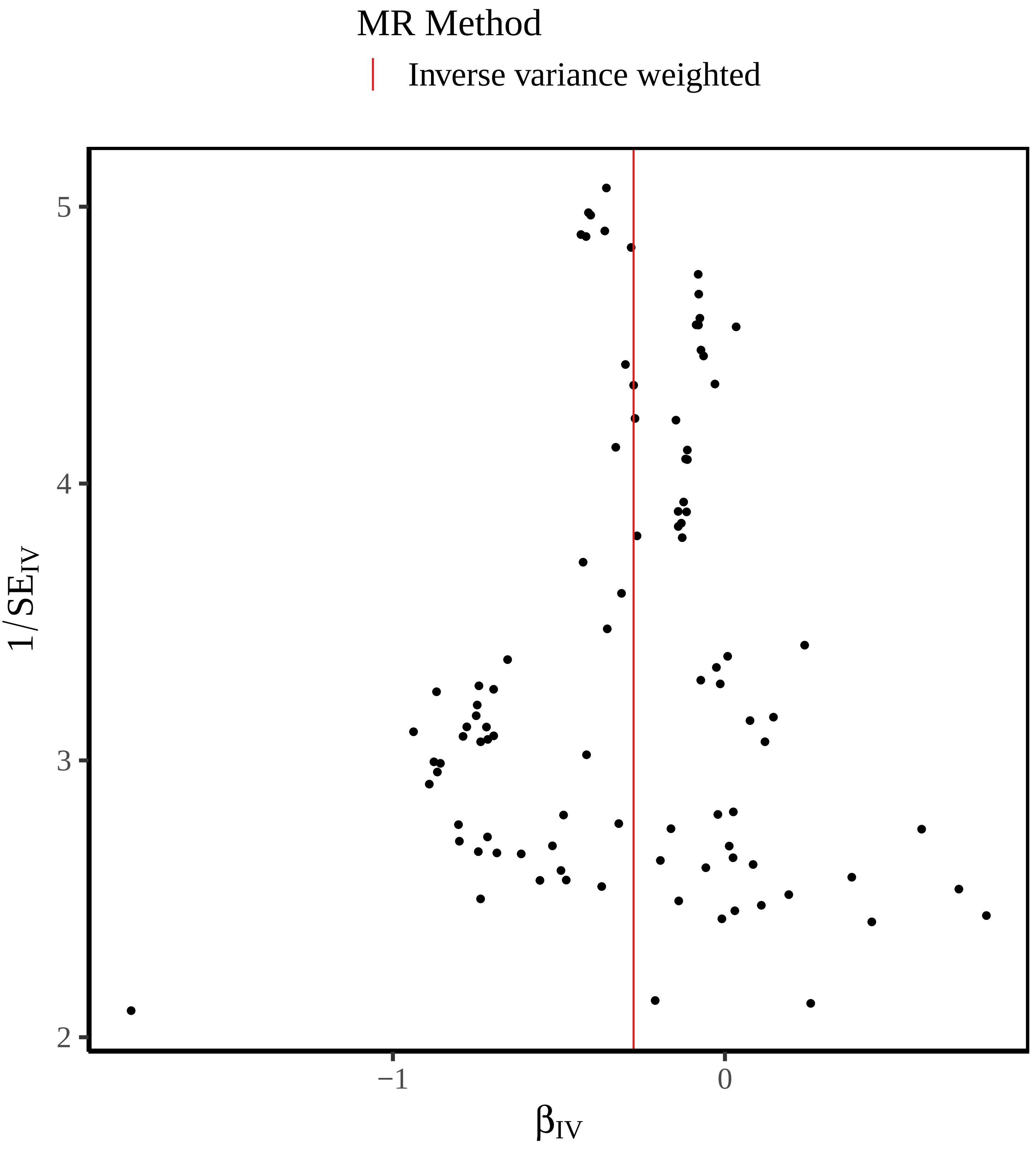
MR Method
Inverse variance weighted
5
4
3
2
1/SE_IV
−1
0
β_IV

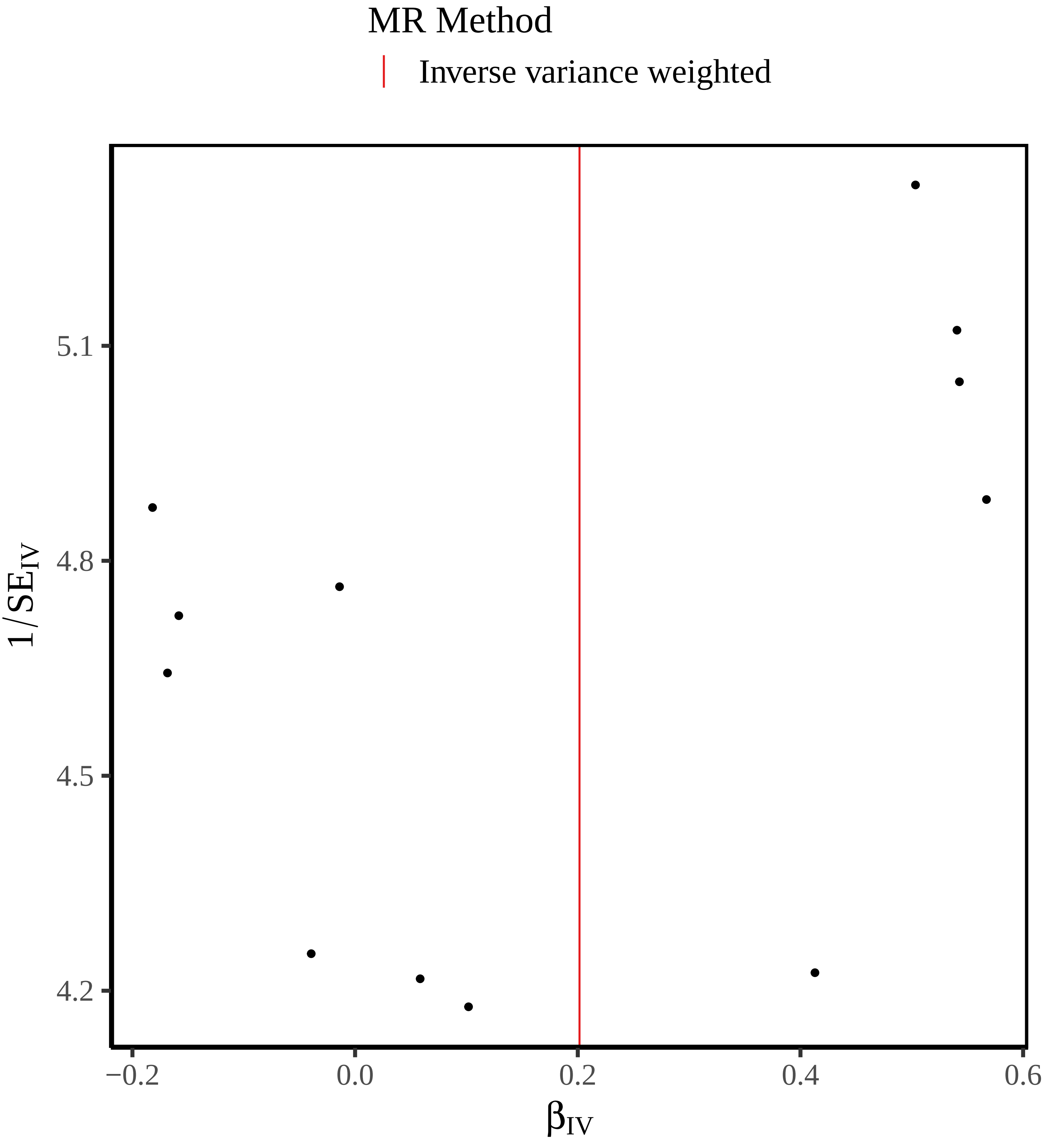

MR Method
Inverse variance weighted
5.1
4.8
4.5
4.2
1/SE_IV
−0.2
0.0
0.2
0.4
0.6
β_IV

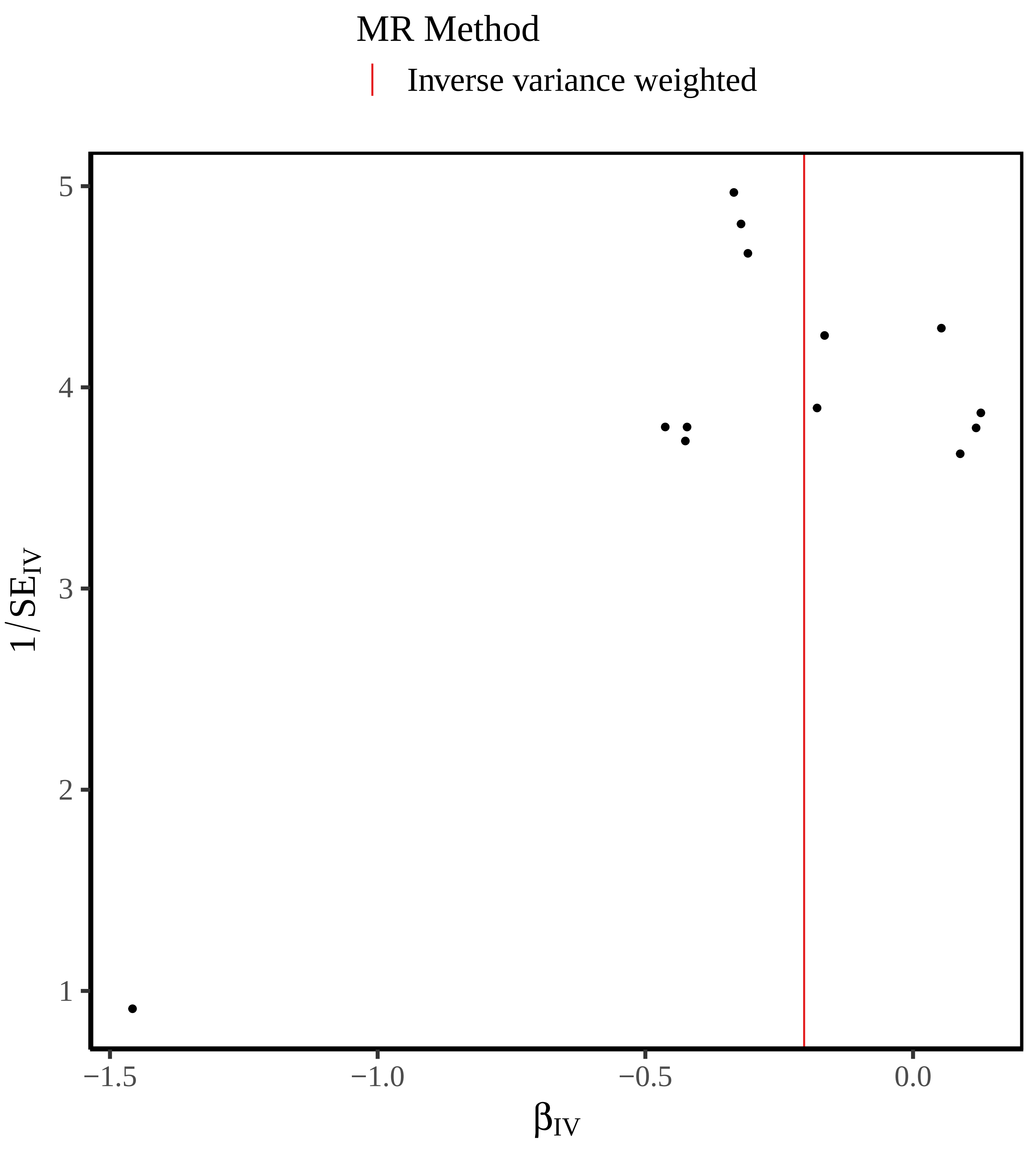
MR Method
Inverse variance weighted
1/SE_IV
β_IV
5
4
3
2
1
−1.5
−1.0
−0.5
0.0

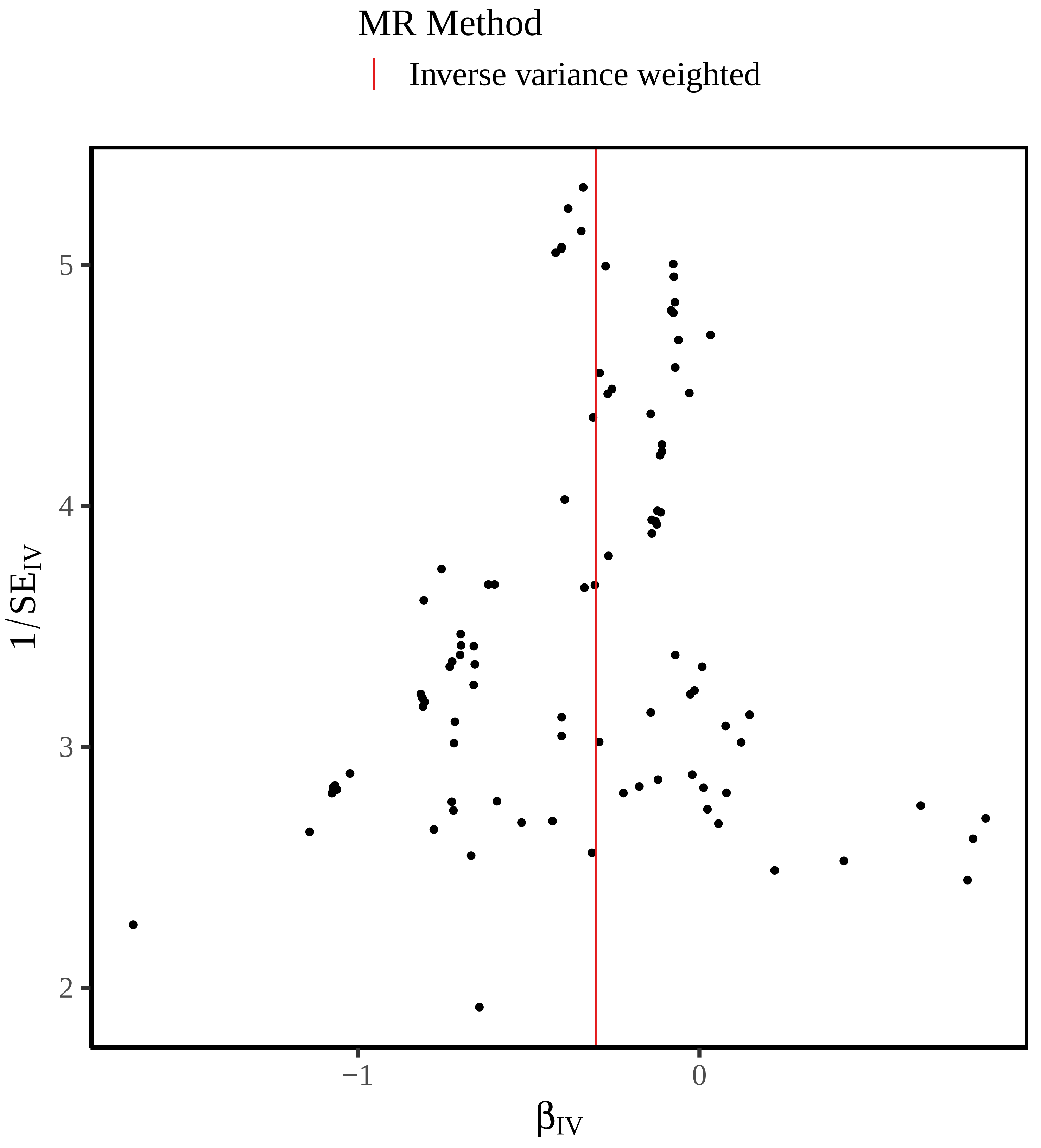
MR Method
Inverse variance weighted
1/SE$_{IV}$
5
4
3
2
−1
0
β$_{IV}$

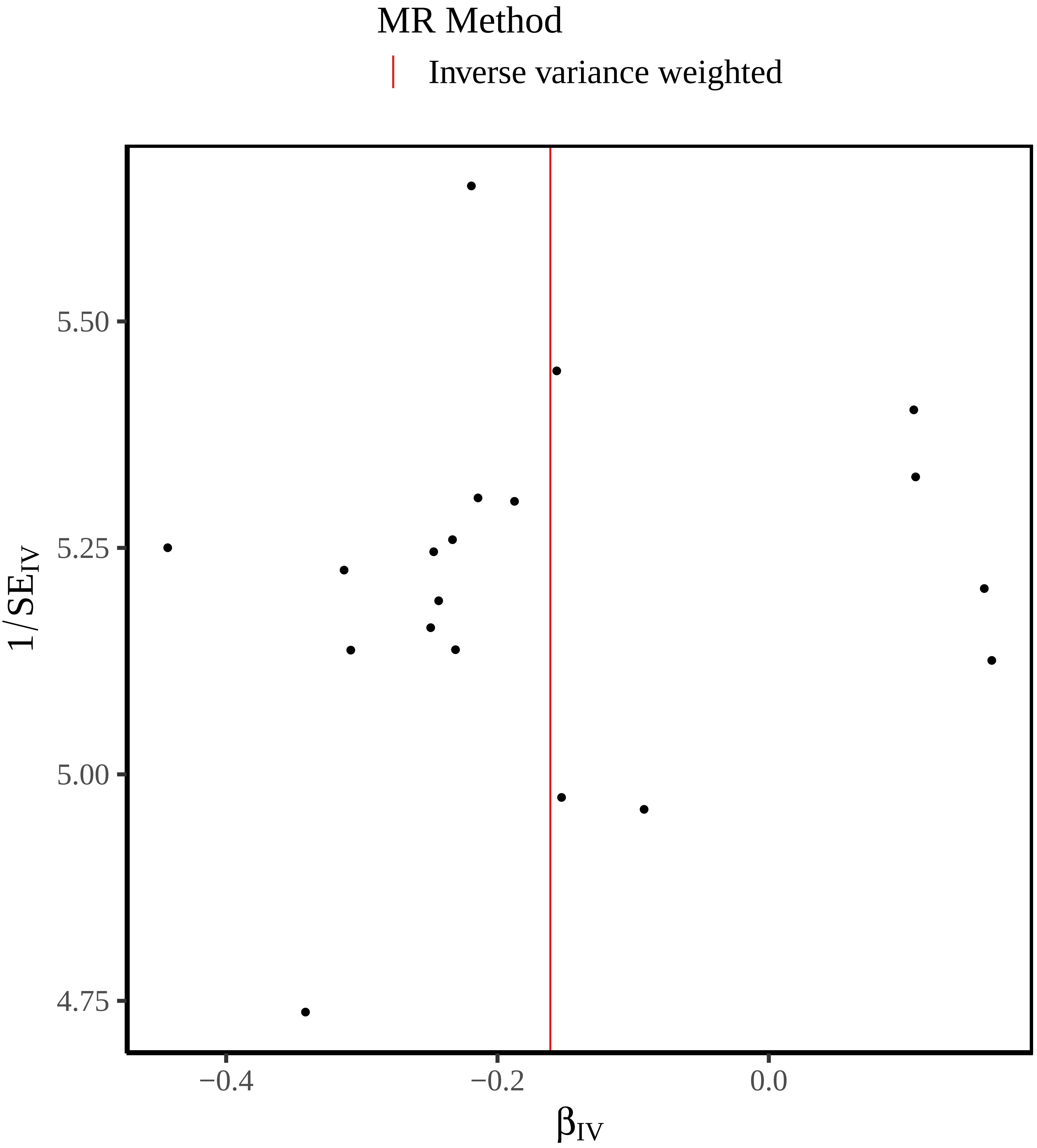
MR Method
Inverse variance weighted
5.50
5.25
5.00
4.75
$1/SE_{IV}$
−0.4
−0.2
0.0
$\beta_{IV}$

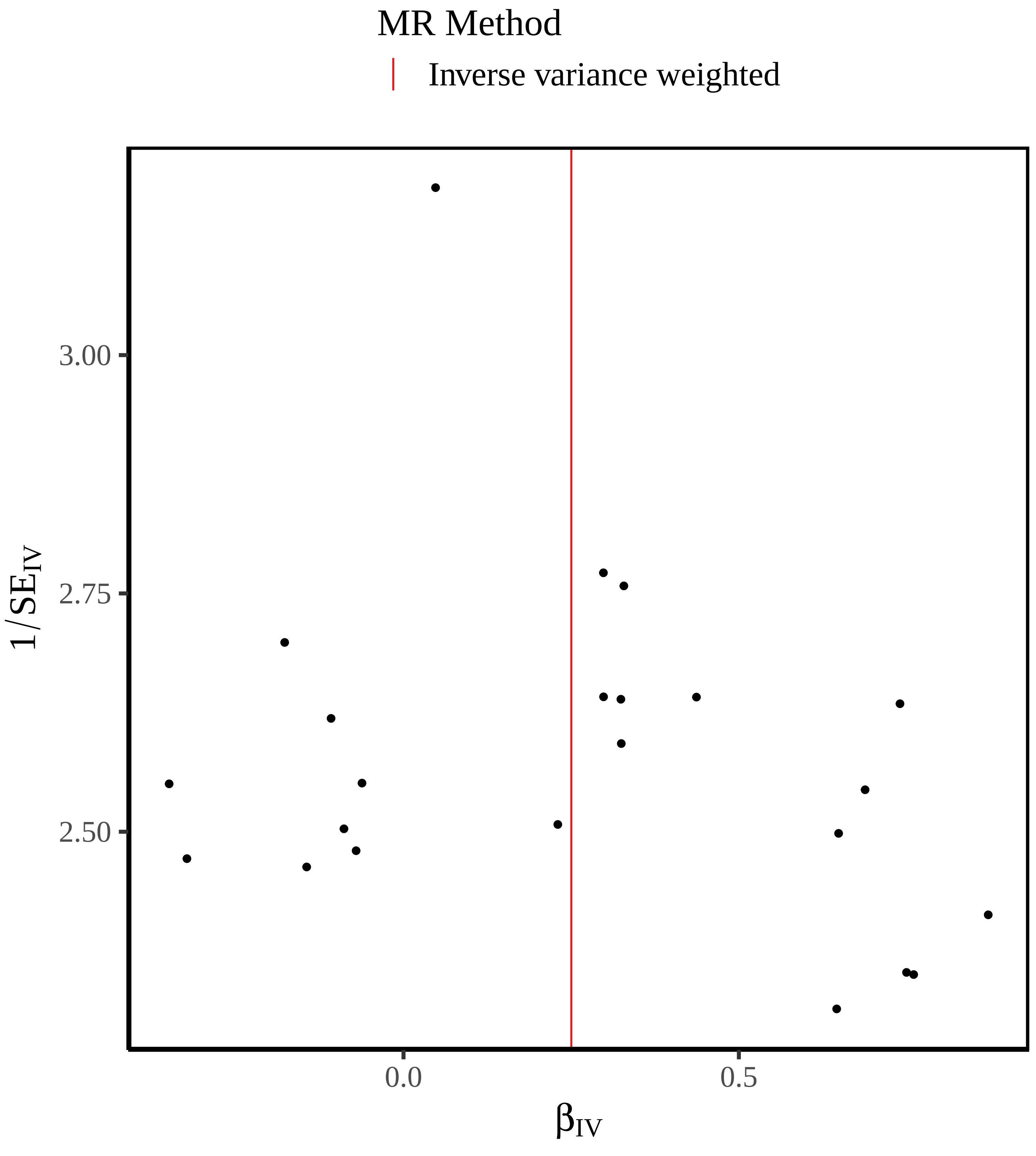

MR Method
Inverse variance weighted
3.00
2.75
2.50
0.0
0.5
$1/SE_{IV}$
$\beta_{IV}$

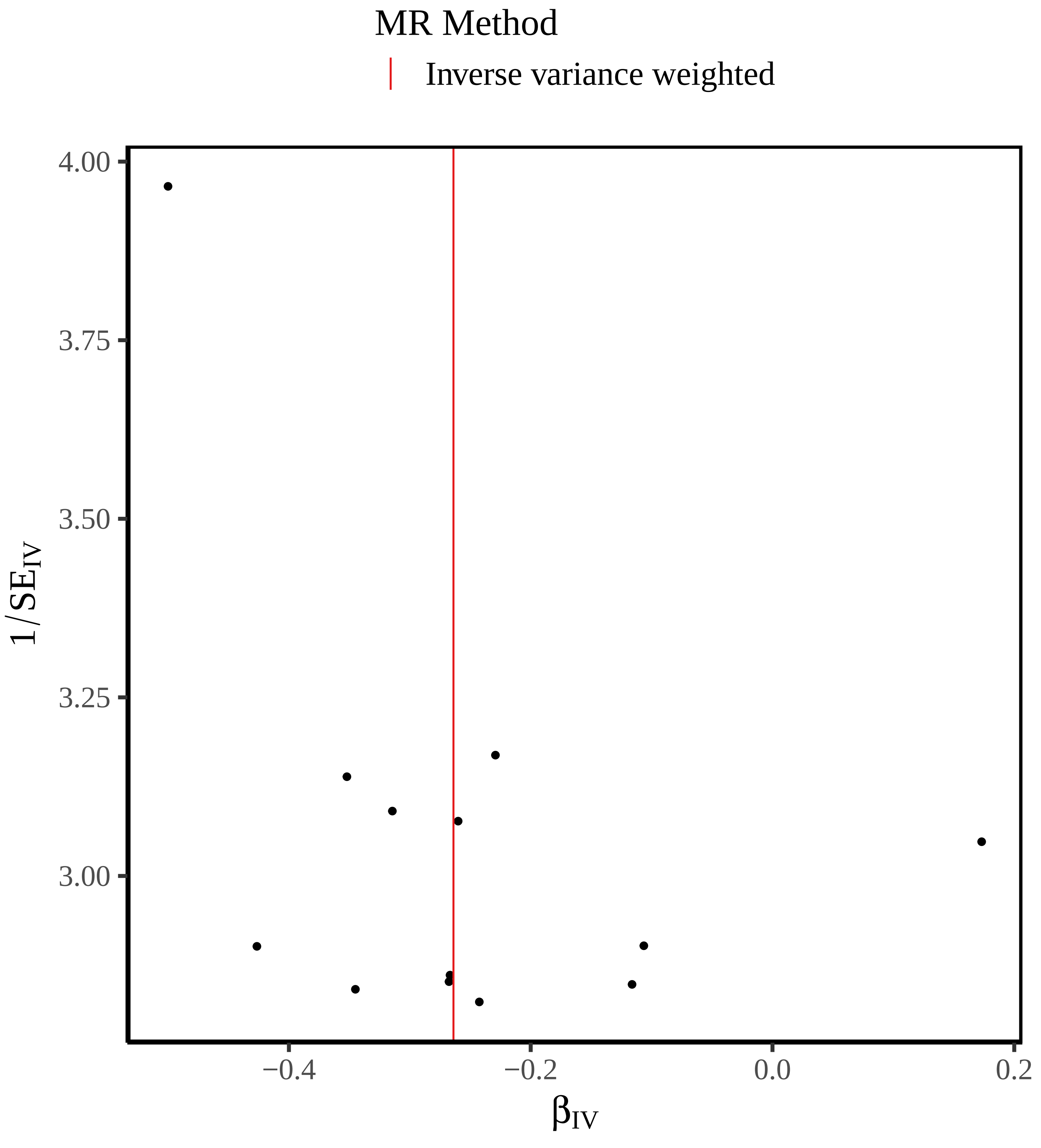
MR Method
Inverse variance weighted
4.00
3.75
3.50
3.25
3.00
1/SE_IV
−0.4
−0.2
0.0
0.2
β_IV

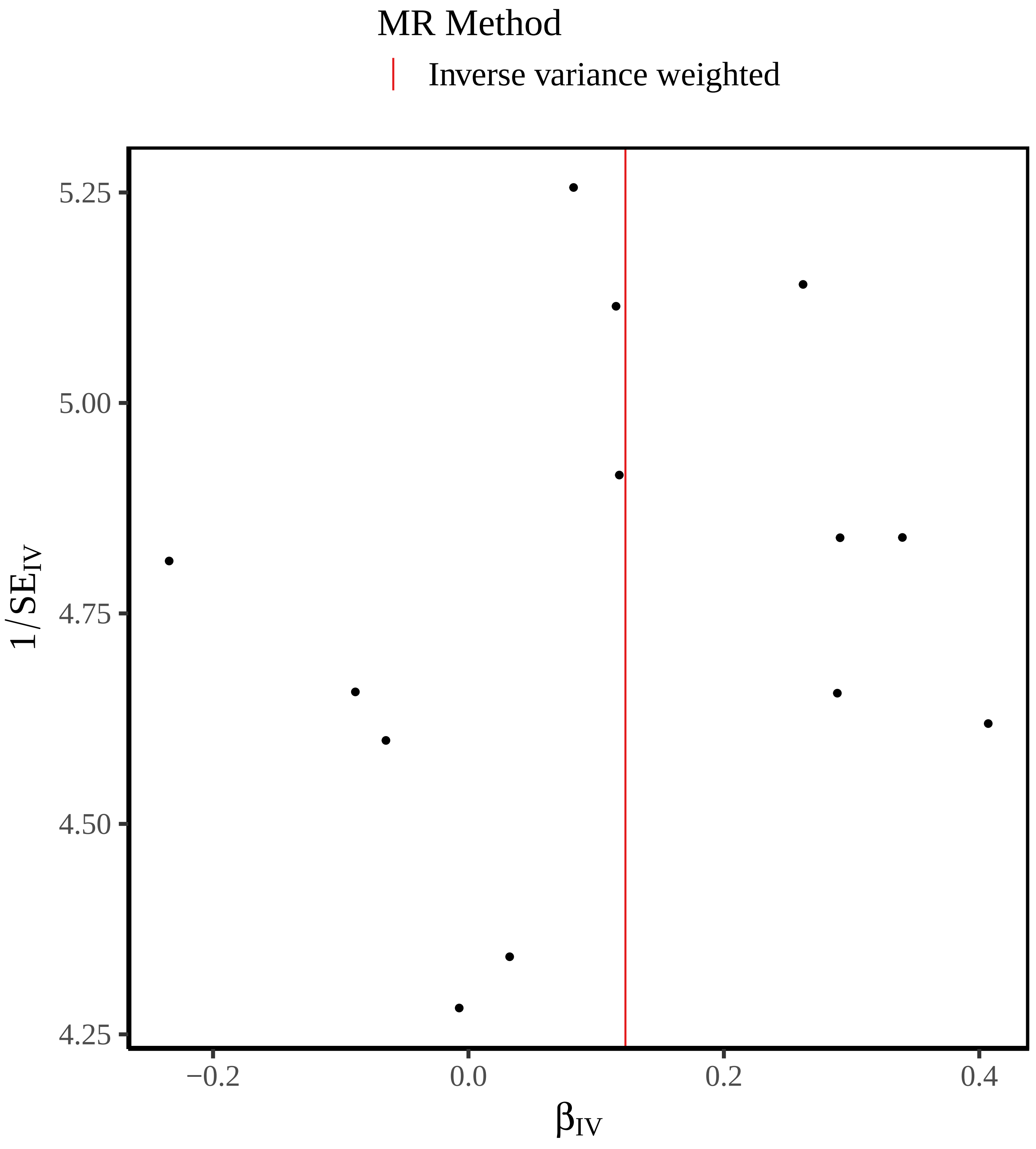

MR Method
Inverse variance weighted
5.25
5.00
4.75
4.50
4.25
$1/SE_{IV}$
−0.2
0.0
0.2
0.4
$\beta_{IV}$

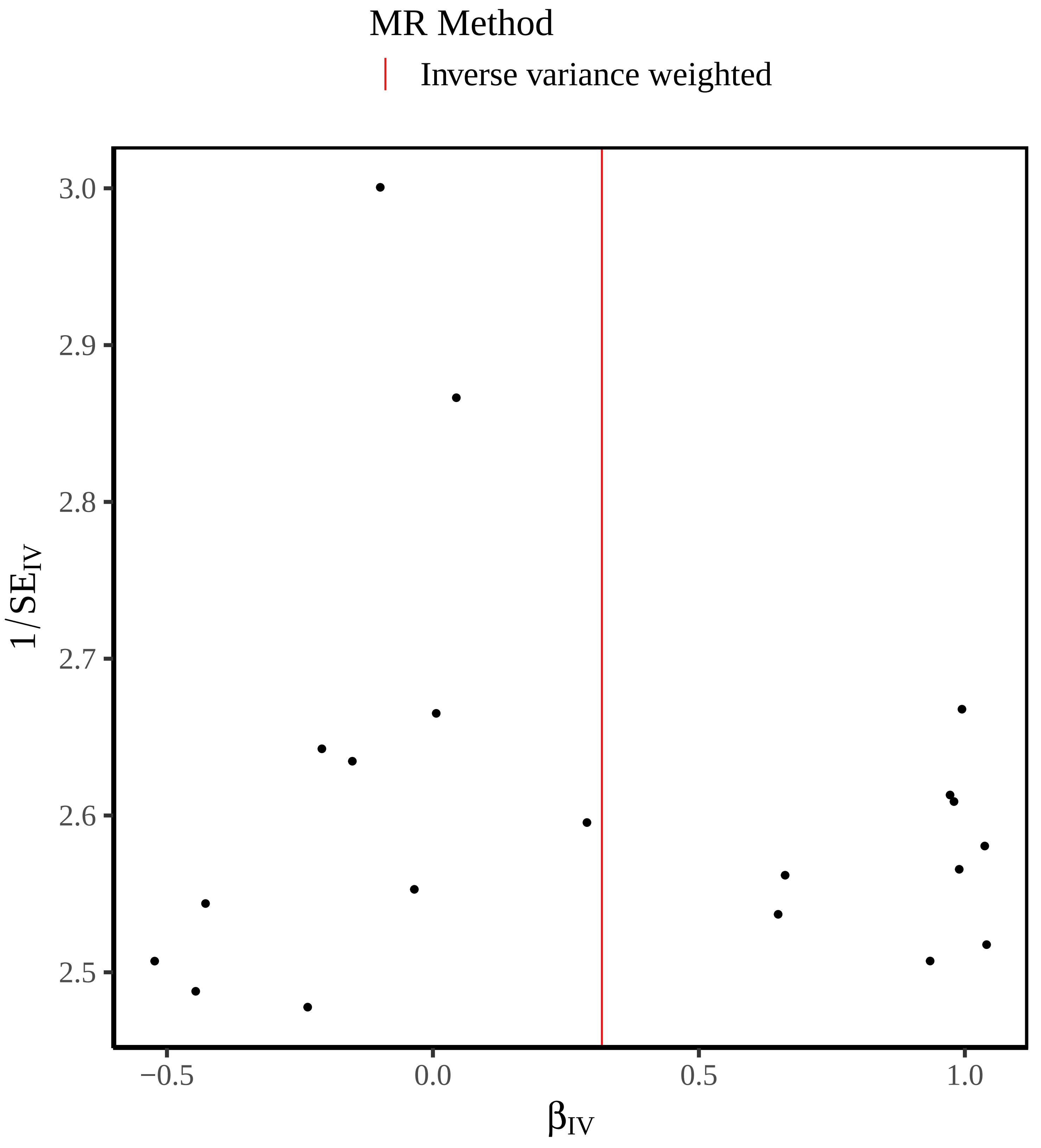

MR Method
Inverse variance weighted
3.0
2.9
2.8
2.7
2.6
2.5
$1/SE_{IV}$
−0.5
0.0
0.5
1.0
$\beta_{IV}$

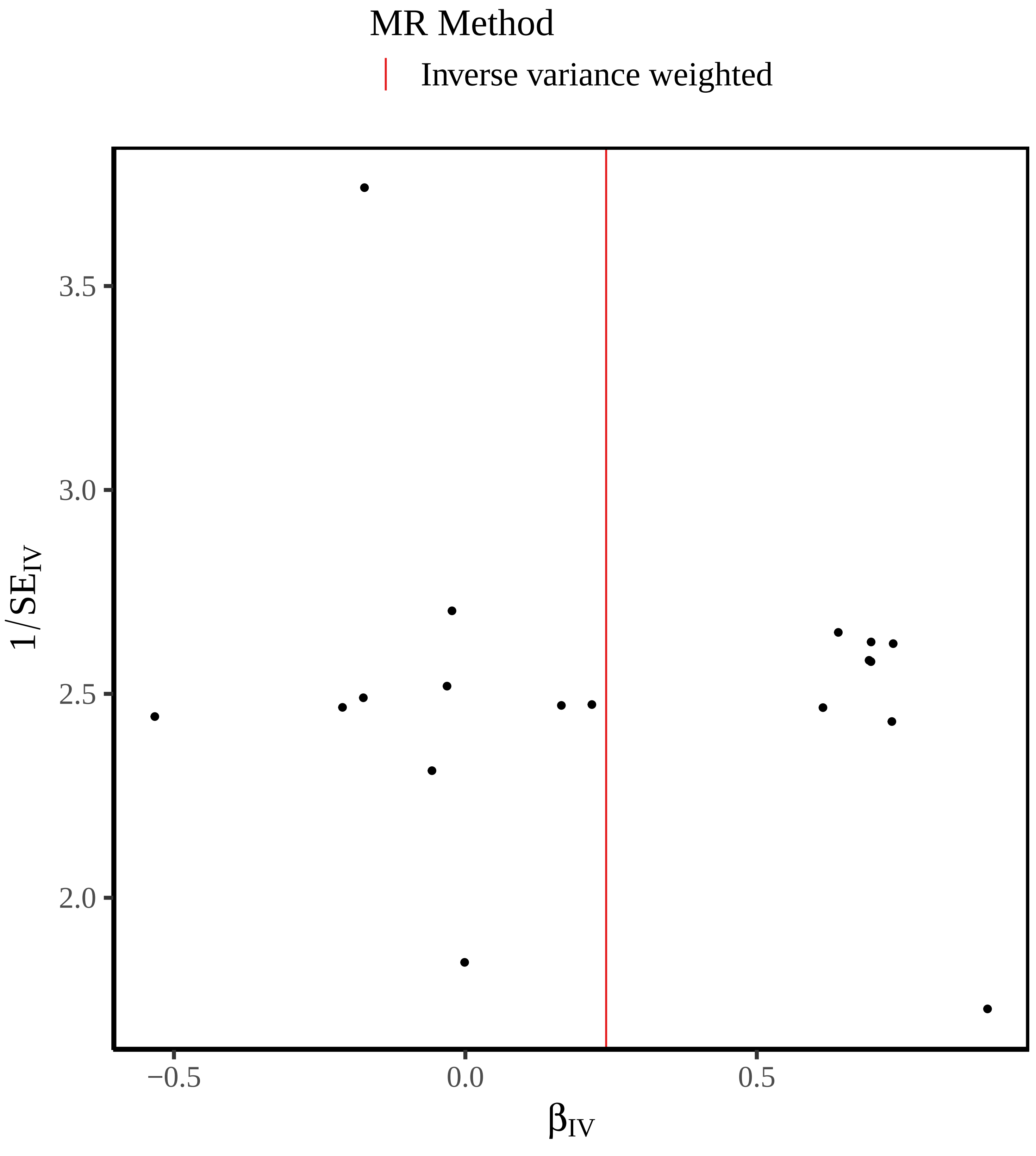

MR Method
Inverse variance weighted
3.5
3.0
2.5
2.0
1/SE_IV
−0.5
0.0
0.5
β_IV

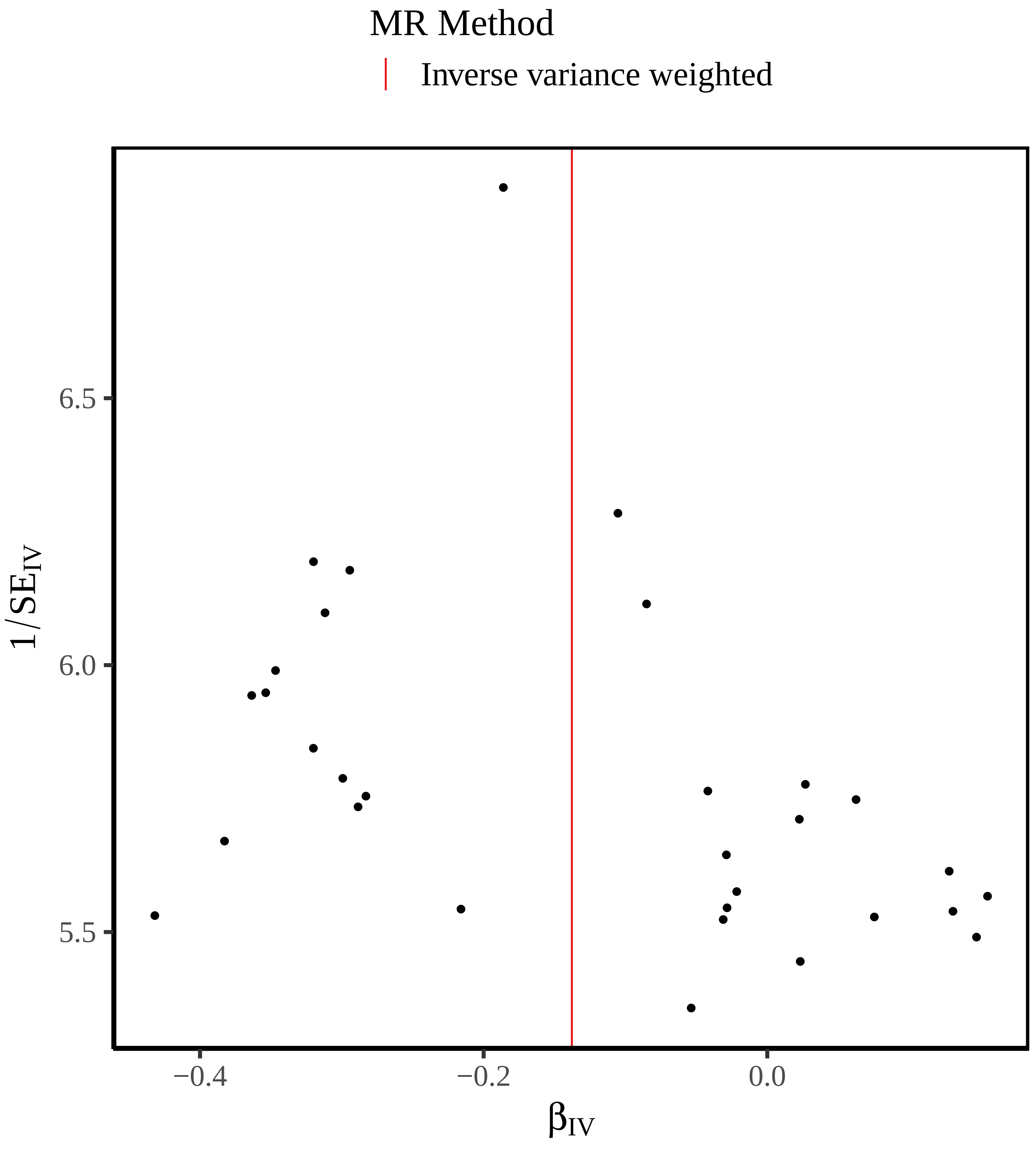

MR Method
Inverse variance weighted
6.5
6.0
5.5
$1/SE_{IV}$
−0.4
−0.2
0.0
$\beta_{IV}$

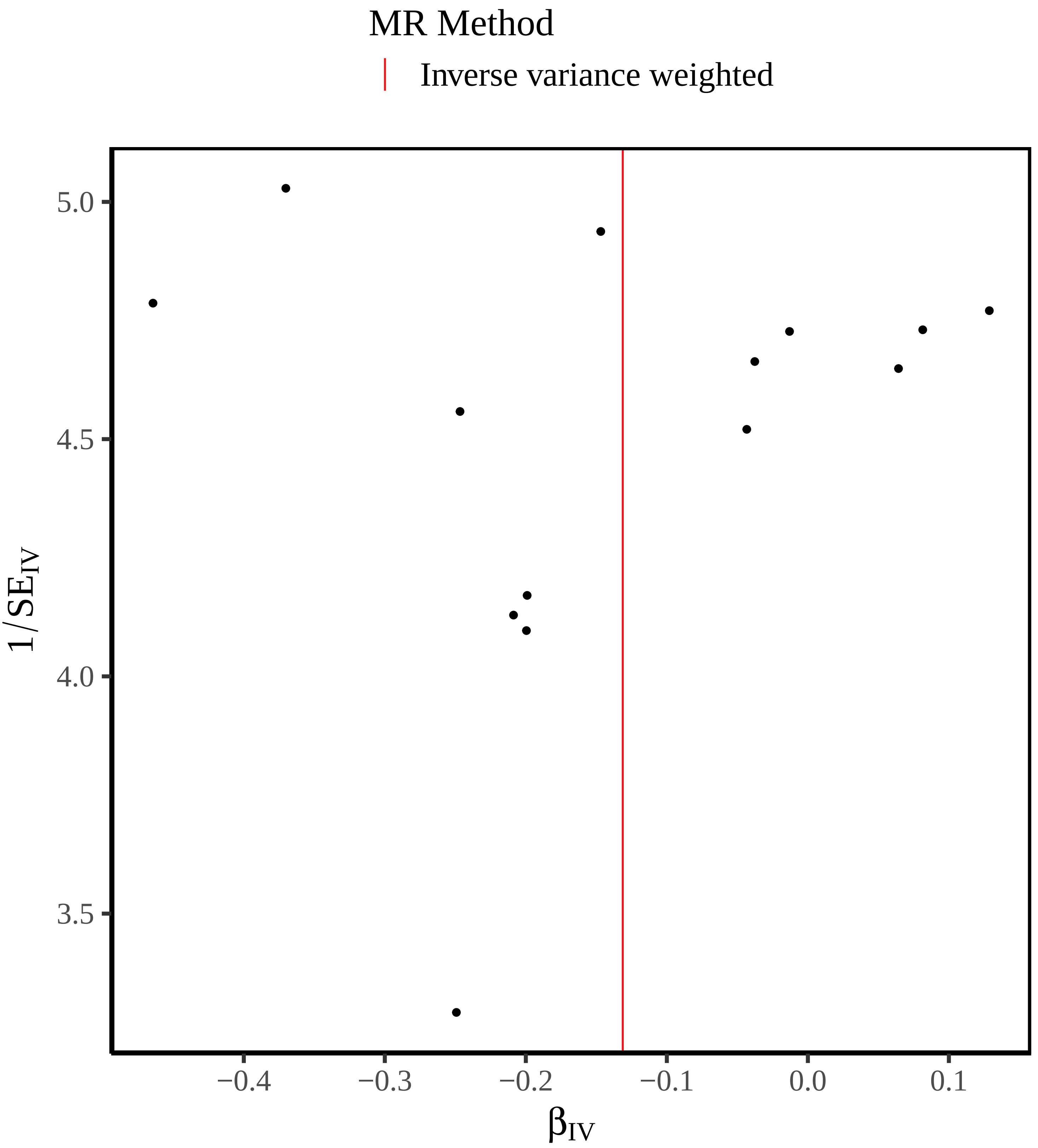

MR Method
Inverse variance weighted
5.0
4.5
4.0
3.5
1/SE_IV
−0.4
−0.3
−0.2
−0.1
0.0
0.1
β_IV

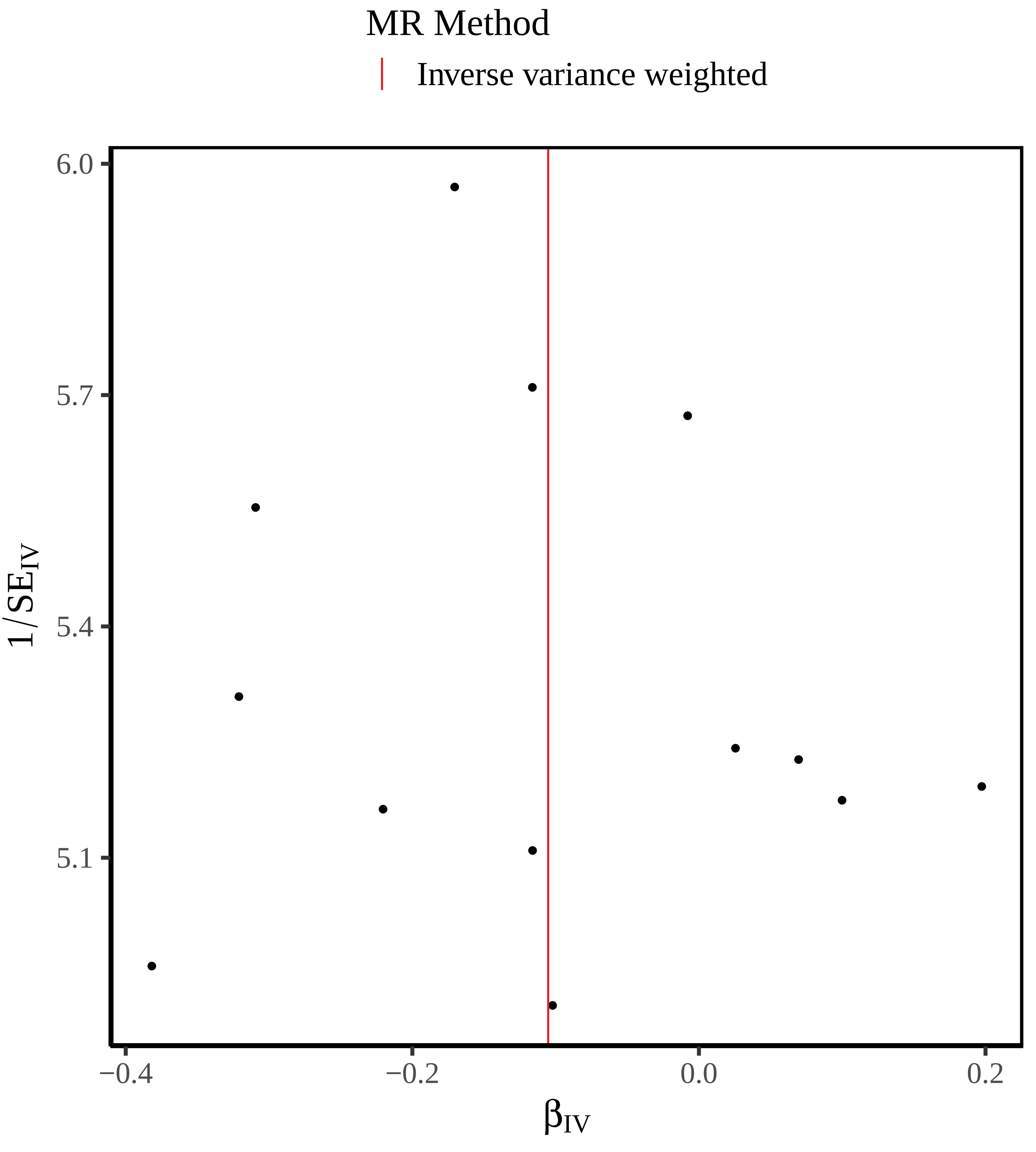
MR Method
Inverse variance weighted
6.0
5.7
5.4
5.1
1/SE_IV
−0.4
−0.2
0.0
0.2
β_IV

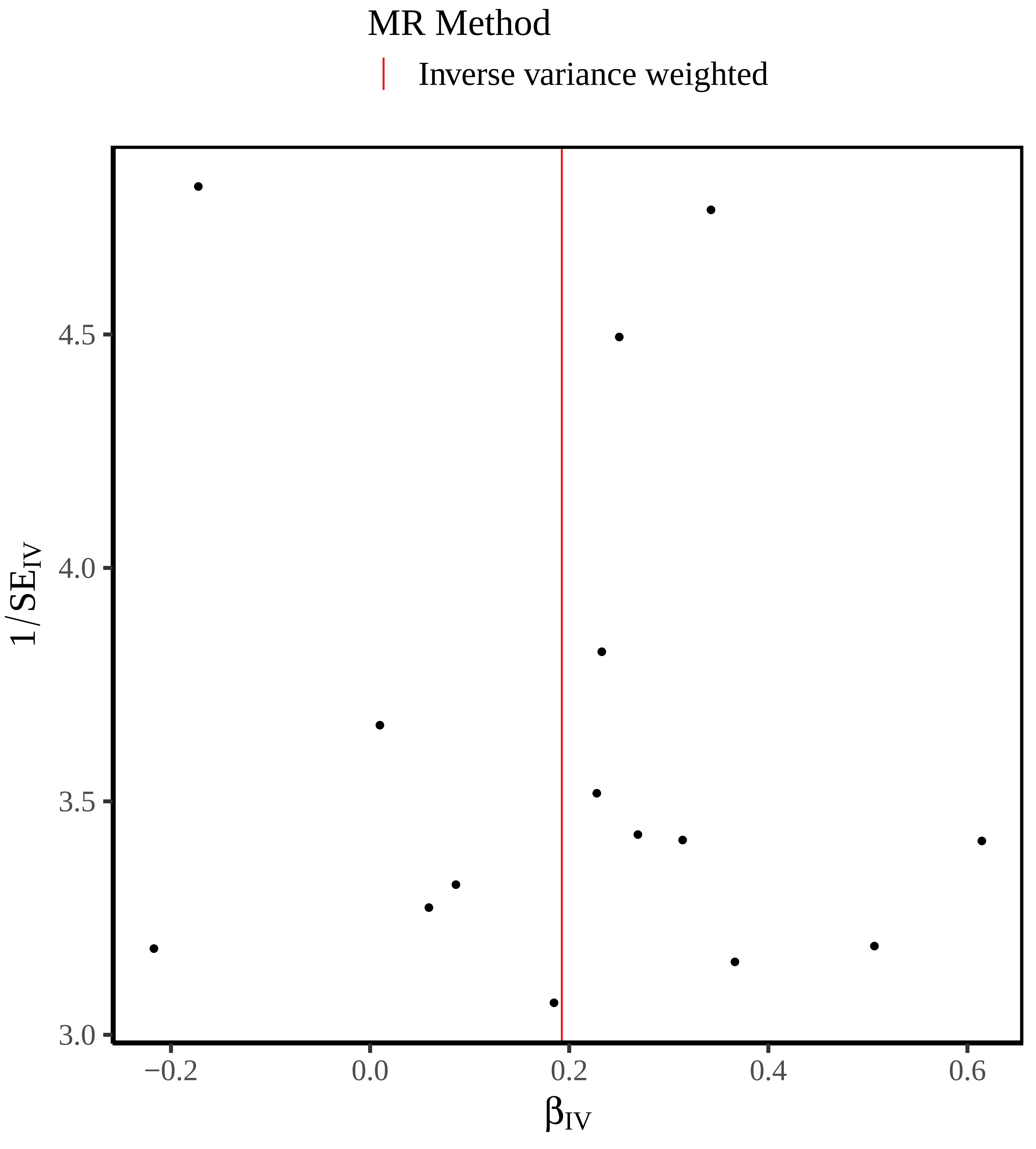
MR Method
Inverse variance weighted
4.5
4.0
3.5
3.0
$1/SE_{IV}$
−0.2
0.0
0.2
0.4
0.6
$\beta_{IV}$

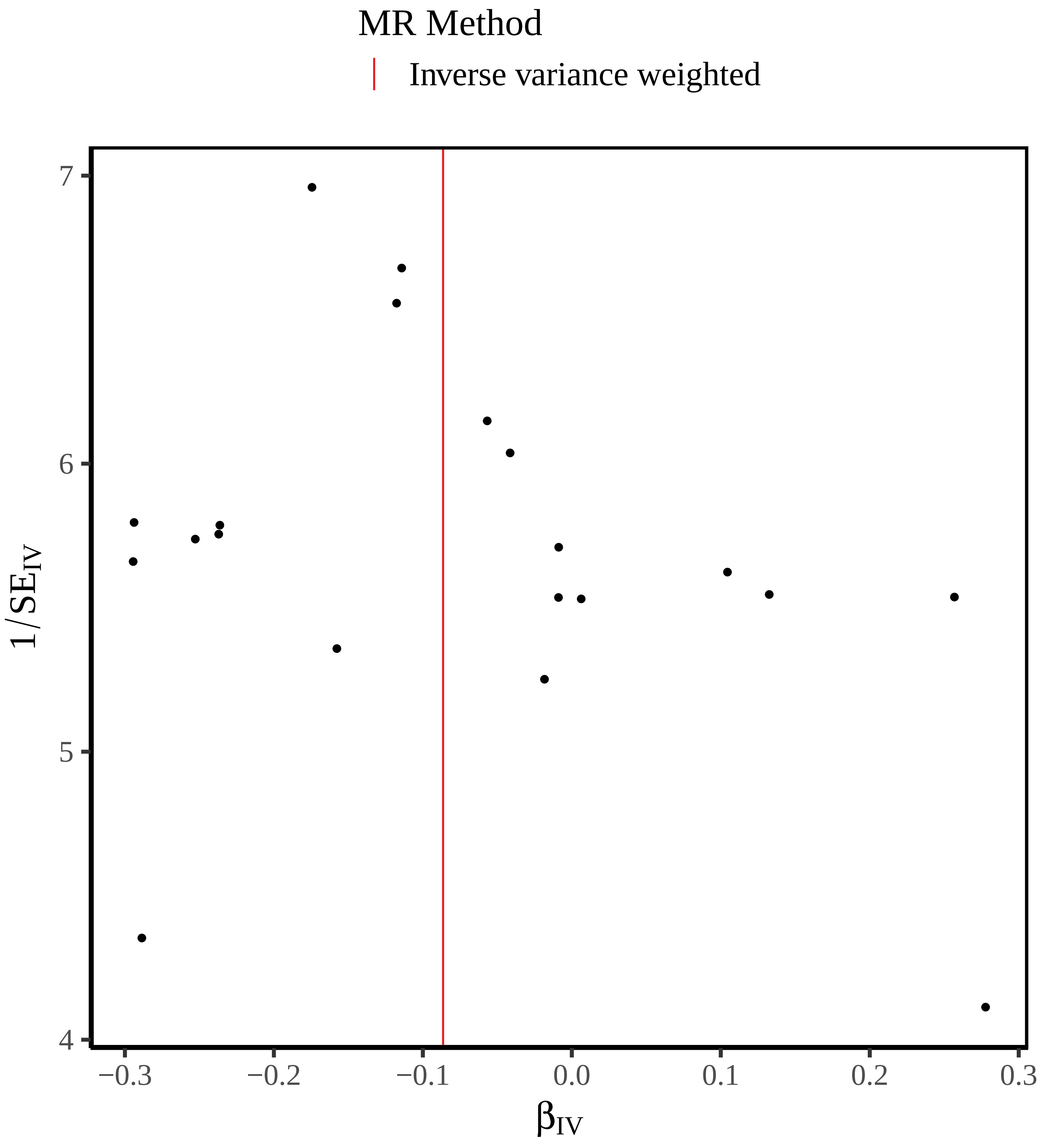

MR Method
Inverse variance weighted
7
6
5
4
1/SE$_{IV}$
−0.3
−0.2
−0.1
0.0
0.1
0.2
0.3
β$_{IV}$

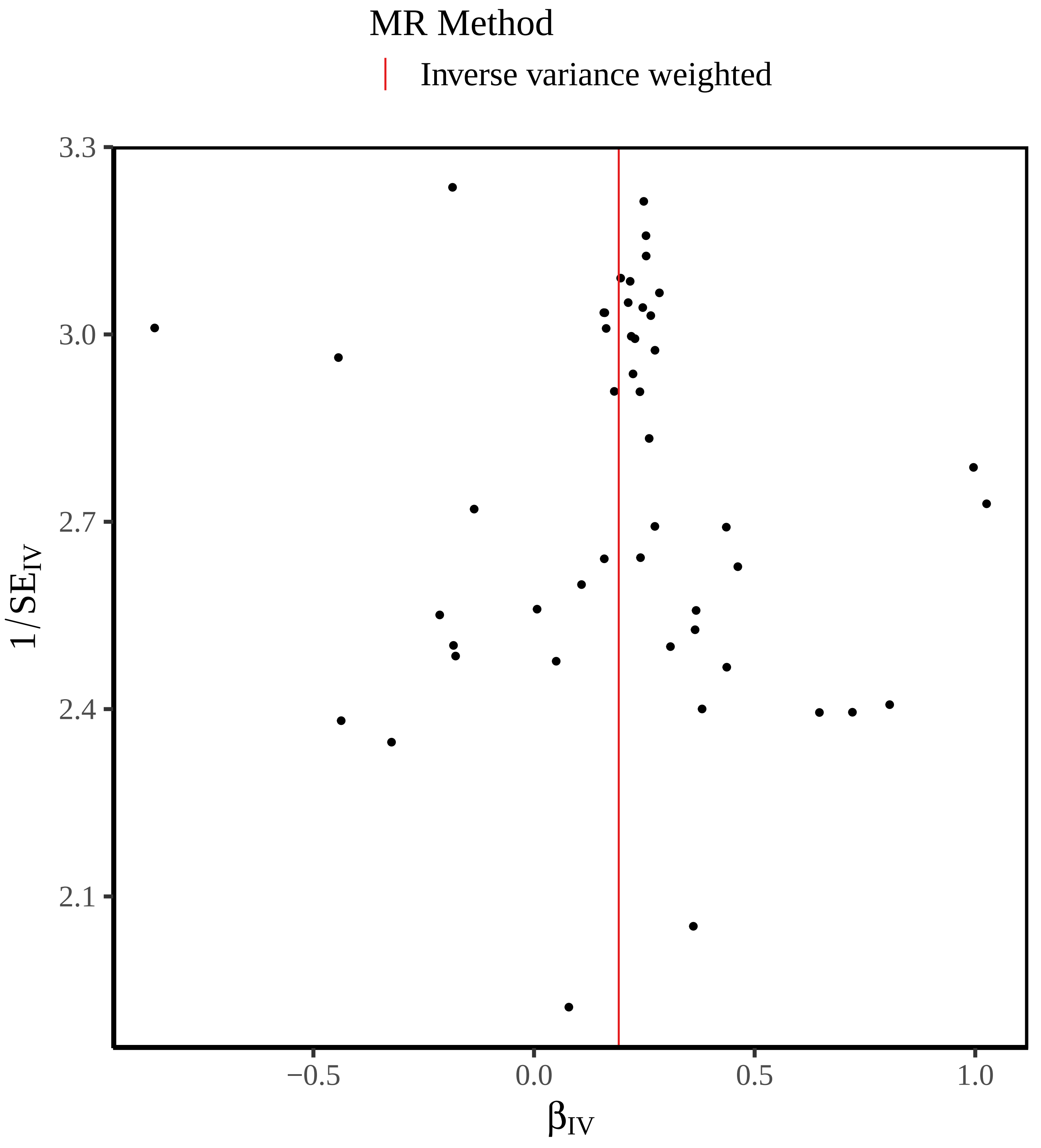
MR Method
Inverse variance weighted
3.3
3.0
2.7
2.4
2.1
1/SE_IV
−0.5
0.0
0.5
1.0
β_IV

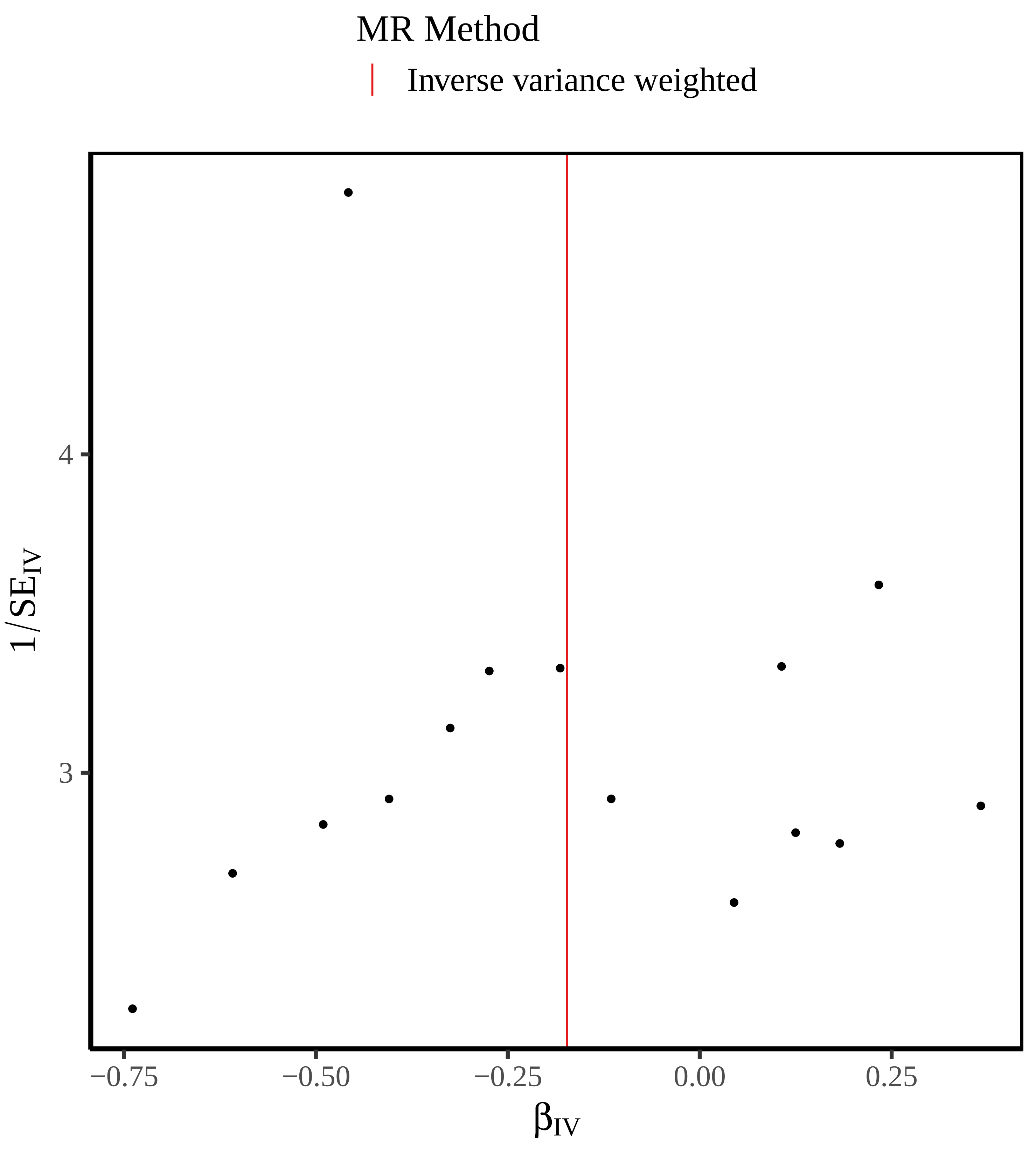

MR Method
Inverse variance weighted
4
3
1/SE$_{IV}$
−0.75
−0.50
−0.25
0.00
0.25
β$_{IV}$

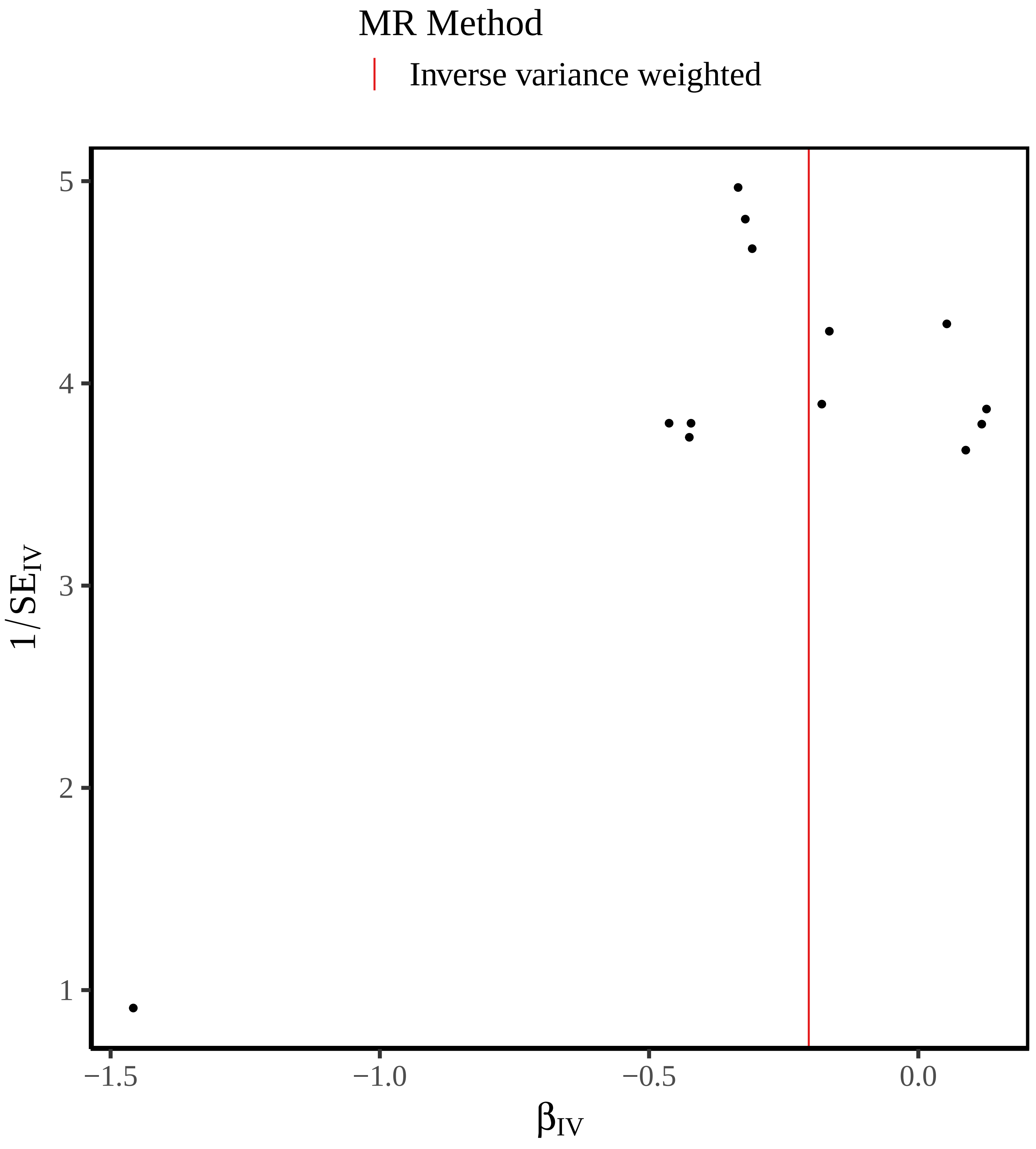

MR Method
Inverse variance weighted
5
4
3
2
1
1/SE$_{IV}$
−1.5
−1.0
−0.5
0.0
β$_{IV}$

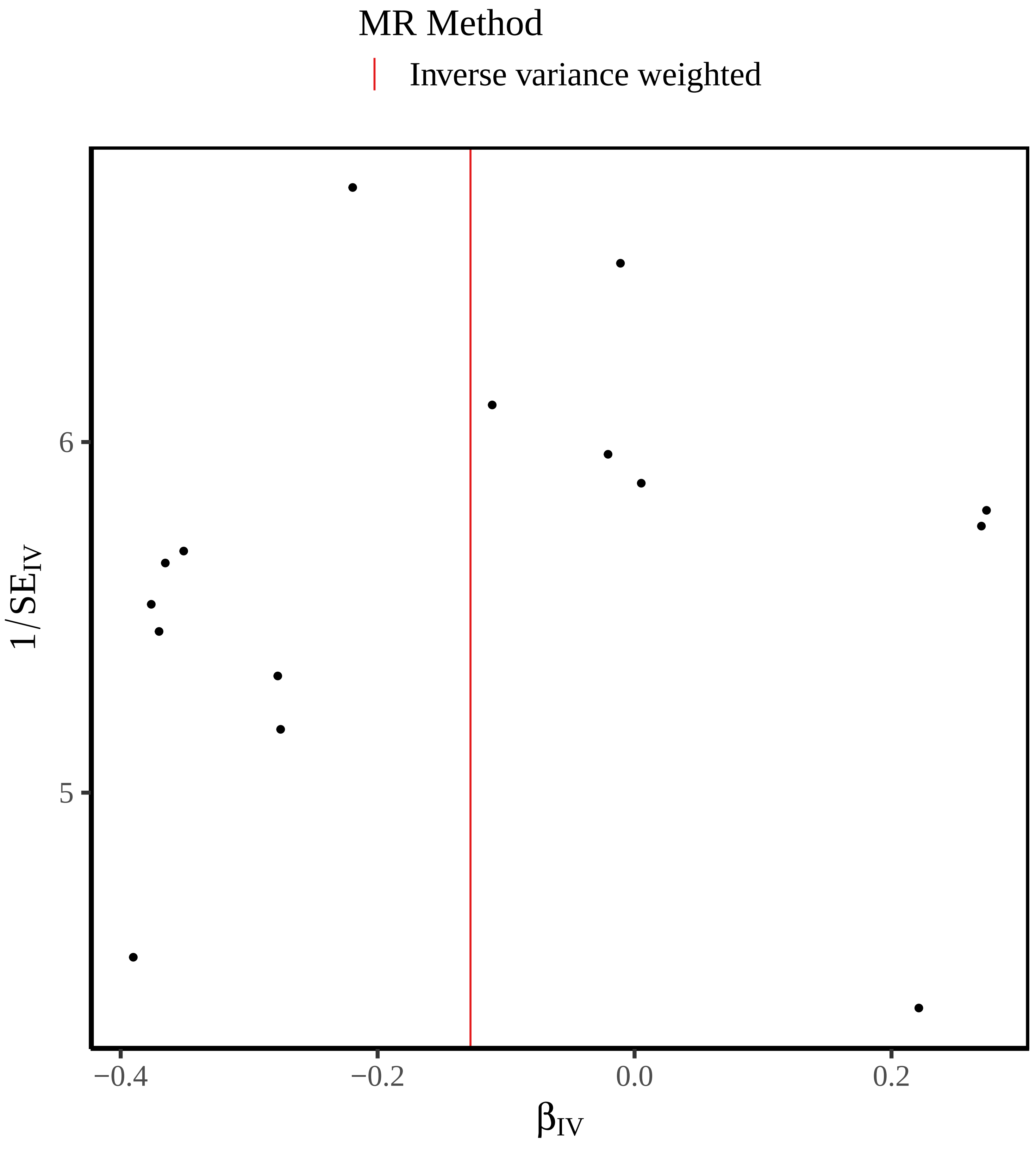
MR Method
Inverse variance weighted
1/SE_IV
6
5
−0.4
−0.2
0.0
0.2
β_IV

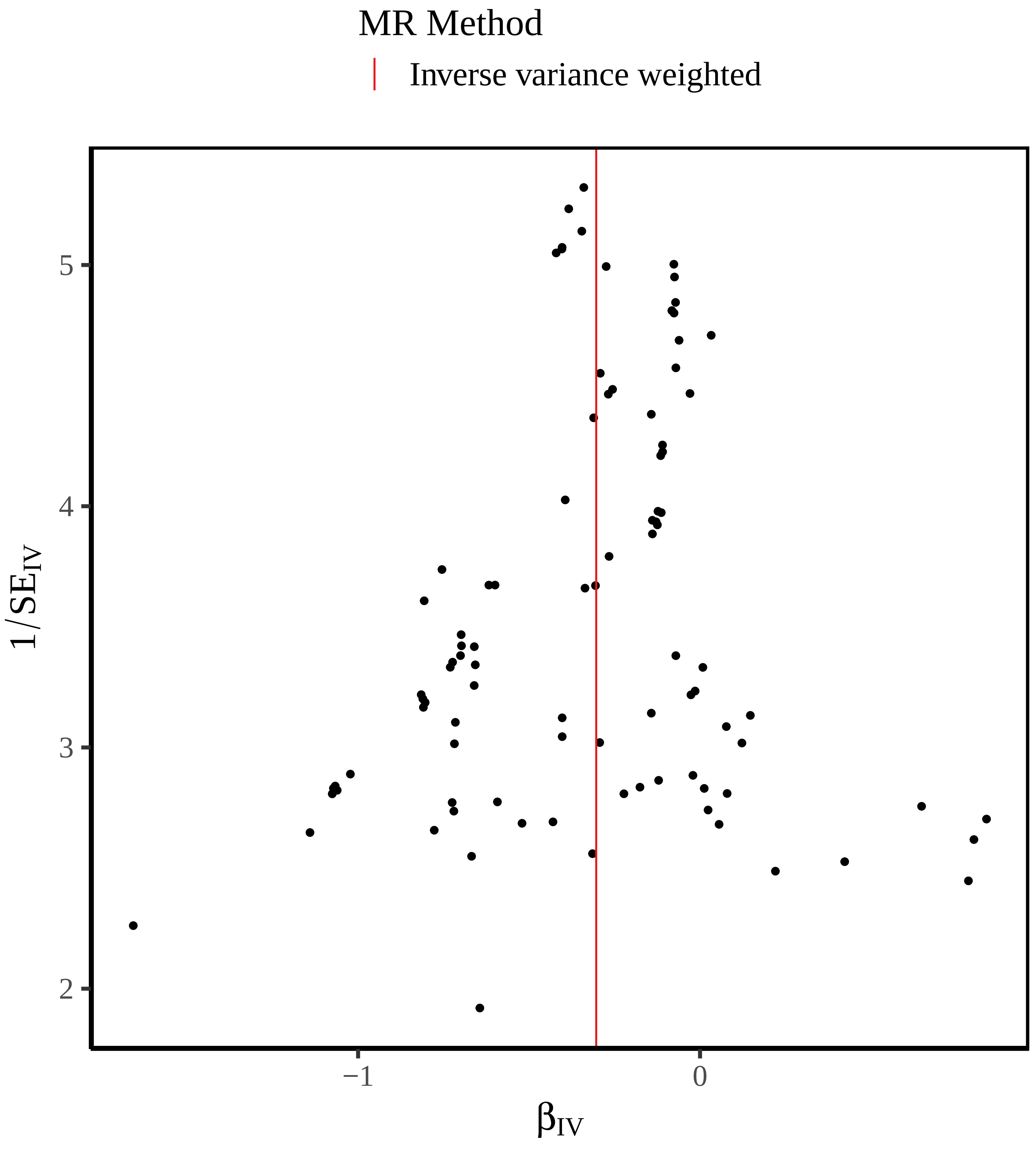
MR Method
Inverse variance weighted
1/SE_IV
5
4
3
2
−1
0
β_IV

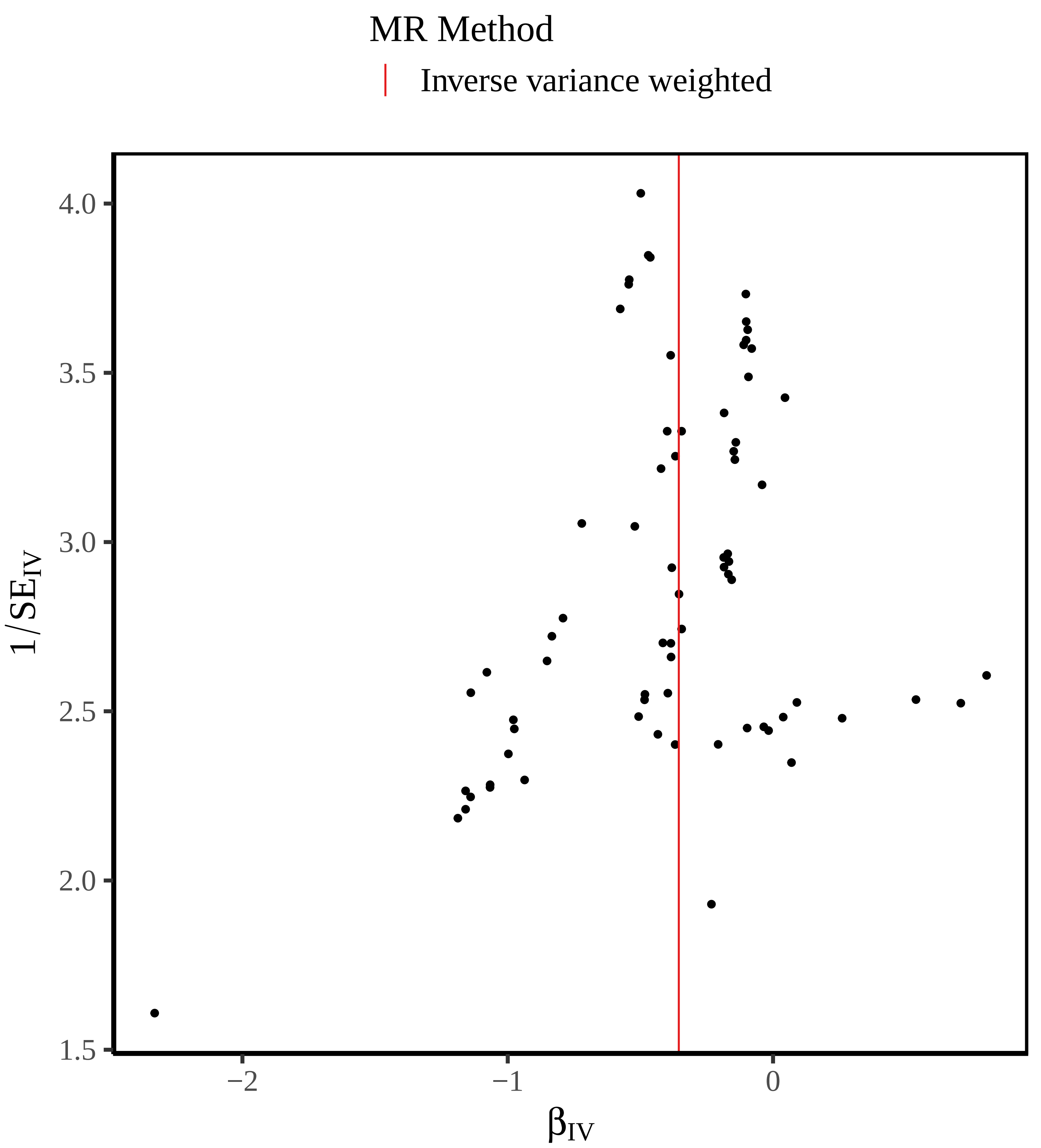
MR Method
Inverse variance weighted
4.0
3.5
3.0
2.5
2.0
1.5
$1/SE_{IV}$
−2
−1
0
$\beta_{IV}$

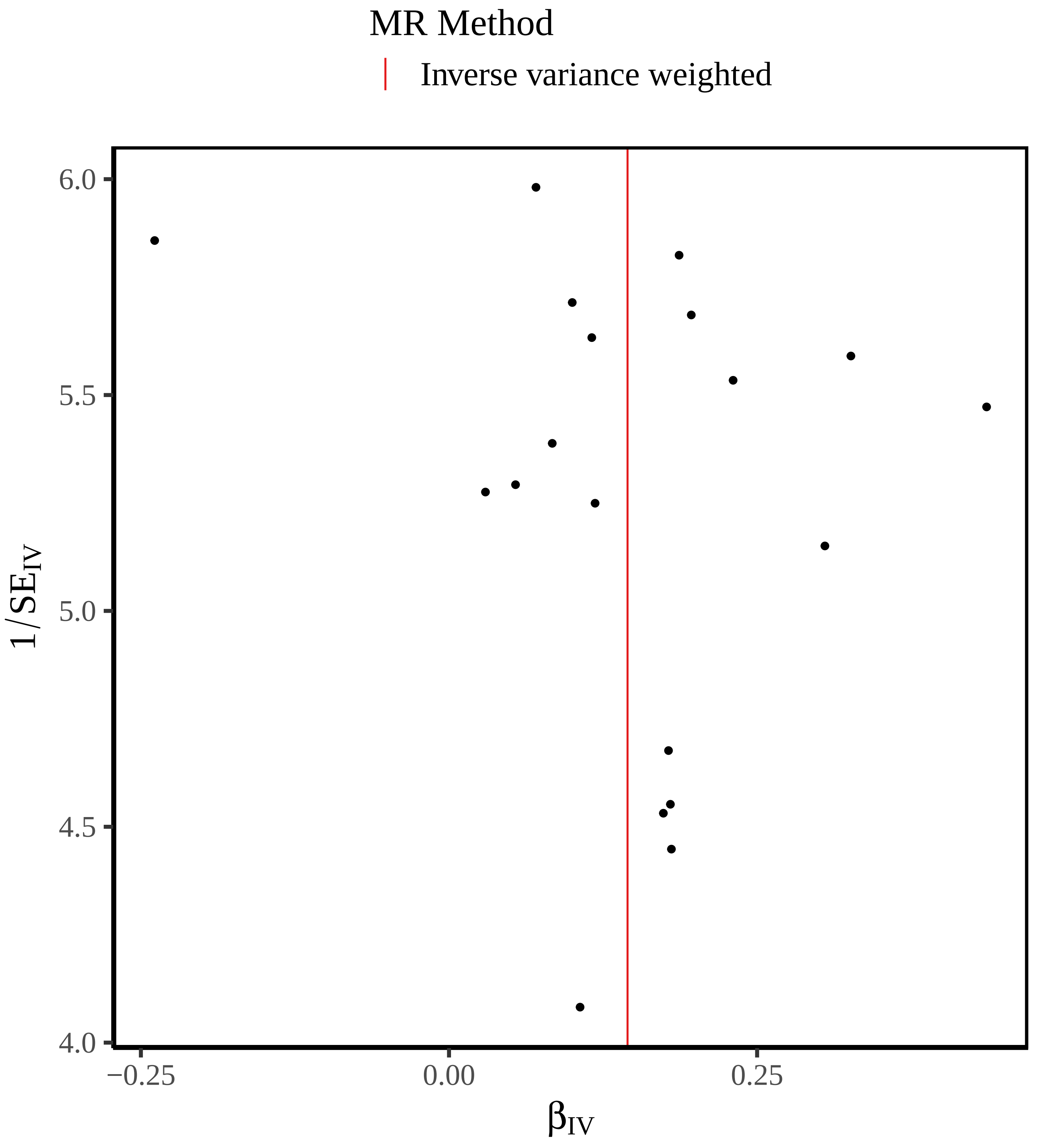

MR Method
Inverse variance weighted
6.0
5.5
5.0
4.5
4.0
1/SE_IV
−0.25
0.00
0.25
β_IV

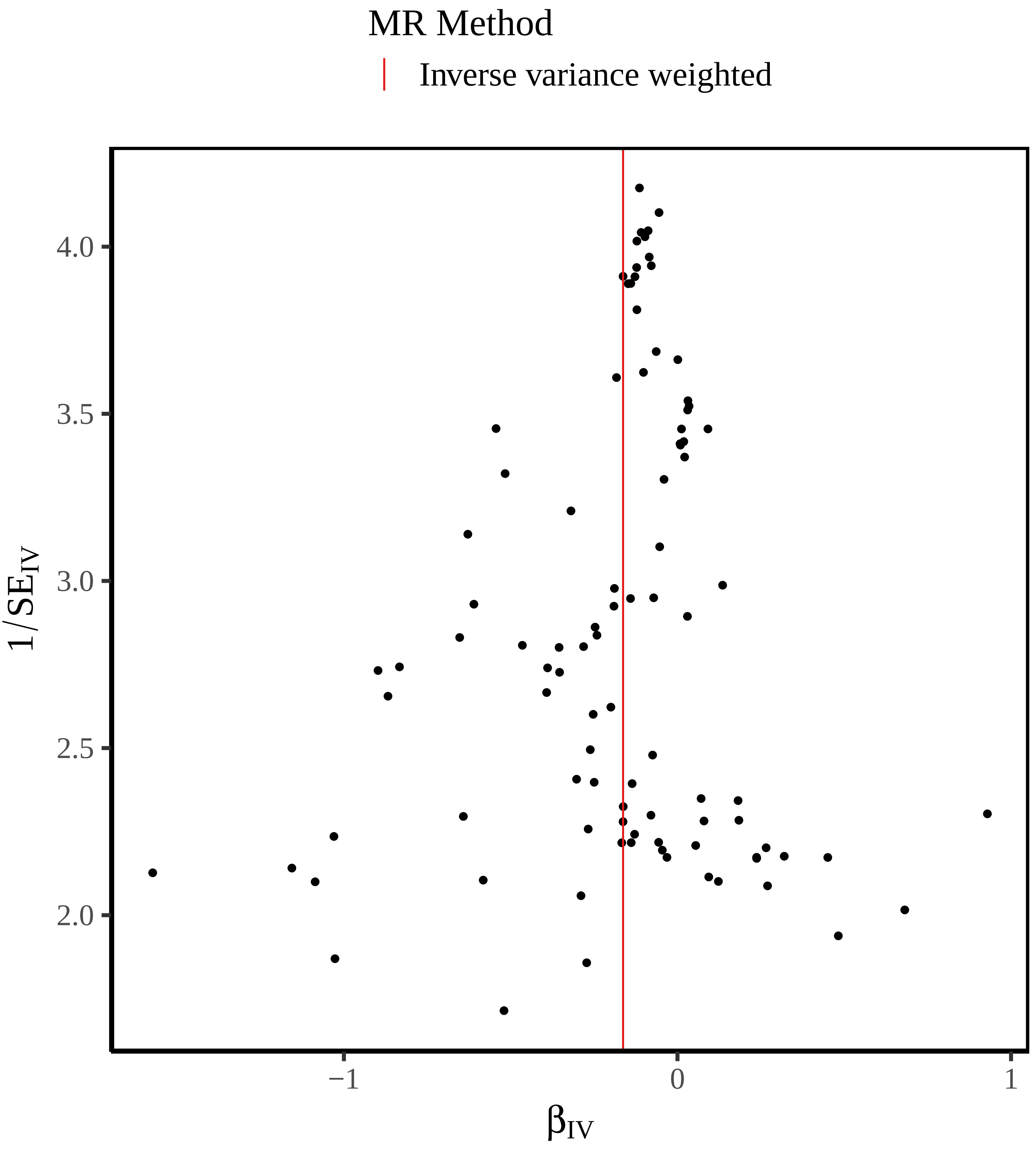
MR Method
Inverse variance weighted
4.0
3.5
3.0
2.5
2.0
$1/SE_{IV}$
−1
0
1
$\beta_{IV}$

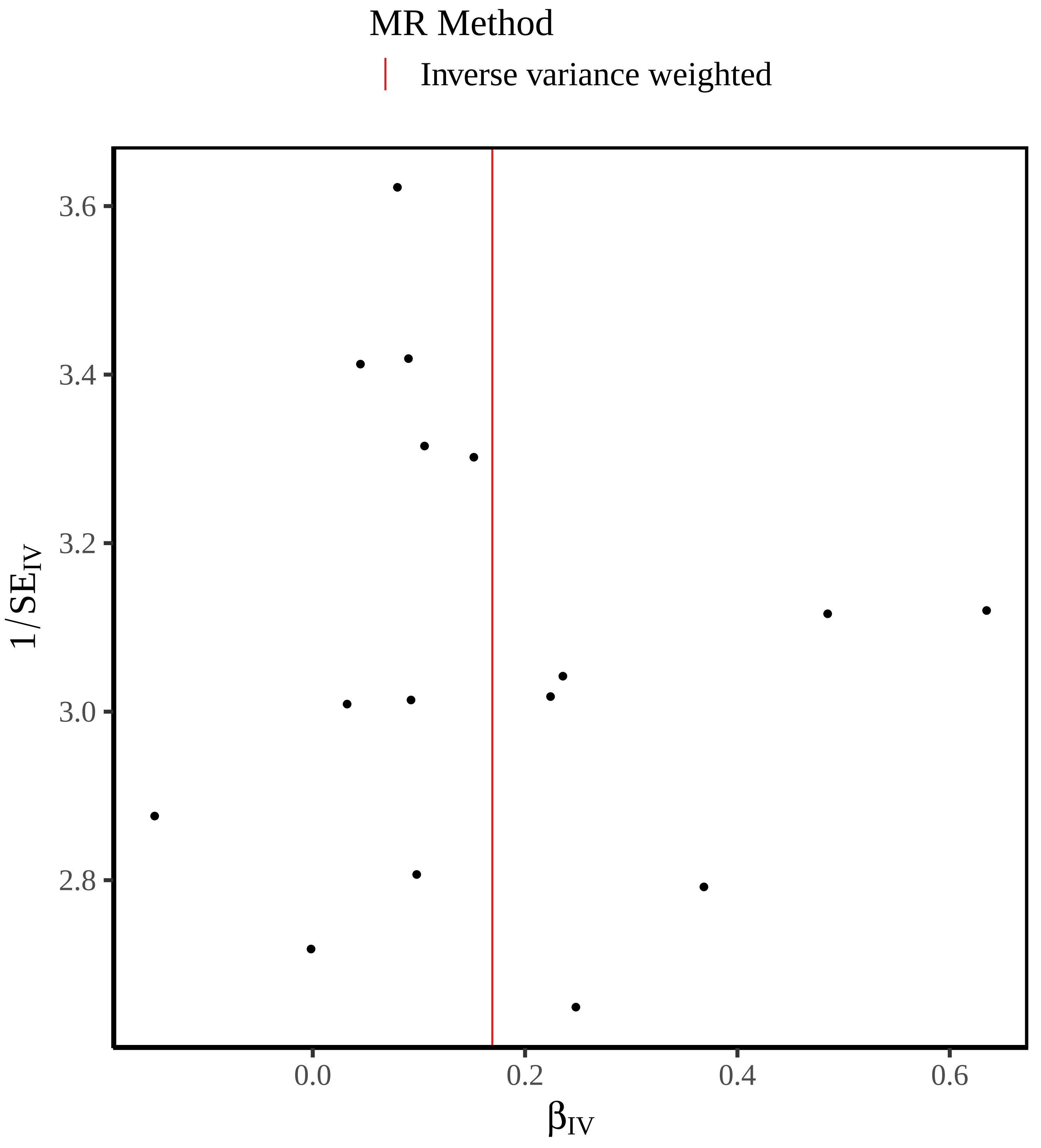
MR Method
Inverse variance weighted
3.6
3.4
3.2
3.0
2.8
$1/SE_{IV}$
0.0
0.2
0.4
0.6
$\beta_{IV}$

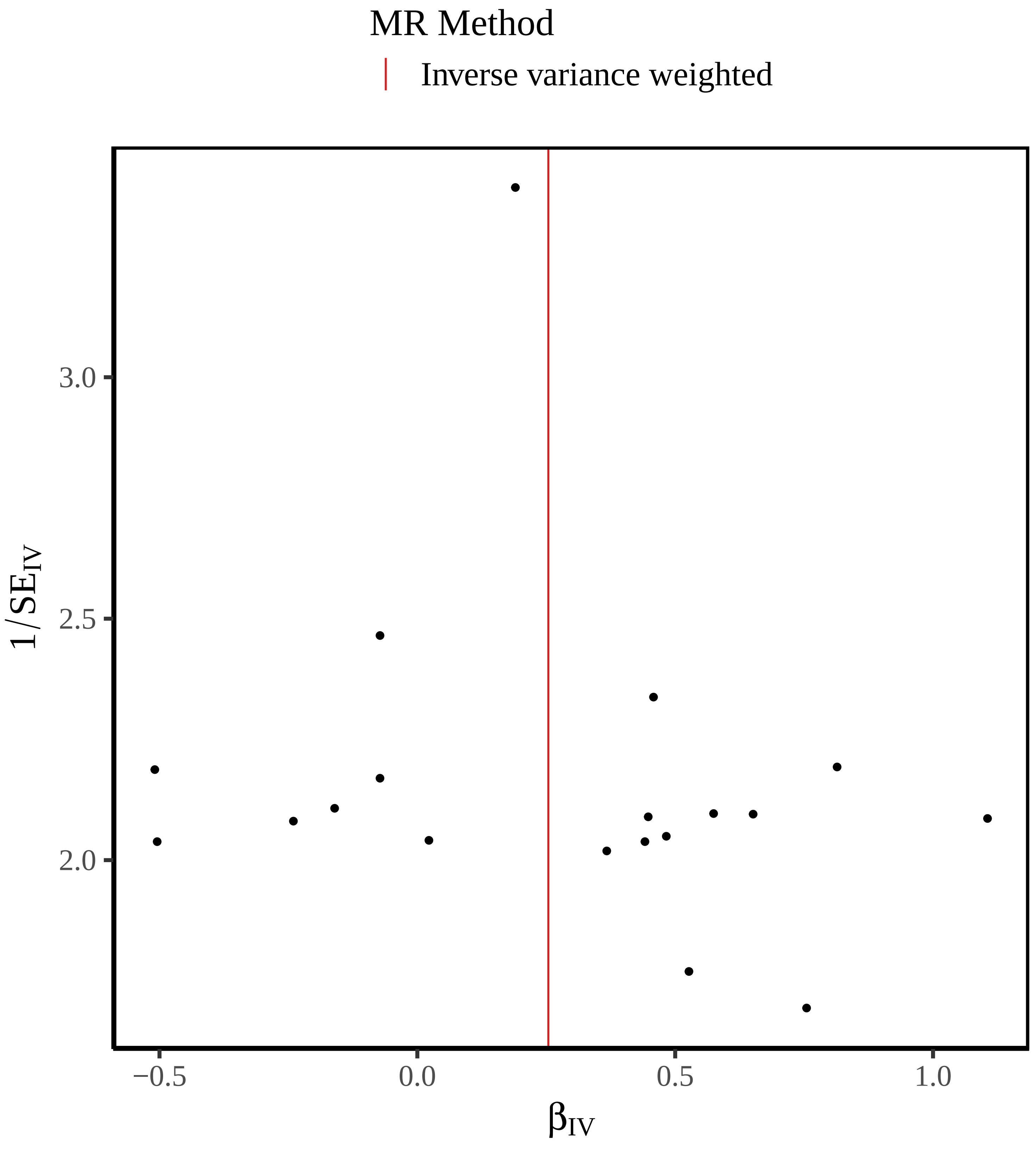
MR Method
Inverse variance weighted
3.0
2.5
2.0
1/SE_IV
−0.5
0.0
0.5
1.0
β_IV

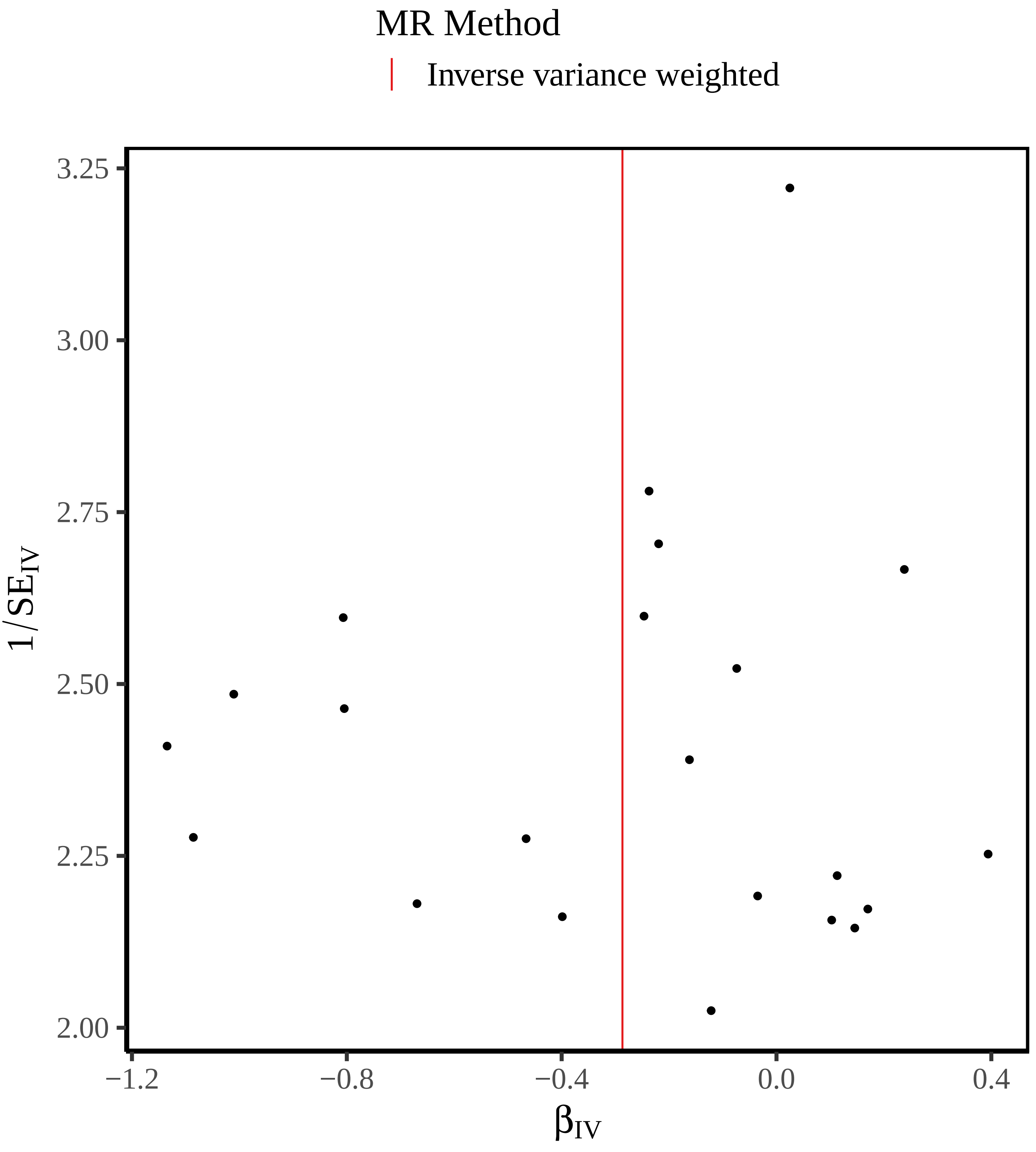
MR Method
Inverse variance weighted
3.25
3.00
2.75
2.50
2.25
2.00
1/SE_IV
−1.2
−0.8
−0.4
0.0
0.4
β_IV

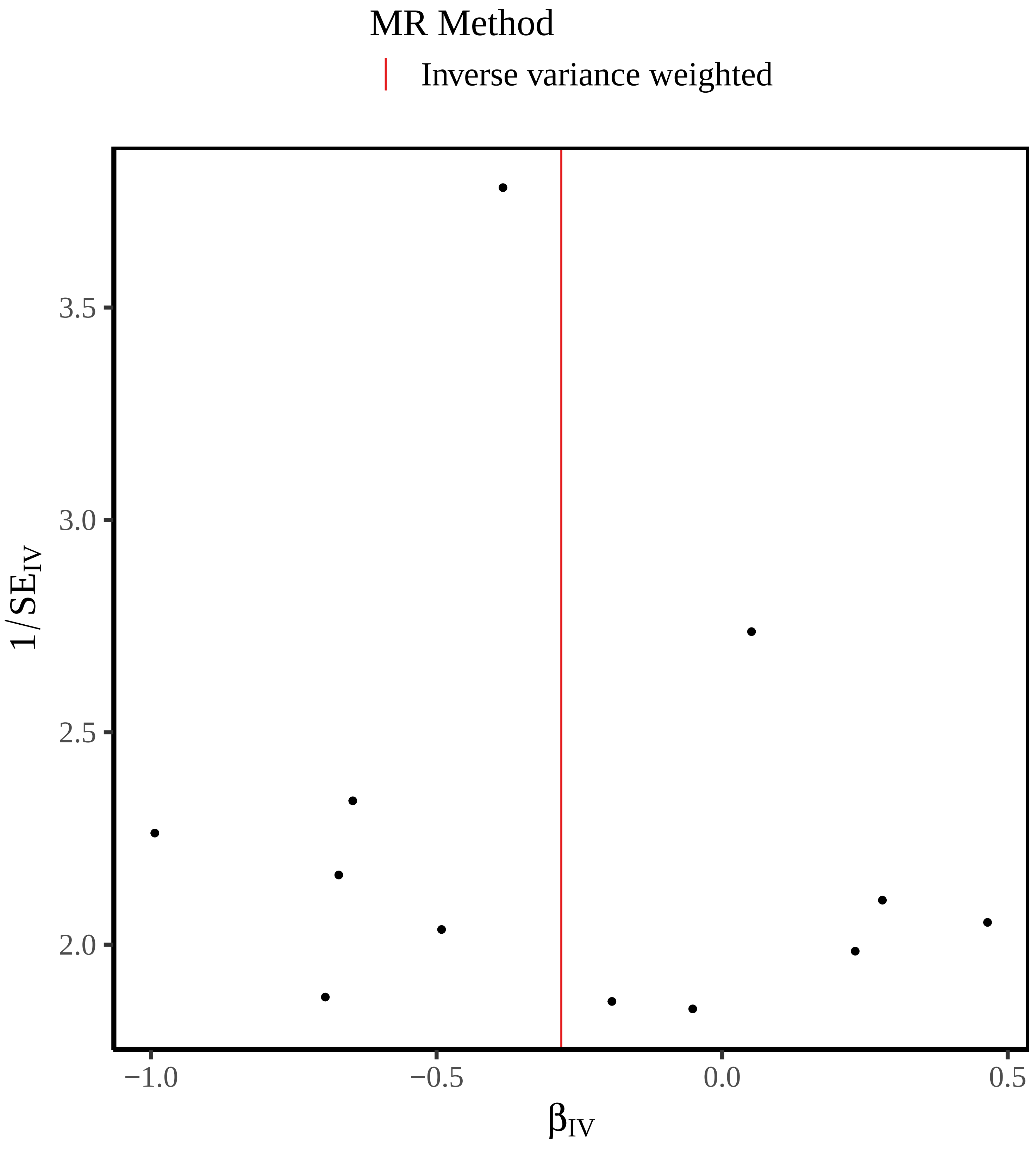
MR Method
Inverse variance weighted
1/SE_IV
3.5
3.0
2.5
2.0
−1.0
−0.5
0.0
0.5
β_IV

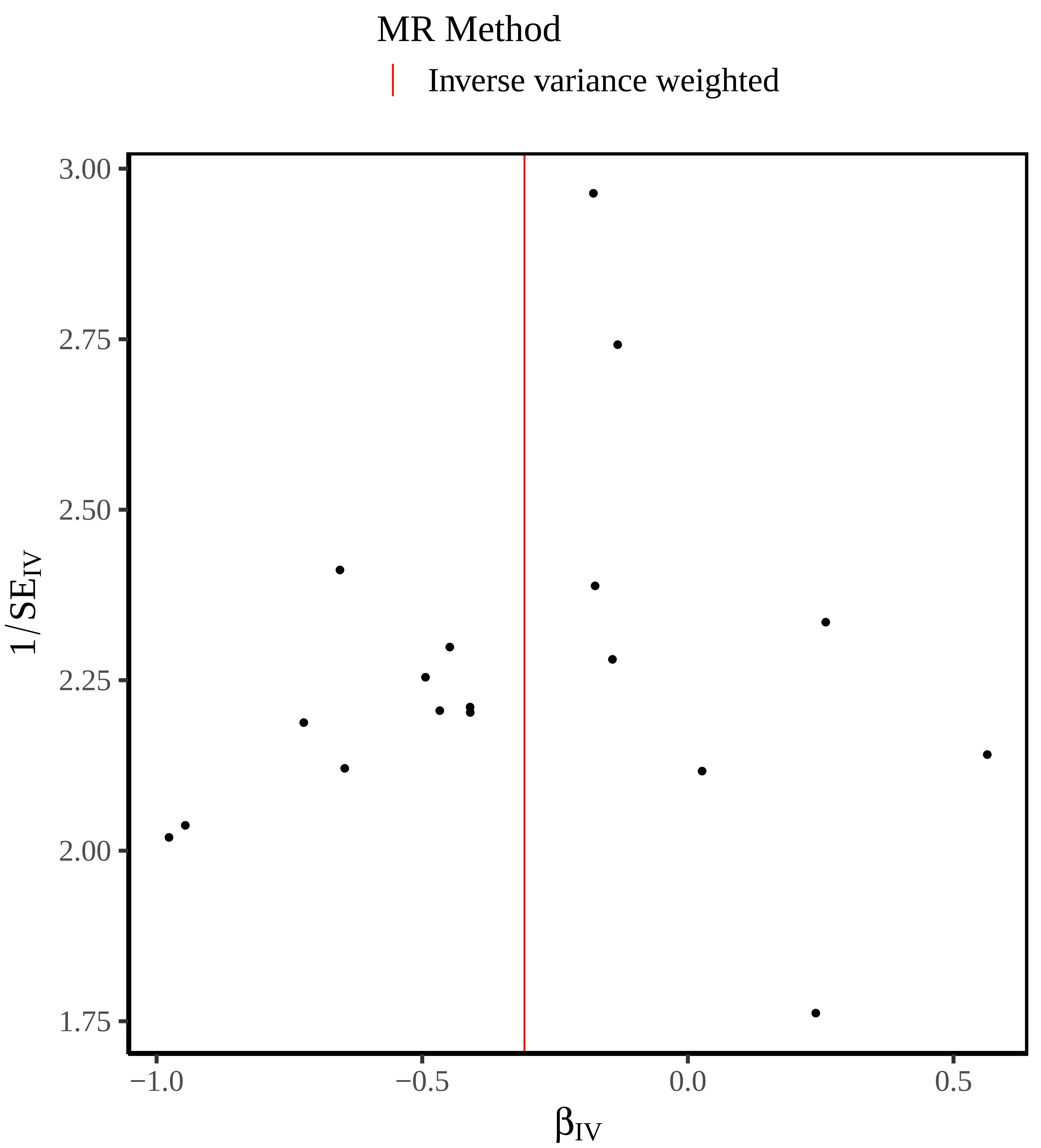
MR Method
Inverse variance weighted
3.00
2.75
2.50
2.25
2.00
1.75
$1/SE_{IV}$
−1.0
−0.5
0.0
0.5
$\beta_{IV}$

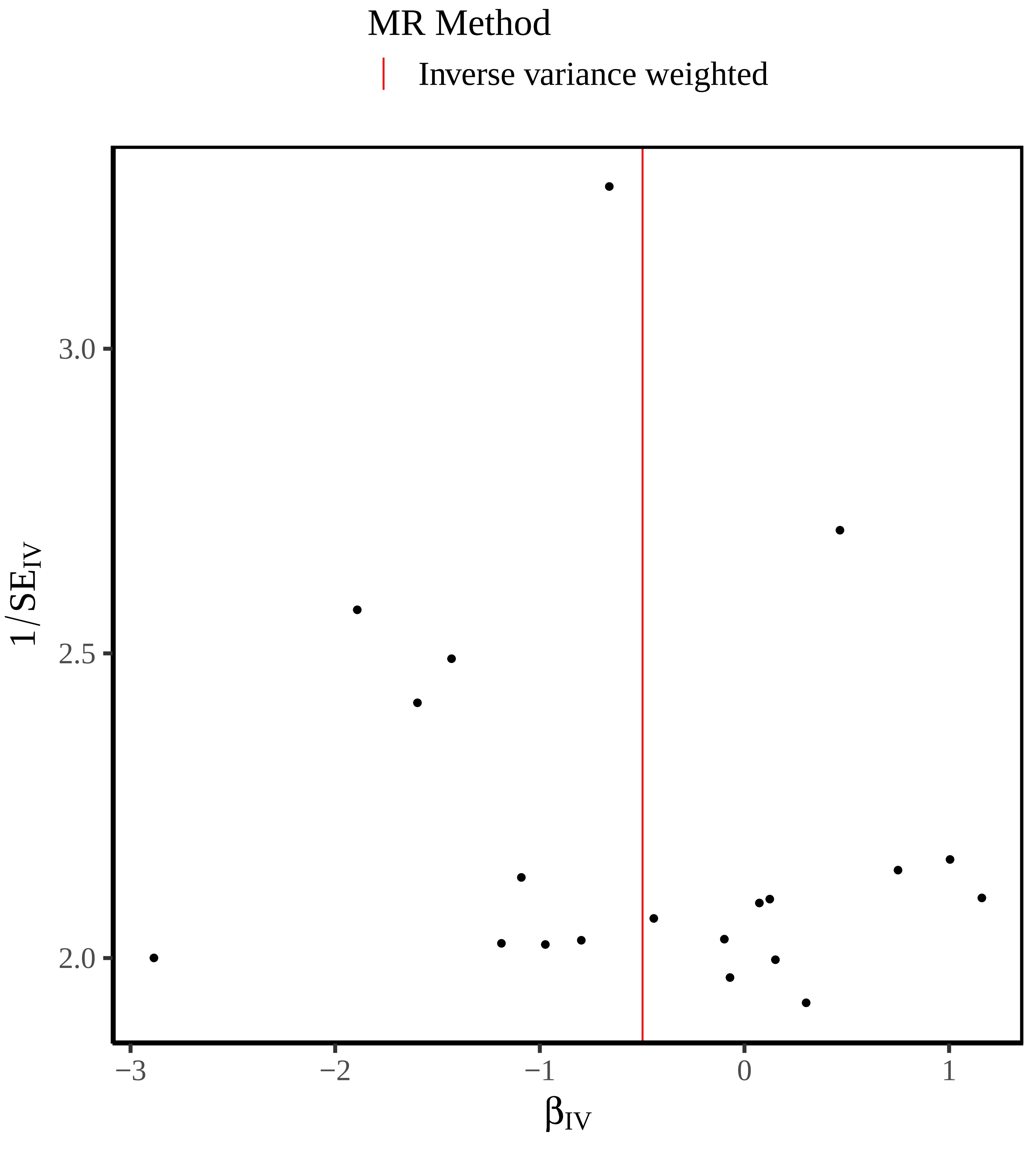

MR Method
Inverse variance weighted
1/SE_IV
3.0
2.5
2.0
−3
−2
−1
0
1
β_IV

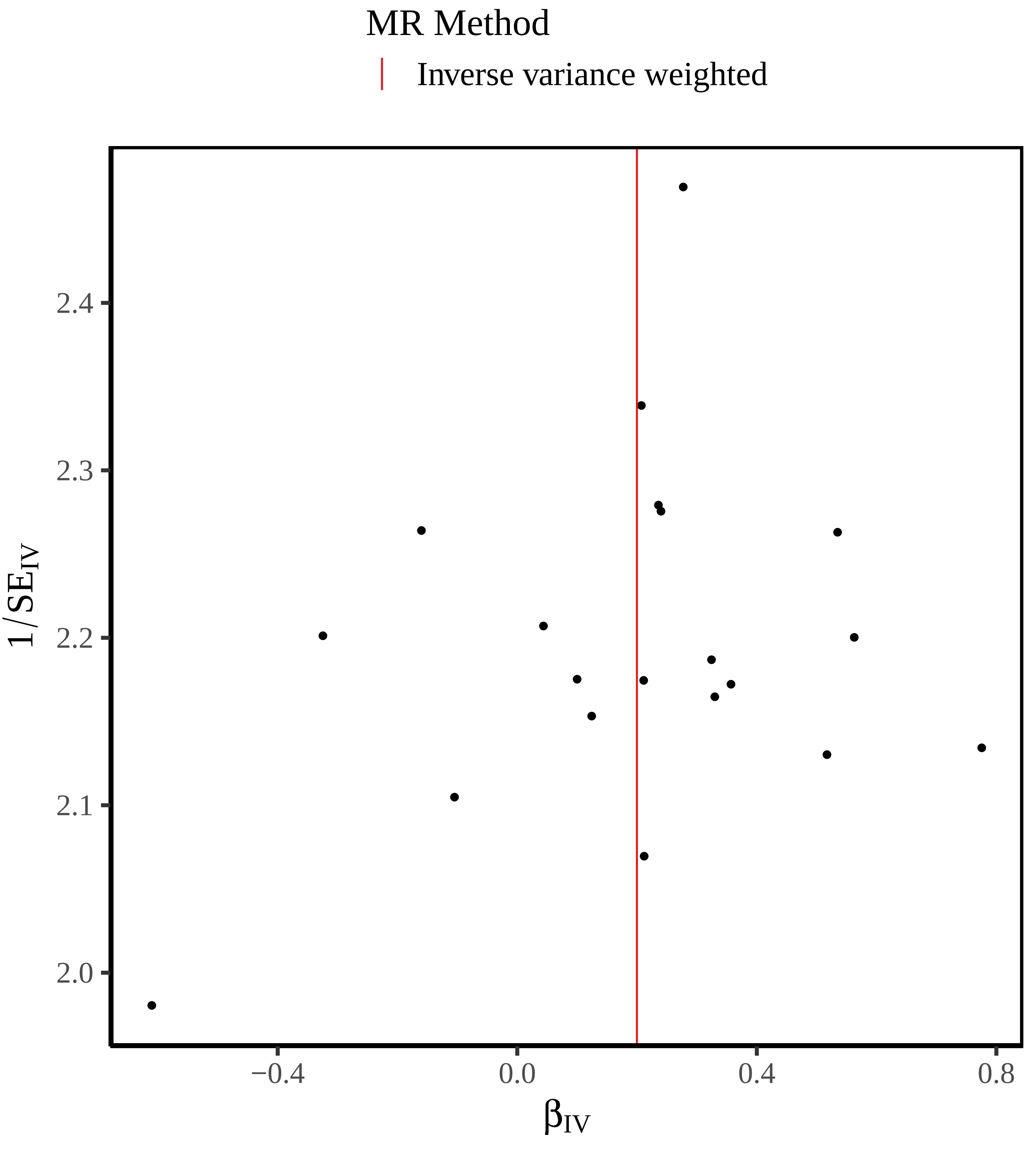

MR Method
Inverse variance weighted
2.4
2.3
2.2
2.1
2.0
$1/SE_{IV}$
−0.4
0.0
0.4
0.8
$\beta_{IV}$

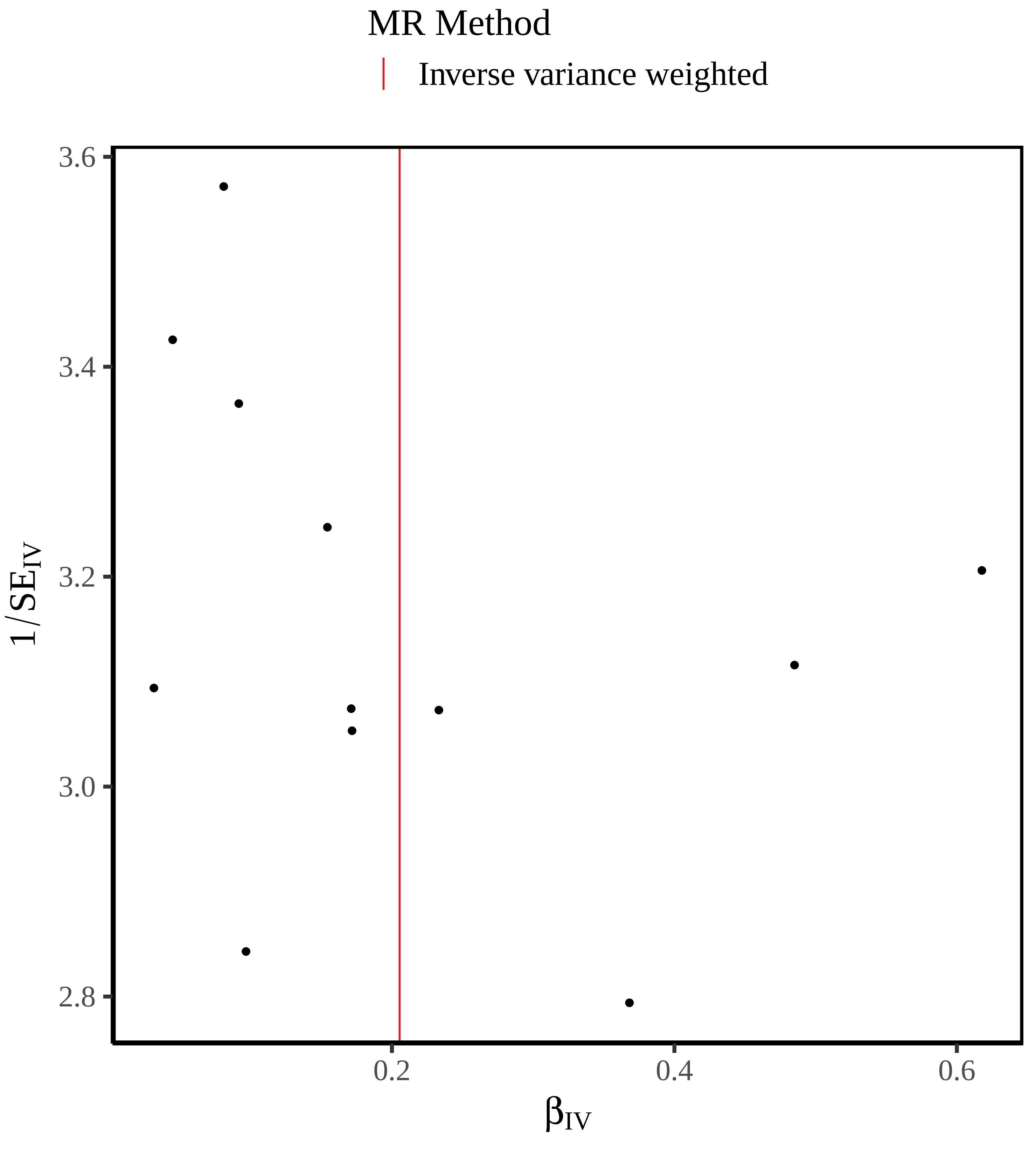
MR Method
Inverse variance weighted
3.6
3.4
3.2
3.0
2.8
$1/SE_{IV}$
0.2
0.4
0.6
$\beta_{IV}$

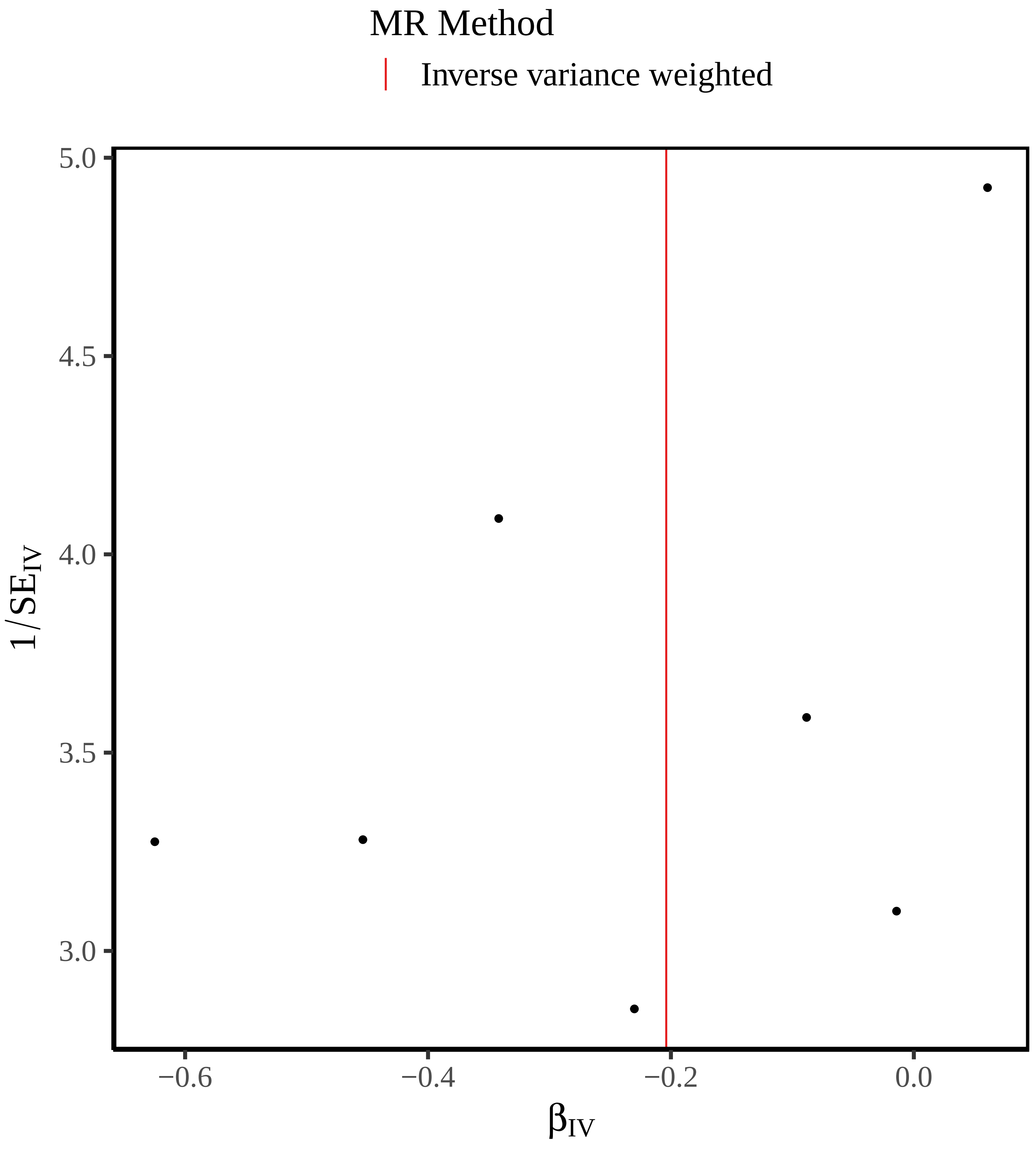
MR Method
Inverse variance weighted
5.0
4.5
4.0
3.5
3.0
1/SE$_{IV}$
−0.6
−0.4
−0.2
0.0
β$_{IV}$

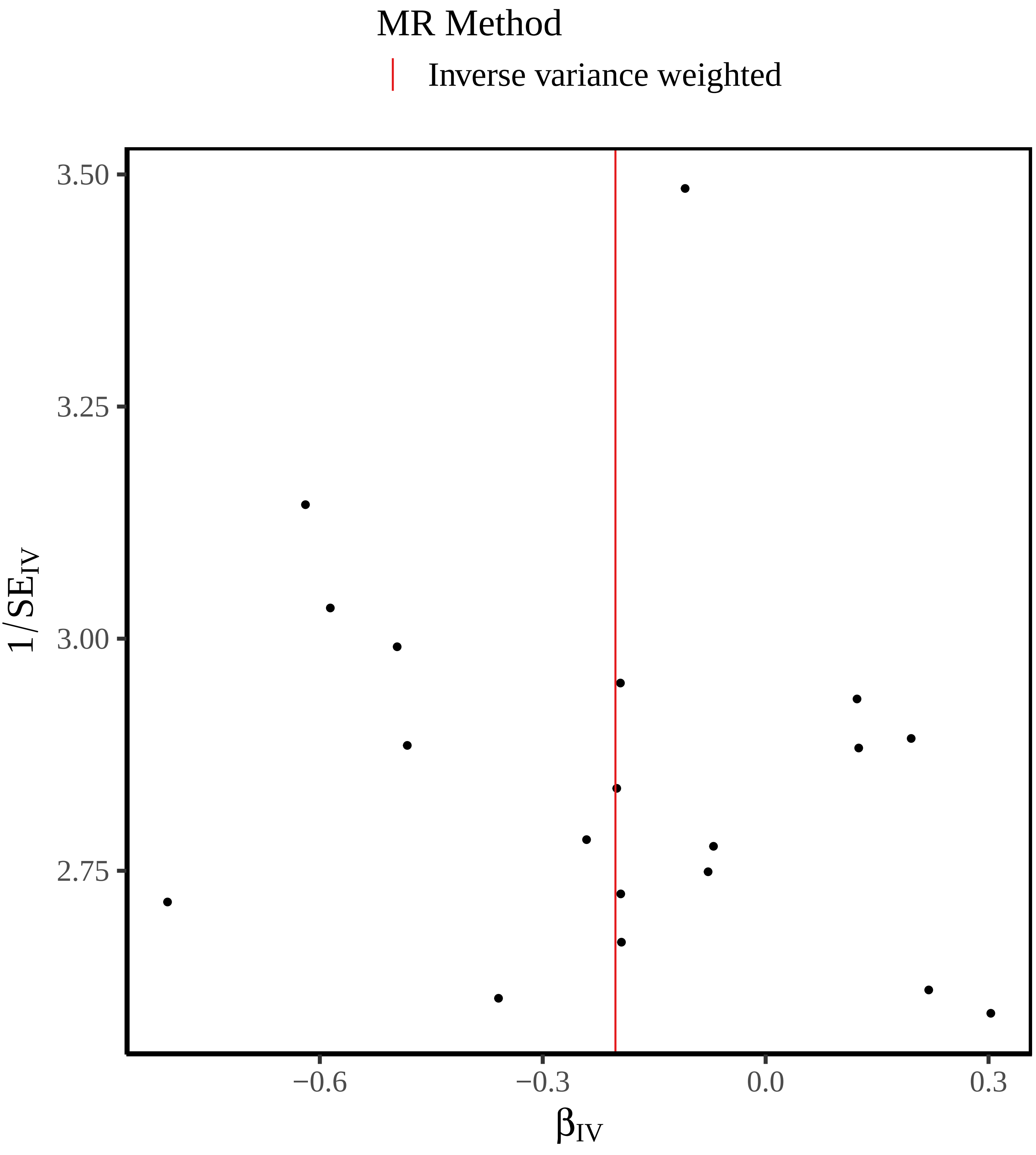

MR Method
Inverse variance weighted
3.50
3.25
3.00
2.75
1/SE$_{IV}$
−0.6
−0.3
0.0
0.3
β$_{IV}$

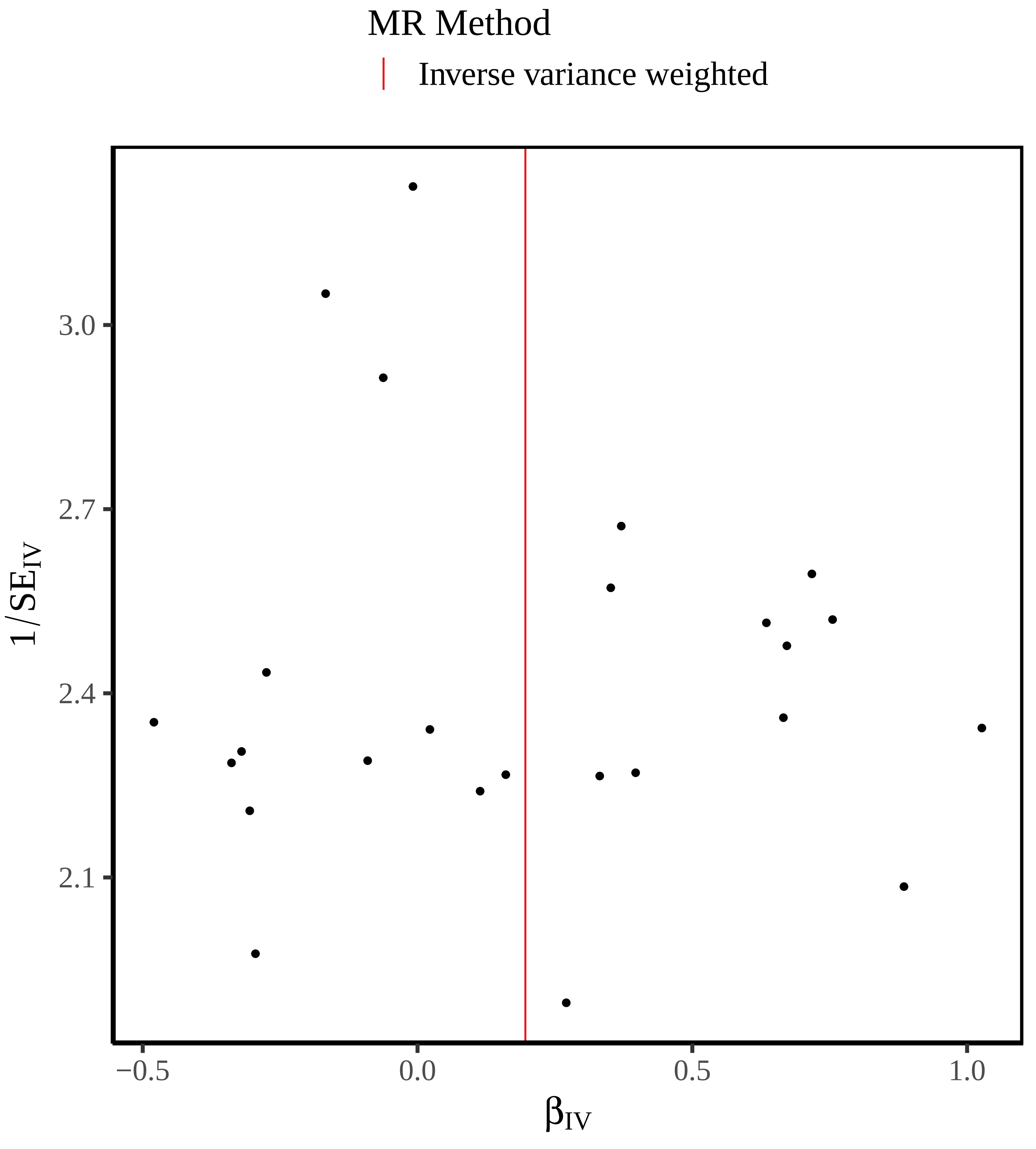
MR Method
Inverse variance weighted
3.0
2.7
2.4
2.1
1/SE_IV
−0.5
0.0
0.5
1.0
β_IV

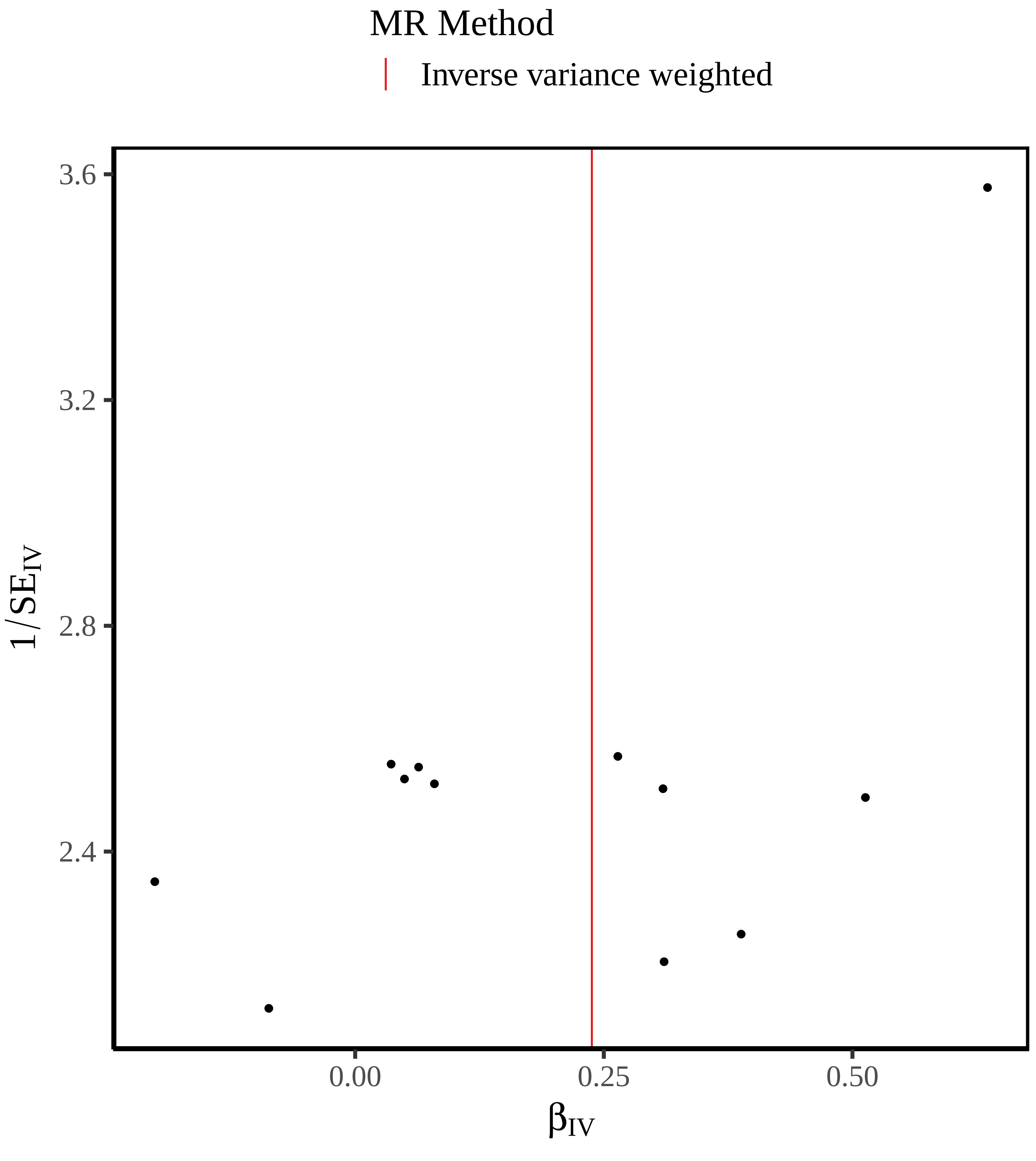
MR Method
Inverse variance weighted
3.6
3.2
2.8
2.4
$1/SE_{IV}$
0.00
0.25
0.50
$\beta_{IV}$

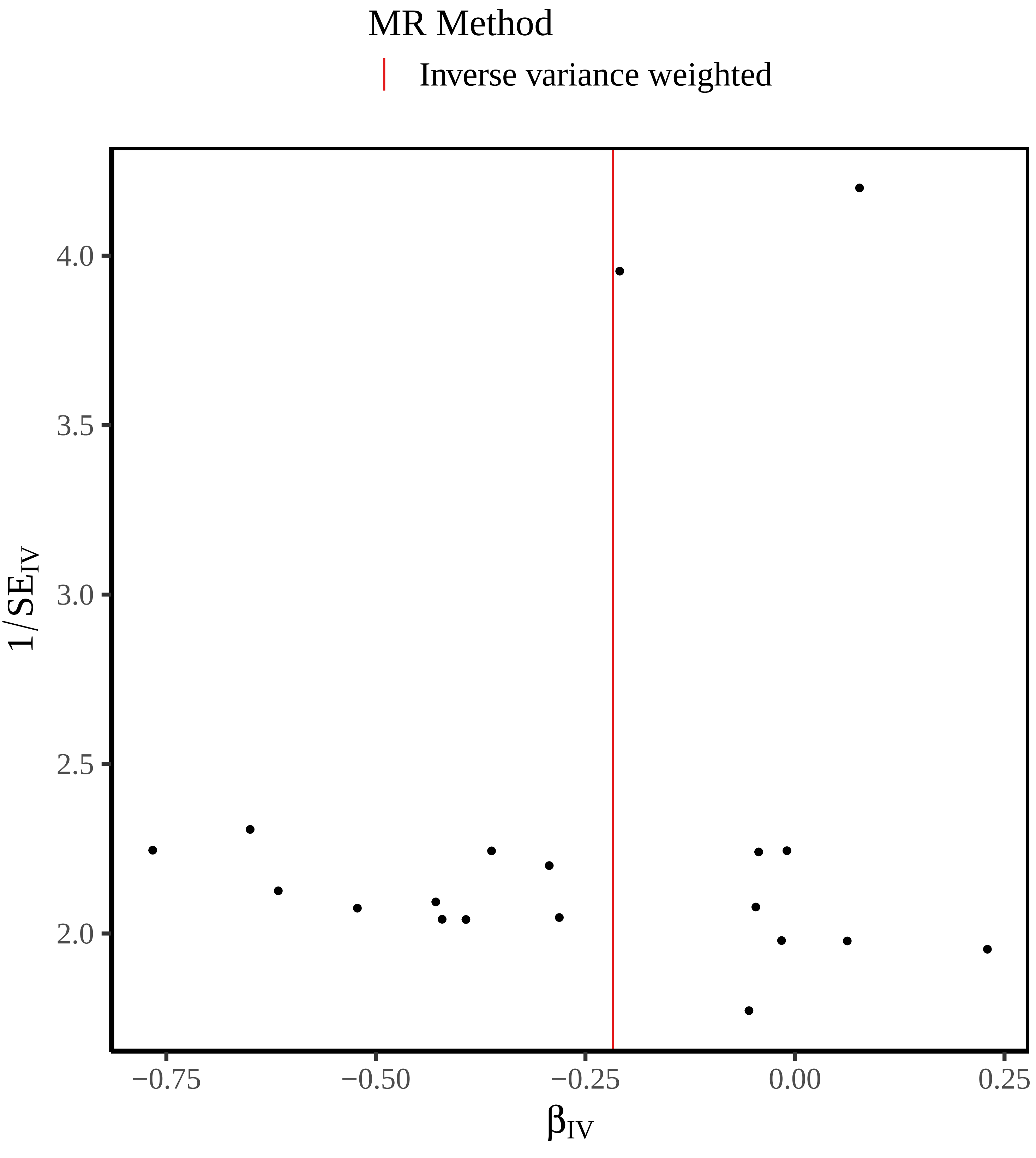
MR Method
Inverse variance weighted
4.0
3.5
3.0
2.5
2.0
1/SE$_{IV}$
−0.75
−0.50
−0.25
0.00
0.25
$\beta_{IV}$

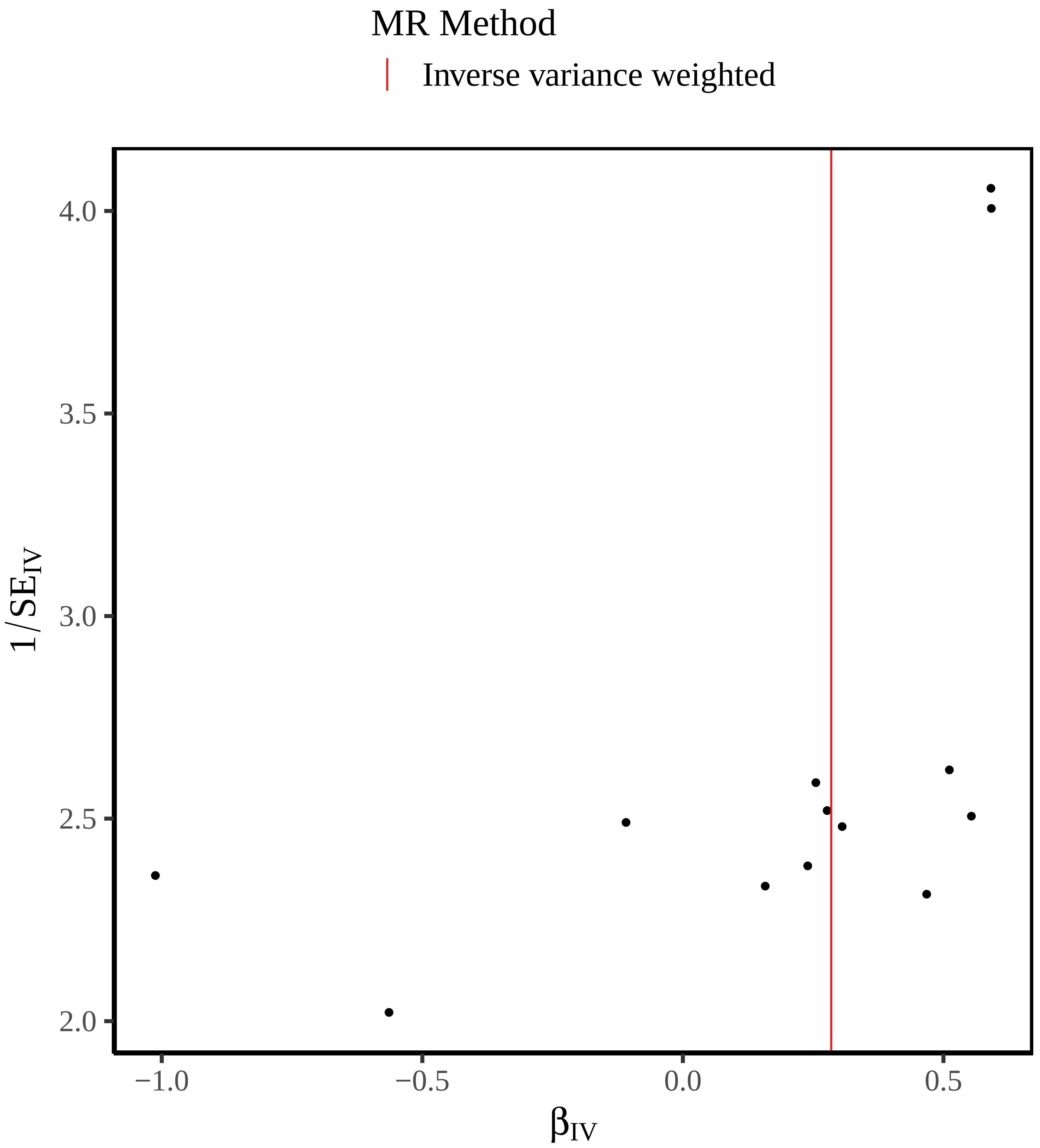

MR Method
Inverse variance weighted
1/SE_IV
4.0
3.5
3.0
2.5
2.0
−1.0
−0.5
0.0
0.5
β_IV

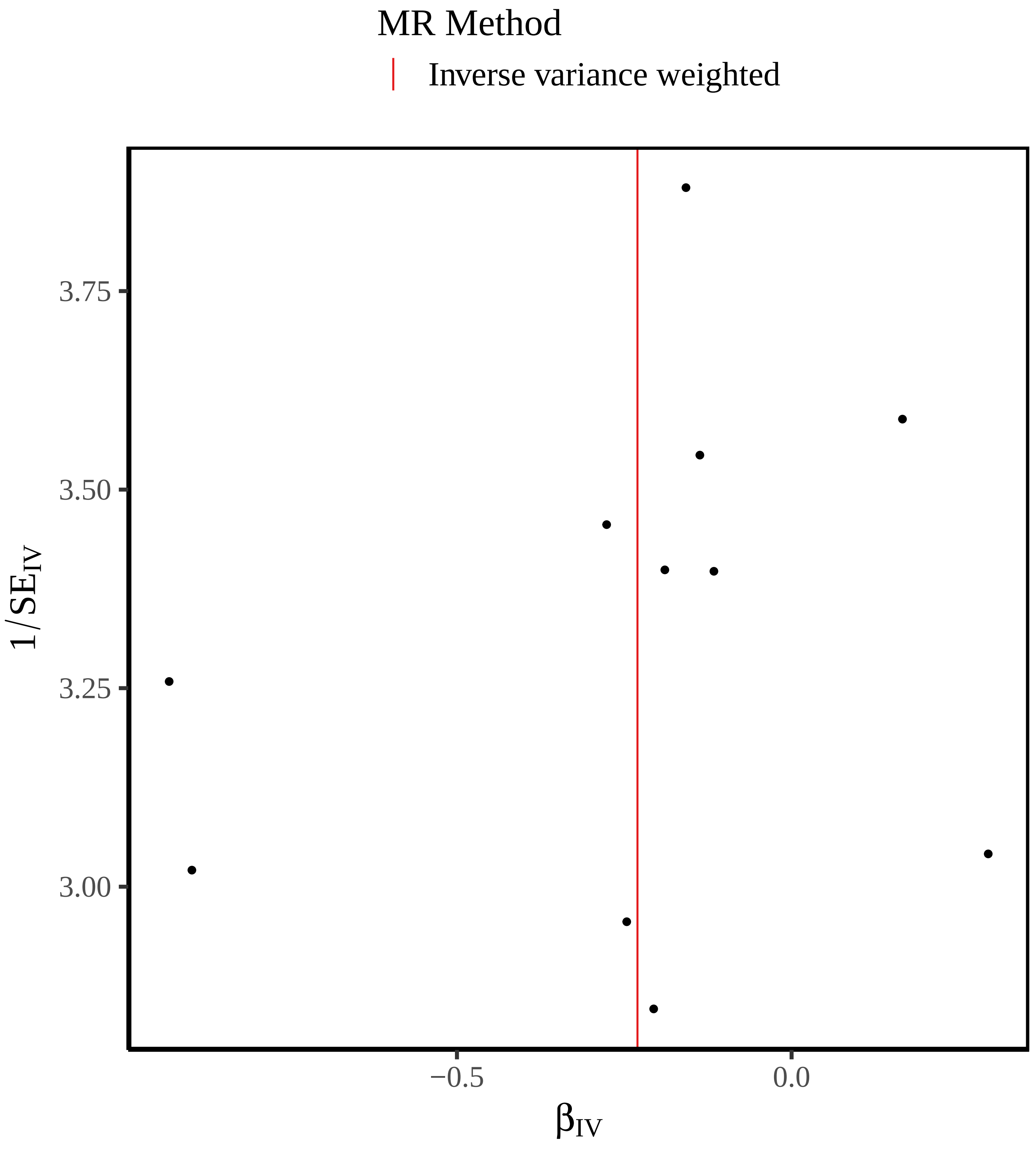

MR Method
Inverse variance weighted
3.75
3.50
3.25
3.00
−0.5
0.0
$1/SE_{IV}$
$\beta_{IV}$

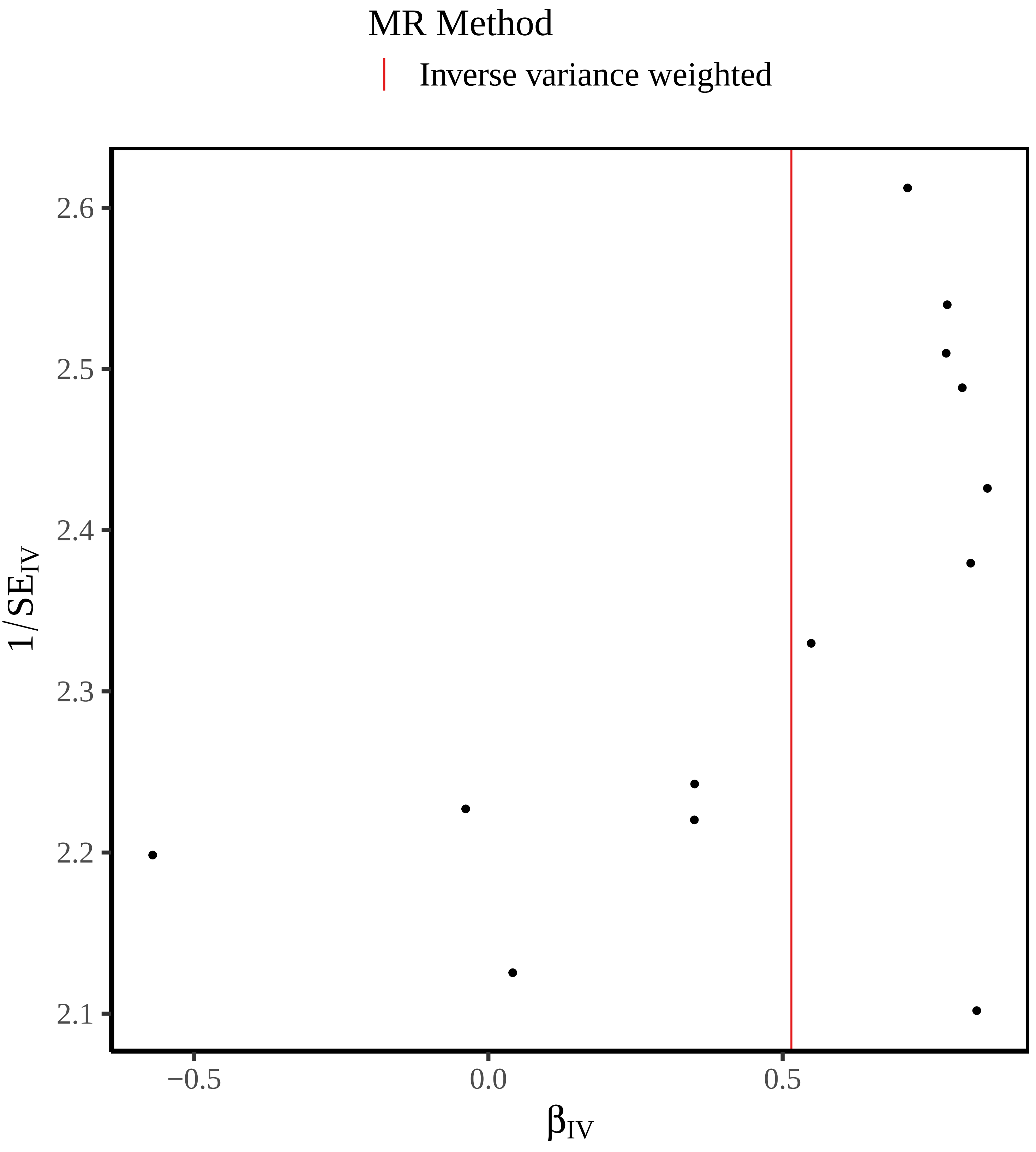
MR Method
Inverse variance weighted
2.6
2.5
2.4
2.3
2.2
2.1
1/SE$_{IV}$
−0.5
0.0
0.5
β$_{IV}$

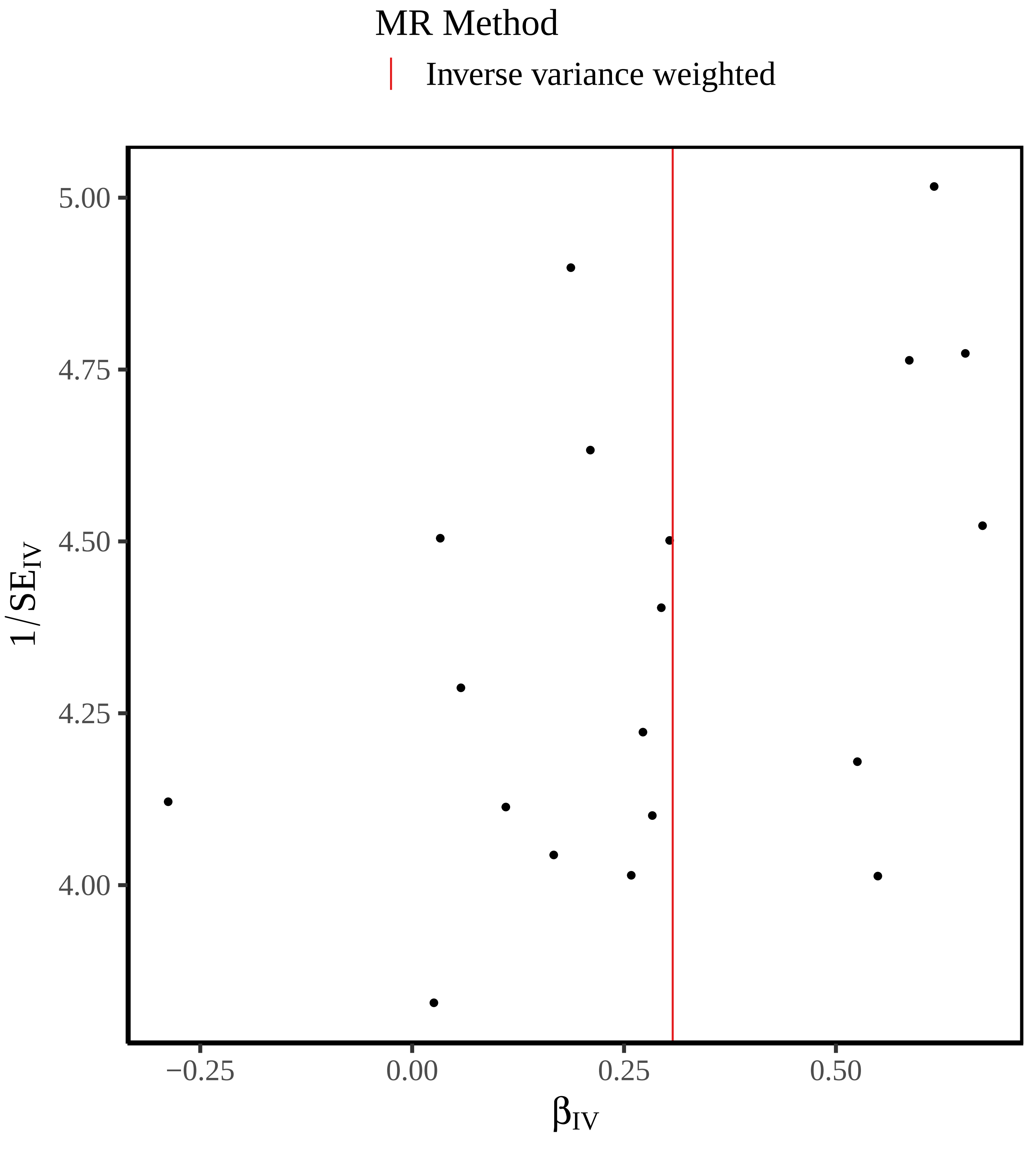

MR Method
Inverse variance weighted
5.00
4.75
4.50
4.25
4.00
1/SE_IV
−0.25
0.00
0.25
0.50
β_IV

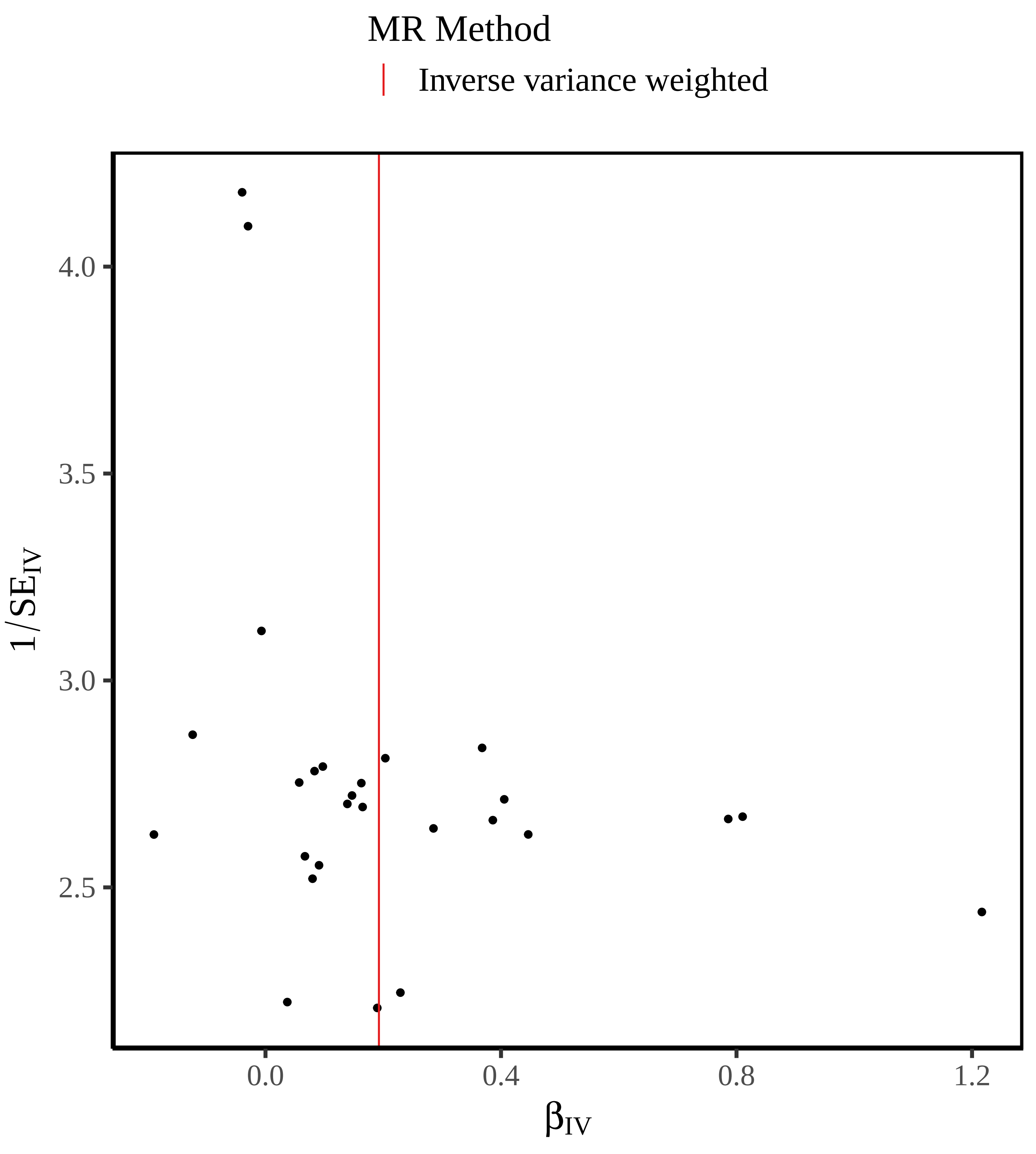
MR Method
Inverse variance weighted
1/SE_IV
4.0
3.5
3.0
2.5
0.0
0.4
0.8
1.2
β_IV

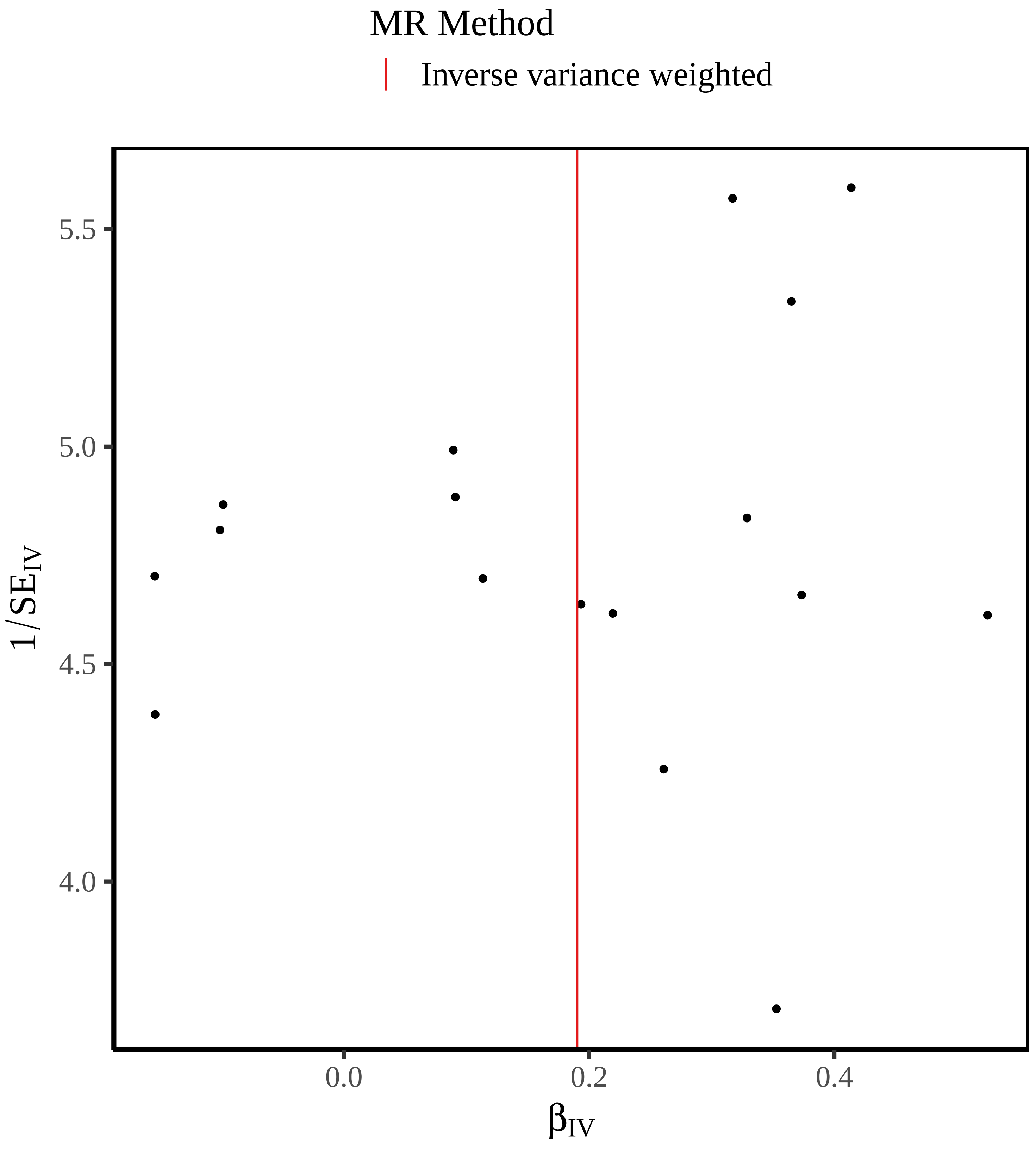

MR Method
Inverse variance weighted
5.5
5.0
4.5
4.0
1/SE_IV
0.0
0.2
0.4
β_IV

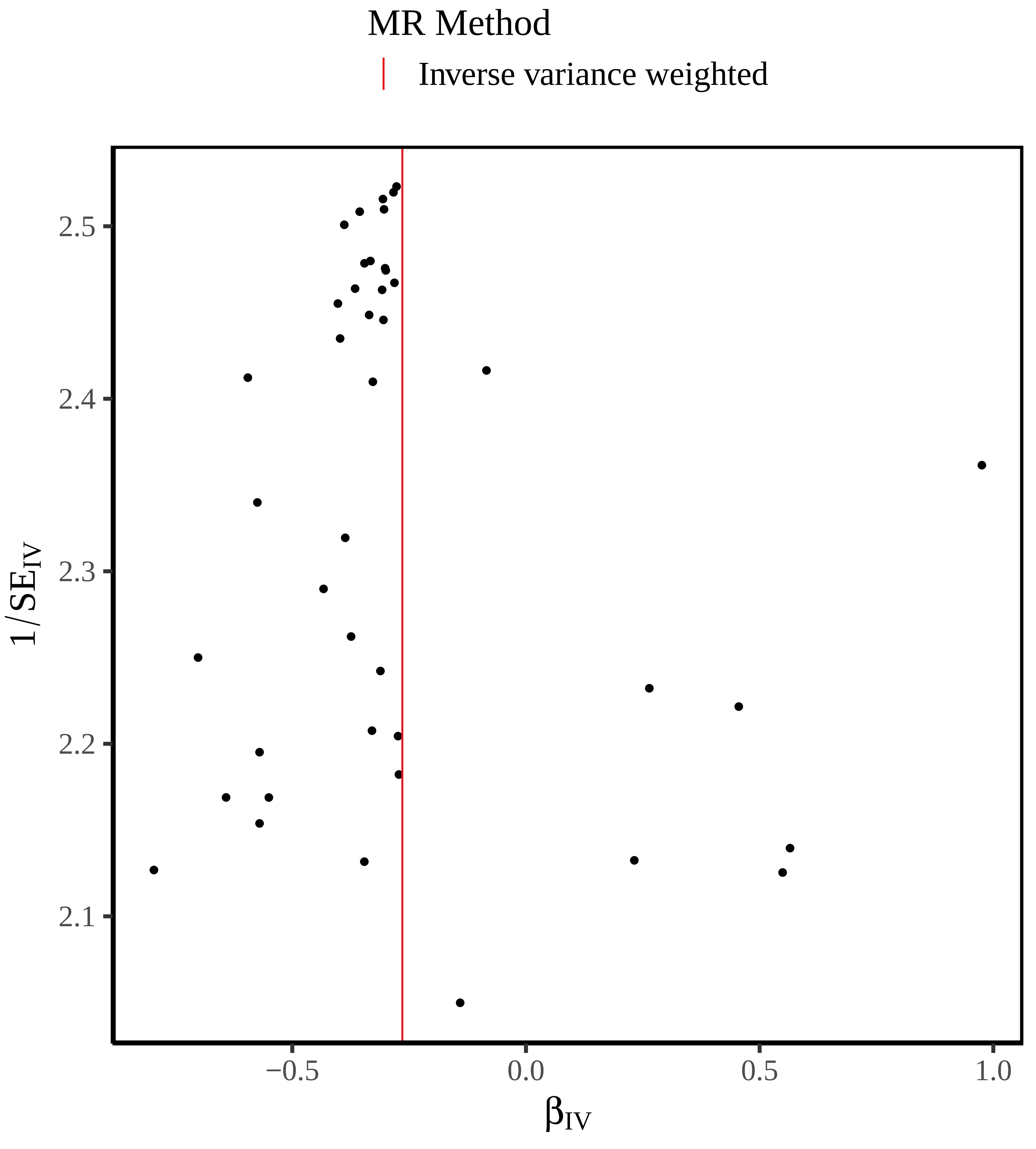
MR Method
Inverse variance weighted
2.5
2.4
2.3
2.2
2.1
1/SE_IV
−0.5
0.0
0.5
1.0
β_IV

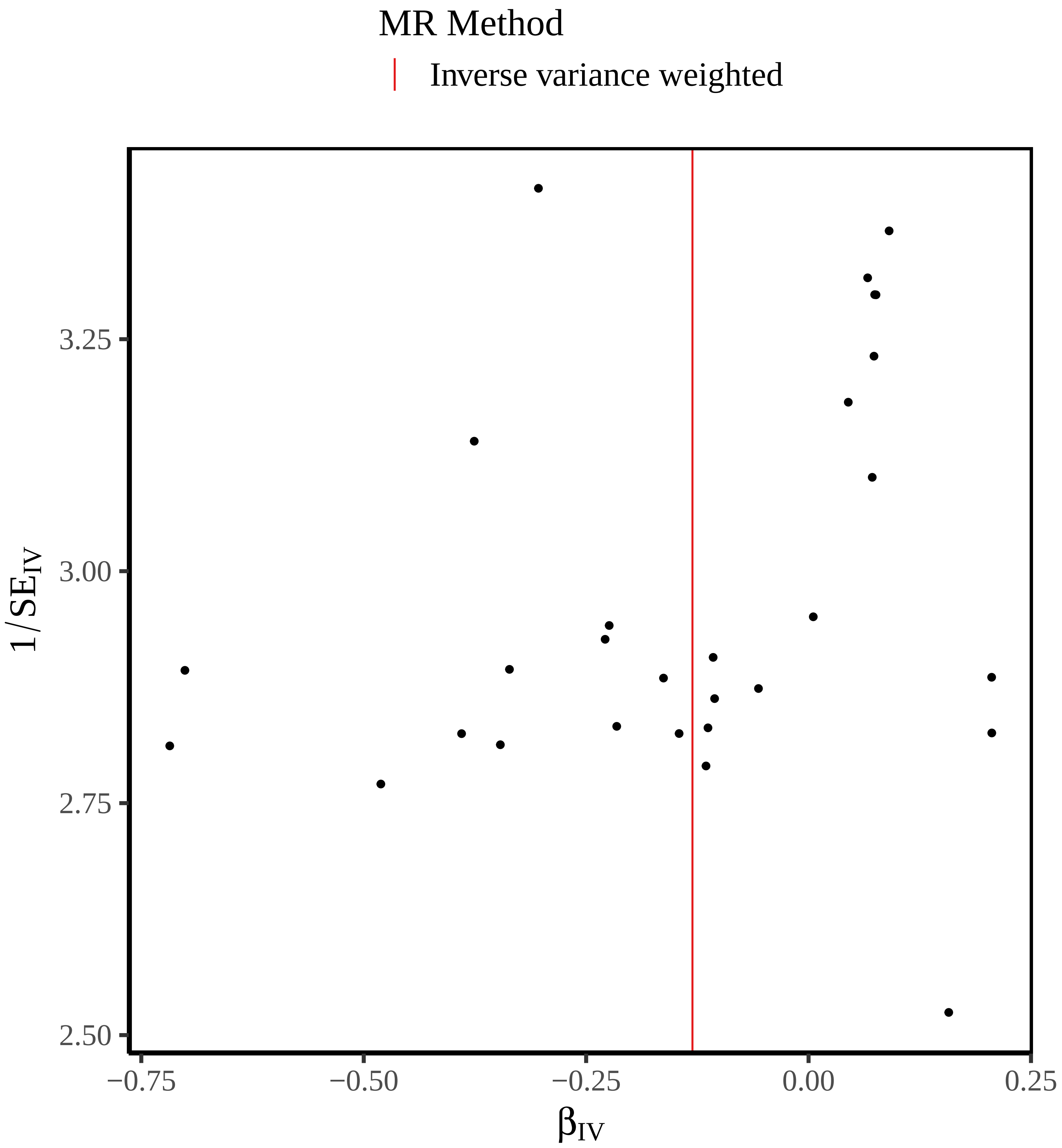

MR Method
Inverse variance weighted
3.25
3.00
2.75
2.50
1/SE$_{IV}$
−0.75
−0.50
−0.25
0.00
0.25
β$_{IV}$

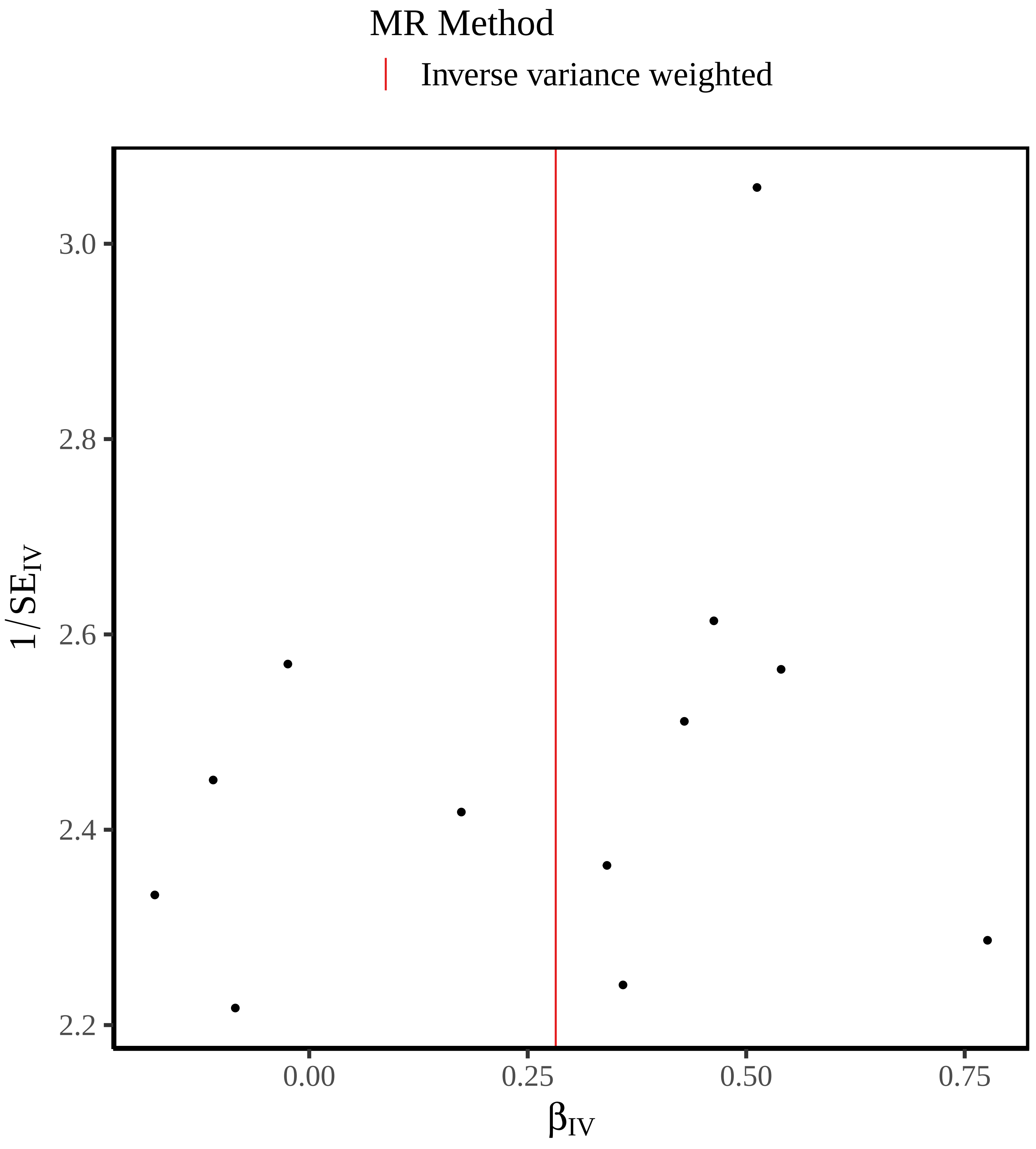
MR Method
Inverse variance weighted
1/SE_IV
3.0
2.8
2.6
2.4
2.2
0.00
0.25
0.50
0.75
β_IV

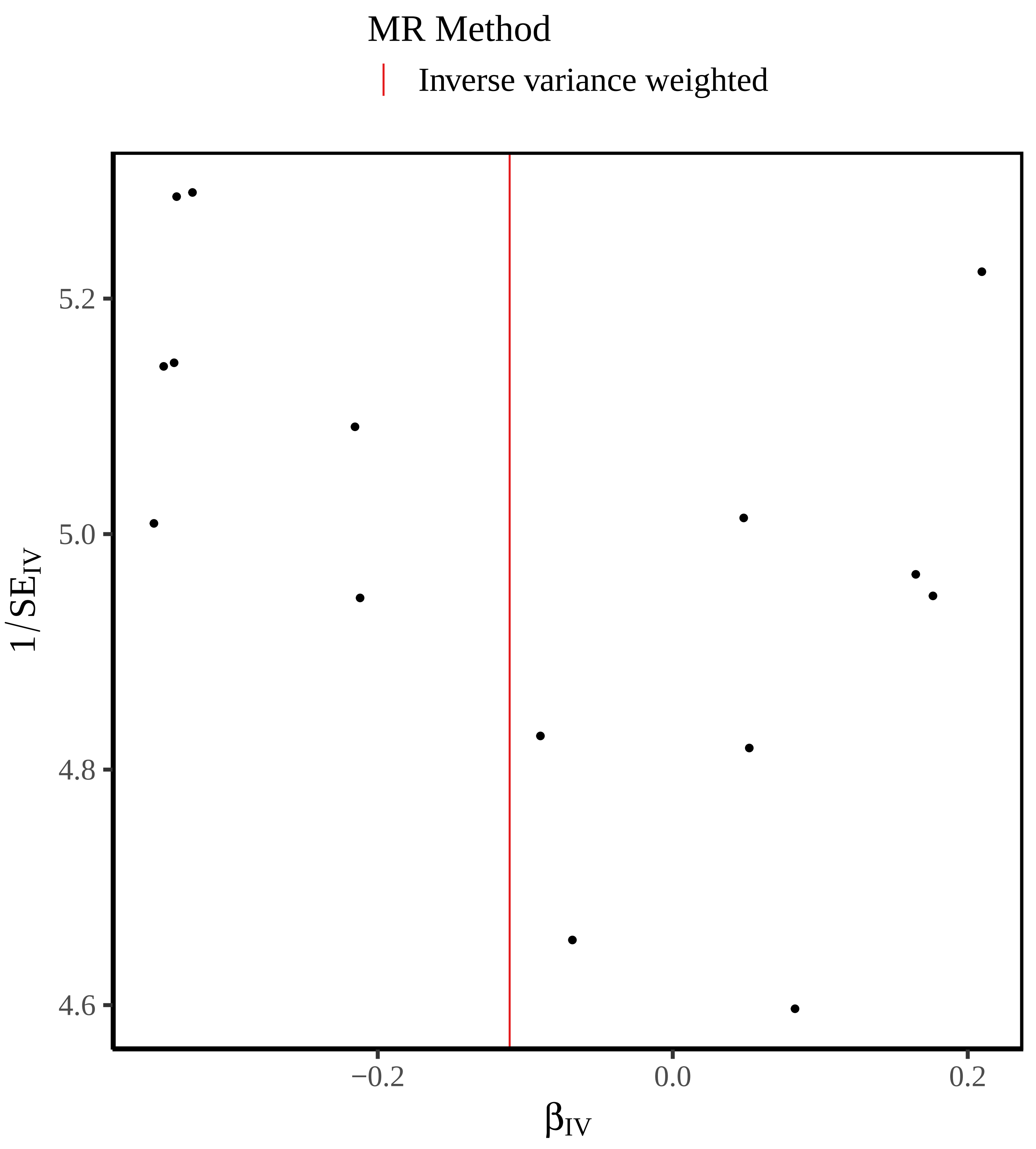
MR Method
Inverse variance weighted
5.2
5.0
4.8
4.6
1/SE$_{IV}$
−0.2
0.0
0.2
β$_{IV}$

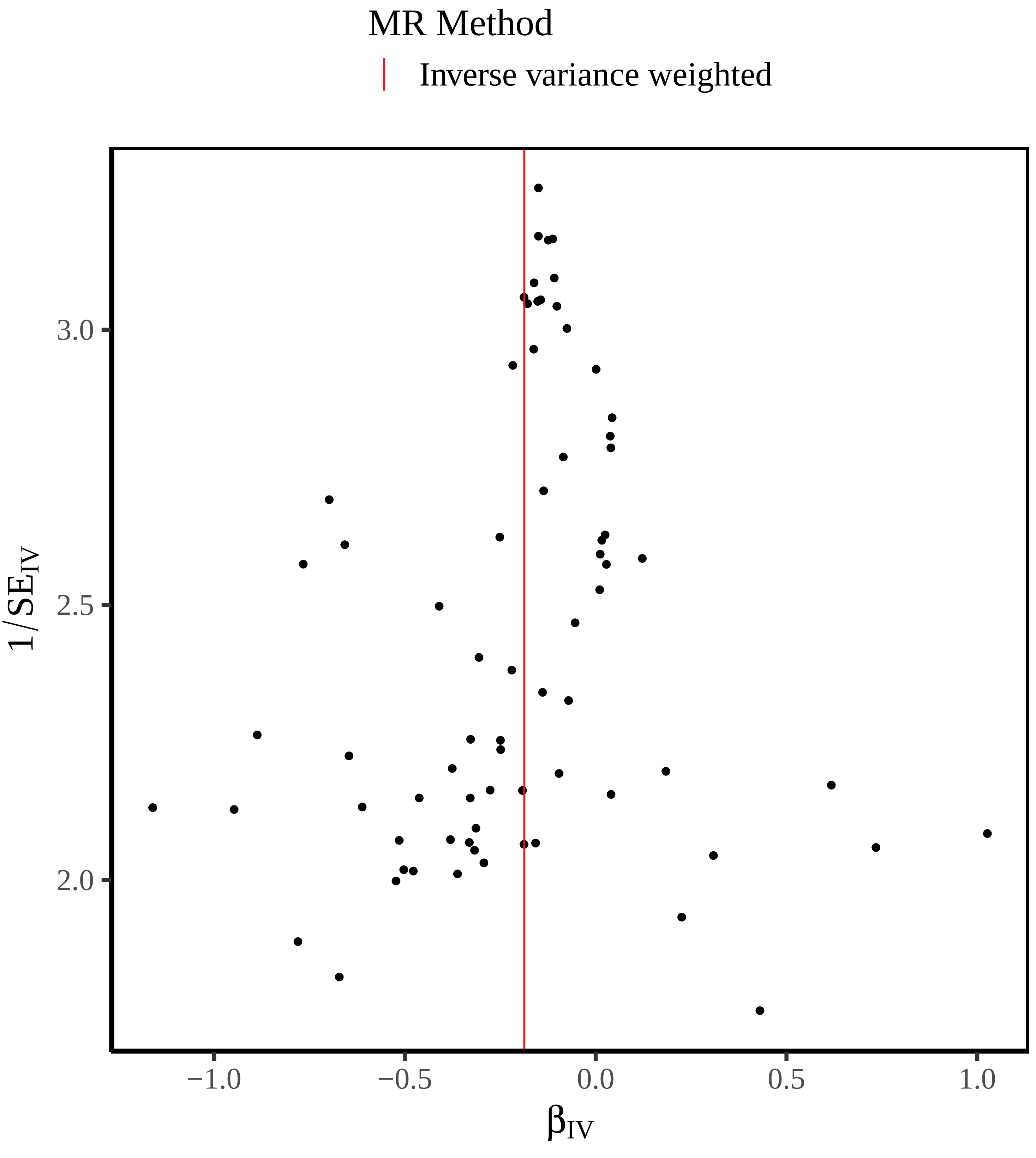

MR Method
Inverse variance weighted
1/SE$_{IV}$
3.0
2.5
2.0
−1.0
−0.5
0.0
0.5
1.0
β$_{IV}$

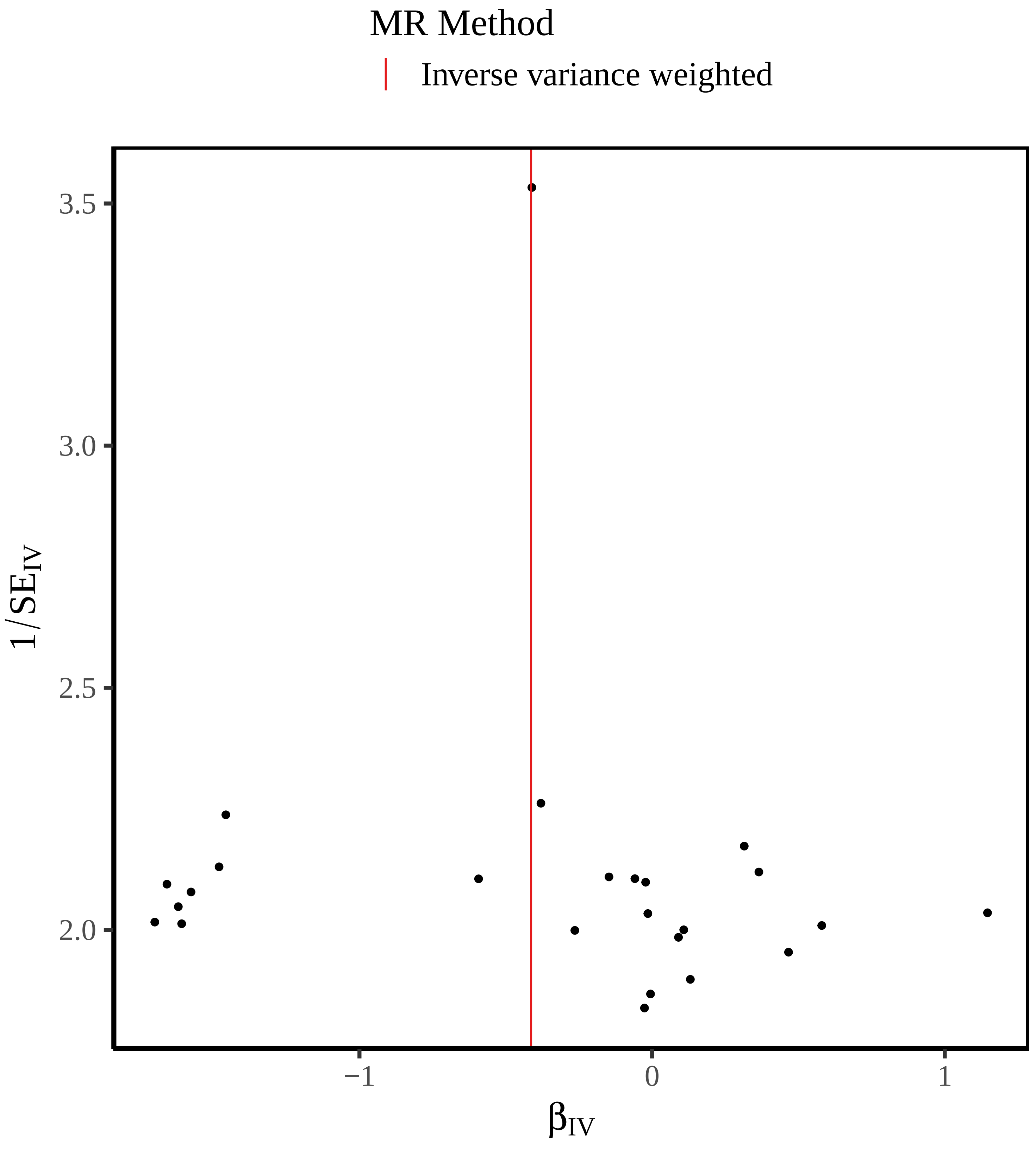
MR Method
Inverse variance weighted
3.5
3.0
2.5
2.0
$1/SE_{IV}$
−1
0
1
$\beta_{IV}$
