# Supplementary material for: Mendelian randomization integrated with multi-omics analysis identifies TNIK as a key gene in gut microbiota-induced IBD development
Source: Front Immunol. 2025 Nov 18;16:1678444. doi: 10.3389/fimmu.2025.1678444 (PMC12669205; doi:10.3389/fimmu.2025.1678444)
Supplement: Supplementary file 4 [file DataSheet4.pdf]

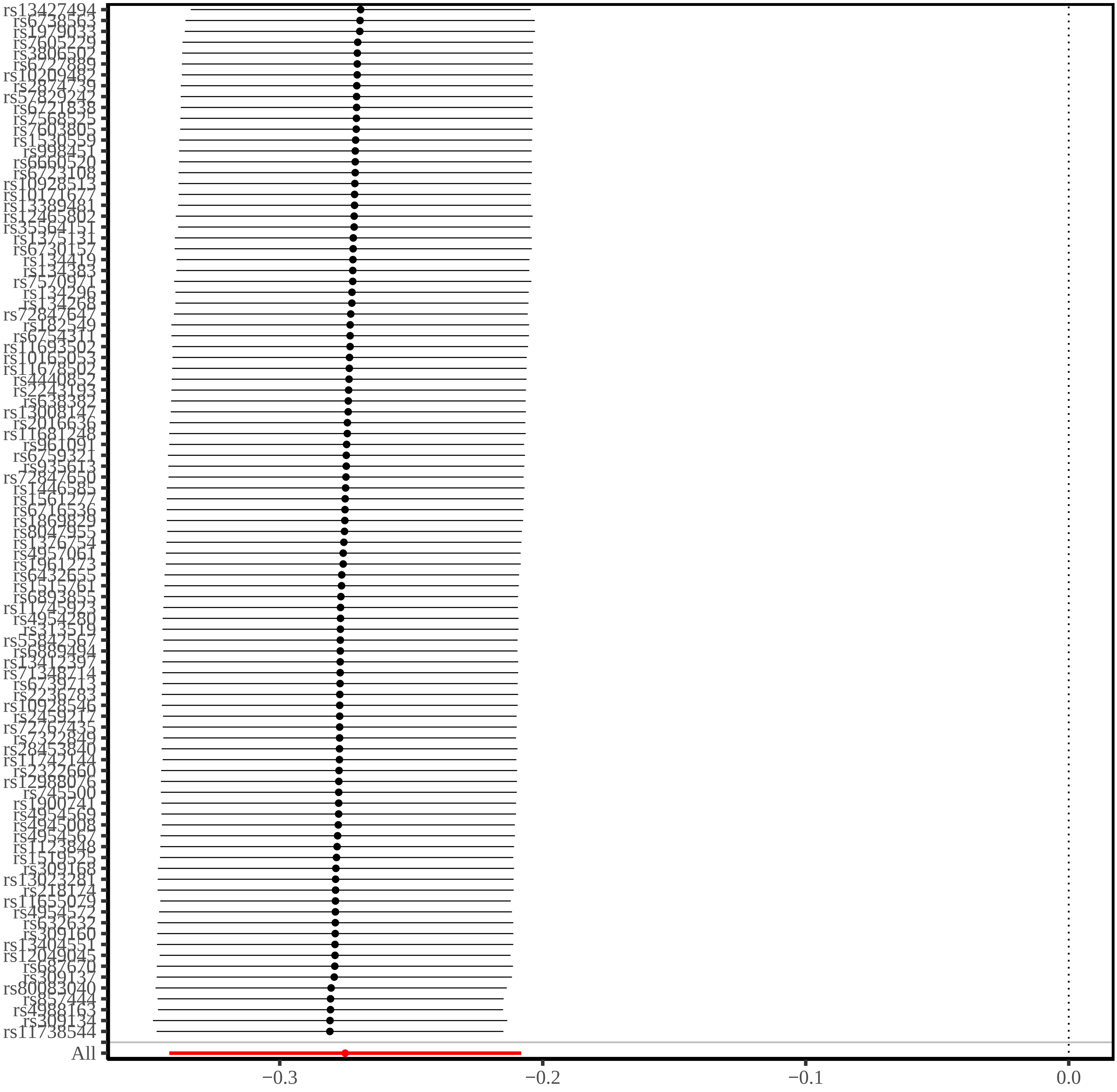

MR leave−one−out sensitivity analysis for
'class.Actinobacteria.id.419' on 'ulcerative colitis || id:ebi−a−GCST90018933'

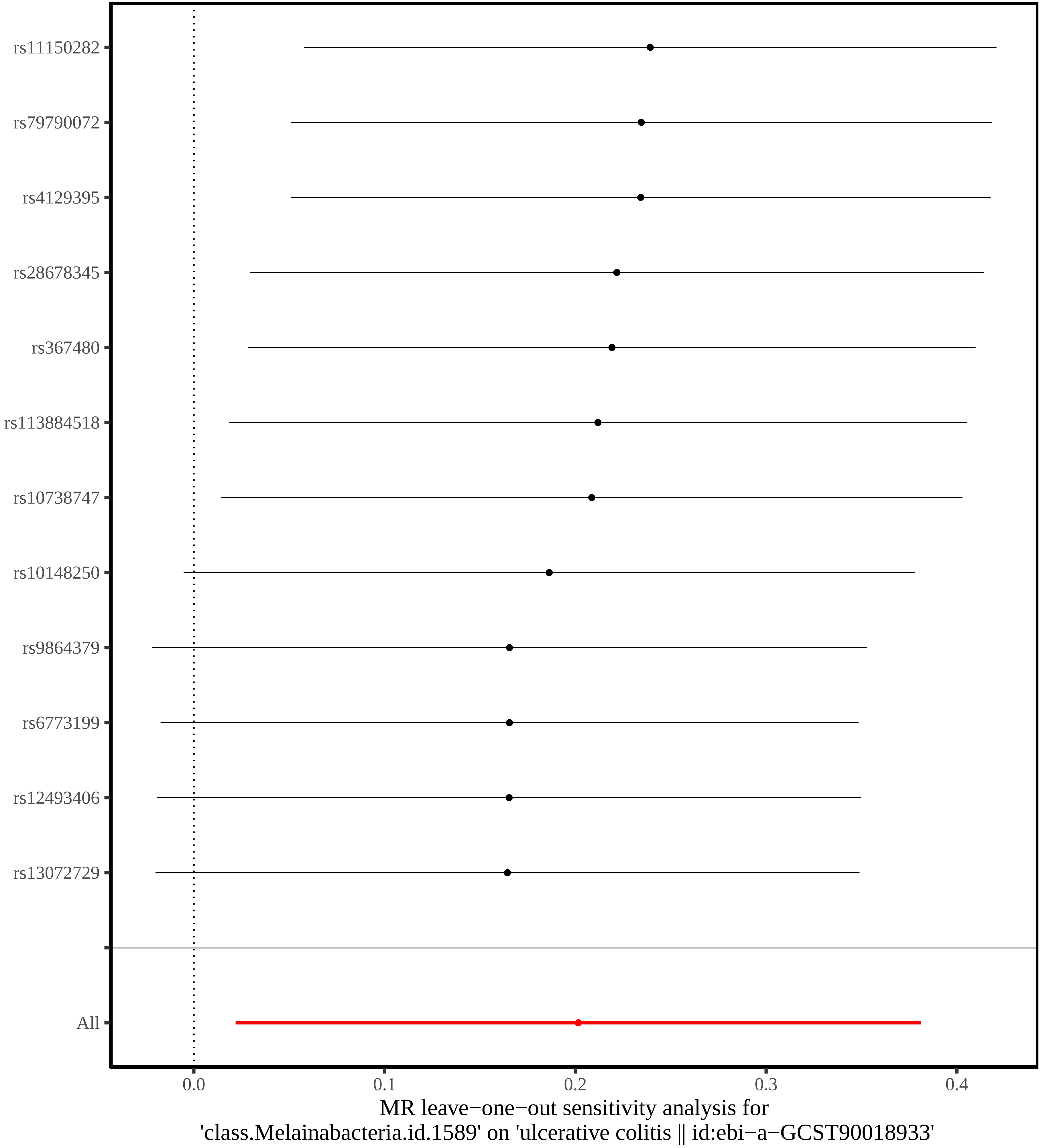
rs11150282
rs79790072
rs4129395
rs28678345
rs367480
rs113884518
rs10738747
rs10148250
rs9864379
rs6773199
rs12493406
rs13072729
All
0.0
0.1
0.2
0.3
0.4
MR leave−one−out sensitivity analysis for
'class.Melainabacteria.id.1589' on 'ulcerative colitis || id:ebi−a−GCST90018933'

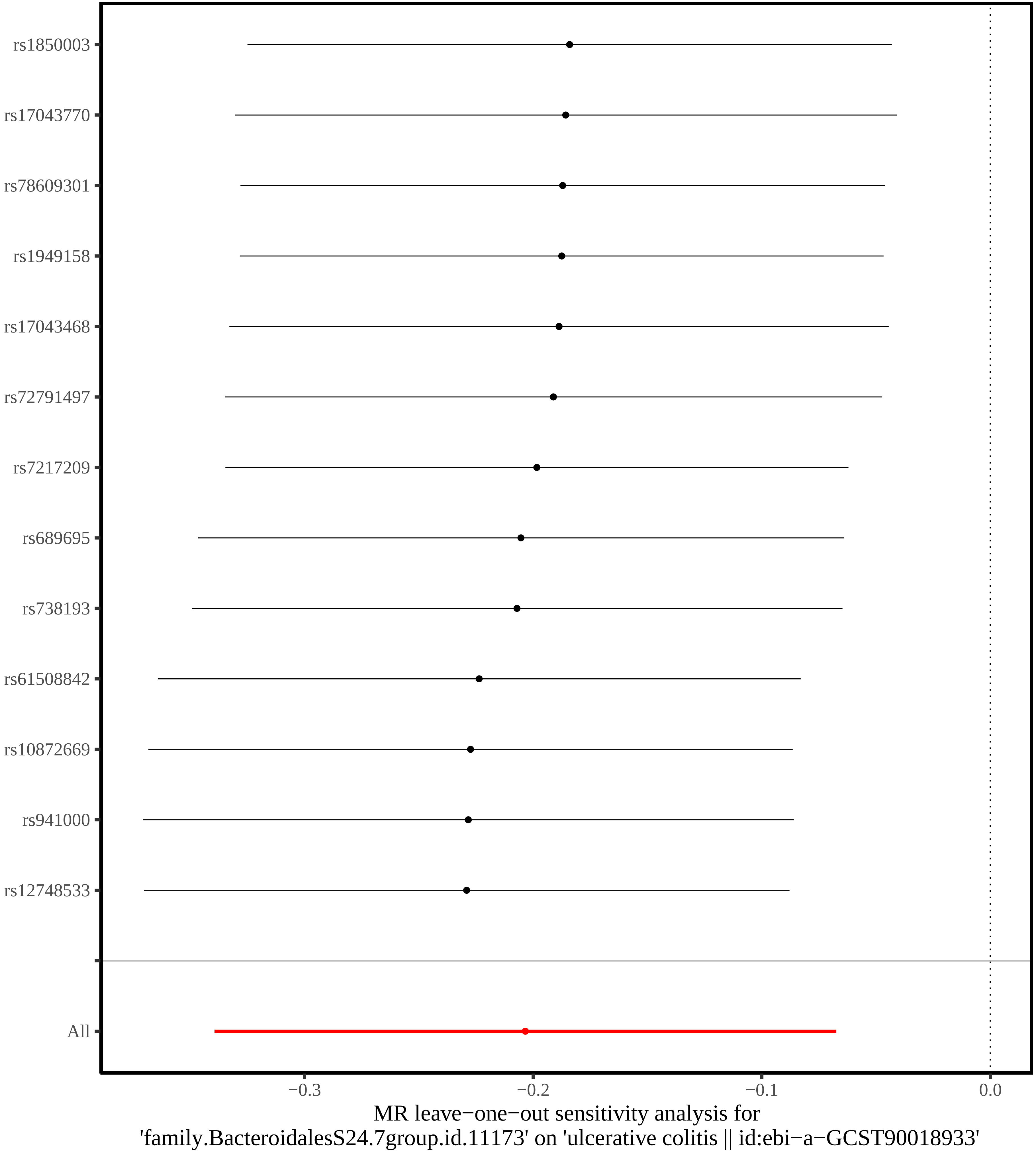
rs1850003
rs17043770
rs78609301
rs1949158
rs17043468
rs72791497
rs7217209
rs689695
rs738193
rs61508842
rs10872669
rs941000
rs12748533
All
−0.3
−0.2
−0.1
0.0
MR leave−one−out sensitivity analysis for
'family.BacteroidalesS24.7group.id.11173' on 'ulcerative colitis || id:ebi−a−GCST90018933'

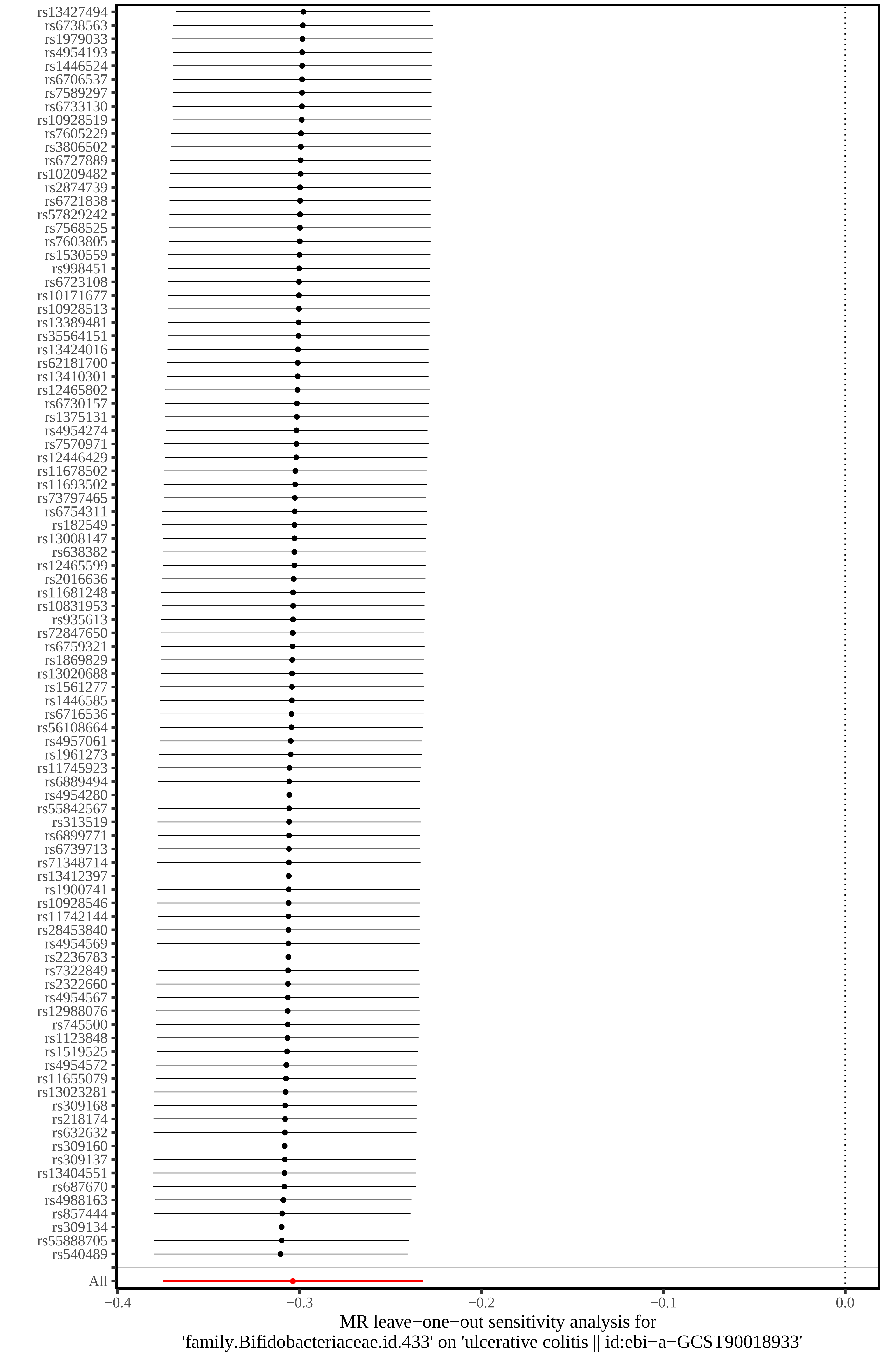
rs13427494
rs6738563
rs1979033
rs4954193
rs1446524
rs6706537
rs7589297
rs6733130
rs10928519
rs7605229
rs3806502
rs6727889
rs10209482
rs2874739
rs6721838
rs57829242
rs7568525
rs7603805
rs1530559
rs998451
rs6723108
rs10171677
rs10928513
rs13389481
rs35564151
rs13424016
rs62181700
rs13410301
rs12465802
rs6730157
rs1375131
rs4954274
rs7570971
rs12446429
rs11678502
rs11693502
rs73797465
rs6754311
rs182549
rs13008147
rs638382
rs12465599
rs2016636
rs11681248
rs10831953
rs935613
rs72847650
rs6759321
rs1869829
rs13020688
rs1561277
rs1446585
rs6716536
rs56108664
rs4957061
rs1961273
rs11745923
rs6889494
rs4954280
rs55842567
rs313519
rs6899771
rs6739713
rs71348714
rs13412397
rs1900741
rs10928546
rs11742144
rs28453840
rs4954569
rs2236783
rs7322849
rs2322660
rs4954567
rs12988076
rs745500
rs1123848
rs1519525
rs4954572
rs11655079
rs13023281
rs309168
rs218174
rs632632
rs309160
rs309137
rs13404551
rs687670
rs4988163
rs857444
rs309134
rs55888705
rs540489
All
−0.4
−0.3
−0.2
−0.1
0.0
MR leave−one−out sensitivity analysis for
'family.Bifidobacteriaceae.id.433' on 'ulcerative colitis || id:ebi−a−GCST90018933'

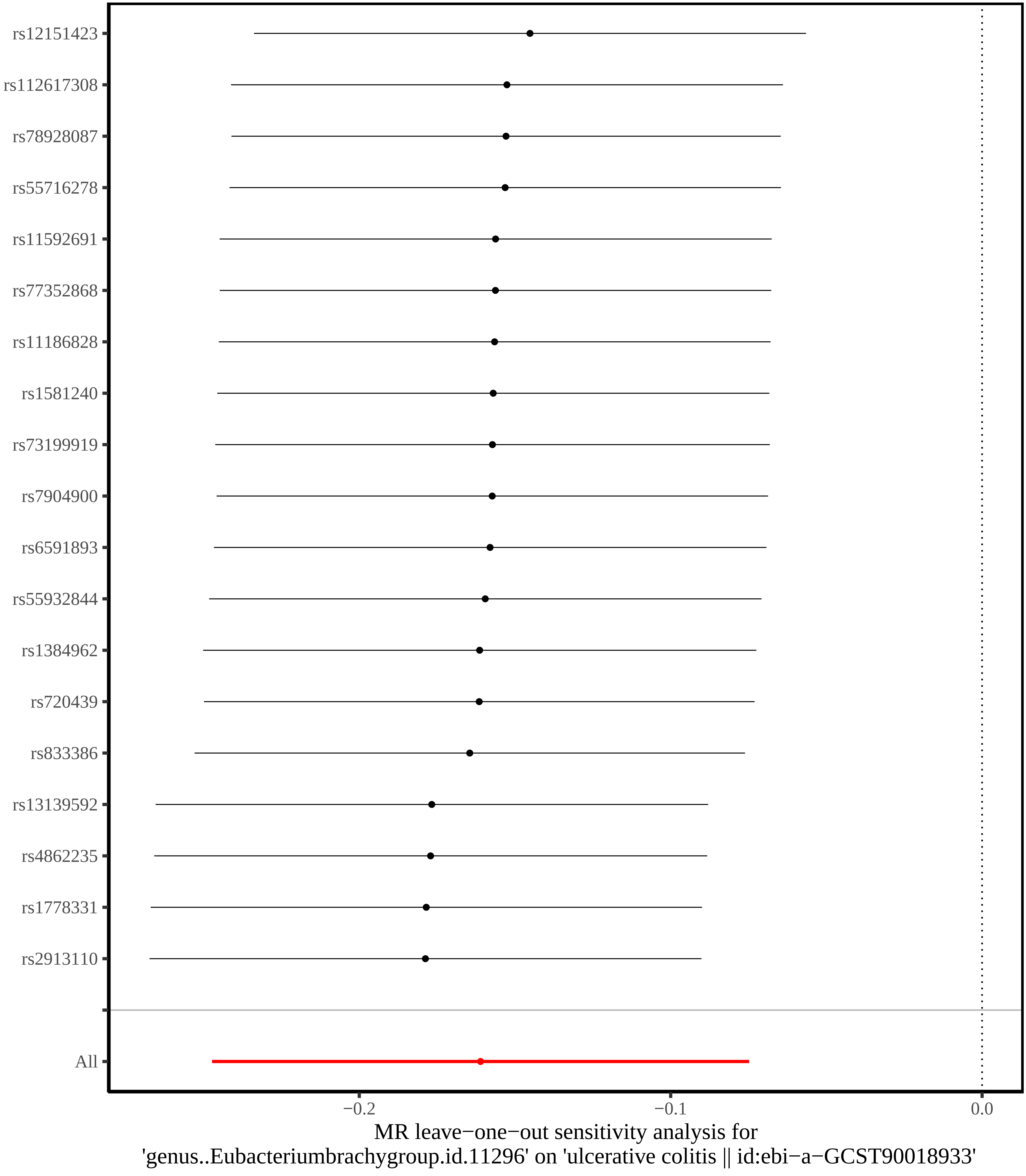
rs12151423
rs112617308
rs78928087
rs55716278
rs11592691
rs77352868
rs11186828
rs1581240
rs73199919
rs7904900
rs6591893
rs55932844
rs1384962
rs720439
rs833386
rs13139592
rs4862235
rs1778331
rs2913110
All
−0.2
−0.1
0.0
MR leave−one−out sensitivity analysis for
'genus..Eubacteriumbrachygroup.id.11296' on 'ulcerative colitis || id:ebi−a−GCST90018933'

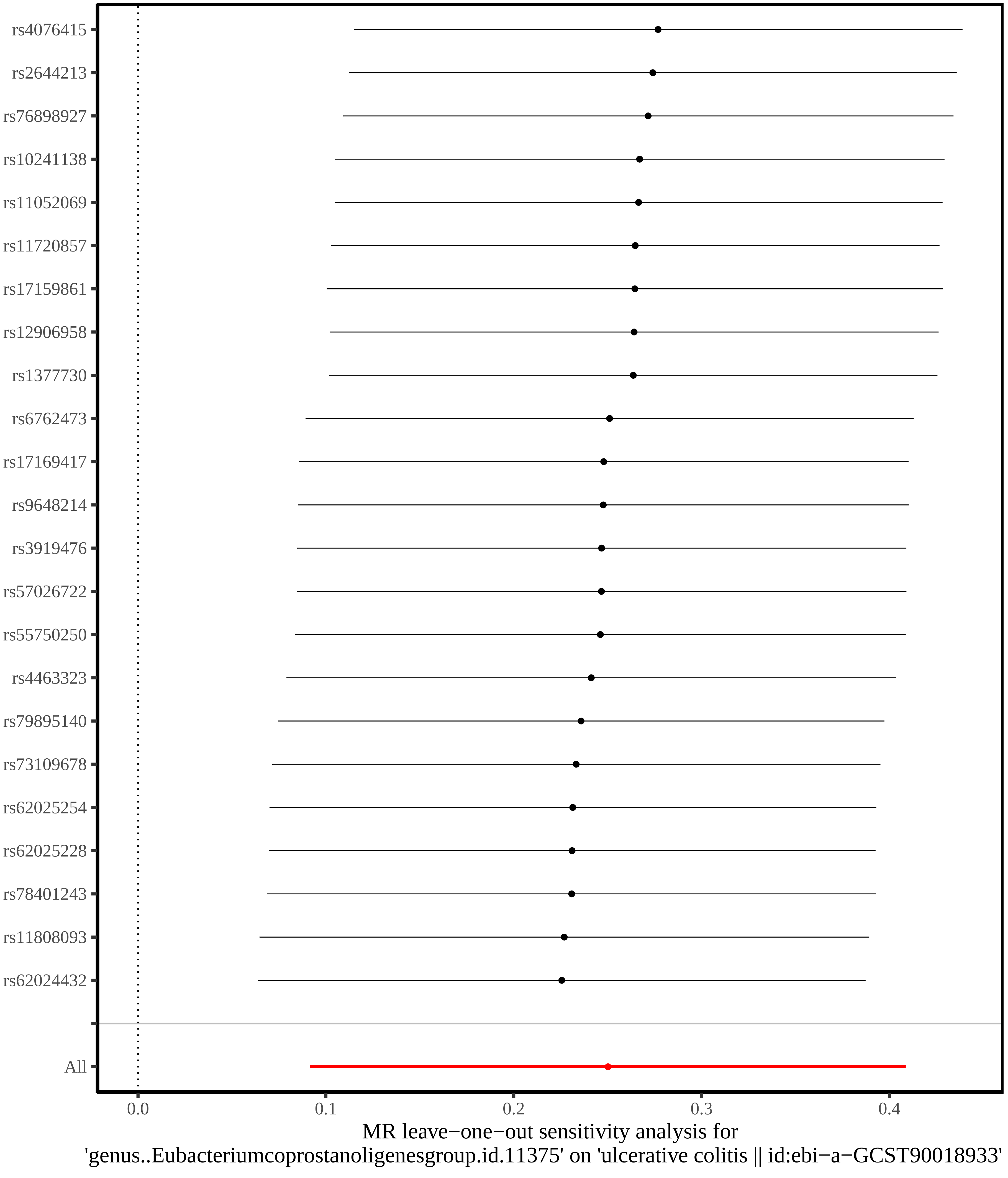
rs4076415
rs2644213
rs76898927
rs10241138
rs11052069
rs11720857
rs17159861
rs12906958
rs1377730
rs6762473
rs17169417
rs9648214
rs3919476
rs57026722
rs55750250
rs4463323
rs79895140
rs73109678
rs62025254
rs62025228
rs78401243
rs11808093
rs62024432
All
0.0
0.1
0.2
0.3
0.4
MR leave−one−out sensitivity analysis for
'genus..Eubacteriumcoprostanoligenesgroup.id.11375' on 'ulcerative colitis || id:ebi−a−GCST90018933'

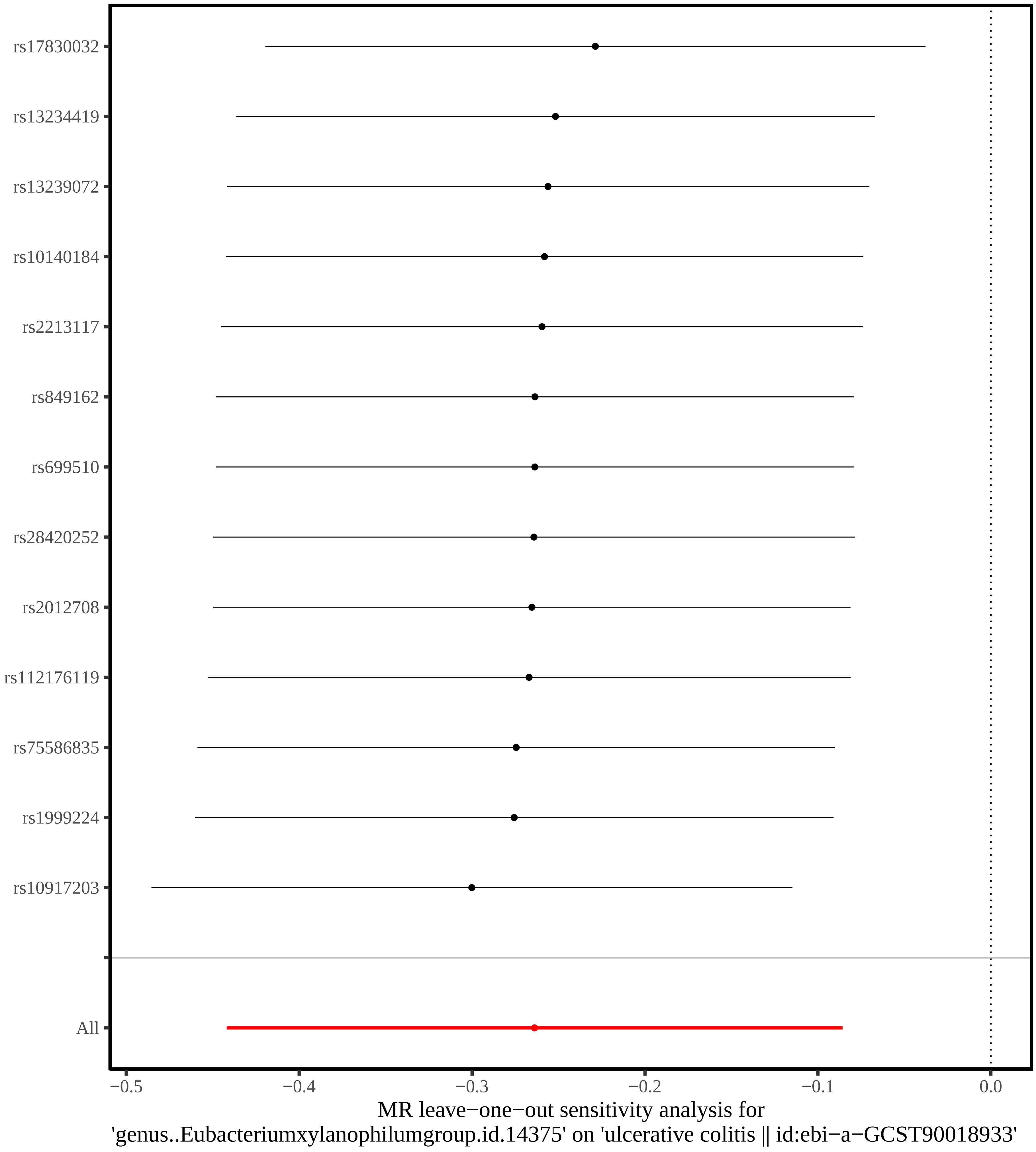

rs17830032
rs13234419
rs13239072
rs10140184
rs2213117
rs849162
rs699510
rs28420252
rs2012708
rs112176119
rs75586835
rs1999224
rs10917203
All
−0.5
−0.4
−0.3
−0.2
−0.1
0.0
MR leave−one−out sensitivity analysis for
'genus..Eubacteriumxylanophilumgroup.id.14375' on 'ulcerative colitis || id:ebi−a−GCST90018933'

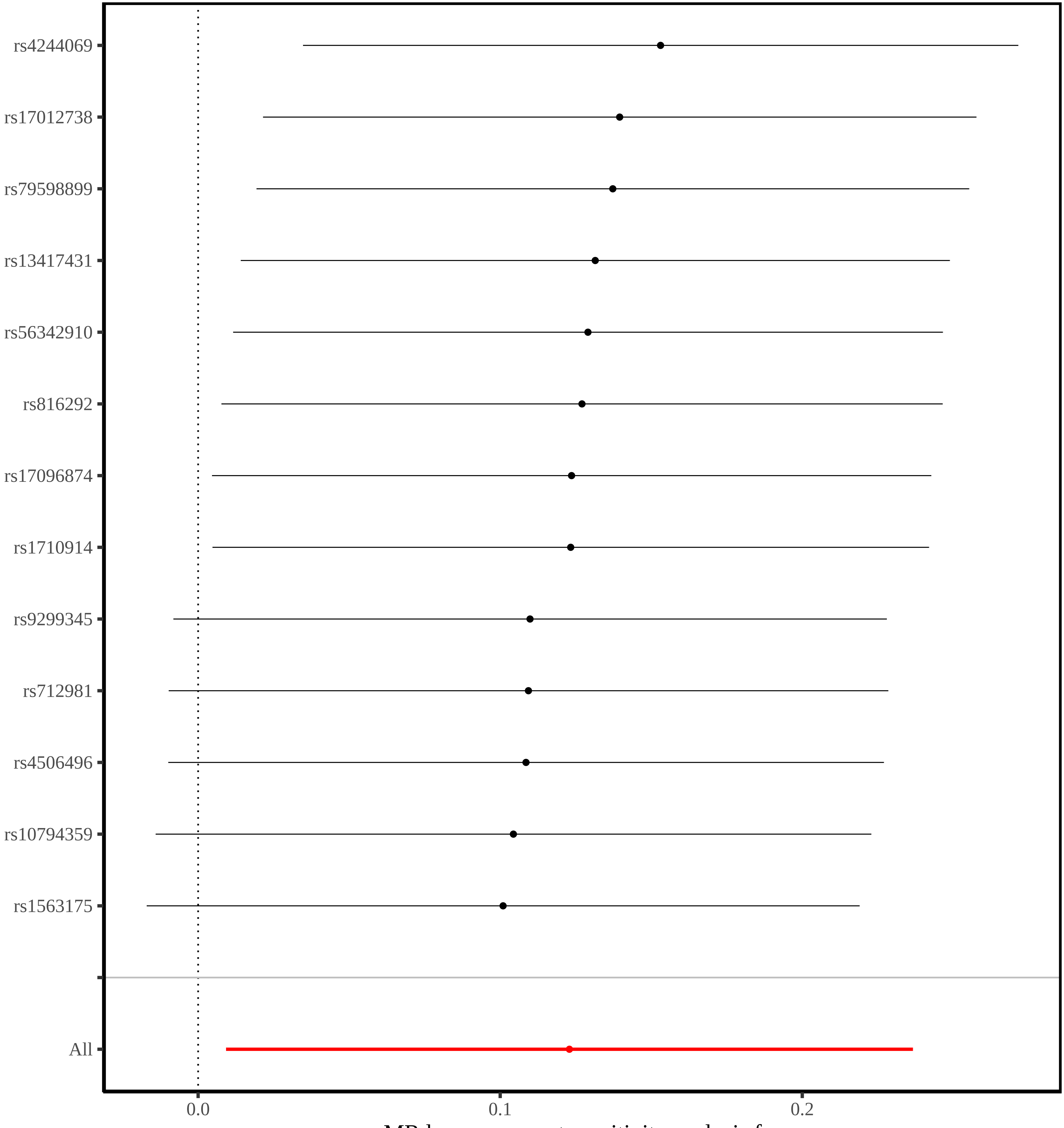

MR leave−one−out sensitivity analysis for
'genus.Anaerofilum.id.2053' on 'ulcerative colitis || id:ebi−a−GCST90018933'

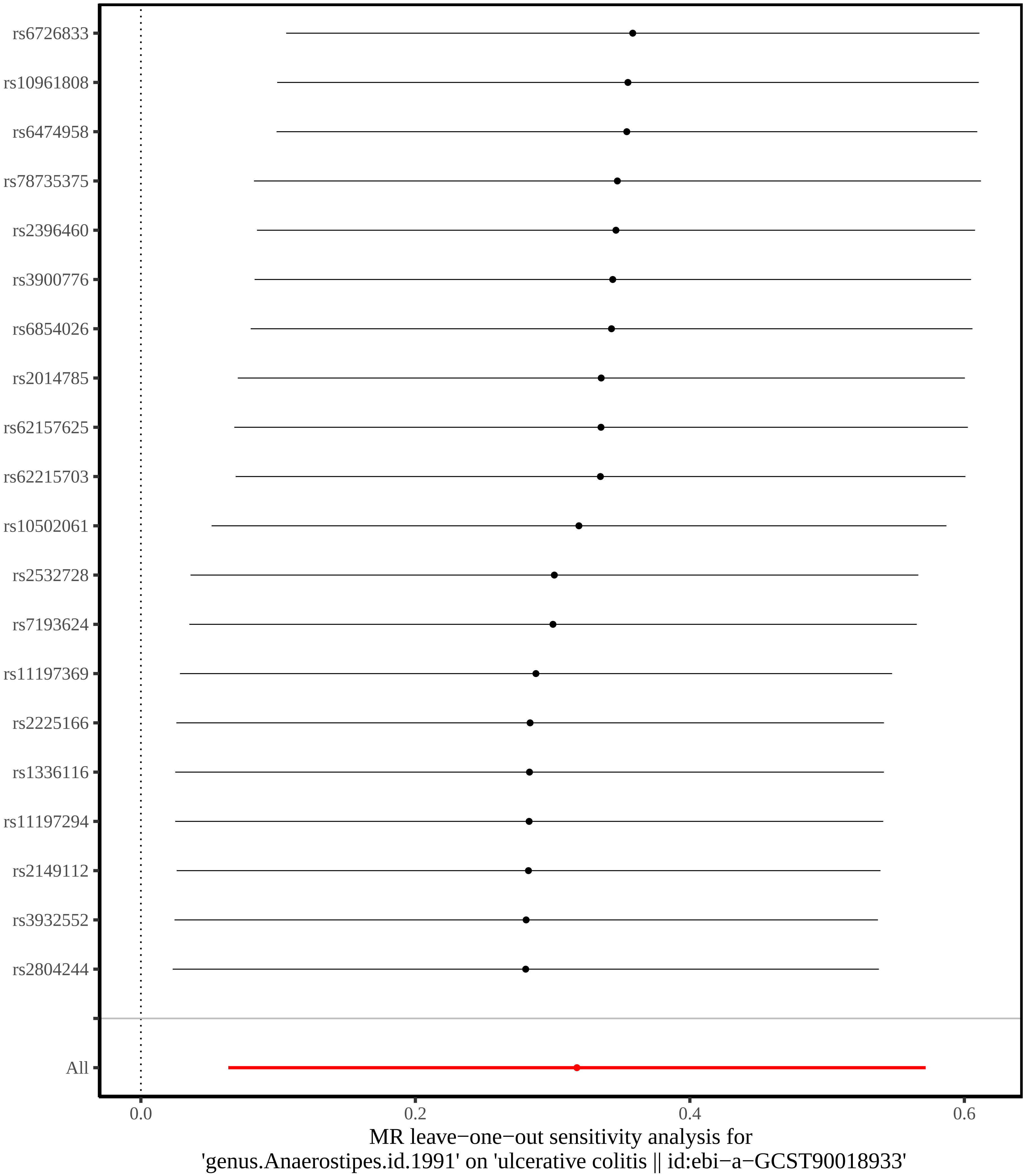
rs6726833
rs10961808
rs6474958
rs78735375
rs2396460
rs3900776
rs6854026
rs2014785
rs62157625
rs62215703
rs10502061
rs2532728
rs7193624
rs11197369
rs2225166
rs1336116
rs11197294
rs2149112
rs3932552
rs2804244
All
0.0
0.2
0.4
0.6
MR leave−one−out sensitivity analysis for
'genus.Anaerostipes.id.1991' on 'ulcerative colitis || id:ebi−a−GCST90018933'

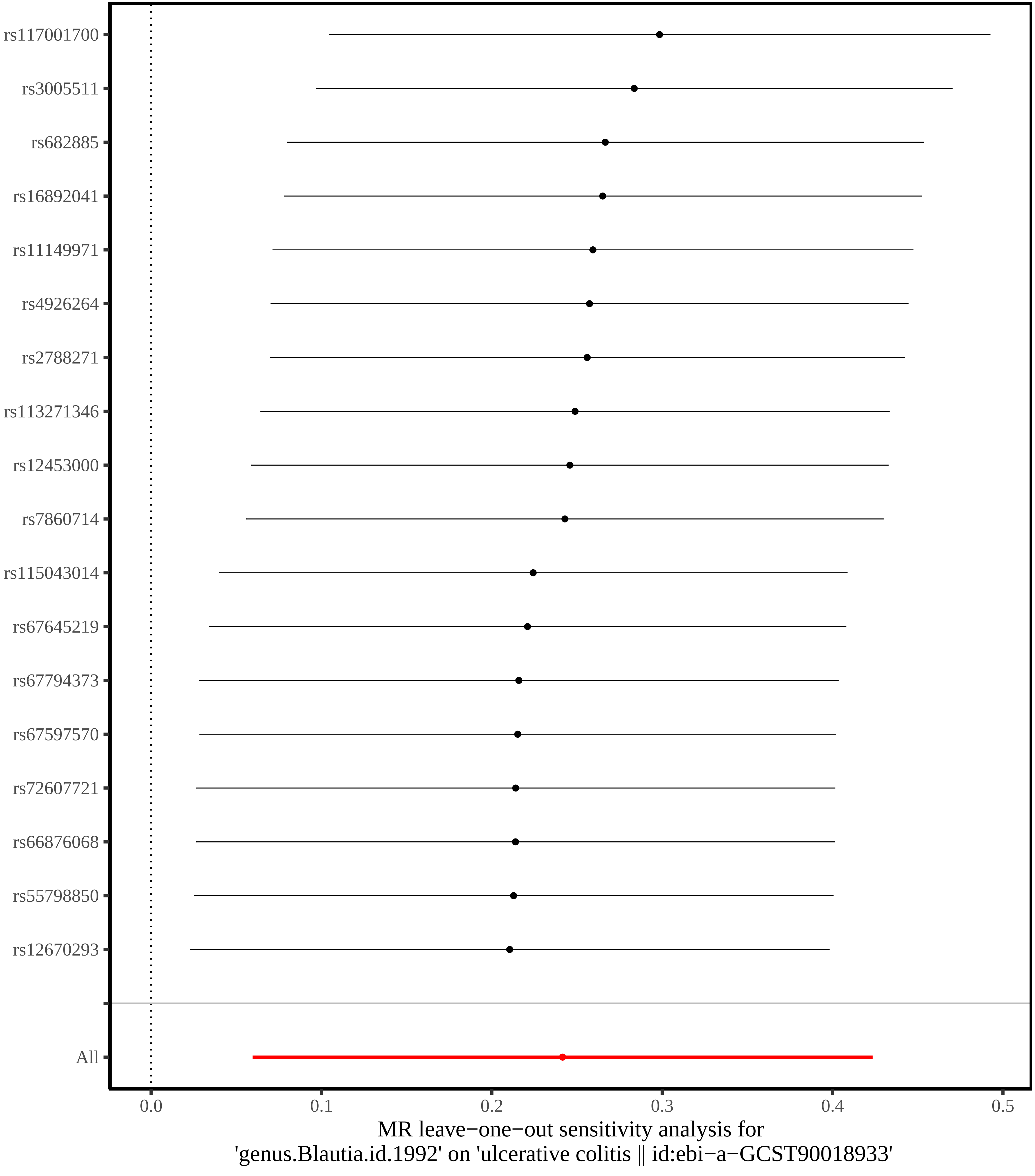
rs117001700
rs3005511
rs682885
rs16892041
rs11149971
rs4926264
rs2788271
rs113271346
rs12453000
rs7860714
rs115043014
rs67645219
rs67794373
rs67597570
rs72607721
rs66876068
rs55798850
rs12670293
All
0.0
0.1
0.2
0.3
0.4
0.5
MR leave−one−out sensitivity analysis for
'genus.Blautia.id.1992' on 'ulcerative colitis || id:ebi−a−GCST90018933'

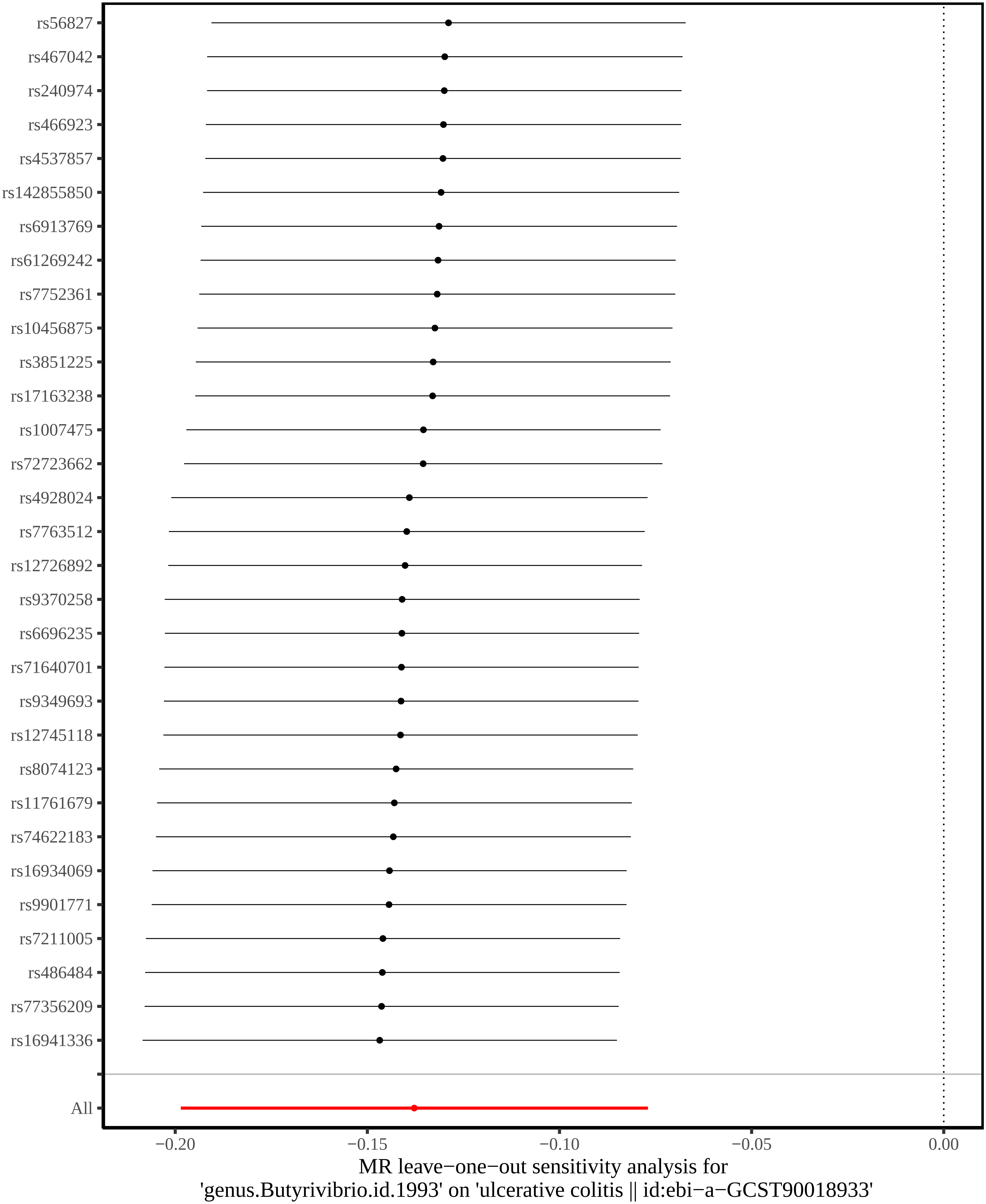
rs56827
rs467042
rs240974
rs466923
rs4537857
rs142855850
rs6913769
rs61269242
rs7752361
rs10456875
rs3851225
rs17163238
rs1007475
rs72723662
rs4928024
rs7763512
rs12726892
rs9370258
rs6696235
rs71640701
rs9349693
rs12745118
rs8074123
rs11761679
rs74622183
rs16934069
rs9901771
rs7211005
rs486484
rs77356209
rs16941336
All
−0.20
−0.15
−0.10
−0.05
0.00
MR leave−one−out sensitivity analysis for
'genus.Butyrivibrio.id.1993' on 'ulcerative colitis || id:ebi−a−GCST90018933'

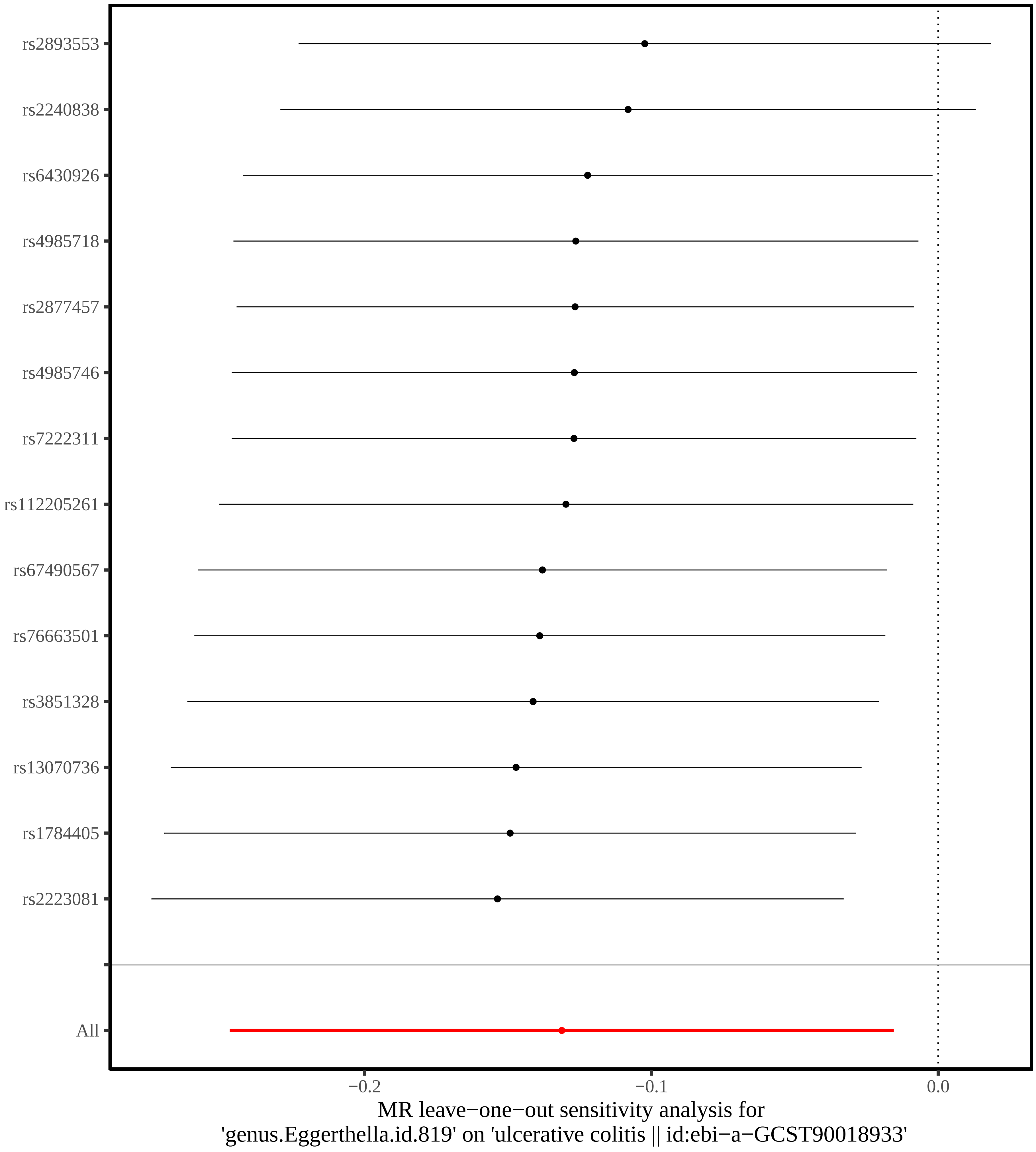
rs2893553
rs2240838
rs6430926
rs4985718
rs2877457
rs4985746
rs7222311
rs112205261
rs67490567
rs76663501
rs3851328
rs13070736
rs1784405
rs2223081
All
−0.2
−0.1
0.0
MR leave−one−out sensitivity analysis for
'genus.Eggerthella.id.819' on 'ulcerative colitis || id:ebi−a−GCST90018933'

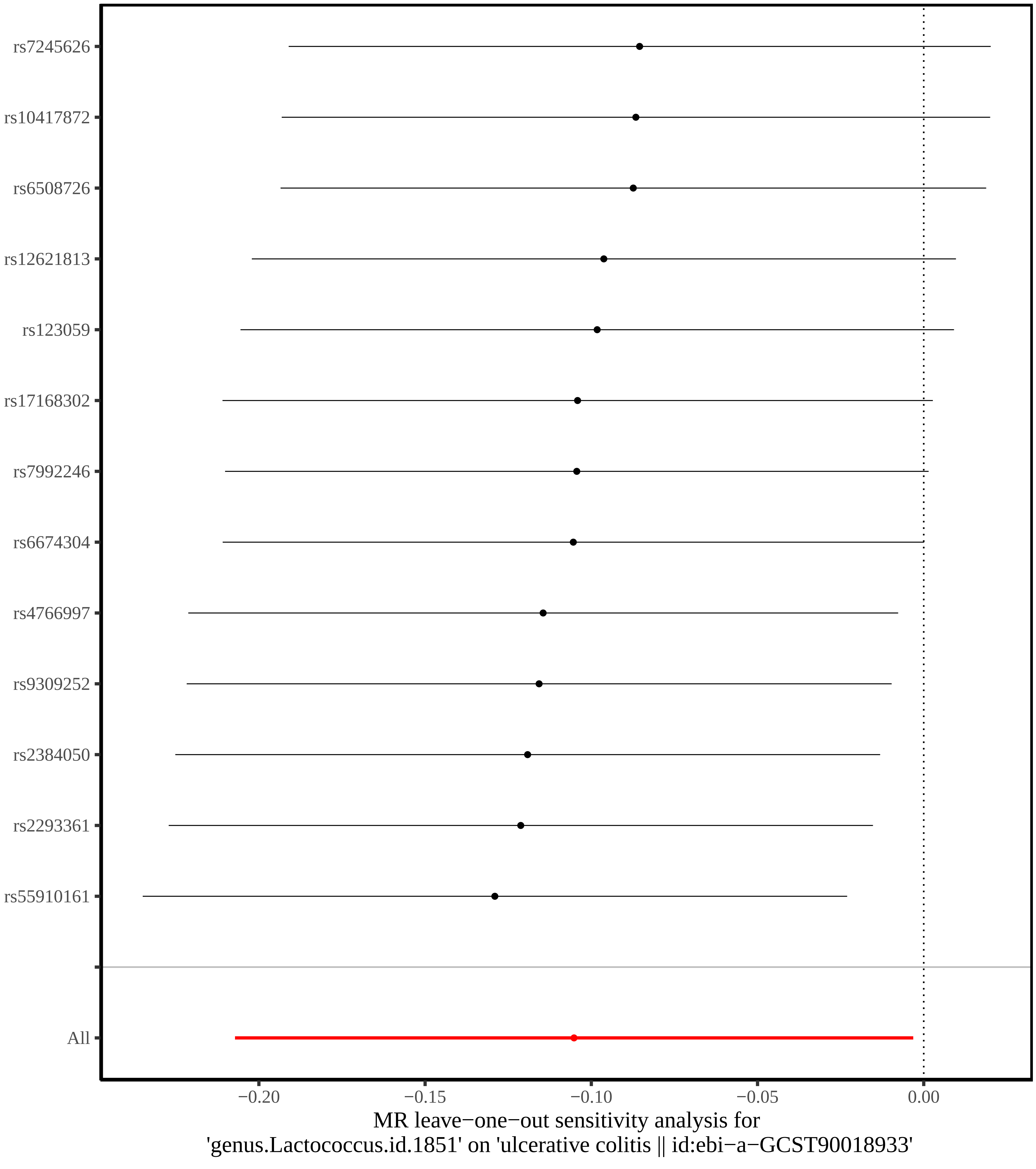

rs7245626
rs10417872
rs6508726
rs12621813
rs123059
rs17168302
rs7992246
rs6674304
rs4766997
rs9309252
rs2384050
rs2293361
rs55910161
All
−0.20
−0.15
−0.10
−0.05
0.00
MR leave−one−out sensitivity analysis for
'genus.Lactococcus.id.1851' on 'ulcerative colitis || id:ebi−a−GCST90018933'

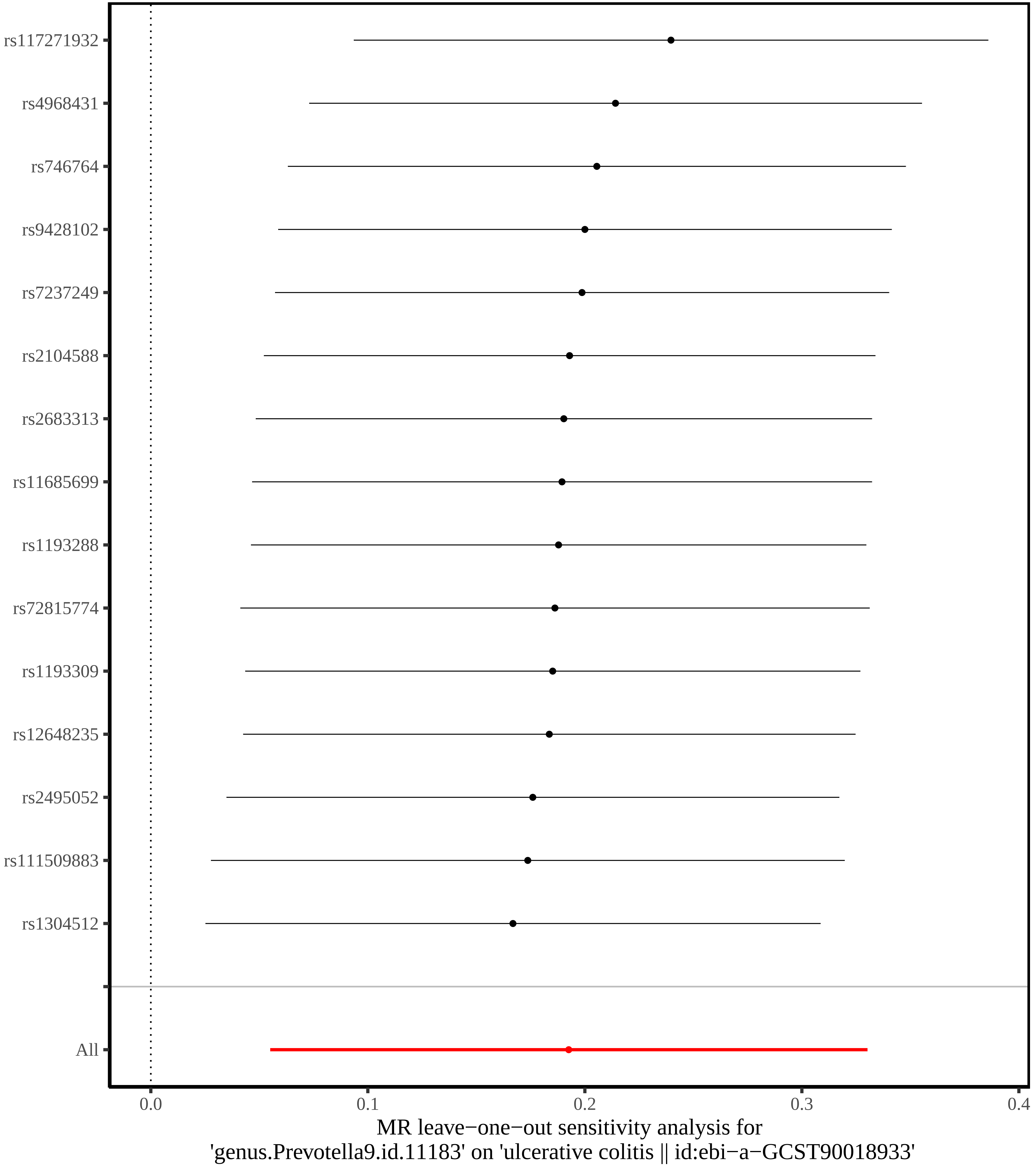
rs117271932
rs4968431
rs746764
rs9428102
rs7237249
rs2104588
rs2683313
rs11685699
rs1193288
rs72815774
rs1193309
rs12648235
rs2495052
rs111509883
rs1304512
All
0.0
0.1
0.2
0.3
0.4
MR leave−one−out sensitivity analysis for
'genus.Prevotella9.id.11183' on 'ulcerative colitis || id:ebi−a−GCST90018933'

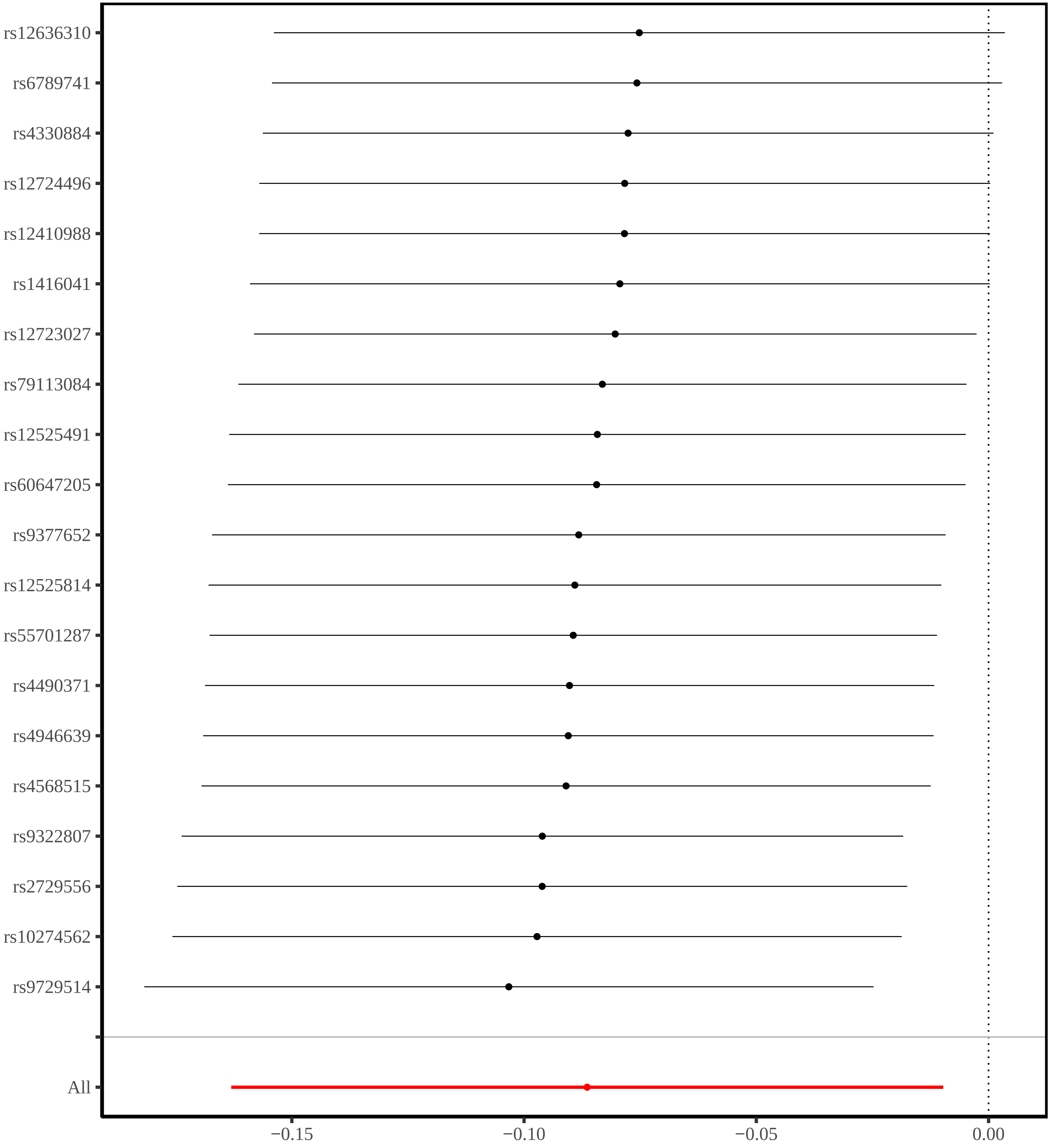

MR leave−one−out sensitivity analysis for
'genus.RuminococcaceaeUCG011.id.11368' on 'ulcerative colitis || id:ebi−a−GCST90018933'

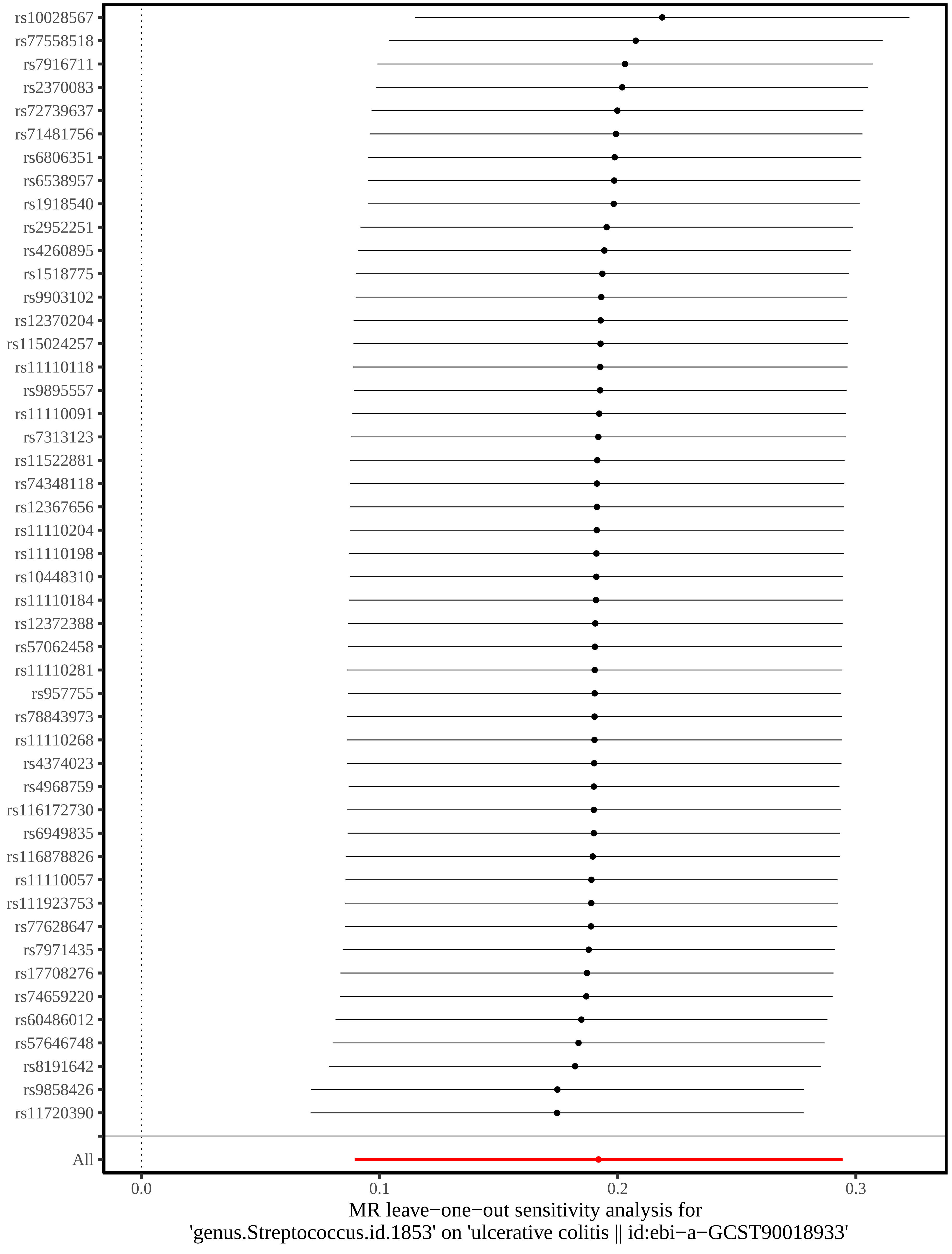
rs10028567
rs77558518
rs7916711
rs2370083
rs72739637
rs71481756
rs6806351
rs6538957
rs1918540
rs2952251
rs4260895
rs1518775
rs9903102
rs12370204
rs115024257
rs11110118
rs9895557
rs11110091
rs7313123
rs11522881
rs74348118
rs12367656
rs11110204
rs11110198
rs10448310
rs11110184
rs12372388
rs57062458
rs11110281
rs957755
rs78843973
rs11110268
rs4374023
rs4968759
rs116172730
rs6949835
rs116878826
rs11110057
rs111923753
rs77628647
rs7971435
rs17708276
rs74659220
rs60486012
rs57646748
rs8191642
rs9858426
rs11720390
All
0.0
0.1
0.2
0.3
MR leave−one−out sensitivity analysis for
'genus.Streptococcus.id.1853' on 'ulcerative colitis || id:ebi−a−GCST90018933'

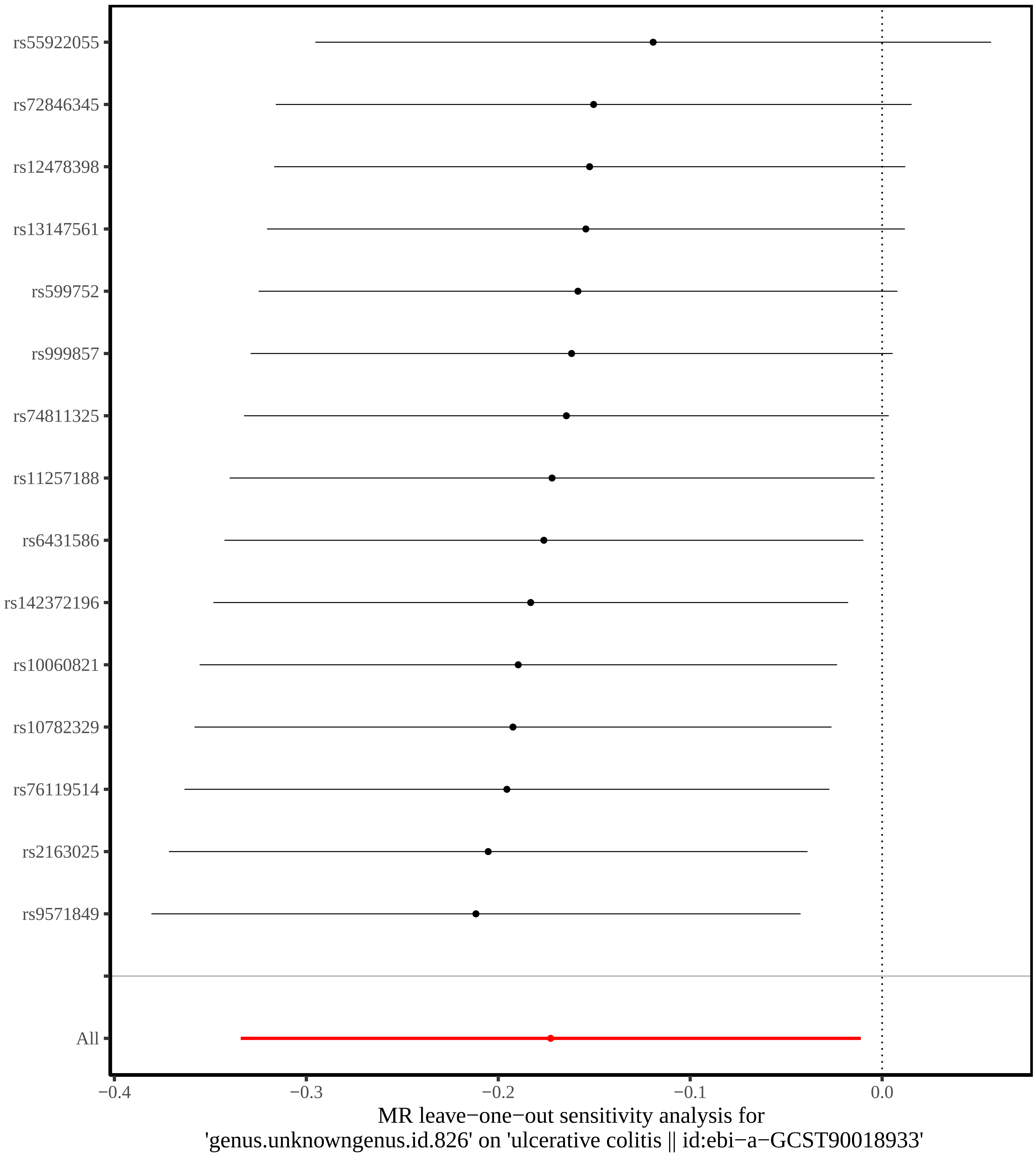

rs55922055
rs72846345
rs12478398
rs13147561
rs599752
rs999857
rs74811325
rs11257188
rs6431586
rs142372196
rs10060821
rs10782329
rs76119514
rs2163025
rs9571849
All
−0.4
−0.3
−0.2
−0.1
0.0
MR leave−one−out sensitivity analysis for
'genus.unknowngenus.id.826' on 'ulcerative colitis || id:ebi−a−GCST90018933'

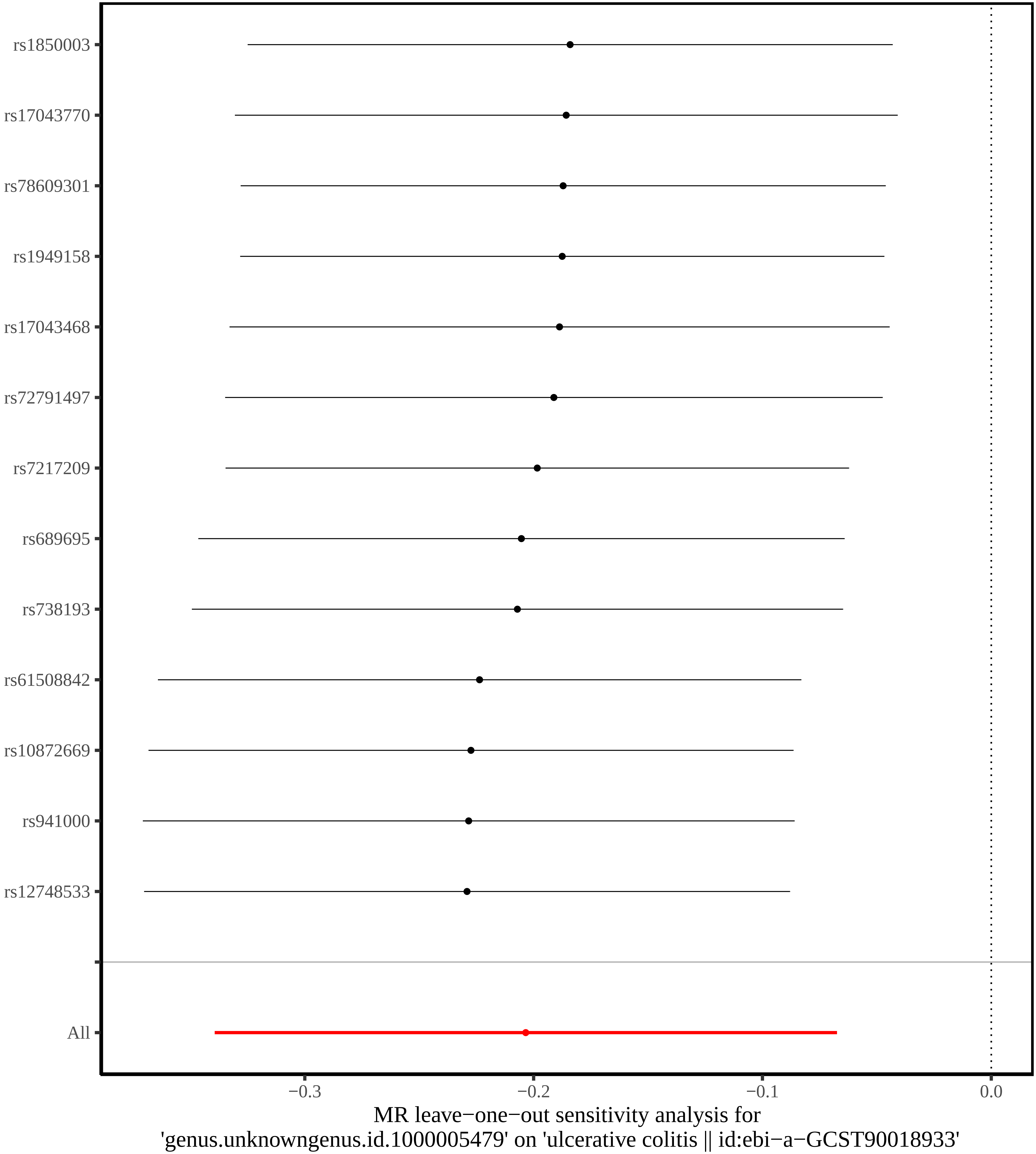

rs1850003
rs17043770
rs78609301
rs1949158
rs17043468
rs72791497
rs7217209
rs689695
rs738193
rs61508842
rs10872669
rs941000
rs12748533
All
−0.3
−0.2
−0.1
0.0
MR leave−one−out sensitivity analysis for
'genus.unknowngenus.id.1000005479' on 'ulcerative colitis || id:ebi−a−GCST90018933'

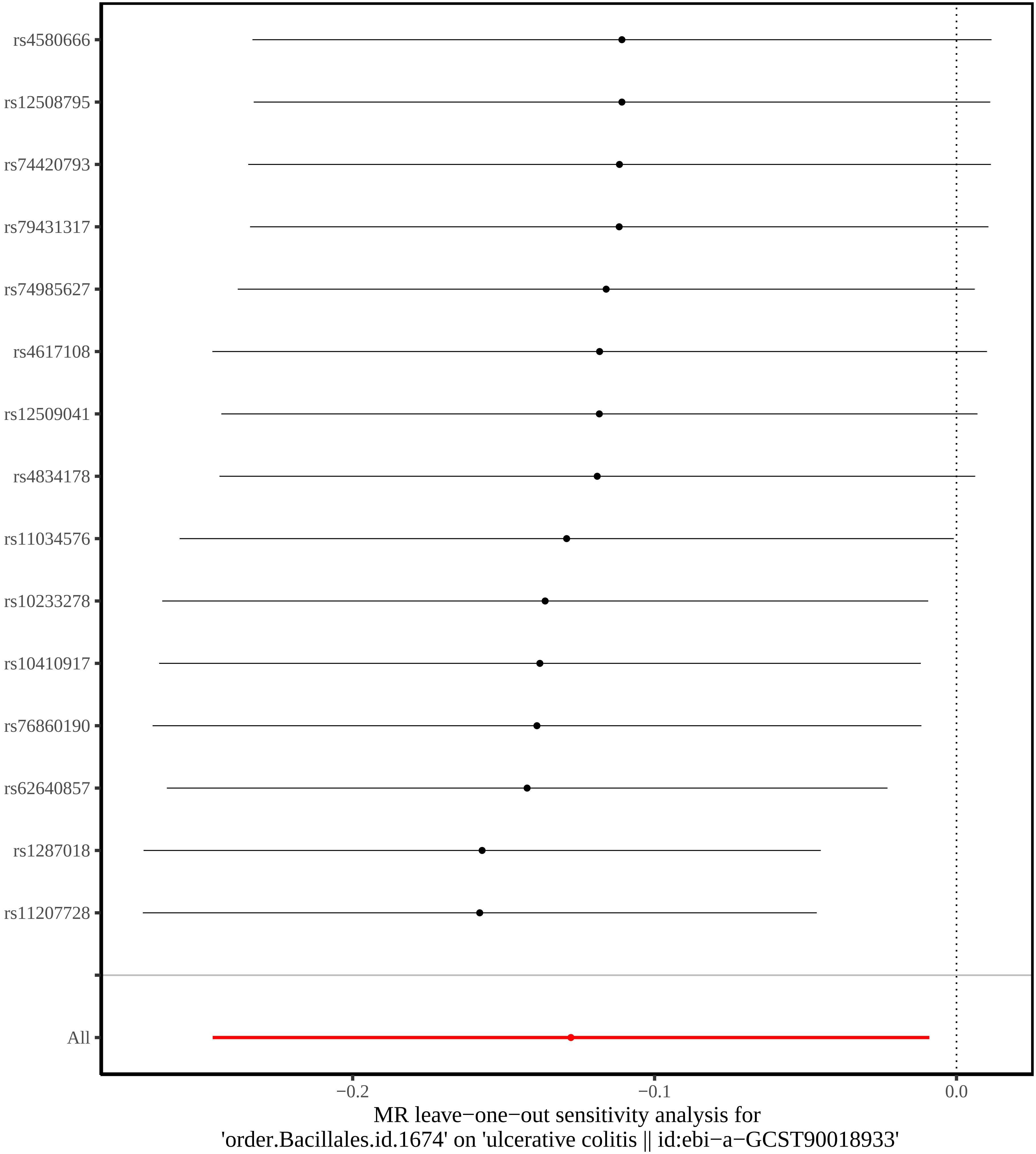
rs4580666
rs12508795
rs74420793
rs79431317
rs74985627
rs4617108
rs12509041
rs4834178
rs11034576
rs10233278
rs10410917
rs76860190
rs62640857
rs1287018
rs11207728
All
−0.2
−0.1
0.0
MR leave−one−out sensitivity analysis for
'order.Bacillales.id.1674' on 'ulcerative colitis || id:ebi−a−GCST90018933'

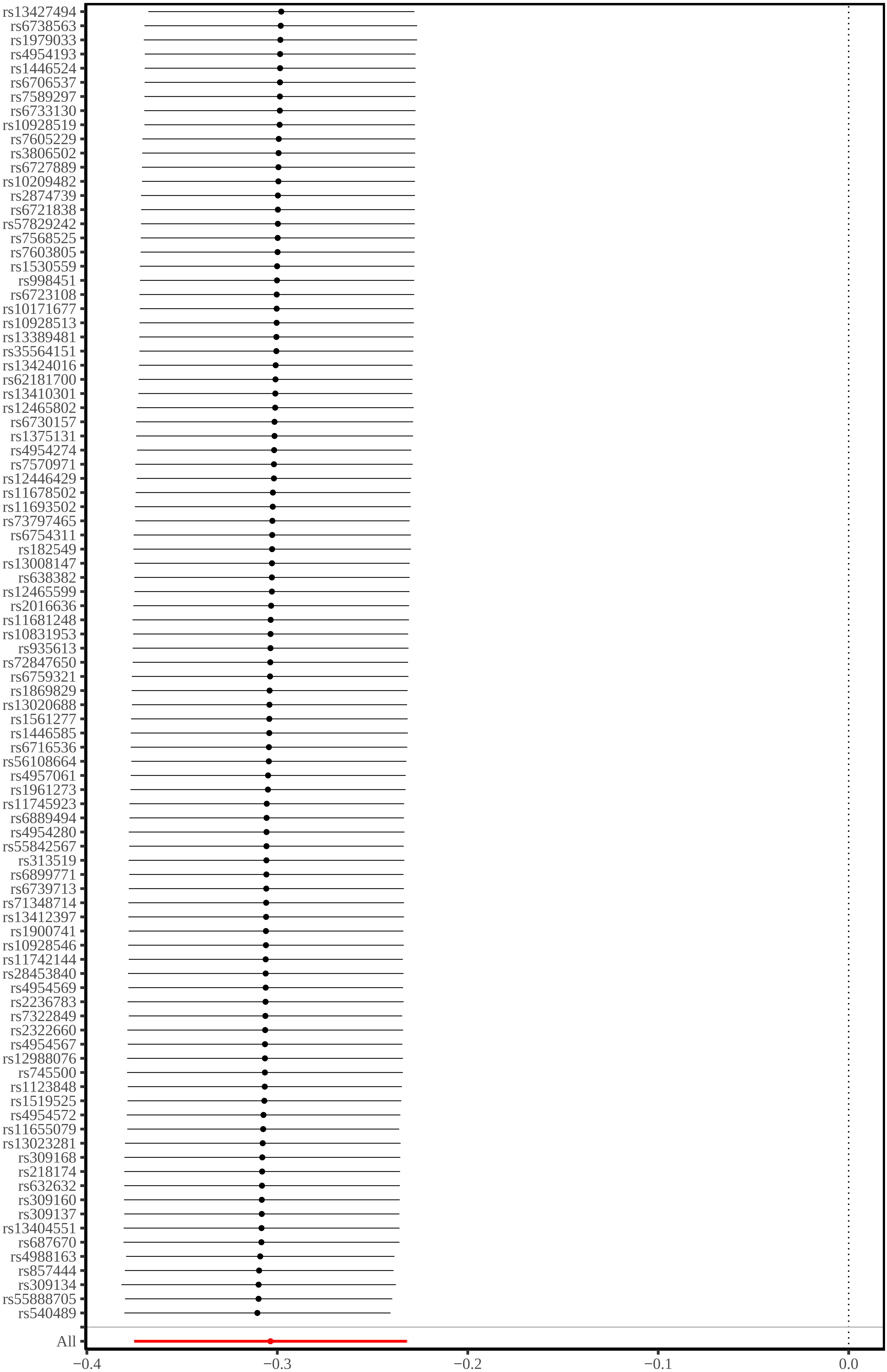

MR leave−one−out sensitivity analysis for
'order.Bifidobacteriales.id.432' on 'ulcerative colitis || id:ebi−a−GCST90018933'

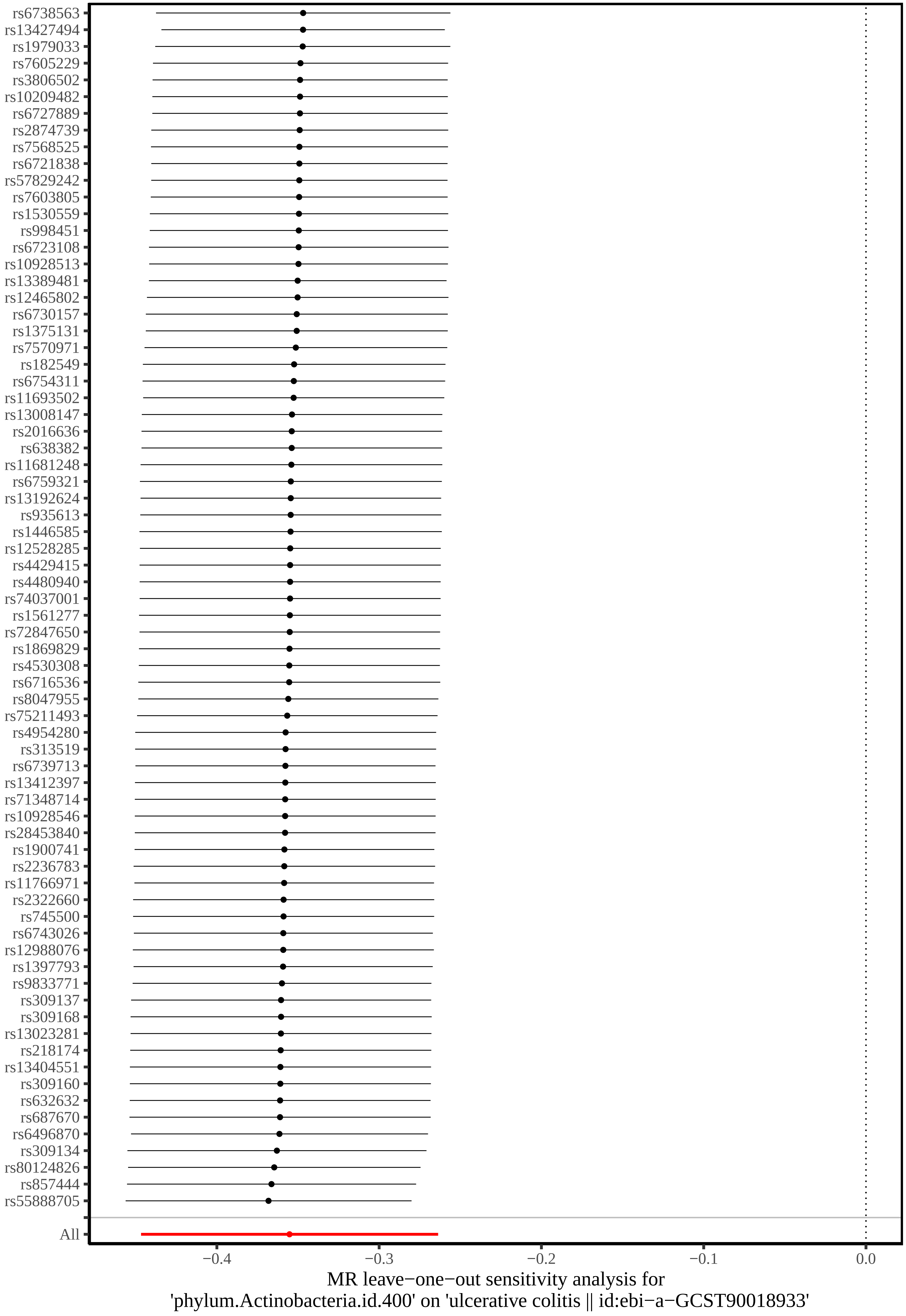
rs6738563
rs13427494
rs1979033
rs7605229
rs3806502
rs10209482
rs6727889
rs2874739
rs7568525
rs6721838
rs57829242
rs7603805
rs1530559
rs998451
rs6723108
rs10928513
rs13389481
rs12465802
rs6730157
rs1375131
rs7570971
rs182549
rs6754311
rs11693502
rs13008147
rs2016636
rs638382
rs11681248
rs6759321
rs13192624
rs935613
rs1446585
rs12528285
rs4429415
rs4480940
rs74037001
rs1561277
rs72847650
rs1869829
rs4530308
rs6716536
rs8047955
rs75211493
rs4954280
rs313519
rs6739713
rs13412397
rs71348714
rs10928546
rs28453840
rs1900741
rs2236783
rs11766971
rs2322660
rs745500
rs6743026
rs12988076
rs1397793
rs9833771
rs309137
rs309168
rs13023281
rs218174
rs13404551
rs309160
rs632632
rs687670
rs6496870
rs309134
rs80124826
rs857444
rs55888705
All
−0.4
−0.3
−0.2
−0.1
0.0
MR leave−one−out sensitivity analysis for
'phylum.Actinobacteria.id.400' on 'ulcerative colitis || id:ebi−a−GCST90018933'

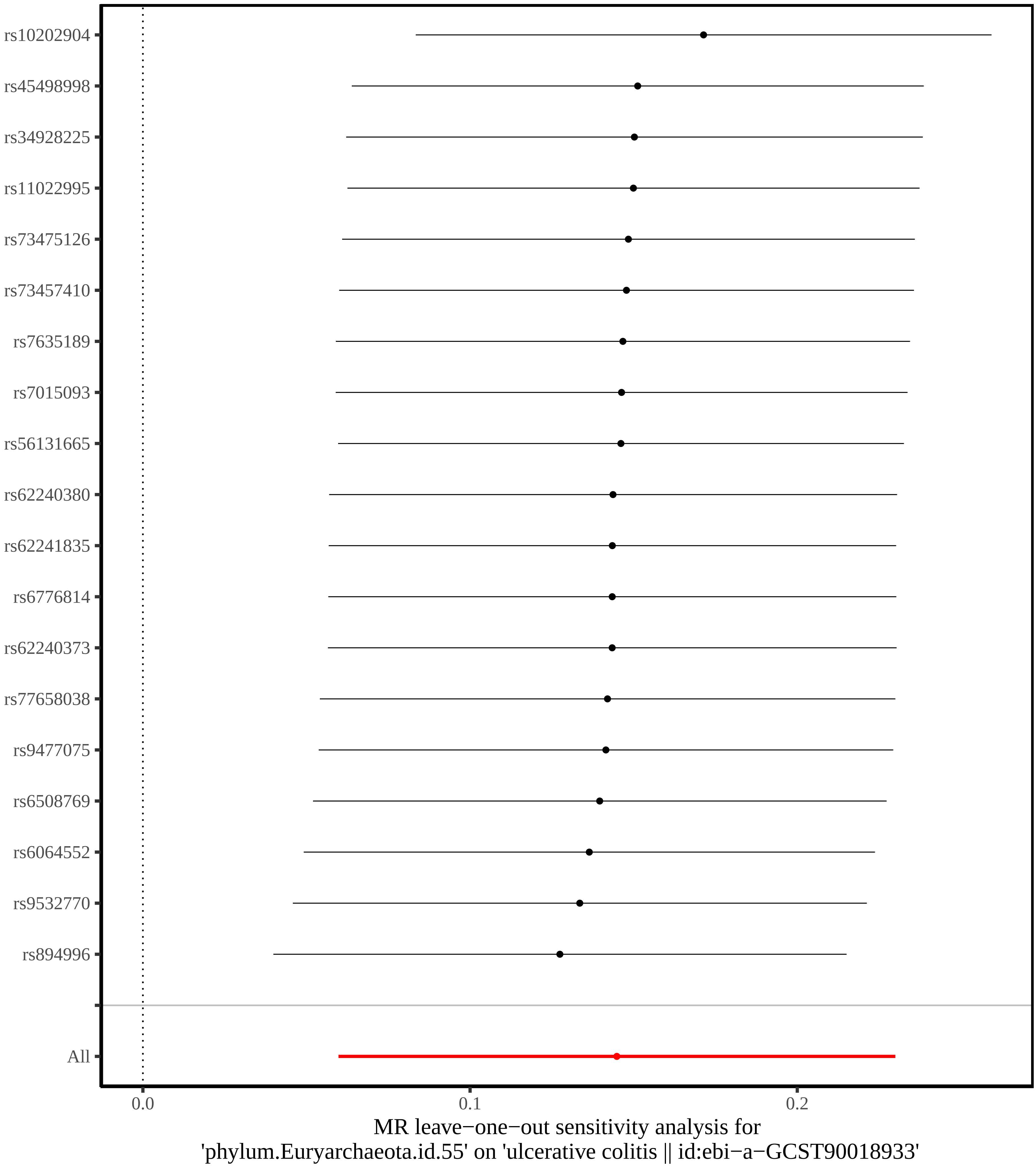

rs10202904
rs45498998
rs34928225
rs11022995
rs73475126
rs73457410
rs7635189
rs7015093
rs56131665
rs62240380
rs62241835
rs6776814
rs62240373
rs77658038
rs9477075
rs6508769
rs6064552
rs9532770
rs894996
All
0.0
0.1
0.2
MR leave−one−out sensitivity analysis for
'phylum.Euryarchaeota.id.55' on 'ulcerative colitis || id:ebi−a−GCST90018933'

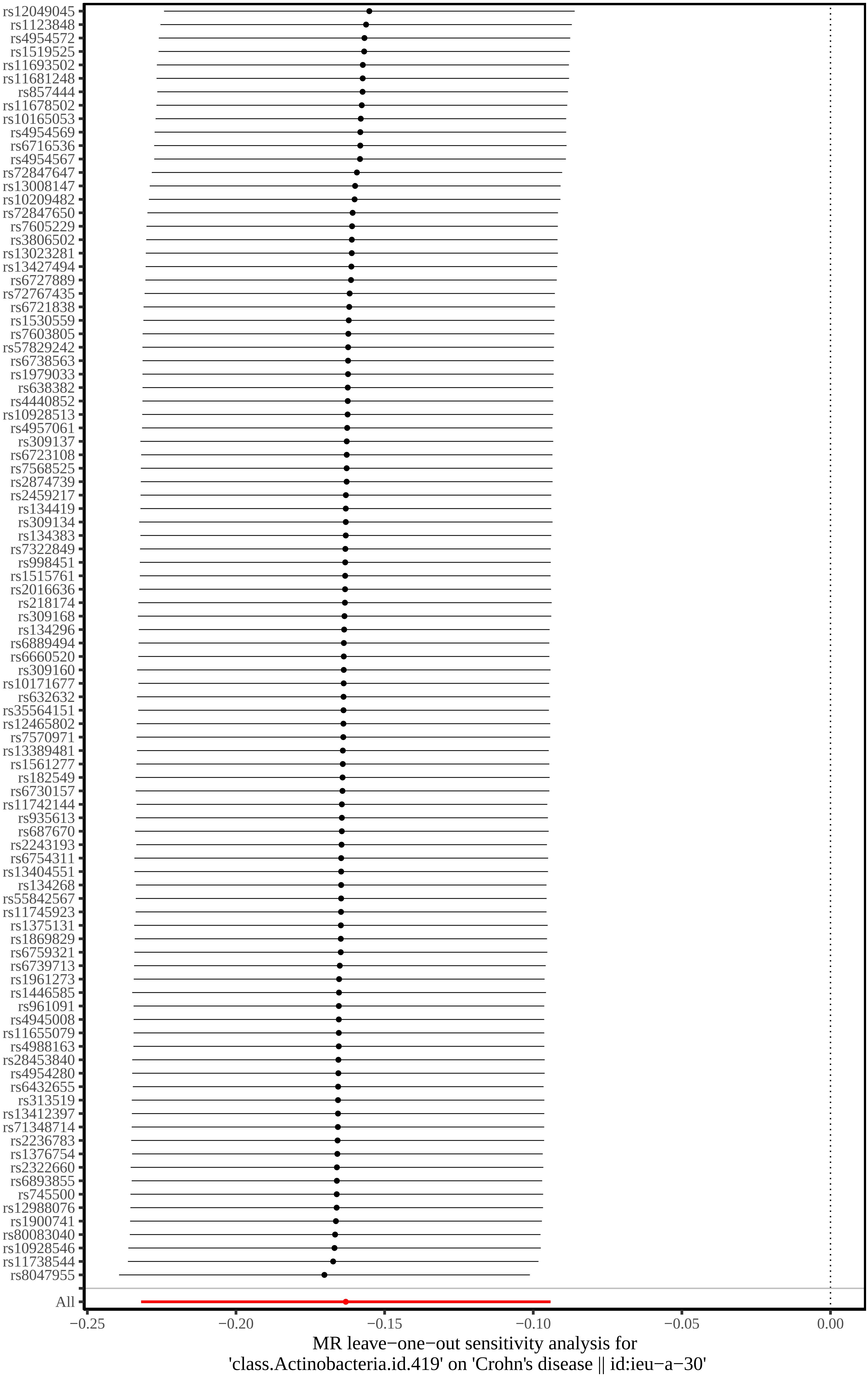
rs12049045
rs1123848
rs4954572
rs1519525
rs11693502
rs11681248
rs857444
rs11678502
rs10165053
rs4954569
rs6716536
rs4954567
rs72847647
rs13008147
rs10209482
rs72847650
rs7605229
rs3806502
rs13023281
rs13427494
rs6727889
rs72767435
rs6721838
rs1530559
rs7603805
rs57829242
rs6738563
rs1979033
rs638382
rs4440852
rs10928513
rs4957061
rs309137
rs6723108
rs7568525
rs2874739
rs2459217
rs134419
rs309134
rs134383
rs7322849
rs998451
rs1515761
rs2016636
rs218174
rs309168
rs134296
rs6889494
rs6660520
rs309160
rs10171677
rs632632
rs35564151
rs12465802
rs7570971
rs13389481
rs1561277
rs182549
rs6730157
rs11742144
rs935613
rs687670
rs2243193
rs6754311
rs13404551
rs134268
rs55842567
rs11745923
rs1375131
rs1869829
rs6759321
rs6739713
rs1961273
rs1446585
rs961091
rs4945008
rs11655079
rs4988163
rs28453840
rs4954280
rs6432655
rs313519
rs13412397
rs71348714
rs2236783
rs1376754
rs2322660
rs6893855
rs745500
rs12988076
rs1900741
rs80083040
rs10928546
rs11738544
rs8047955
All
−0.25
−0.20
−0.15
−0.10
−0.05
0.00
MR leave−one−out sensitivity analysis for
'class.Actinobacteria.id.419' on 'Crohn's disease || id:ieu−a−30'

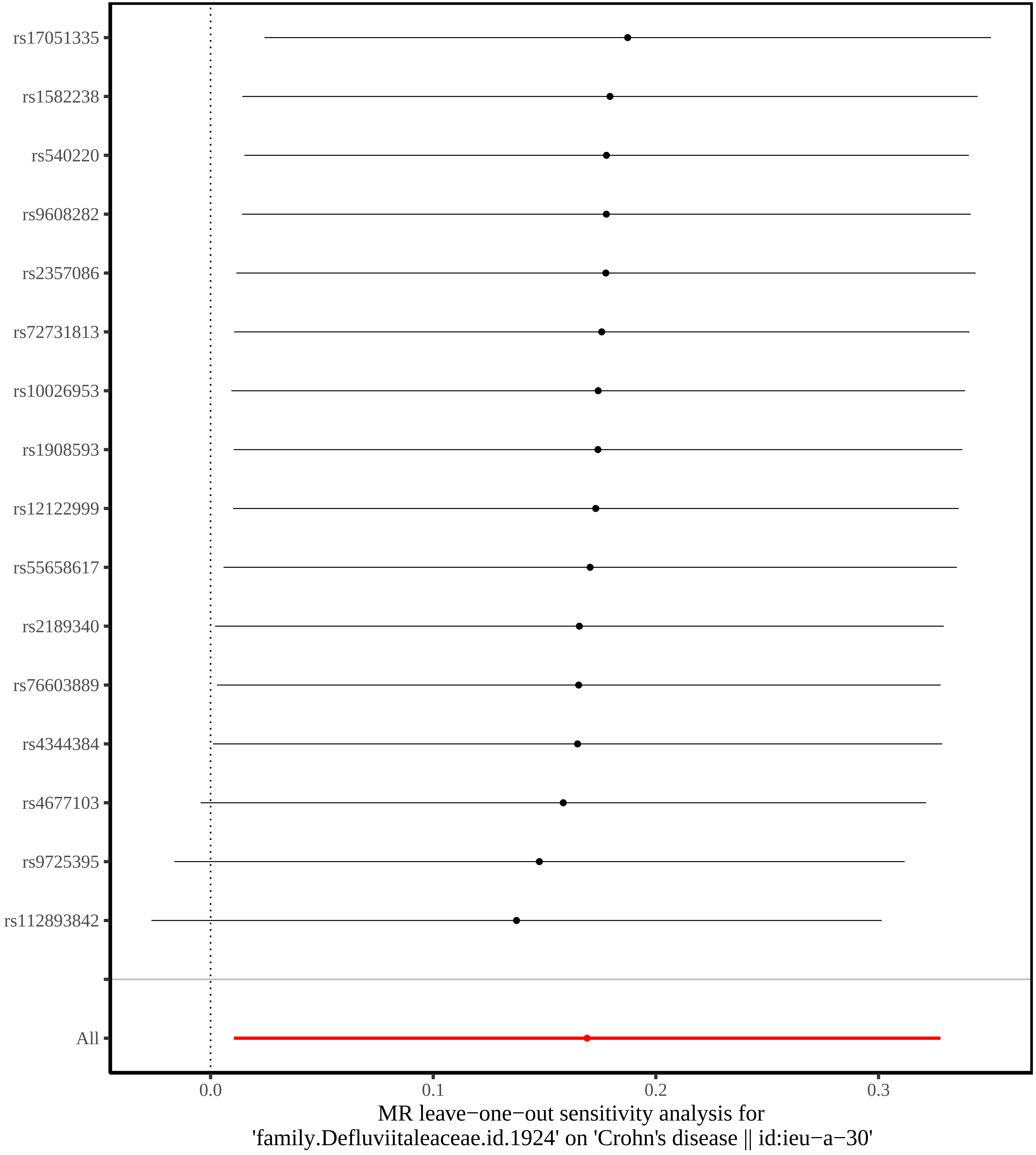

rs17051335
rs1582238
rs540220
rs9608282
rs2357086
rs72731813
rs10026953
rs1908593
rs12122999
rs55658617
rs2189340
rs76603889
rs4344384
rs4677103
rs9725395
rs112893842
All
0.0
0.1
0.2
0.3
MR leave−one−out sensitivity analysis for
'family.Defluviitaleaceae.id.1924' on 'Crohn's disease || id:ieu−a−30'

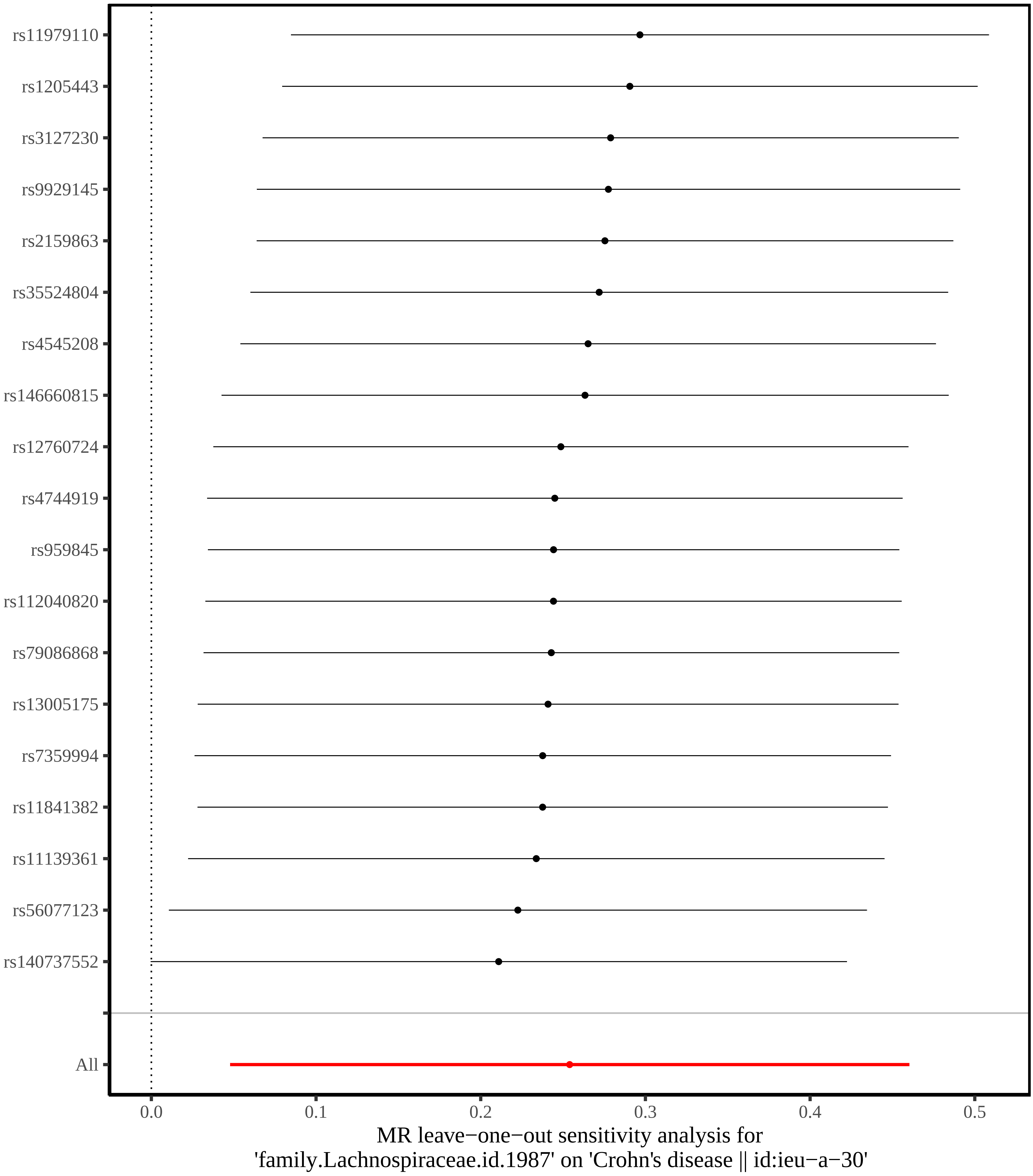
rs11979110
rs1205443
rs3127230
rs9929145
rs2159863
rs35524804
rs4545208
rs146660815
rs12760724
rs4744919
rs959845
rs112040820
rs79086868
rs13005175
rs7359994
rs11841382
rs11139361
rs56077123
rs140737552
All
0.0
0.1
0.2
0.3
0.4
0.5
MR leave−one−out sensitivity analysis for
'family.Lachnospiraceae.id.1987' on 'Crohn's disease || id:ieu−a−30'

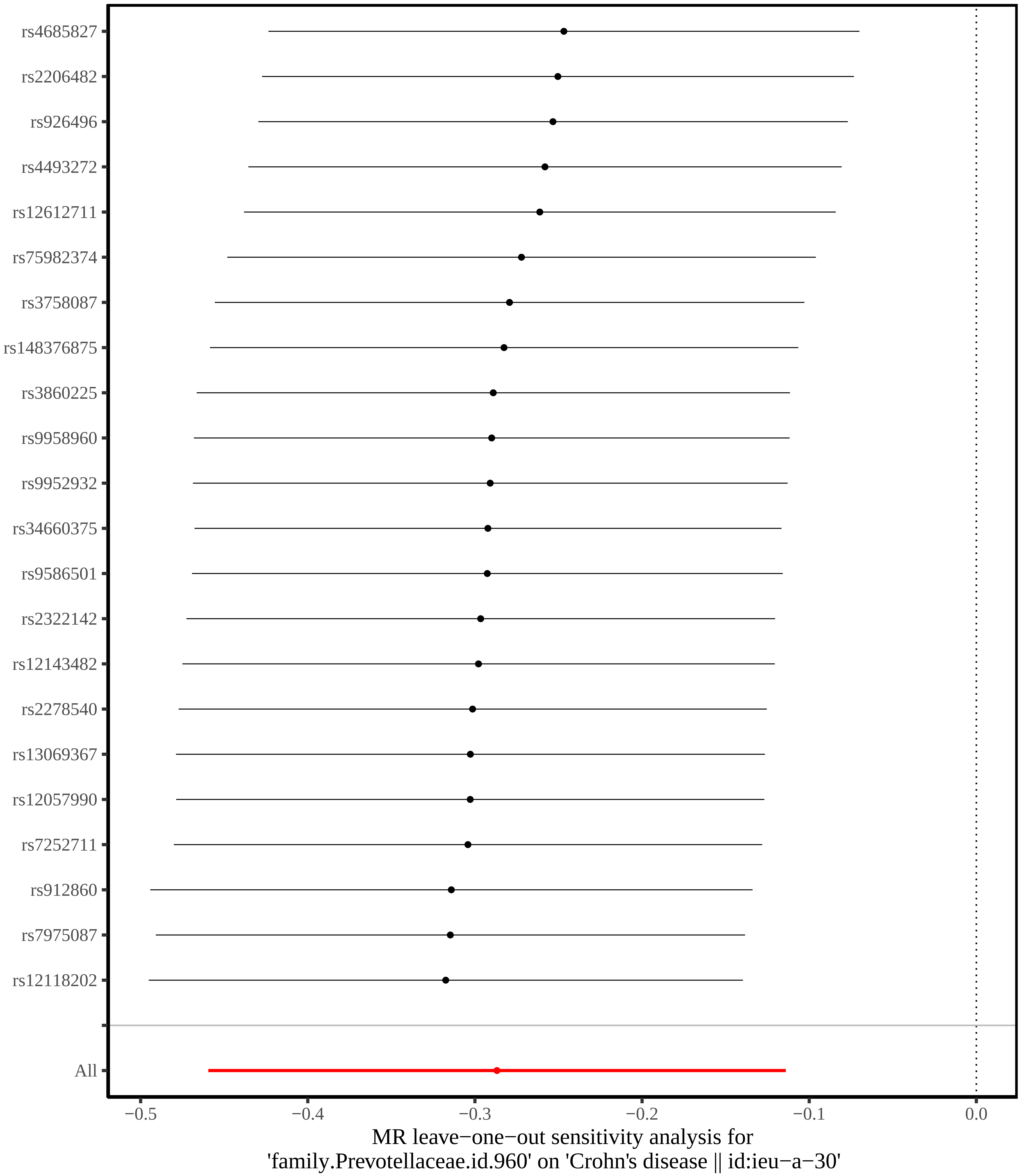
rs4685827
rs2206482
rs926496
rs4493272
rs12612711
rs75982374
rs3758087
rs148376875
rs3860225
rs9958960
rs9952932
rs34660375
rs9586501
rs2322142
rs12143482
rs2278540
rs13069367
rs12057990
rs7252711
rs912860
rs7975087
rs12118202
All
−0.5
−0.4
−0.3
−0.2
−0.1
0.0
MR leave−one−out sensitivity analysis for
'family.Prevotellaceae.id.960' on 'Crohn's disease || id:ieu−a−30'

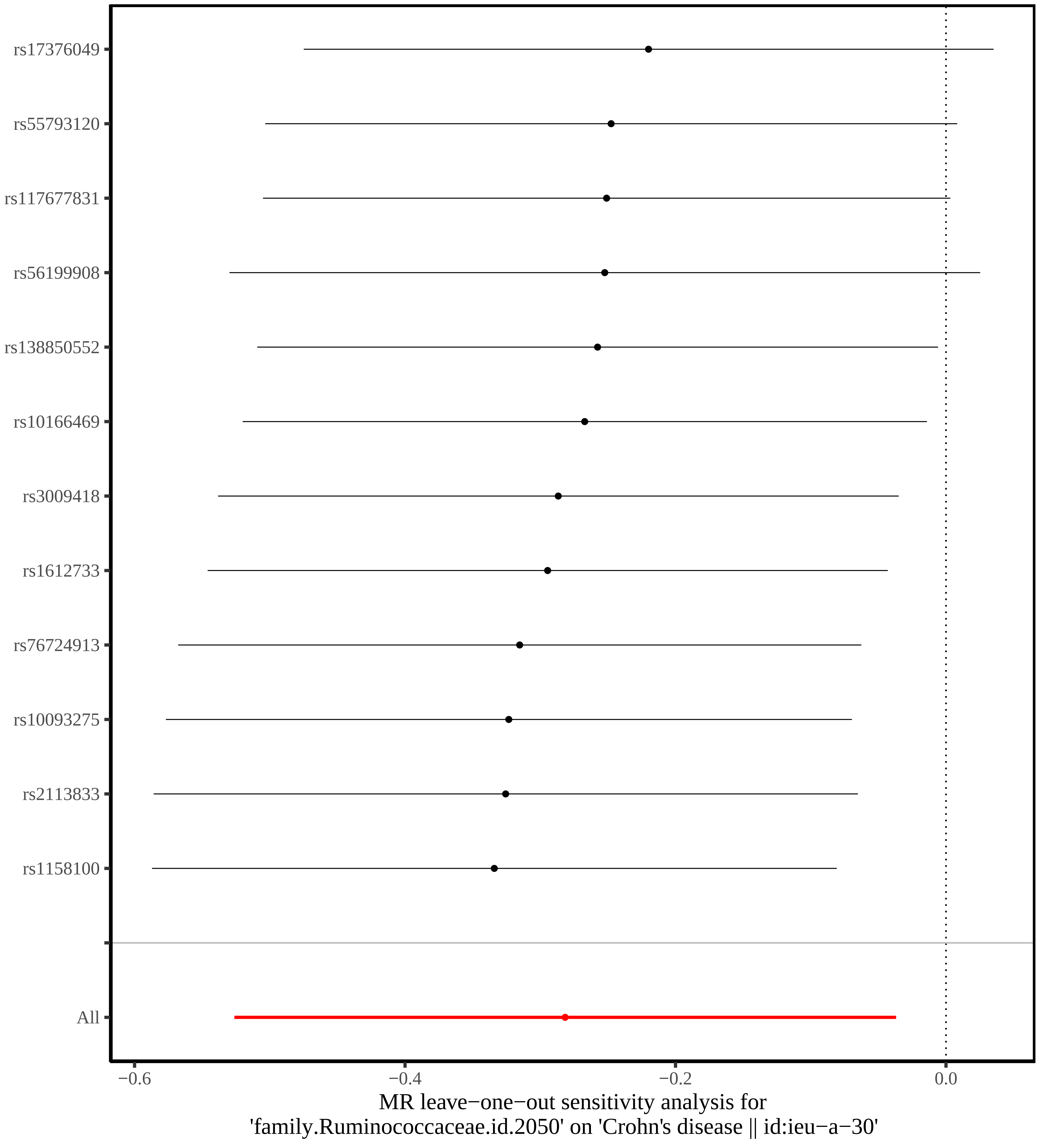
rs17376049
rs55793120
rs117677831
rs56199908
rs138850552
rs10166469
rs3009418
rs1612733
rs76724913
rs10093275
rs2113833
rs1158100
All
−0.6
−0.4
−0.2
0.0
MR leave−one−out sensitivity analysis for
'family.Ruminococcaceae.id.2050' on 'Crohn's disease || id:ieu−a−30'

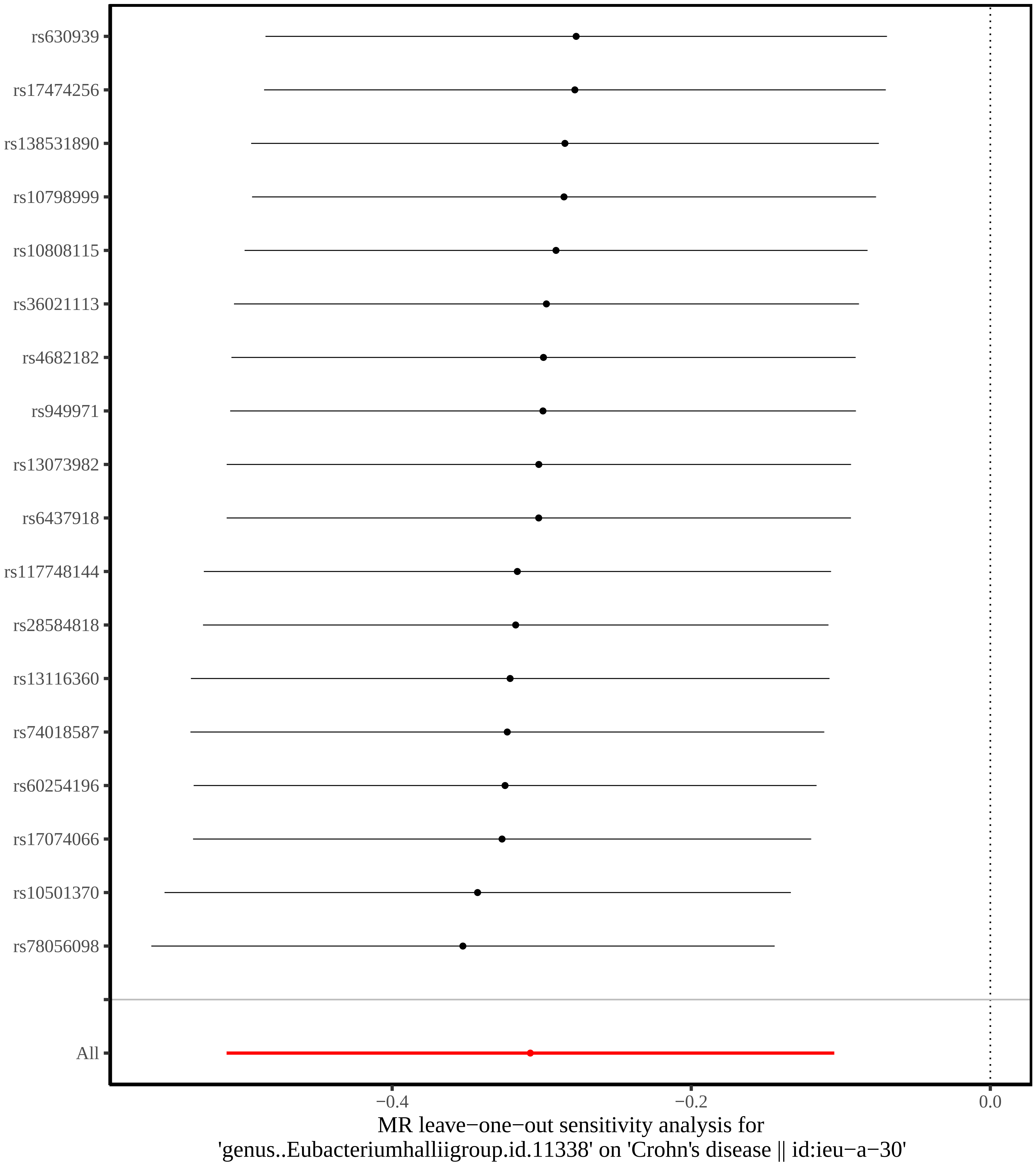
rs630939
rs17474256
rs138531890
rs10798999
rs10808115
rs36021113
rs4682182
rs949971
rs13073982
rs6437918
rs117748144
rs28584818
rs13116360
rs74018587
rs60254196
rs17074066
rs10501370
rs78056098
All
−0.4
−0.2
0.0
MR leave−one−out sensitivity analysis for
'genus..Eubacteriumhalliigroup.id.11338' on 'Crohn's disease || id:ieu−a−30'

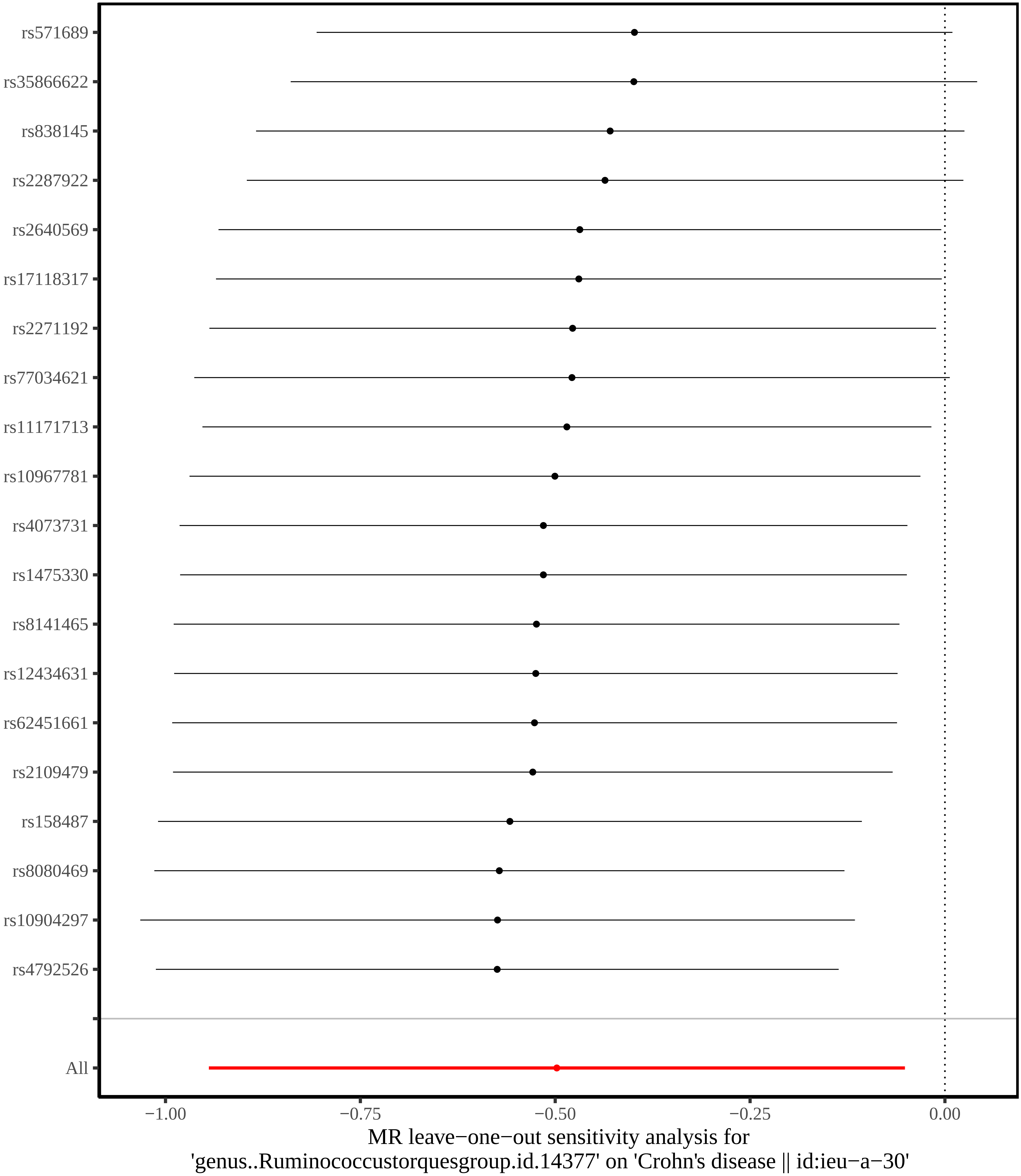
rs571689
rs35866622
rs838145
rs2287922
rs2640569
rs17118317
rs2271192
rs77034621
rs11171713
rs10967781
rs4073731
rs1475330
rs8141465
rs12434631
rs62451661
rs2109479
rs158487
rs8080469
rs10904297
rs4792526
All
−1.00
−0.75
−0.50
−0.25
0.00
MR leave−one−out sensitivity analysis for
'genus..Ruminococcustorquesgroup.id.14377' on 'Crohn's disease || id:ieu−a−30'

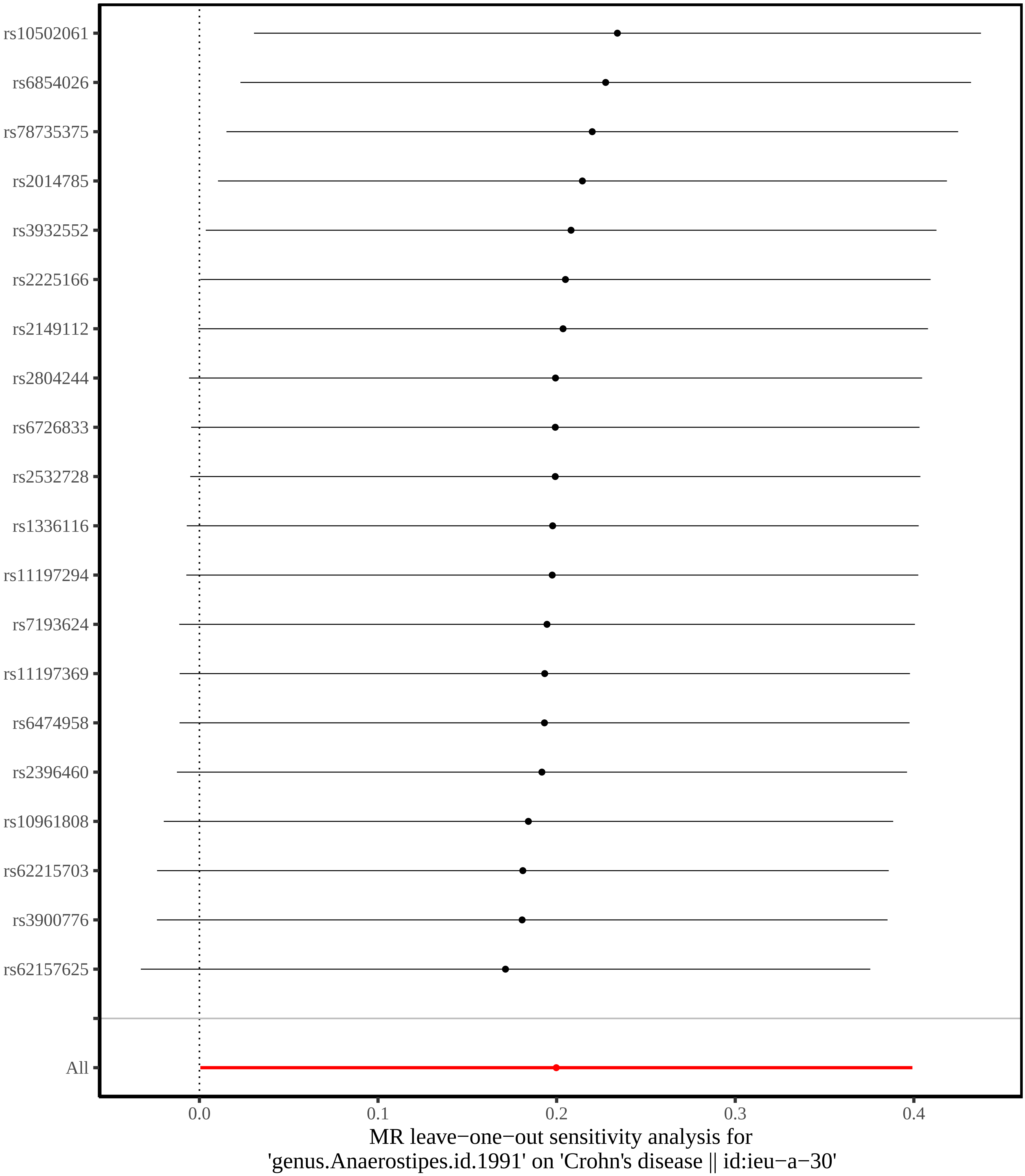
rs10502061
rs6854026
rs78735375
rs2014785
rs3932552
rs2225166
rs2149112
rs2804244
rs6726833
rs2532728
rs1336116
rs11197294
rs7193624
rs11197369
rs6474958
rs2396460
rs10961808
rs62215703
rs3900776
rs62157625
All
0.0
0.1
0.2
0.3
0.4
MR leave−one−out sensitivity analysis for
'genus.Anaerostipes.id.1991' on 'Crohn's disease || id:ieu−a−30'

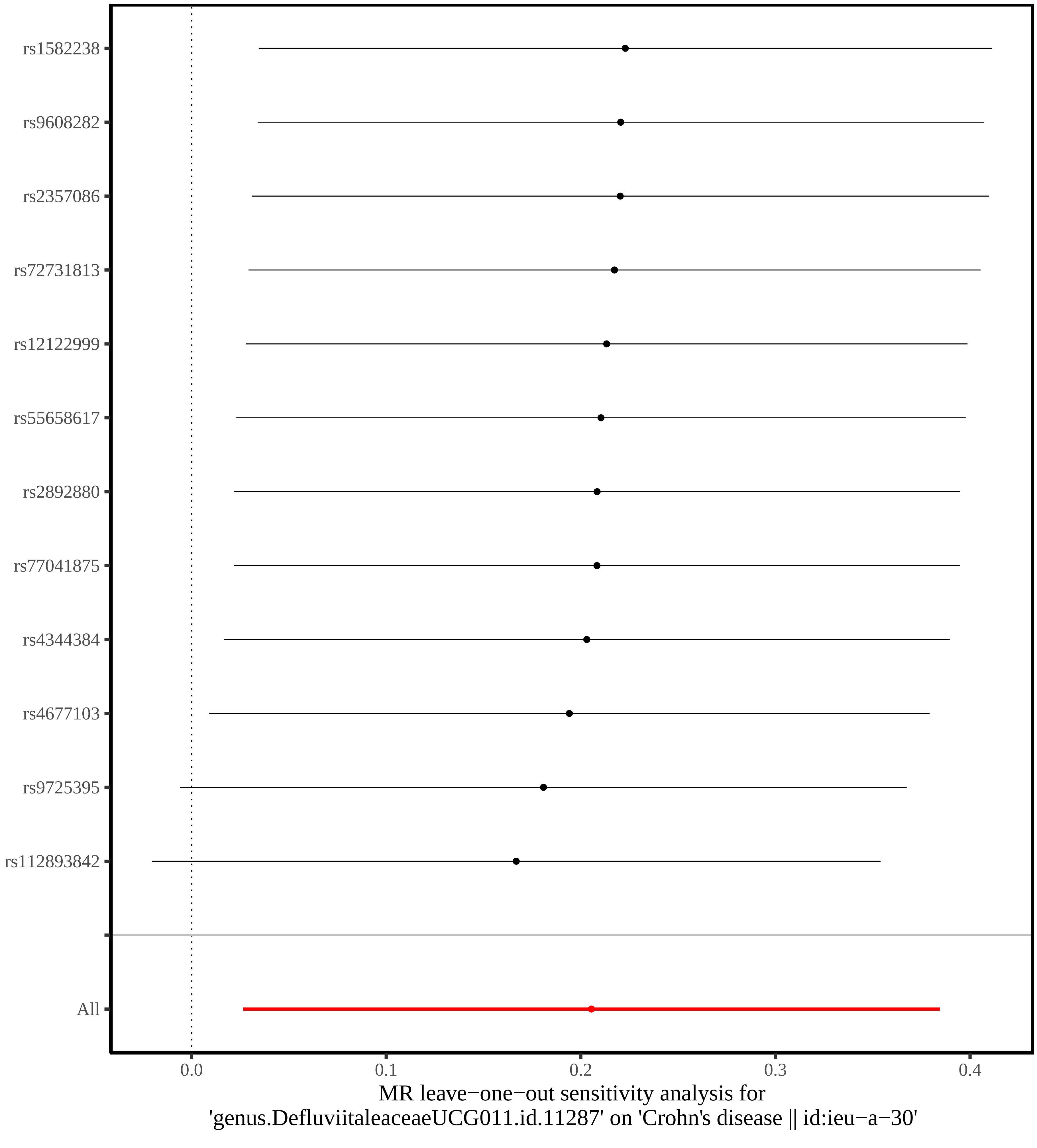

rs1582238
rs9608282
rs2357086
rs72731813
rs12122999
rs55658617
rs2892880
rs77041875
rs4344384
rs4677103
rs9725395
rs112893842
All
0.0
0.1
0.2
0.3
0.4
MR leave−one−out sensitivity analysis for
'genus.DefluviitaleaceaeUCG011.id.11287' on 'Crohn's disease || id:ieu−a−30'

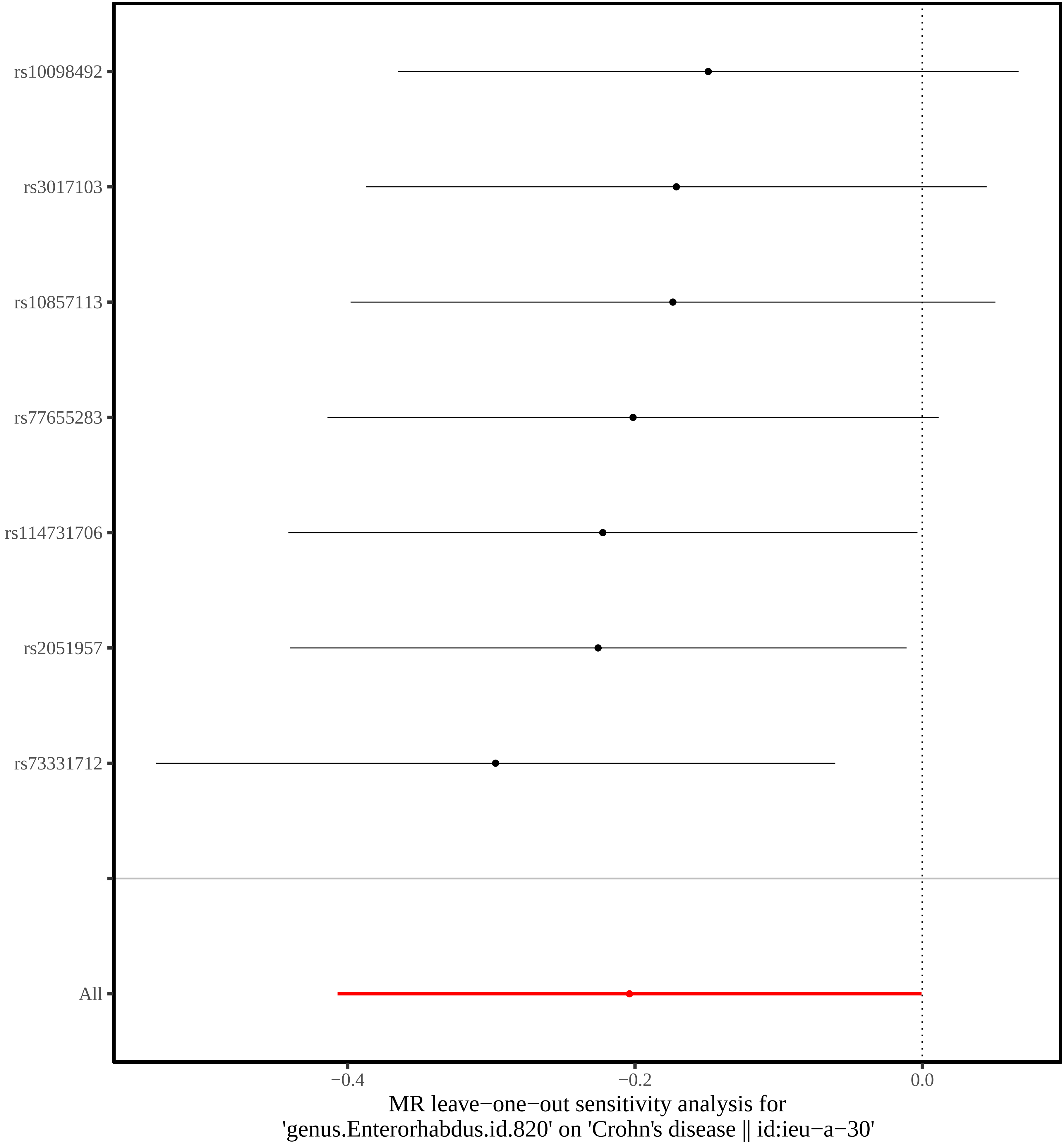
rs10098492
rs3017103
rs10857113
rs77655283
rs114731706
rs2051957
rs73331712
All
−0.4
−0.2
0.0
MR leave−one−out sensitivity analysis for
'genus.Enterorhabdus.id.820' on 'Crohn's disease || id:ieu−a−30'

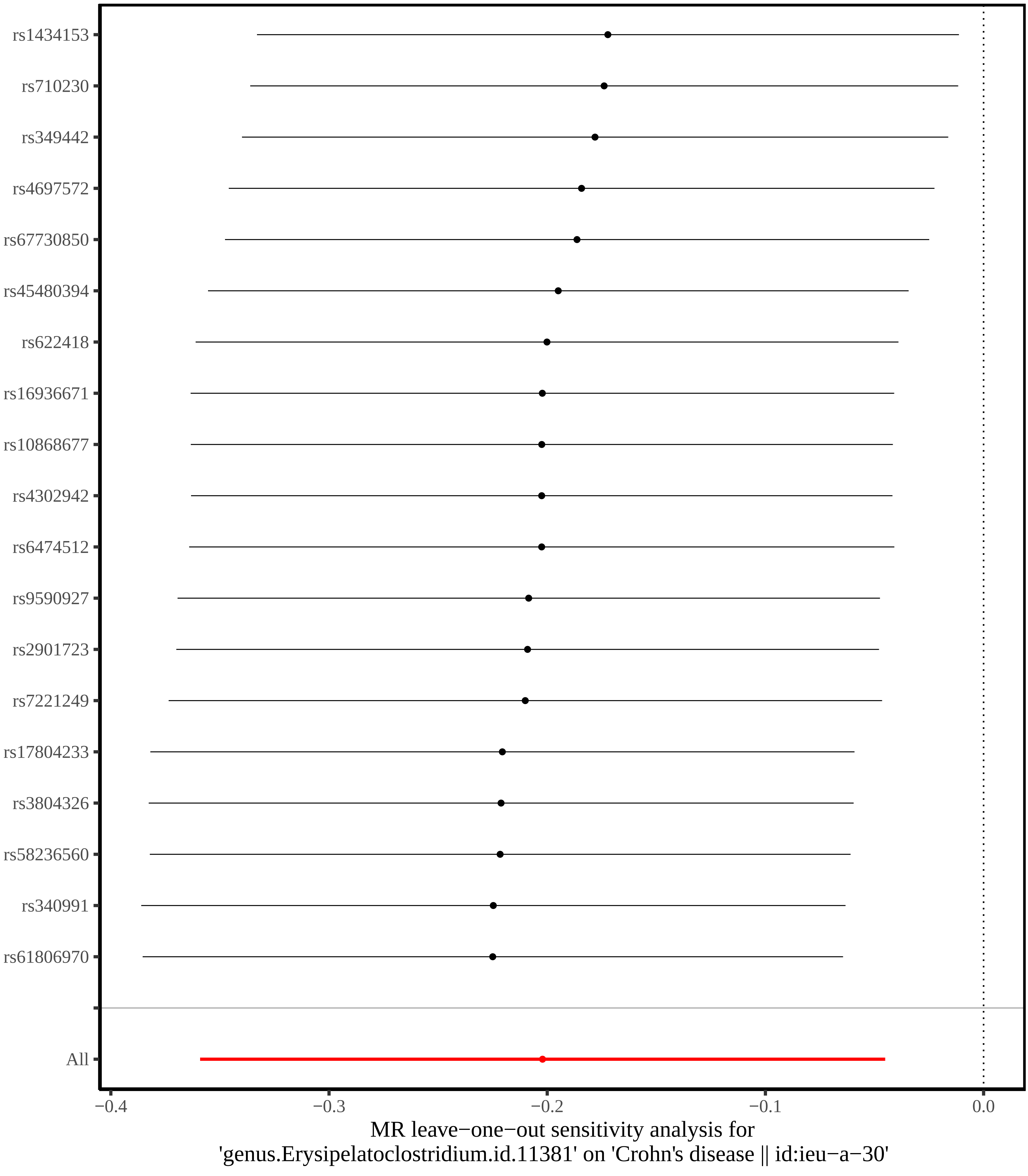
rs1434153
rs710230
rs349442
rs4697572
rs67730850
rs45480394
rs622418
rs16936671
rs10868677
rs4302942
rs6474512
rs9590927
rs2901723
rs7221249
rs17804233
rs3804326
rs58236560
rs340991
rs61806970
All
−0.4
−0.3
−0.2
−0.1
0.0
MR leave−one−out sensitivity analysis for
'genus.Erysipelatoclostridium.id.11381' on 'Crohn's disease || id:ieu−a−30'

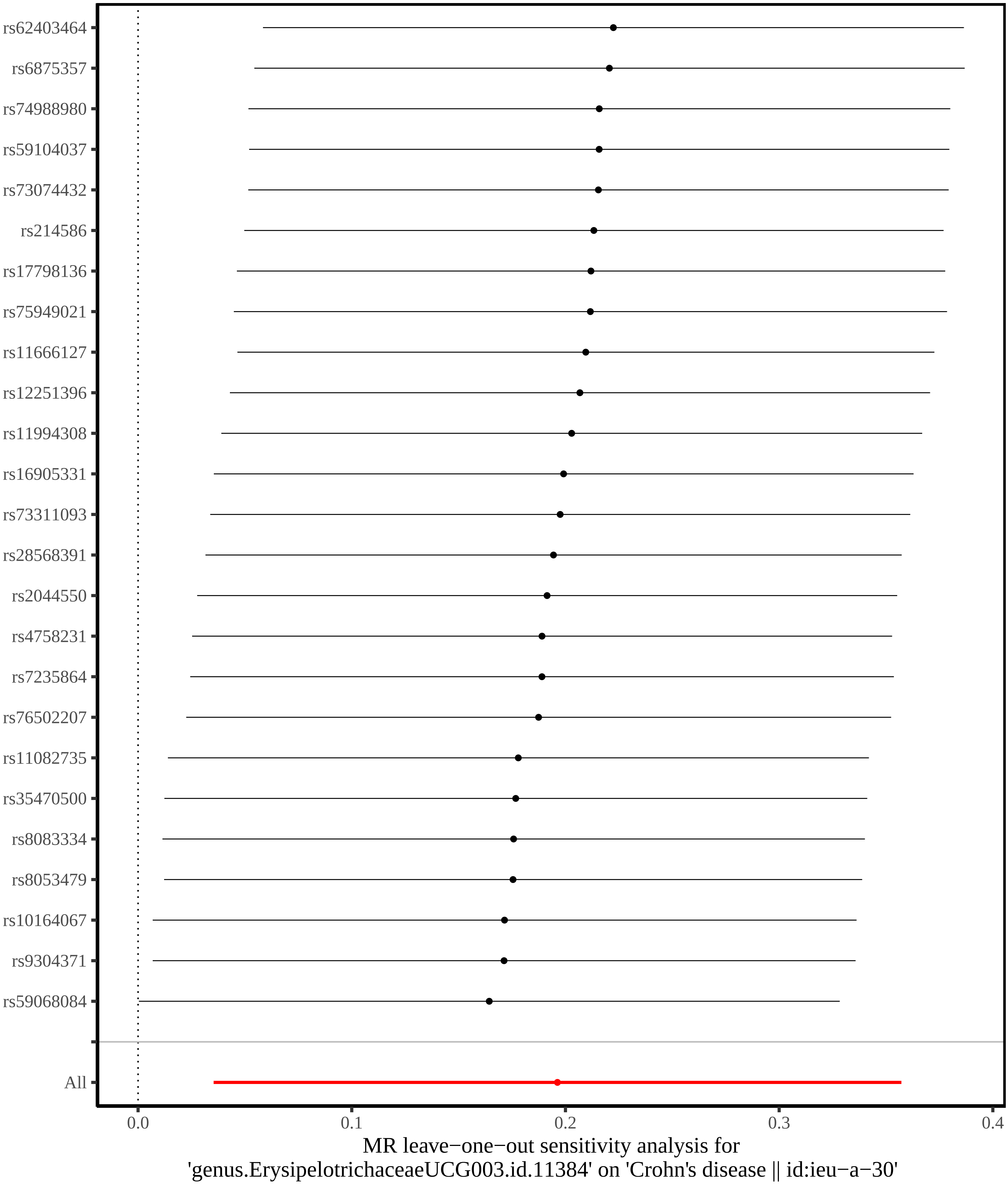
rs62403464
rs6875357
rs74988980
rs59104037
rs73074432
rs214586
rs17798136
rs75949021
rs11666127
rs12251396
rs11994308
rs16905331
rs73311093
rs28568391
rs2044550
rs4758231
rs7235864
rs76502207
rs11082735
rs35470500
rs8083334
rs8053479
rs10164067
rs9304371
rs59068084
All
0.0
0.1
0.2
0.3
0.4
MR leave−one−out sensitivity analysis for
'genus.ErysipelotrichaceaeUCG003.id.11384' on 'Crohn's disease || id:ieu−a−30'

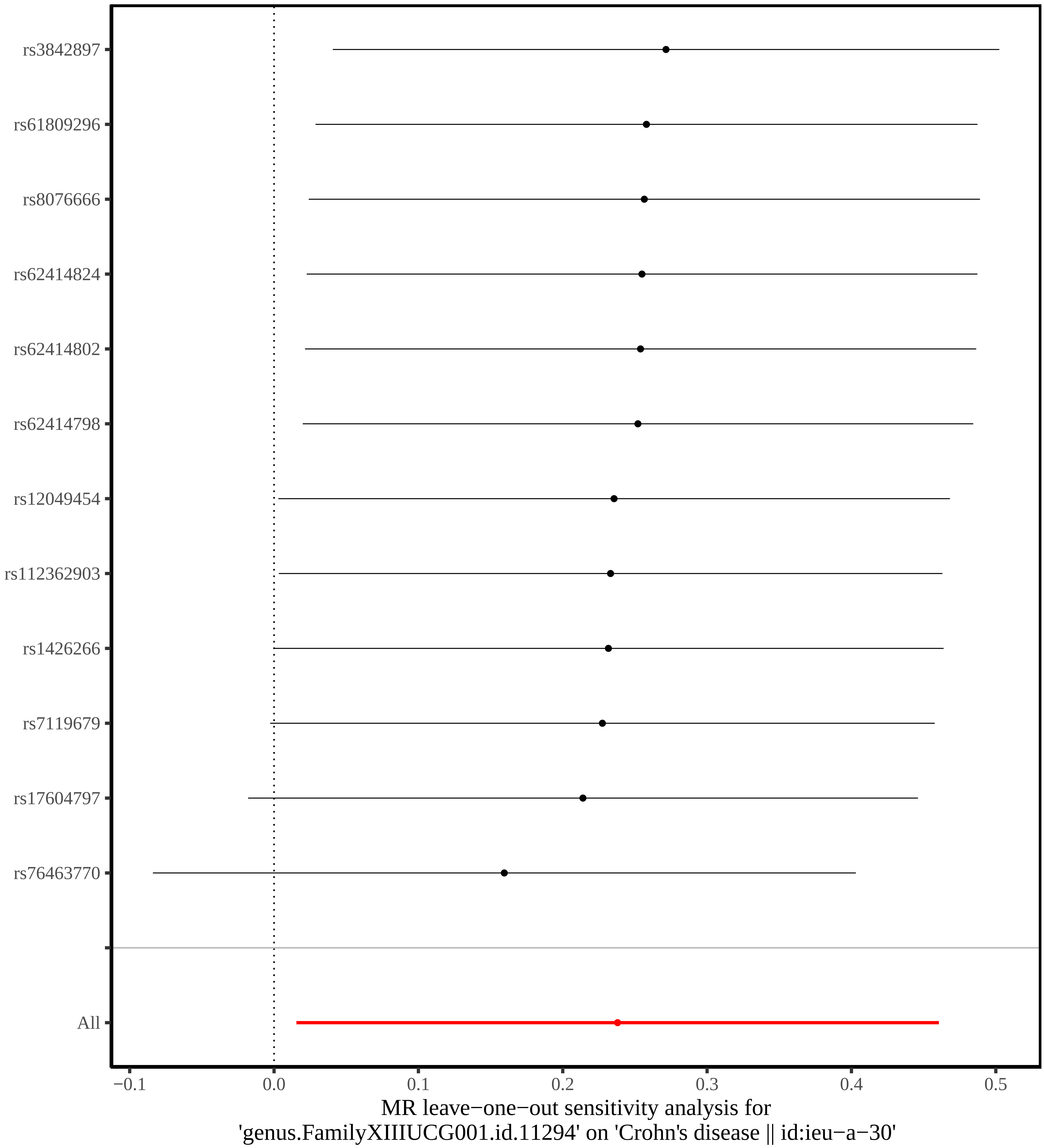
rs3842897
rs61809296
rs8076666
rs62414824
rs62414802
rs62414798
rs12049454
rs112362903
rs1426266
rs7119679
rs17604797
rs76463770
All
−0.1
0.0
0.1
0.2
0.3
0.4
0.5
MR leave−one−out sensitivity analysis for
'genus.FamilyXIIIUCG001.id.11294' on 'Crohn's disease || id:ieu−a−30'

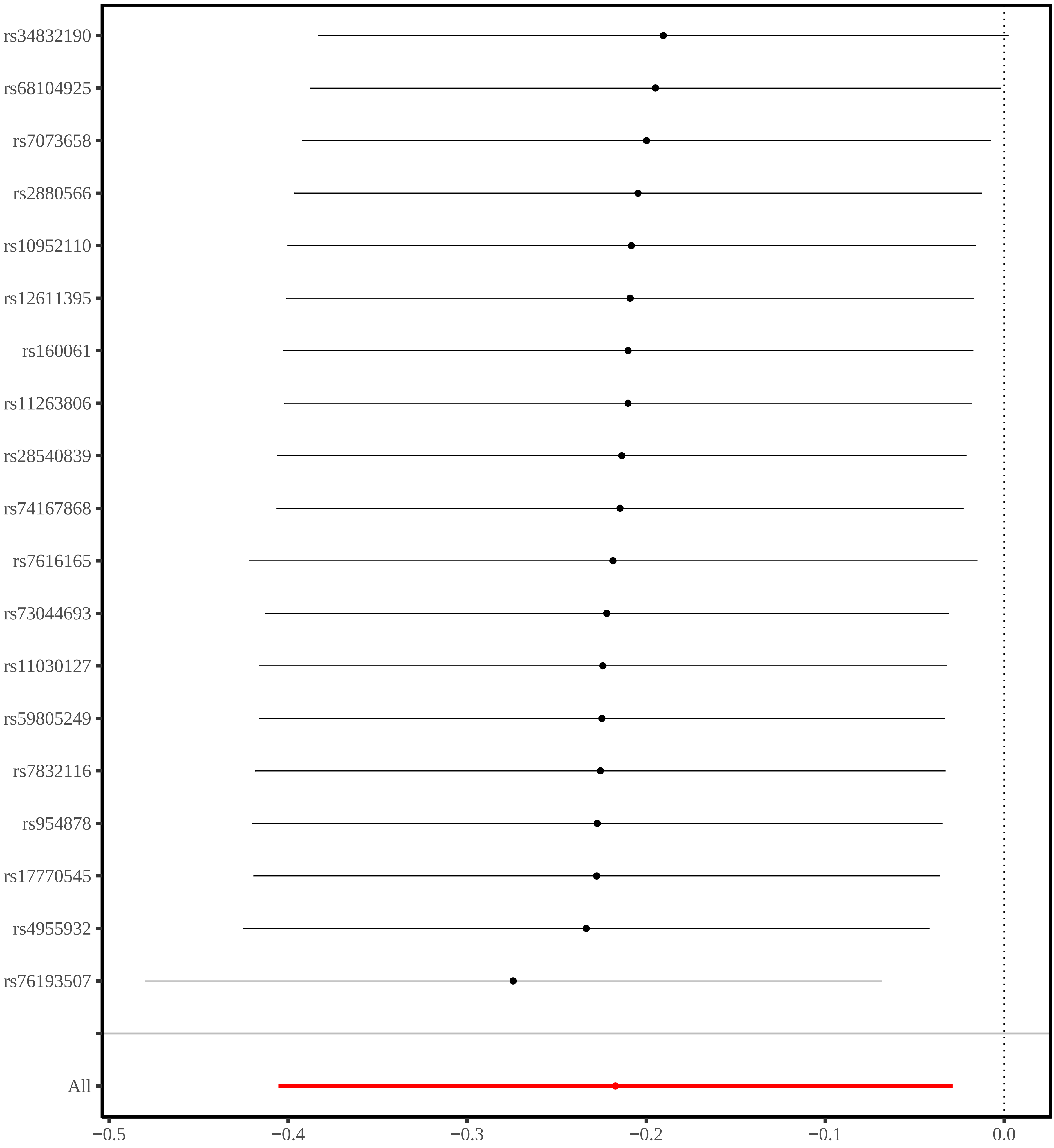

MR leave−one−out sensitivity analysis for
'genus.LachnospiraceaeNK4A136group.id.11319' on 'Crohn's disease || id:ieu−a−30'

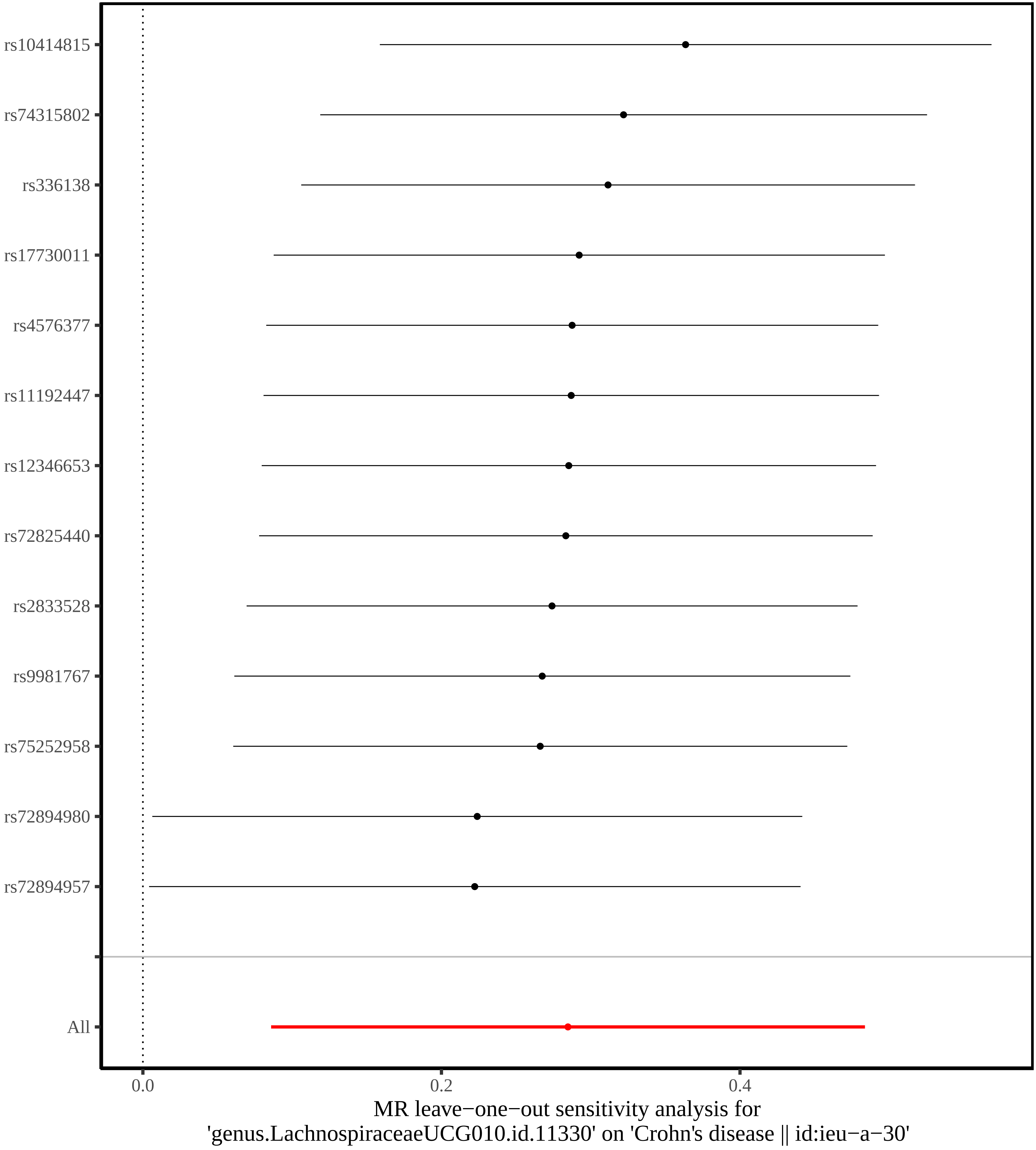
rs10414815
rs74315802
rs336138
rs17730011
rs4576377
rs11192447
rs12346653
rs72825440
rs2833528
rs9981767
rs75252958
rs72894980
rs72894957
All
0.0
0.2
0.4
MR leave−one−out sensitivity analysis for
'genus.LachnospiraceaeUCG010.id.11330' on 'Crohn's disease || id:ieu−a−30'

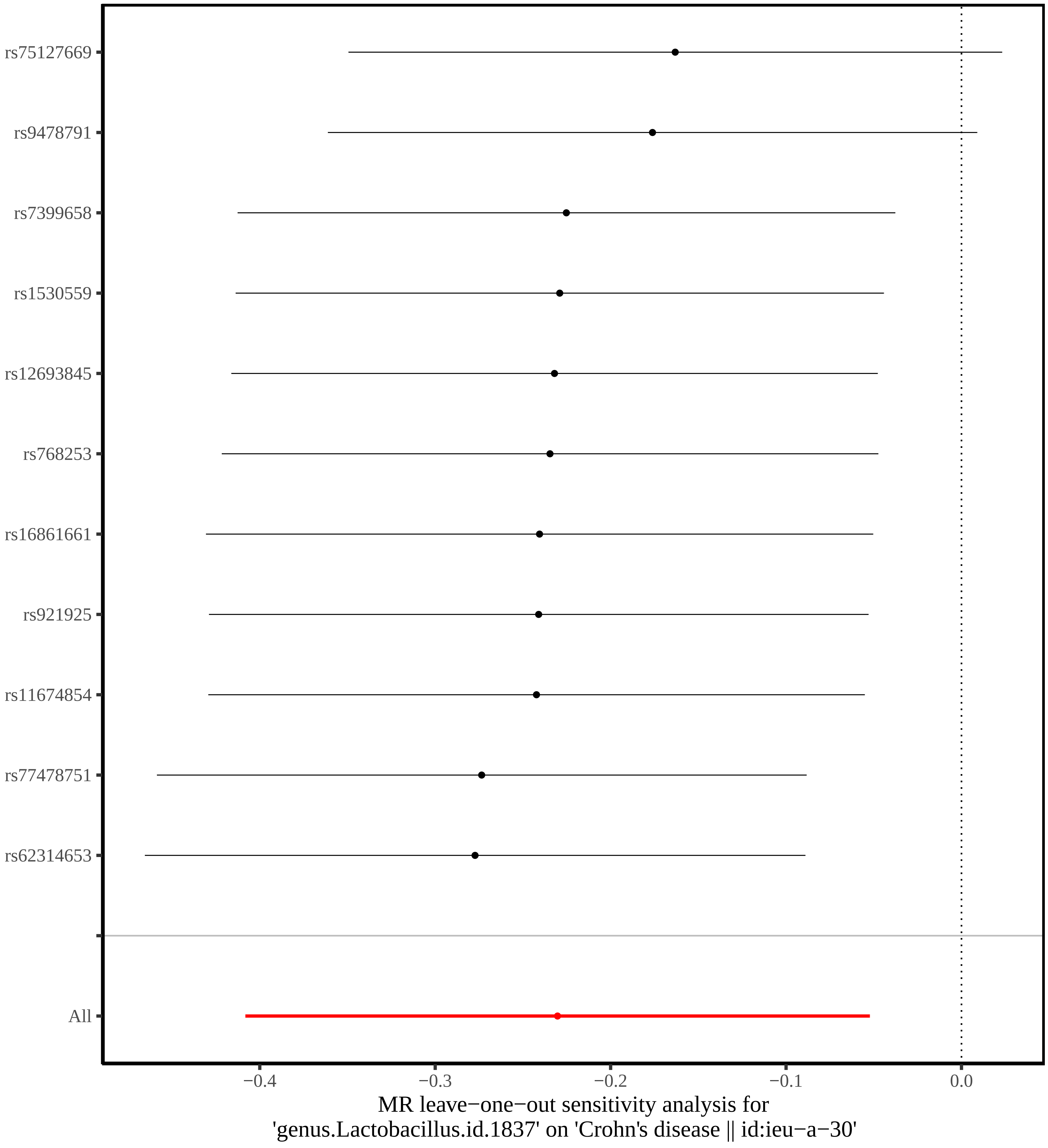
rs75127669
rs9478791
rs7399658
rs1530559
rs12693845
rs768253
rs16861661
rs921925
rs11674854
rs77478751
rs62314653
All
−0.4
−0.3
−0.2
−0.1
0.0
MR leave−one−out sensitivity analysis for
'genus.Lactobacillus.id.1837' on 'Crohn's disease || id:ieu−a−30'

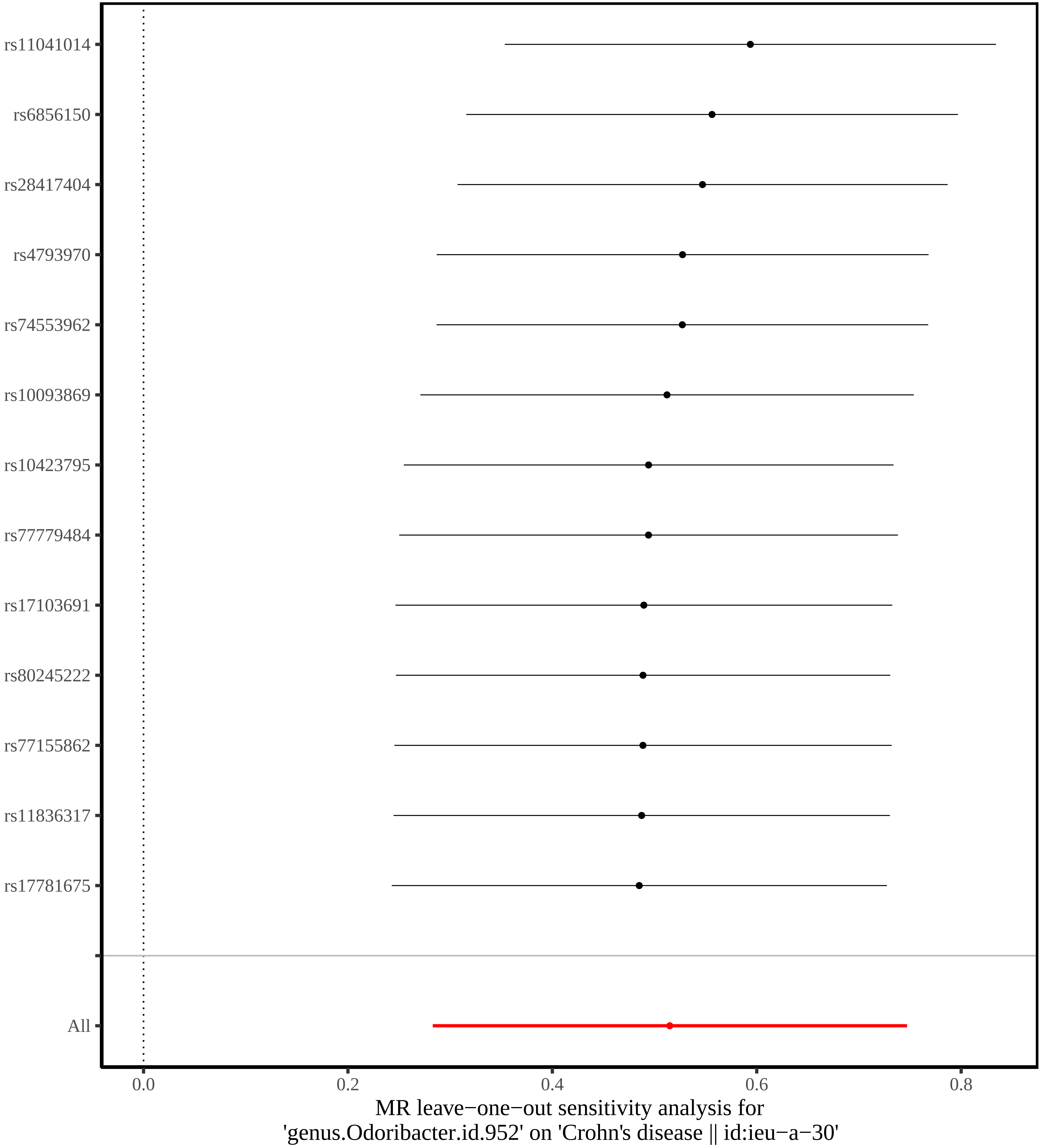

rs11041014
rs6856150
rs28417404
rs4793970
rs74553962
rs10093869
rs10423795
rs77779484
rs17103691
rs80245222
rs77155862
rs11836317
rs17781675
All
0.0
0.2
0.4
0.6
0.8
MR leave−one−out sensitivity analysis for
'genus.Odoribacter.id.952' on 'Crohn's disease || id:ieu−a−30'

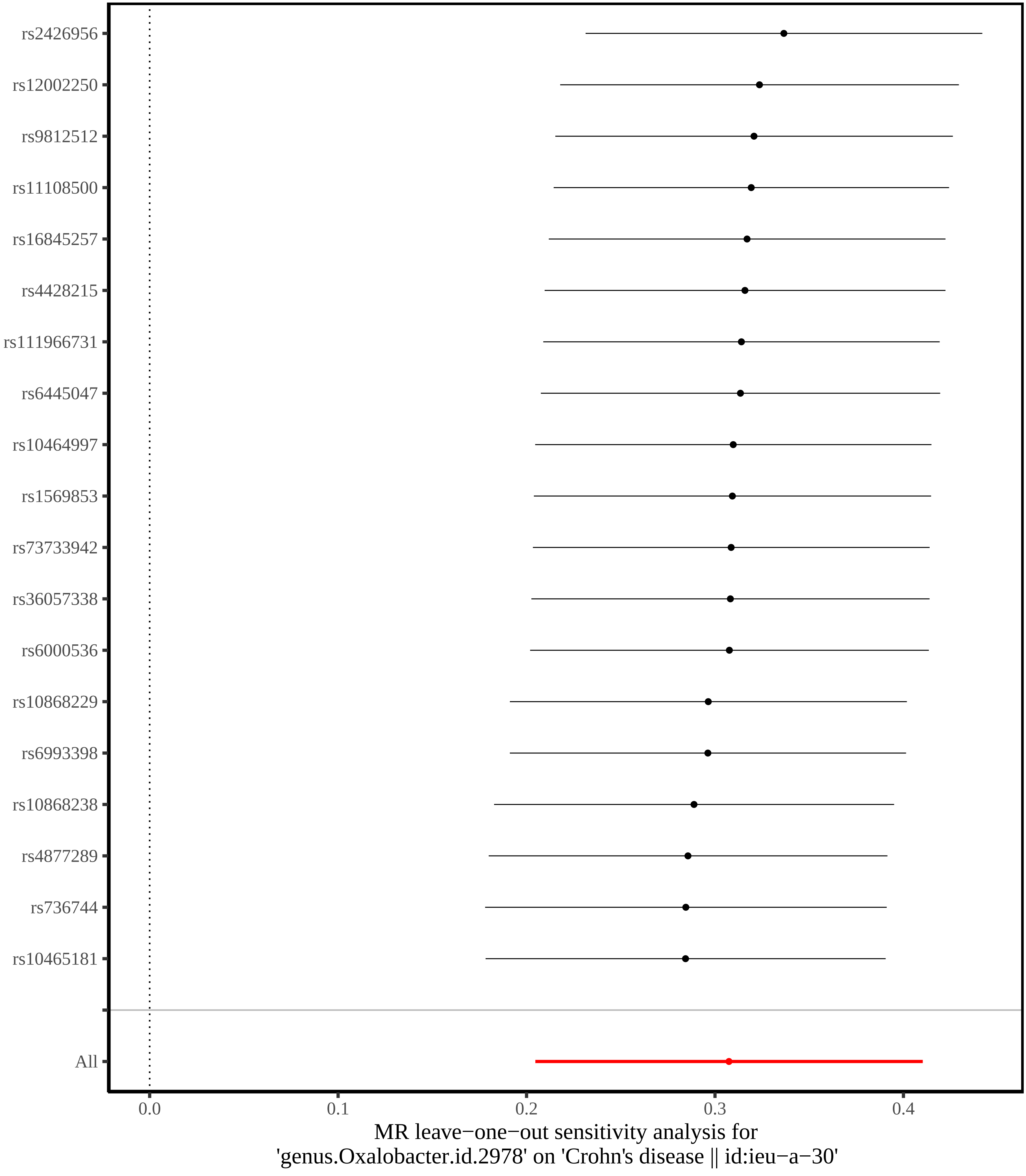
rs2426956
rs12002250
rs9812512
rs11108500
rs16845257
rs4428215
rs111966731
rs6445047
rs10464997
rs1569853
rs73733942
rs36057338
rs6000536
rs10868229
rs6993398
rs10868238
rs4877289
rs736744
rs10465181
All
0.0
0.1
0.2
0.3
0.4
MR leave−one−out sensitivity analysis for
'genus.Oxalobacter.id.2978' on 'Crohn's disease || id:ieu−a−30'

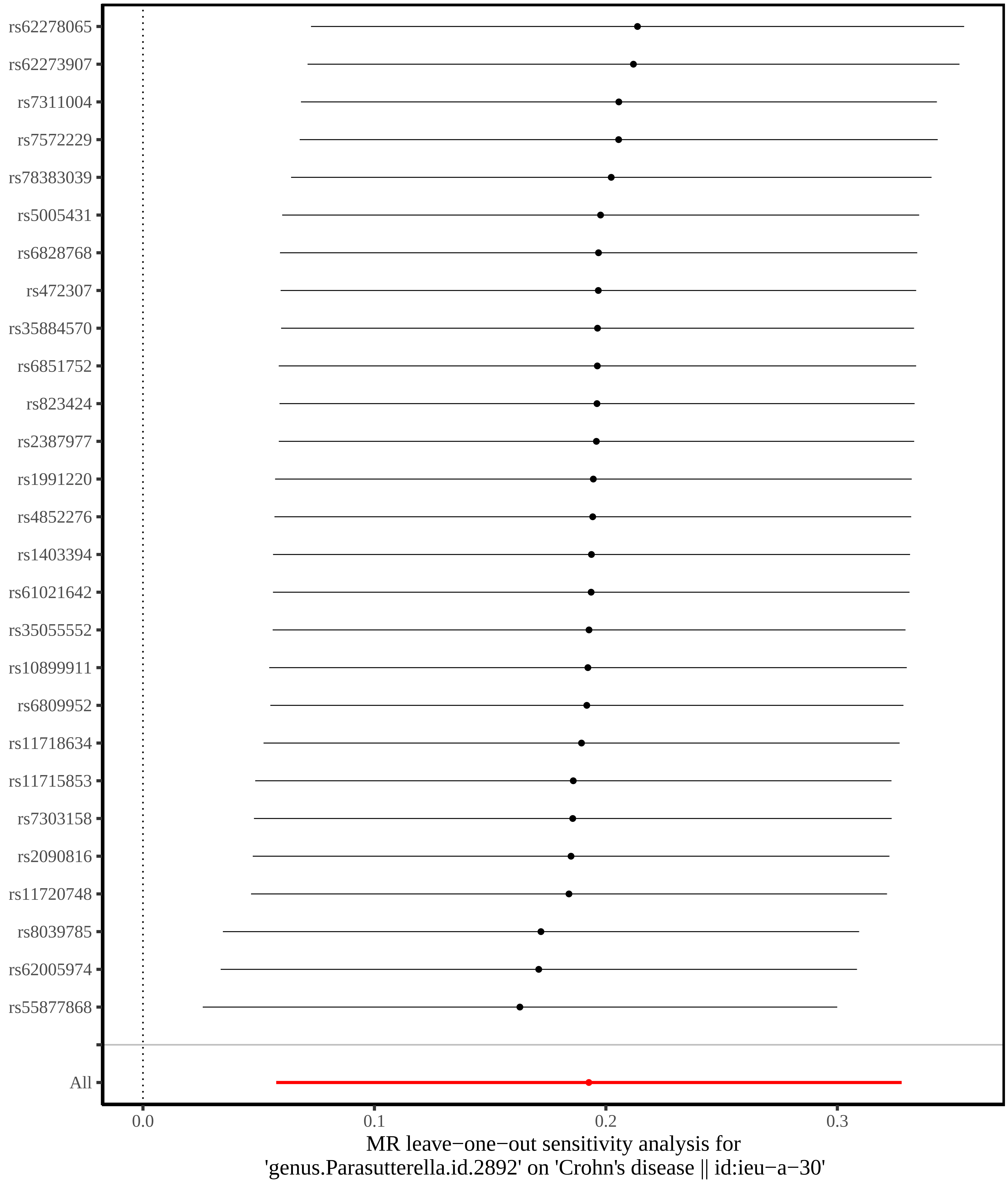

rs62278065
rs62273907
rs7311004
rs7572229
rs78383039
rs5005431
rs6828768
rs472307
rs35884570
rs6851752
rs823424
rs2387977
rs1991220
rs4852276
rs1403394
rs61021642
rs35055552
rs10899911
rs6809952
rs11718634
rs11715853
rs7303158
rs2090816
rs11720748
rs8039785
rs62005974
rs55877868
All
0.0
0.1
0.2
0.3
MR leave−one−out sensitivity analysis for
'genus.Parasutterella.id.2892' on 'Crohn's disease || id:ieu−a−30'

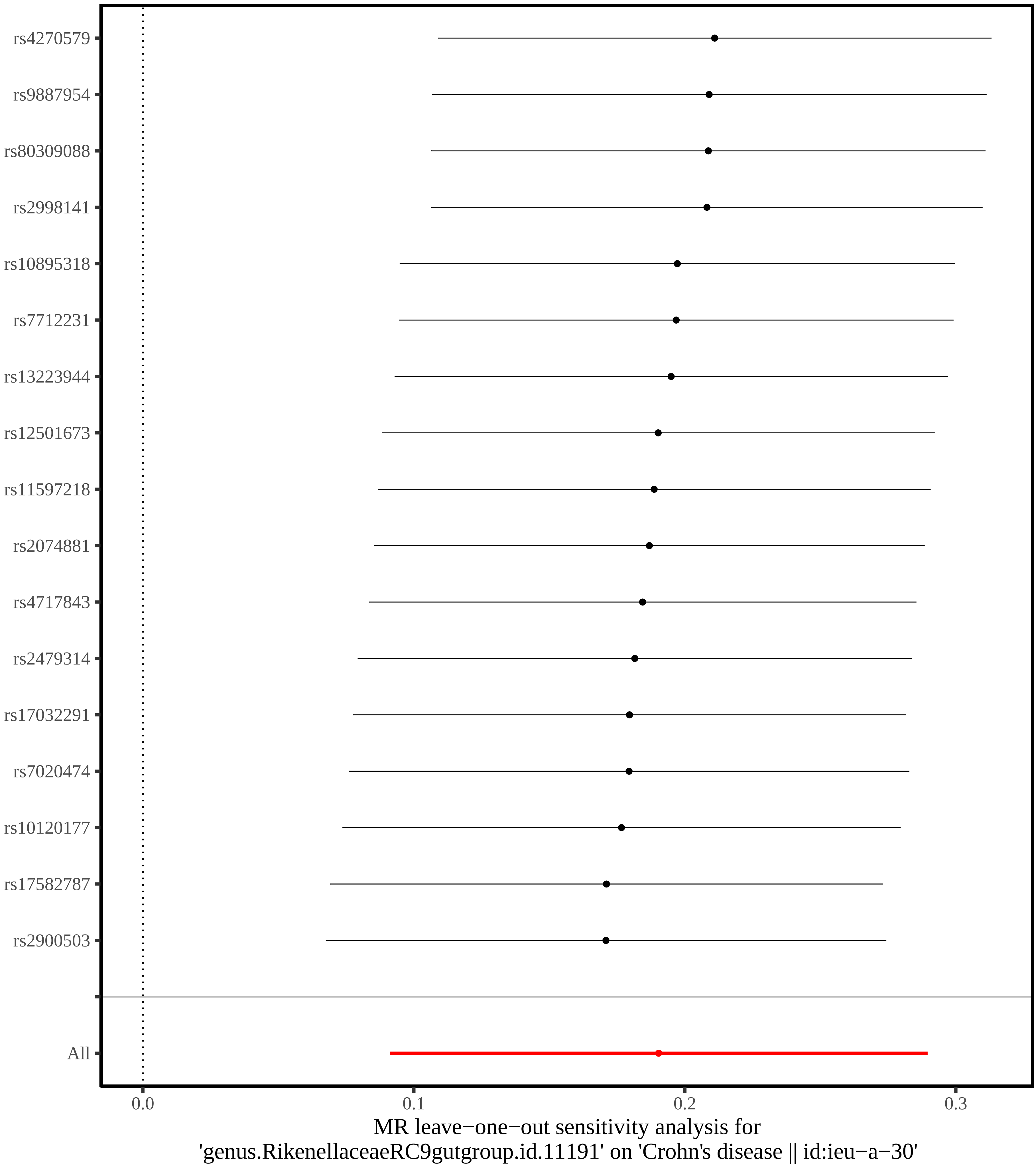
rs4270579
rs9887954
rs80309088
rs2998141
rs10895318
rs7712231
rs13223944
rs12501673
rs11597218
rs2074881
rs4717843
rs2479314
rs17032291
rs7020474
rs10120177
rs17582787
rs2900503
All
0.0
0.1
0.2
0.3
MR leave−one−out sensitivity analysis for
'genus.RikenellaceaeRC9gutgroup.id.11191' on 'Crohn's disease || id:ieu−a−30'

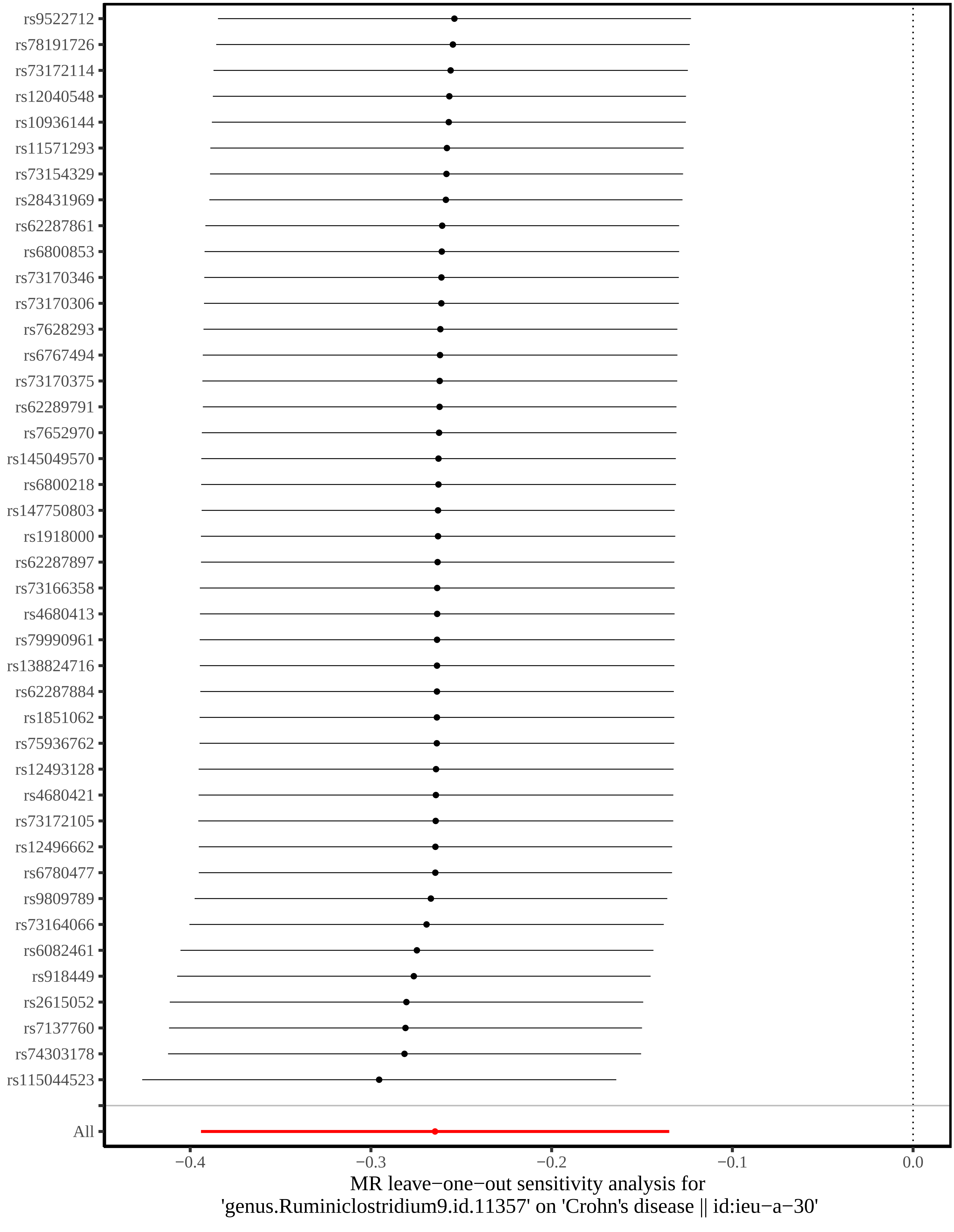
rs9522712
rs78191726
rs73172114
rs12040548
rs10936144
rs11571293
rs73154329
rs28431969
rs62287861
rs6800853
rs73170346
rs73170306
rs7628293
rs6767494
rs73170375
rs62289791
rs7652970
rs145049570
rs6800218
rs147750803
rs1918000
rs62287897
rs73166358
rs4680413
rs79990961
rs138824716
rs62287884
rs1851062
rs75936762
rs12493128
rs4680421
rs73172105
rs12496662
rs6780477
rs9809789
rs73164066
rs6082461
rs918449
rs2615052
rs7137760
rs74303178
rs115044523
All
−0.4
−0.3
−0.2
−0.1
0.0
MR leave−one−out sensitivity analysis for
'genus.Ruminiclostridium9.id.11357' on 'Crohn's disease || id:ieu−a−30'

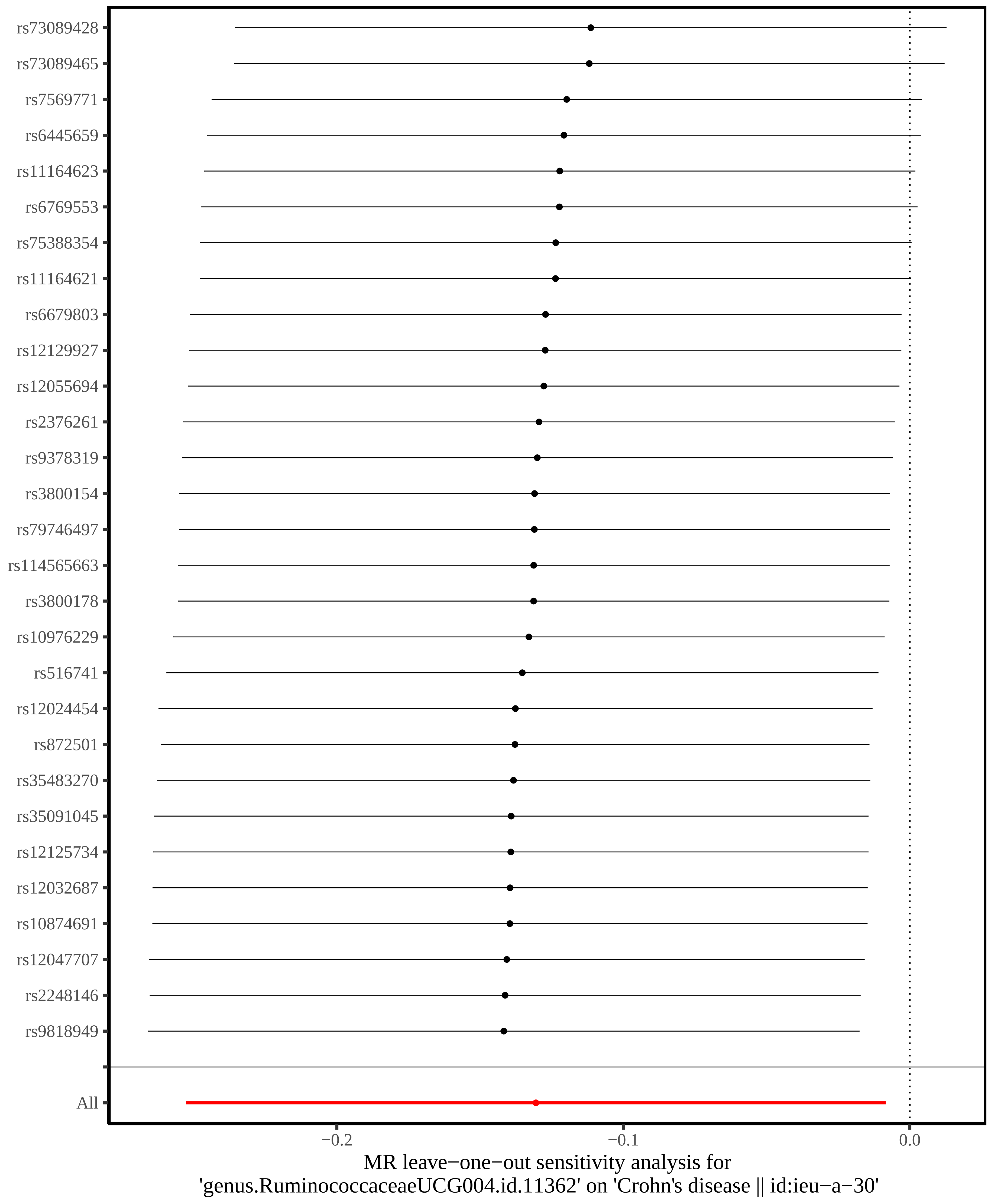
rs73089428
rs73089465
rs7569771
rs6445659
rs11164623
rs6769553
rs75388354
rs11164621
rs6679803
rs12129927
rs12055694
rs2376261
rs9378319
rs3800154
rs79746497
rs114565663
rs3800178
rs10976229
rs516741
rs12024454
rs872501
rs35483270
rs35091045
rs12125734
rs12032687
rs10874691
rs12047707
rs2248146
rs9818949
All
−0.2
−0.1
0.0
MR leave−one−out sensitivity analysis for
'genus.RuminococcaceaeUCG004.id.11362' on 'Crohn's disease || id:ieu−a−30'

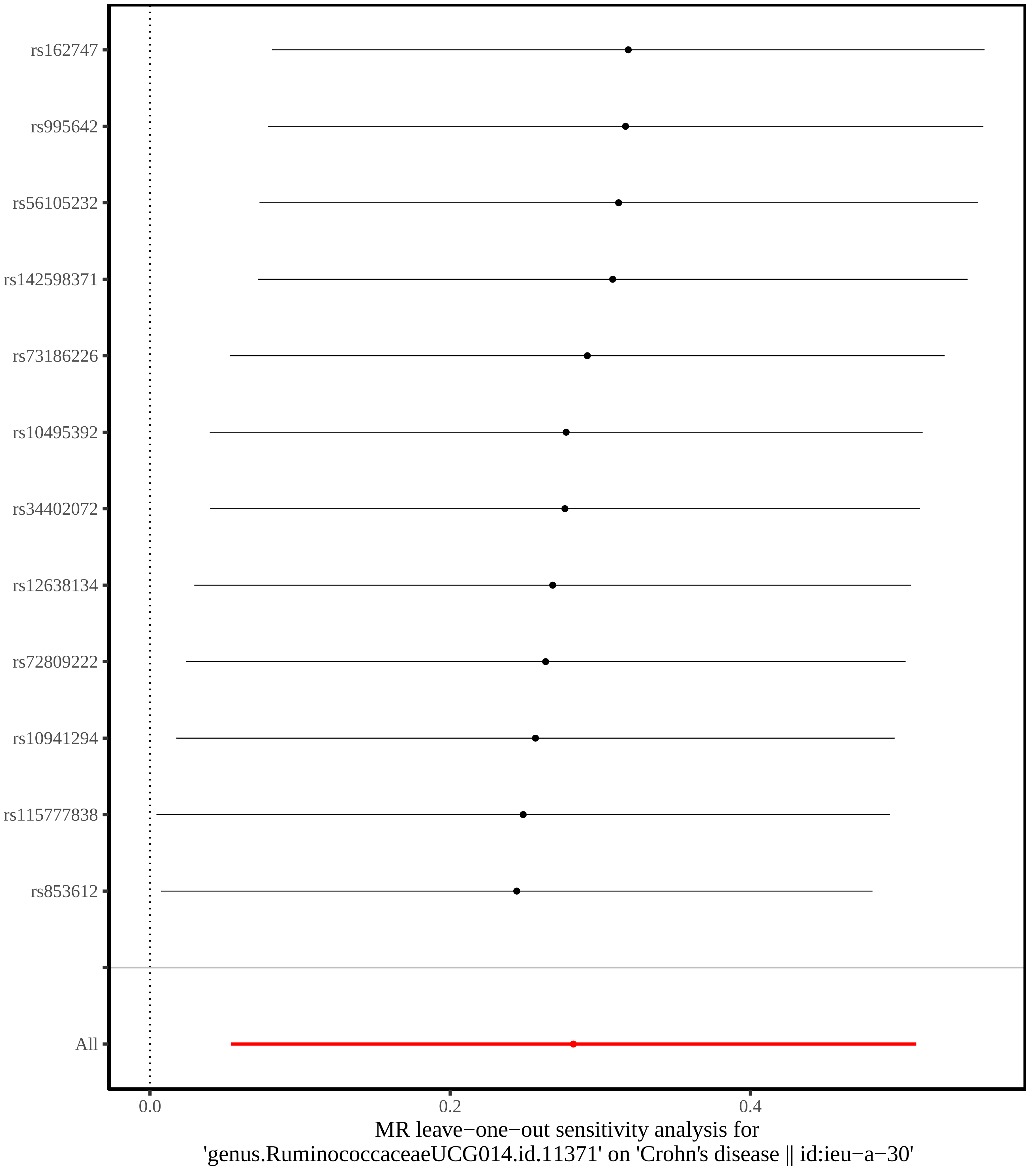
rs162747
rs995642
rs56105232
rs142598371
rs73186226
rs10495392
rs34402072
rs12638134
rs72809222
rs10941294
rs115777838
rs853612
All
0.0
0.2
0.4
MR leave−one−out sensitivity analysis for
'genus.RuminococcaceaeUCG014.id.11371' on 'Crohn's disease || id:ieu−a−30'

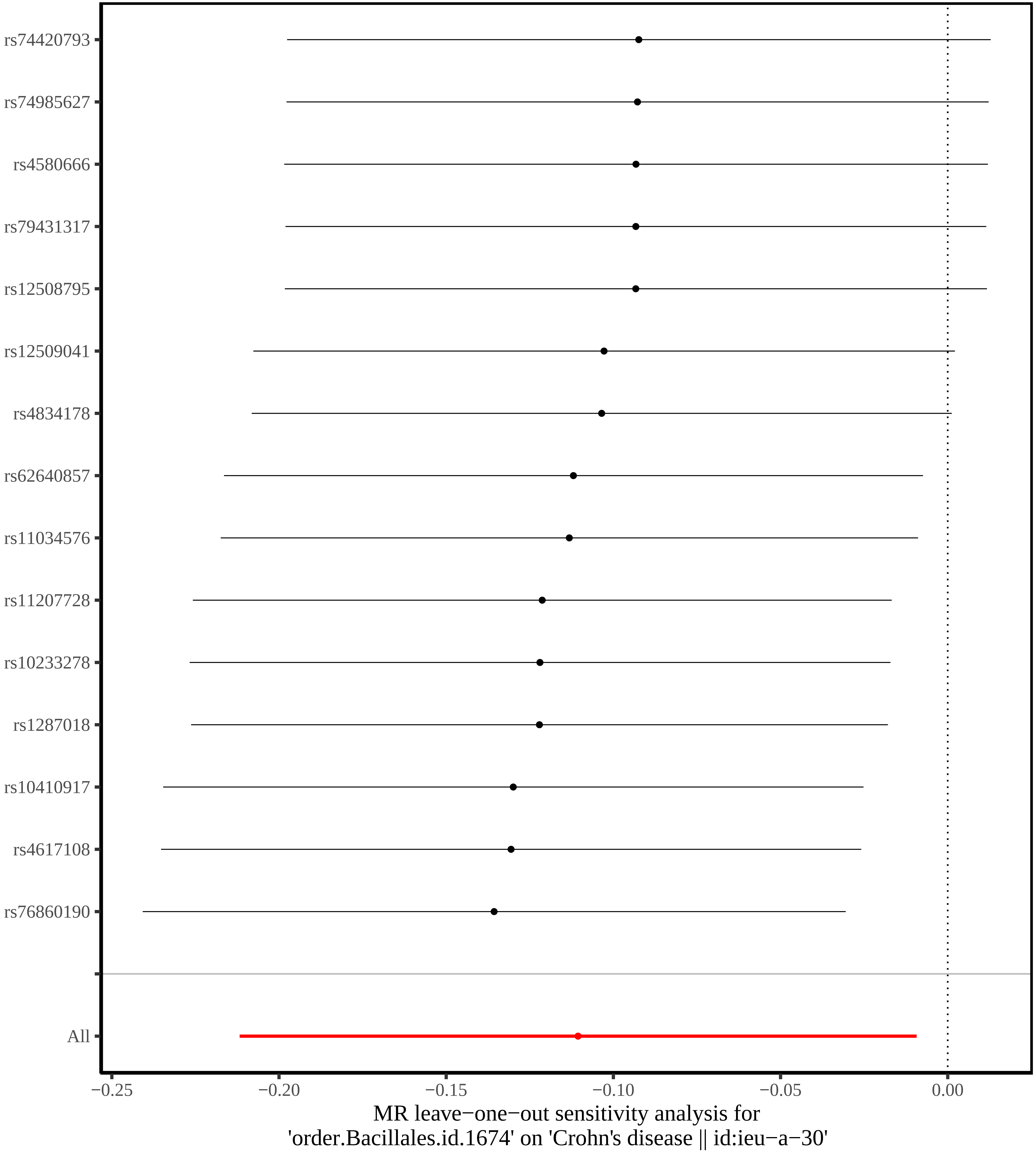
rs74420793
rs74985627
rs4580666
rs79431317
rs12508795
rs12509041
rs4834178
rs62640857
rs11034576
rs11207728
rs10233278
rs1287018
rs10410917
rs4617108
rs76860190
All
−0.25
−0.20
−0.15
−0.10
−0.05
0.00
MR leave−one−out sensitivity analysis for
'order.Bacillales.id.1674' on 'Crohn's disease || id:ieu−a−30'

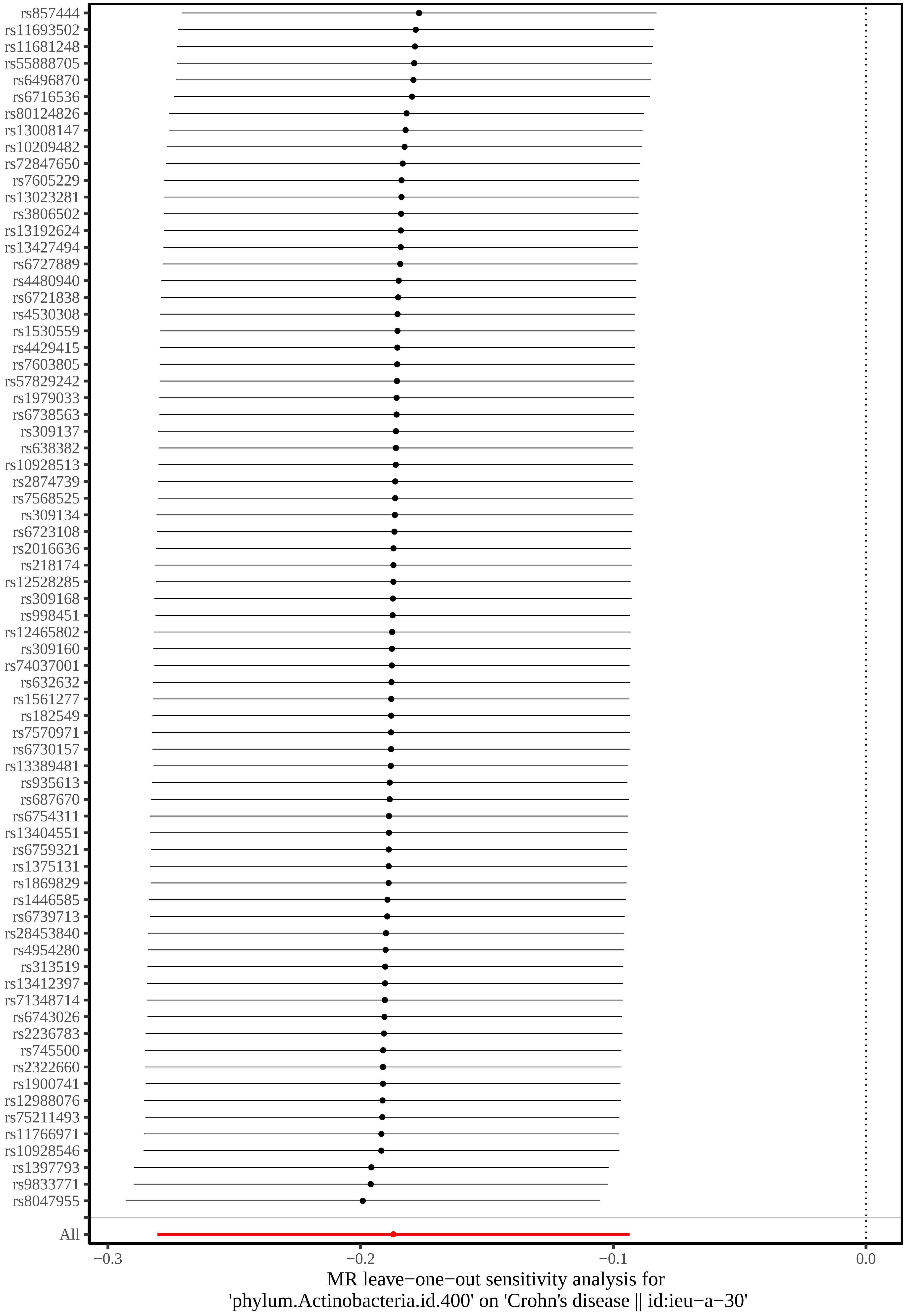
rs857444
rs11693502
rs11681248
rs55888705
rs6496870
rs6716536
rs80124826
rs13008147
rs10209482
rs72847650
rs7605229
rs13023281
rs3806502
rs13192624
rs13427494
rs6727889
rs4480940
rs6721838
rs4530308
rs1530559
rs4429415
rs7603805
rs57829242
rs1979033
rs6738563
rs309137
rs638382
rs10928513
rs2874739
rs7568525
rs309134
rs6723108
rs2016636
rs218174
rs12528285
rs309168
rs998451
rs12465802
rs309160
rs74037001
rs632632
rs1561277
rs182549
rs7570971
rs6730157
rs13389481
rs935613
rs687670
rs6754311
rs13404551
rs6759321
rs1375131
rs1869829
rs1446585
rs6739713
rs28453840
rs4954280
rs313519
rs13412397
rs71348714
rs6743026
rs2236783
rs745500
rs2322660
rs1900741
rs12988076
rs75211493
rs11766971
rs10928546
rs1397793
rs9833771
rs8047955
All
−0.3
−0.2
−0.1
0.0
MR leave−one−out sensitivity analysis for
'phylum.Actinobacteria.id.400' on 'Crohn's disease || id:ieu−a−30'

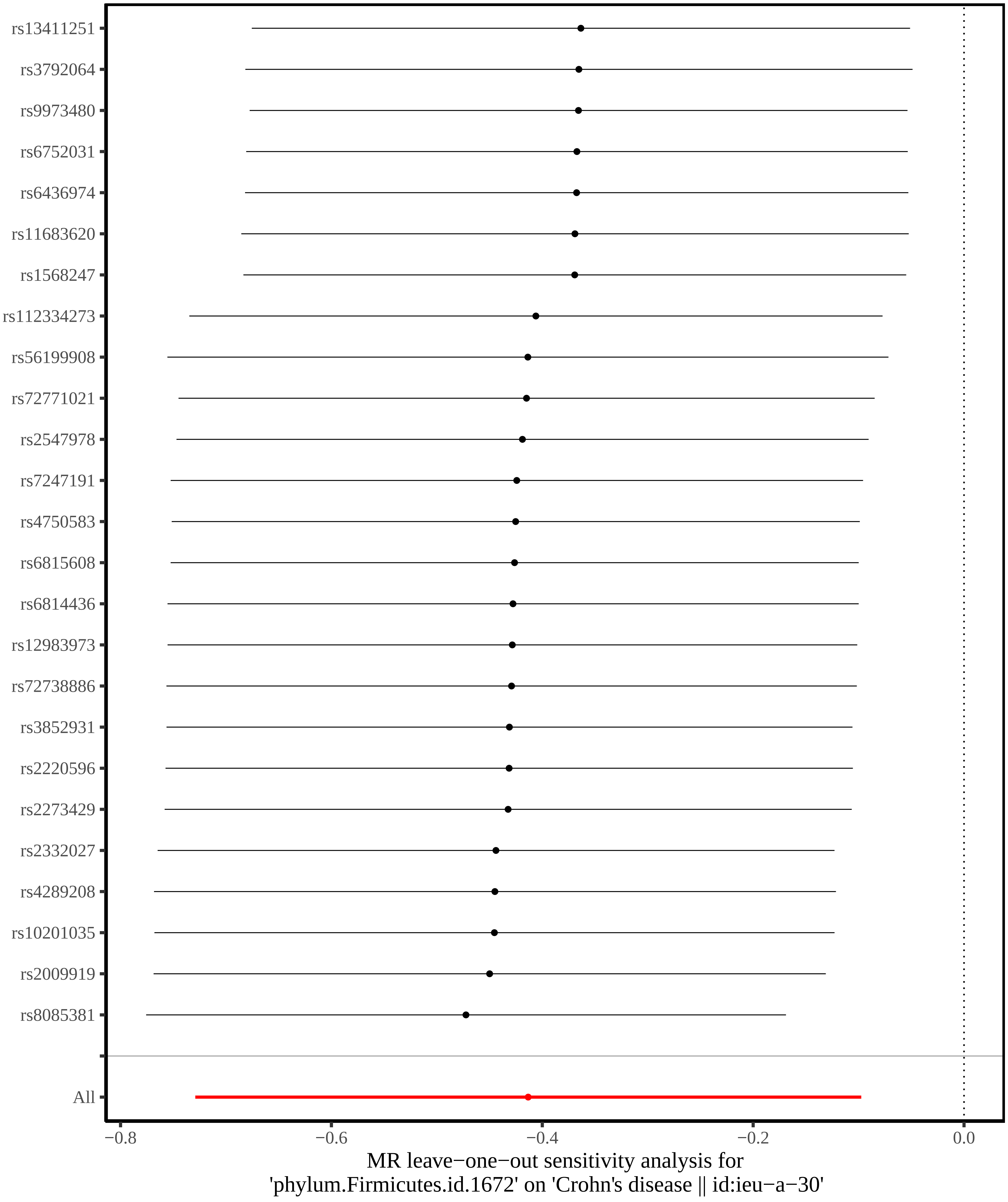
rs13411251
rs3792064
rs9973480
rs6752031
rs6436974
rs11683620
rs1568247
rs112334273
rs56199908
rs72771021
rs2547978
rs7247191
rs4750583
rs6815608
rs6814436
rs12983973
rs72738886
rs3852931
rs2220596
rs2273429
rs2332027
rs4289208
rs10201035
rs2009919
rs8085381
All
−0.8
−0.6
−0.4
−0.2
0.0
MR leave−one−out sensitivity analysis for
'phylum.Firmicutes.id.1672' on 'Crohn's disease || id:ieu−a−30'
