# Supplementary figures and images for: Mendelian randomization integrated with multi-omics analysis identifies TNIK as a key gene in gut microbiota-induced IBD development
Source: Front Immunol. 2025 Nov 18;16:1678444. doi: 10.3389/fimmu.2025.1678444 (PMC12669205; doi:10.3389/fimmu.2025.1678444)

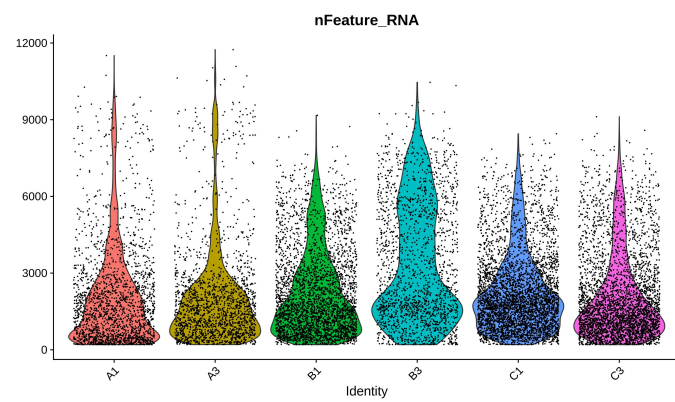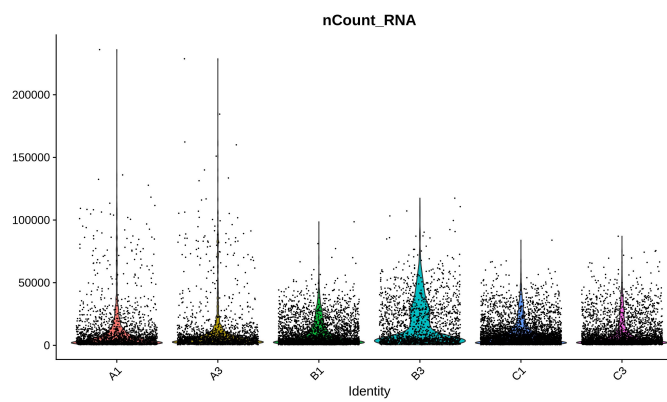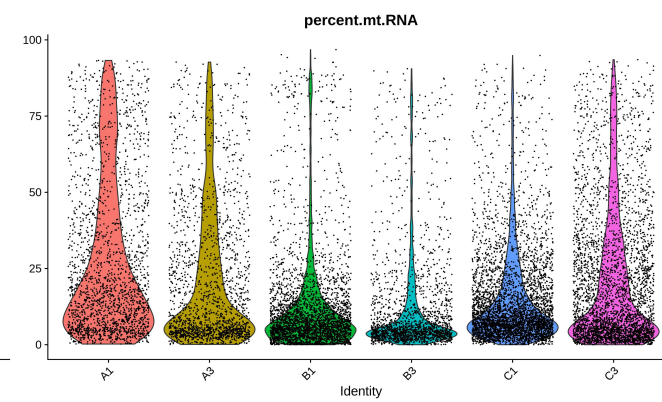

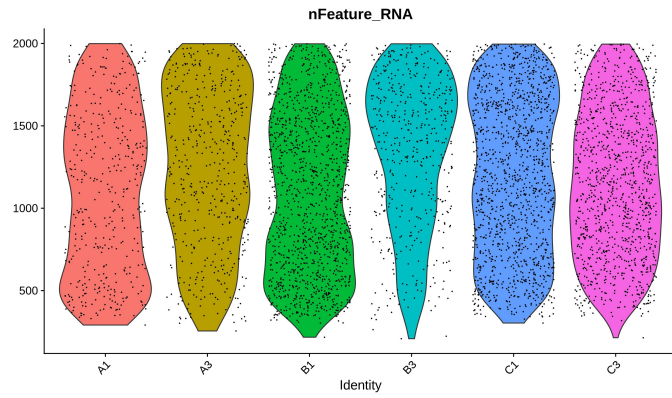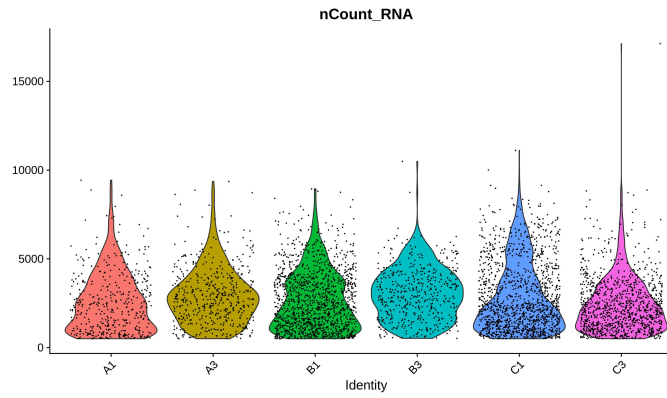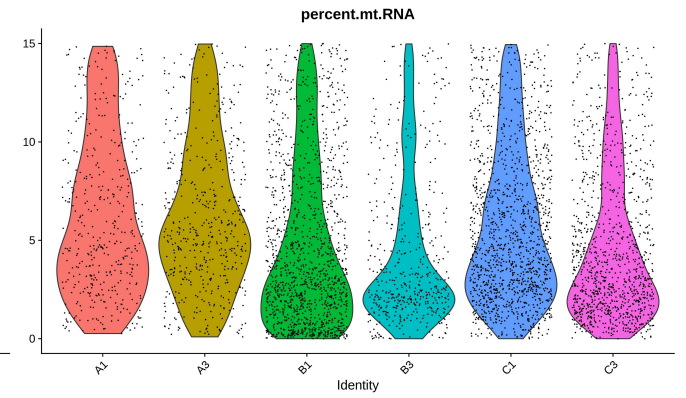

Supplement: Supplementary file 5 [file DataSheet5.pdf]

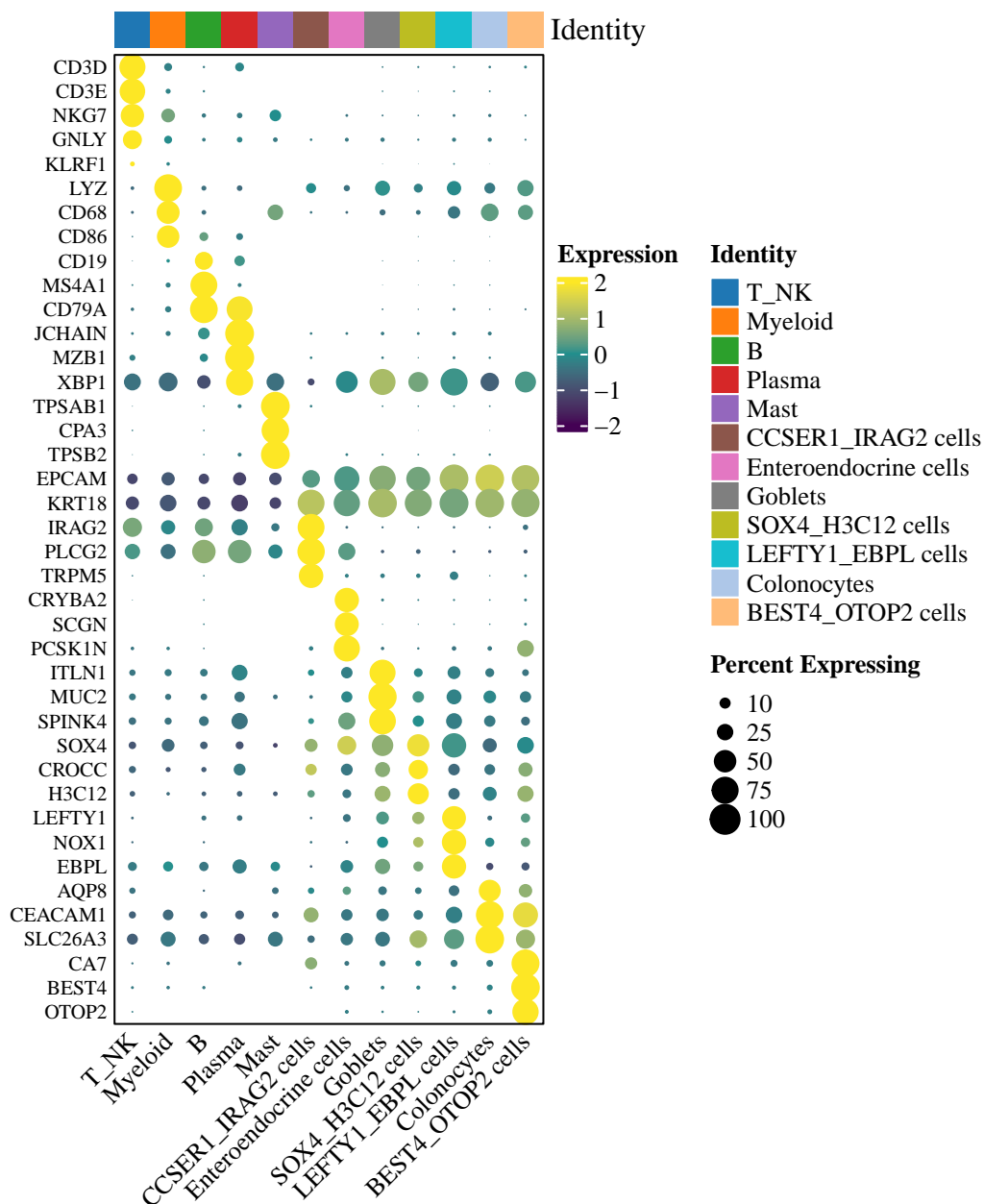

Supplement: Supplementary file 6 [file DataSheet6.pdf]

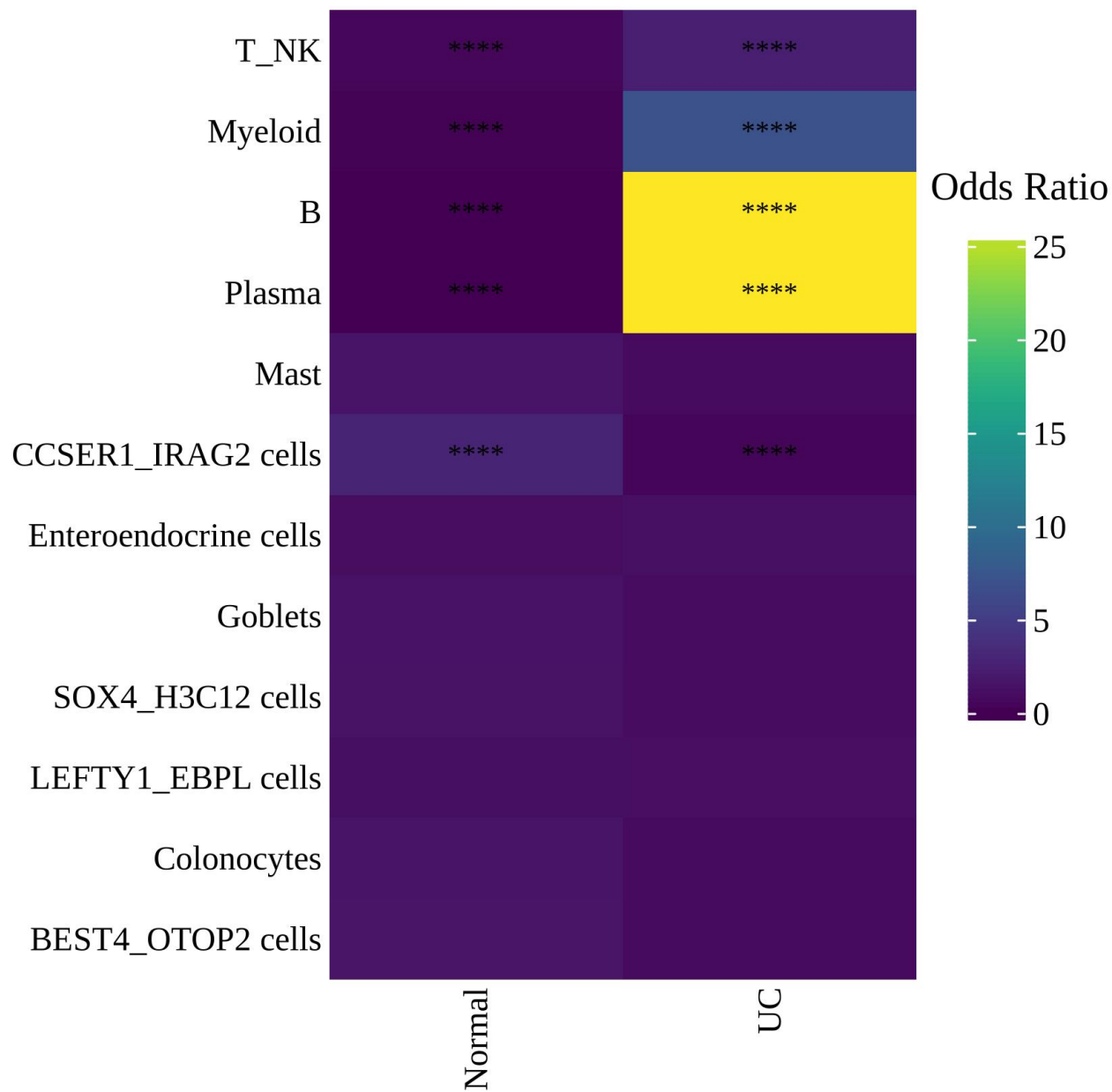

Supplement: Supplementary file 7 [file DataSheet7.pdf]
